# Successive Diels–Alder Cycloadditions of Cyclopentadiene to [10]CPP⊃C_60_: A Computational Study

**DOI:** 10.1021/acs.joc.1c03116

**Published:** 2022-03-23

**Authors:** Gerard Pareras, Sílvia Simon, Albert Poater, Miquel Solà

**Affiliations:** † Institut de Química Computacional i Catàlisi and Departament de Química, Universitat de Girona, c/ Maria Aurèlia Capmany 69, 17003 Girona, Catalonia, Spain; ‡ School of Chemistry, University College Cork, College Road, T12 YN60 Cork, Ireland

## Abstract

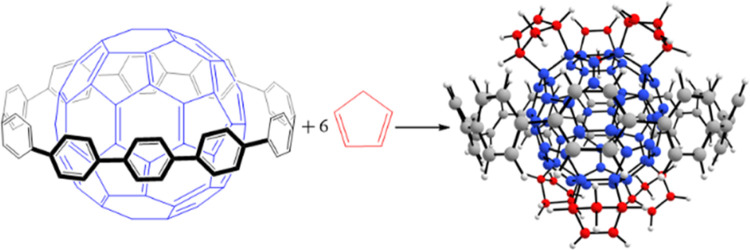

Fullerenes have potential applications in many fields. To reach their full potential, fullerenes have to be functionalized. One of the most common reactions used to functionalize fullerenes is the Diels–Alder cycloaddition. In this case, it is important to control the regioselectivity of the cycloaddition during the formation of higher adducts. In C_60_, successive Diels–Alder cycloadditions lead to the *T*_h_-symmetric hexakisadduct. In this work, we explore computationally using density functional theory (DFT) how the presence of a [10]cycloparaphenylene ring encapsulating C_60_ ([10]CPP⊃C_60_) affects the regioselectivity of multiple additions to C_60_. Our results show that the presence of the [10]CPP ring changes the preferred sites of cycloaddition compared to free C_60_ and leads to the formation of the tetrakisadduct. Somewhat surprisingly, our calculations predict formation of this particular tetrakisadduct to be more favored in [10]CPP⊃C_60_ than in free C_60_.

## Introduction

Potential applications of fullerenes range from materials science (molecular switching devices, magnetic materials, and photovoltaics) to medicinal chemistry.^1−10^ To increase their applicability, fullerenes have to be functionalized.^11^ For instance, the use of C_60_ in molecular heterojunction dye-sensitized solar cells requires to attach a donor group to C_60_ to generate a donor–acceptor (D–A) dyad.^12−14^ Cycloaddition reactions like the [4 + 2] Diels–Alder (DA) cycloadditions,^15,16^ the [3 + 2] Prato reactions,^17,18^ the Bingel cyclopropanations,^19^ or the [2 + 2 + 2] cycloadditions,^20^ among others, are efficient ways to functionalize fullerenes in a regioselective^11^ and, in some cases, enantioselective manner.^21,22^

C_60_ has two types of bonds, namely, the pyracylenic type-[6,6] bond in the ring junction of two fused six-membered rings (six-MRs) and the corannulenic [5,6] bond in the ring junction between five- and six-MRs. Most cycloaddition reactions in empty fullerenes take place in the [6,6] bonds,^23,24^ whereas in endohedral fullerenes, the preference for [6,6] or [5,6] bonds is less clear.^25−29^

Multiple additions to the fullerene cages are also possible depending on the conditions of the reaction.^30−32^ They proceed with a high control of the regioselectivity during the formation of higher adducts. For instance, multiple DA cycloadditions to C_60_ can generate bisadducts, trisadducts, and so on, up to six consecutive additions to finally produce the pseudooctahedral *T*_h_-symmetric hexakisadduct.^33−35^ These multiple DA cycloadditions occur exclusively to the [6,6] bonds of C_60_. In some cases, formed cycloadducts are thermally unstable and can undergo cycloreversion.^35^

In a previous computational study, Solà et al.^36^ discussed the formation of the *T*_h_-symmetric hexakisadduct through multiple DA cycloadditions of 1,3-butadiene to [6,6] bonds of C_60_, concluding that during the successive addition processes enthalpy barriers slightly increase and the exothermicity of the cycloadditions diminishes. In addition, the authors reported that addition of an extra 1,3-butadiene to the hexakisadduct is not possible due to the high energy barrier that has to be surpassed.^36^ Similar results were obtained by Das et al. in the successive DA cycloadditions of 1,3-butadiene to the [6,6] bonds of C_60_ and Li^+^@C_60_.^37^

**Figure 1:**
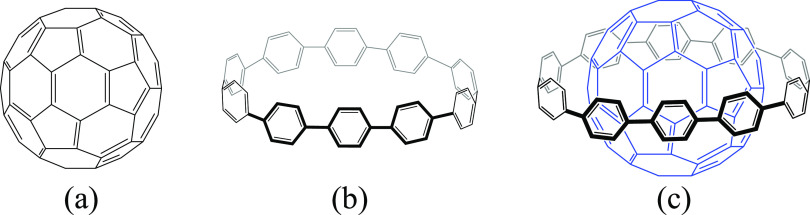
Structures of (a) C_60_, (b) [10]CPP ring (C_60_H_40_), and (c) [10]CPP⊃C_60_.

[n]Cycloparaphenylenes ([n]CPPs) are hoop-shaped π-conjugated molecules in which the *n* paraphenylene units form a cycle.^38−40^ These molecules can act as hosts for fullerenes (see Figure 1).^41,42^ Yamago et al.^43,44^ in 2011 proved that C_60_ can be selectively encapsulated by [10]CPP forming a stable [10]CPP⊃C_60_ system with a binding constant of (2.79 ± 0.03) × 10^6^ L mol^–1^ in toluene. This [10]CPP⊃C_60_ system can act as a D–A dyad in which the charge-transfer process forms the [10]CPP^+^⊃C_60_^–^ system.^45^ A similar charge-transfer process is observed in [10]CPP⊃(C_59_N)_2_⊂[10]CPP, a bis(azafullarene) (C_59_N)_2_ system complexed by two [10]CPP. The first [10]CPP macrocycle stabilizes the binding of the second by maximizing π–π, CH−π, and attractive London dispersion interactions.^46^ Same type of interactions are key in the synthesis of [10]cycloparaphenylene-fullerene [2]rotaxanes^47^ and in the recently synthesized figure-of-eight nanohoop that forms peanut-like 1:2 host–guest complexes with C_60_ or C_70_.^48^

Very recently, Ribas et al.^49^ reported the synthesis of a three-shell, matryoshka-like complex in which C_60_ inside a [10]CPP is in turn encapsulated inside a self-assembled nanocapsule. Bingel cyclopropanation to this matryoshka-like complex leads to the selective formation of a particular fullerene bisadduct. They also discussed how [n]CPPs can be used in combination with nanocages to purify and regioselectively functionalize fullerenes and endohedral metallofullerenes.^50^ Inspired by these results, the main goal of the present work is to computationally investigate how the presence of the [10]CPP ring encapsulating C_60_ ([10]CPP⊃C_60_) affects the regioselectivity of successive DA cycloadditions of cyclopentadiene to C_60_. We anticipate here that our results show that in the presence of the [10]CPP, the pentakis and hexakisadducts of C_60_ are not formed.

## Results and Discussion

**Scheme 1:**
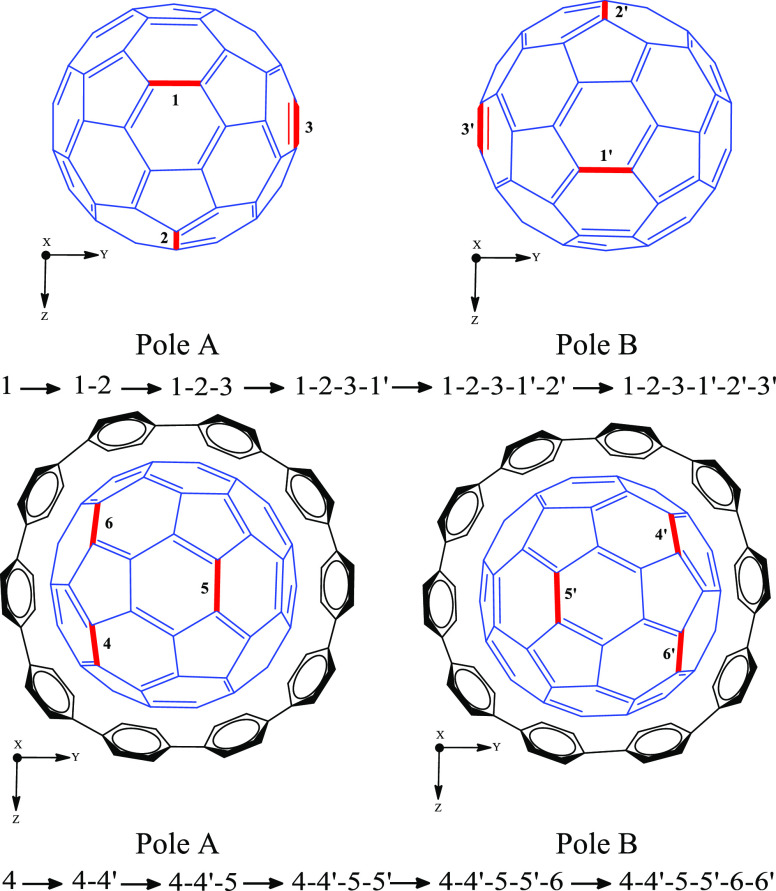
Top. Path Followed When Performing the Six Different Cycloadditions in Free C_60_ to Reach the *T*_h_ Hexakisadduct. Bottom. Path Followed When Performing the Six Different Cycloadditions in [10]CPP⊃C_60_ to Reach the *C*_2h_ Hexakisadduct

In the optimized [10]CPP⊃C_60_ species, the [10]CPP ring is located in the equator of C_60_ where the π–π interactions are more favorable, dividing C_60_ into two equal poles. As mentioned before, in the DA reaction, the cyclopentadiene will attack the [6,6] bonds. We have assumed that the attacks occur sequentially over the freest [6,6] bonds. Considering that the equatorial plane (where the [10]CPP ring is located) is the most hindered region of C_60_ and taking into account the existence of two well-defined poles, we have assumed that additions of the second, fourth, and sixth cyclopentadiene will happen exactly in the same position of first, third, and fifth but on the opposite pole, i.e., path 4 → 4-4′ → 4-4-5 → 4-4′-5-5′→ 4-4′-5-5′-6 → 4-4′-5-5′-6-6′ (see Scheme 1 bottom). For the bisaddition, we also considered formation of the 4-5′ and 4-6′ bisadducts. These two bisadducts are the main products of the Bingel–Hirsch bisaddition to [10]CPP⊃C60 species.^51^ Results given in Table S1 of the SI indicate that the 4-4′ DA bisadduct is about 2 kcal/mol more stable than the 4-5′ and 4-6′ and that the Gibbs energy barrier for the DA leading to the 4-4′ bisadduct is at least 2 kcal/mol lower than that generating the 4-5′ and 4-6′ bisadducts. Therefore, bisadducts 4-5′ and 4-6′ can be discarded as the main outcome in this DA bisaddition. Moreover, for each addition, there are two possible cycloadducts that correspond to the usual *endo* and *exo* attacks in DA cycloadditions. For all DA cycloadditions to [10]CPP⊃C_60_, we have considered the two possible attacks and we report here only the attack with the lowest Gibbs reaction energy. On the other hand, for the addition to free C_60_, we have followed the addition path 1 → 1-1′ → 1-1′-2 → 1-1′-2-2′ → 1-1′-2-2′-3 → 1-1′-2-2′-3-3′ (see Scheme 1 top), which is the thermodynamically most favorable according to Solà et al.^36^

**Figure 2:**
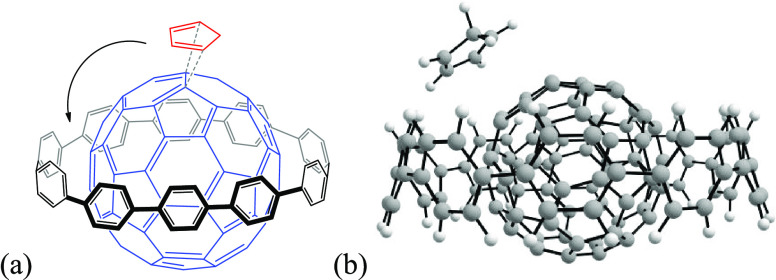
(a) Representation of the rotation produced during the optimization of the first reactant complex and (b) final optimized geometry of the first reactant complex.

**Figure 3:**
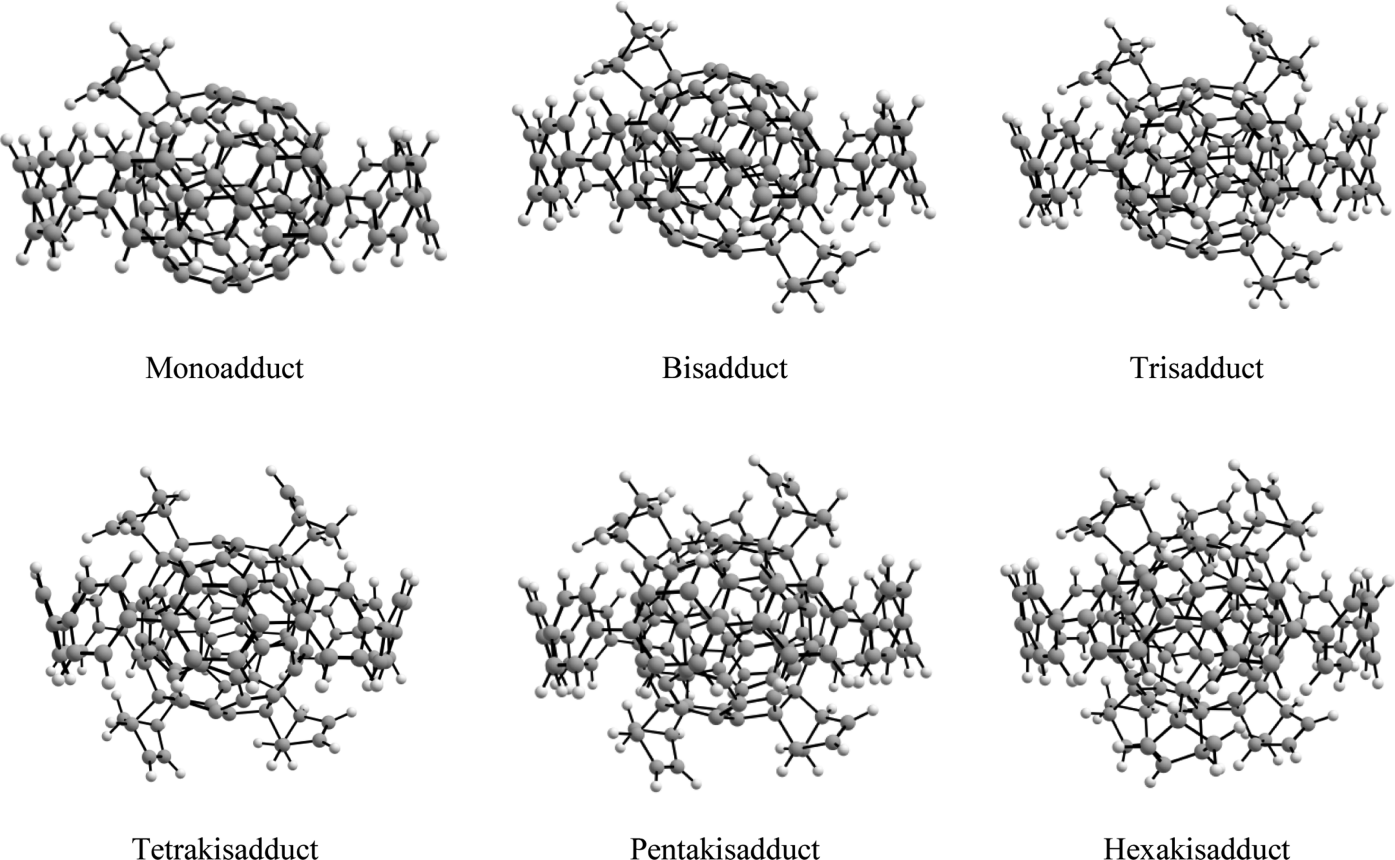
Optimized geometries of the [10]CPP⊃C_60_ products for the successive Diels–Alder reactions, from the first attack until the sixth.

The DA cycloaddition of cyclopentadiene to [10]CPP⊃C_60_ starts with the formation of a reactant complex (RC) where the cyclopentadiene weakly interacts with [10]CPP⊃C_60_, followed by the transition state (TS) of the [6,6]-attack, and finally, the product with the cyclopentadiene already attached to [10]CPP⊃C_60_. The first addition of the cyclopentadiene is considered to happen on the [6,6] bond at the very top of the C_60_ cage, being this position the freest as it is the one located furthest from the [10]CPP ring (see Figure 2). The reaction mechanism under the study begins with the RC where the cyclopentadiene interacts weakly with the [10]CPP⊃C_60_. In the RC formed, the cyclopentadiene not only interacts with C_60_ but also with the [10]CPP ring. The optimized geometry for the RC of the first insertion shows how C_60_ and cyclopentadiene rotate until the cyclopentadiene is interacting with both C_60_ and the [10]CPP unit to maximize dispersion interactions (see Figure 2). Indeed, the four first additions occur close to the [10]CPP ring (Figure 3), which stabilizes RCs and TSs through dispersion interactions (vide infra).

Consecutively, the second insertion will happen on the same [6,6] bond but on the opposite pole. The methodology followed to study the subsequent cycloadditions is the same as for the first two insertions. Figure 3 collects the six products obtained by successive cycloadditions until the hexakisadduct is formed. The attacked positions differ from the ones attacked in free C_60_ (see Scheme 1).^36^ In the following sections, we discuss in terms of energy values the path followed until the hexakisadduct is generated as well as the role of the [10]CPP ring over this process. For the sake of comparison, we also study the same DA cycloadditions on the same [6,6] bonds of the free C_60_ system without the [10]CPP ring.

**Figure 4:**
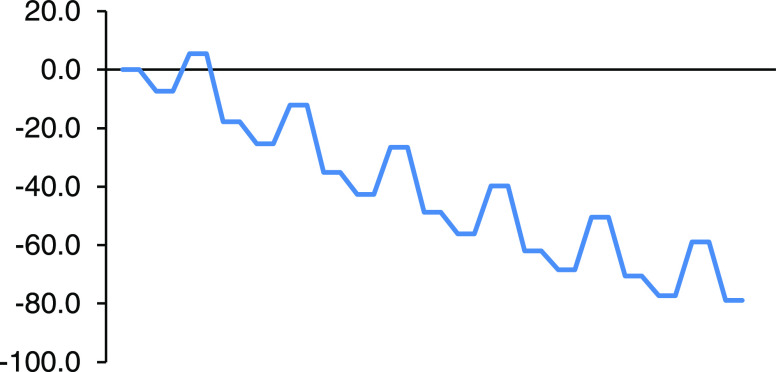
Enthalpy profile (kcal/mol) in toluene solution for the successive additions of cyclopentadiene to [10]CPP⊃C_60_.

**Figure 5:**
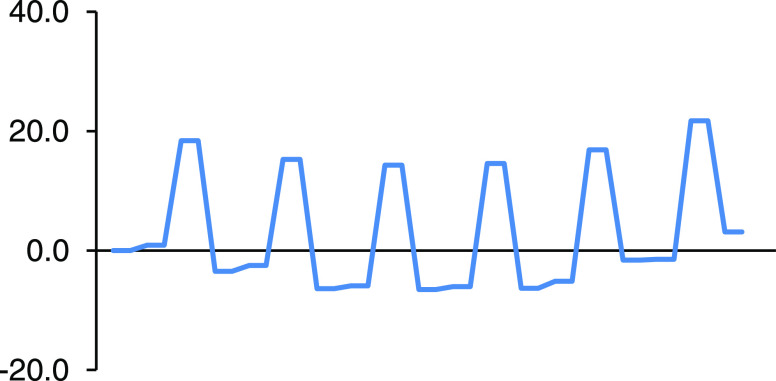
Gibbs energy profile (kcal/mol) in toluene solution for the successive additions of cyclopentadiene to [10]CPP⊃C_60_.

Table 1 collects relative enthalpies and Gibbs energies of the RCs, TSs, and products of the different successive cycloadditions leading to the formation of the *C*_2h_ hexakisadduct system in [10]CPP⊃C_60_. Both sets of relative energy values are also represented in the energy profiles of Figures 4 and 5.

**Table 1:** Relative Energy Values for All of the Reaction Steps Through the Six Cycloadditions, Collected Enthalpies, and Gibbs Energies (in kcal/mol) in Toluene Solution

structure^a^	Δ*H*	Δ*G*
[10]CPP⊃C_60_+cyclopentadiene	0.00	0.00
RC 1^b^	–7.36	0.92
TS 1^c^	5.44	18.40
product 1	–17.79	–3.44
RC 2	–25.33	–2.52
TS 2	–12.10	15.28
product 2	–35.12	–6.38
RC 3	–42.63	–5.92
TS 3	–26.52	14.30
product 3	–48.69	–6.50
RC 4	–56.21	–6.06
TS 4	–39.82	14.56
product 4	–61.96	–6.30
RC 5	–68.46	–5.15
TS 5	–50.53	16.84
product 5	–70.49	–1.63
RC 6	–77.36	–1.44
TS 6	–58.88	21.71
product 6	–78.93	3.10

a Order of addition is 4 → 4-4′ → 4-4′-5 → 4-4′-5-5′ → 4-4′-5-5′-6 → 4-4′-5-5′-6-6′ (Scheme 1).

b RC = Reactant complex.

c TS = transition state.

Table 2 lists the activation energies and reaction energies derived from the enthalpies and Gibbs energies of Table 1. Enthalpies and Gibbs energies provide different trends. As to enthalpies, we find that reaction enthalpies are exothermic for all additions. After each addition, the individual enthalpy barriers (enthalpy difference between RC and TS) collected in Table 2 increase, whereas the exothermicity of the reaction decreases (same observations were made by Solà et al.^36^ when discussing the formation of the *T*_h_ hexakisadduct in C_60_). Although the first insertion needs to overcome an enthalpy barrier of only 12.8 kcal/mol, the last one requires 18.5 kcal/mol, an increase of around 6 kcal/mol (see Table 2). Reaction enthalpies decrease from −10.4 kcal/mol in the first insertion until −1.6 kcal/mol in the last one.

**Table 2:** Activation Energies and Reaction Energies for Successive Additions to [10]CPP⊃C_60_^a^

	enthalpy		Gibbs energy	
addition^b^	activation energy	reaction energy	activation energy	reaction energy
1	12.80	–10.43	17.48	–4.36
2	13.23	–9.79	17.79	–3.86
3	16.10	–6.06	20.23	–0.58
4	16.38	–5.75	20.62	–0.24
5	17.93	–2.04	21.98	3.52
6	18.48	–1.57	23.15	4.54

a (Enthalpies and Gibbs Energies in kcal/mol) in Toluene Solution.

b Order of addition is 4 → 4-4′ → 4-4-5 → 4-4′-5-5′ → 4-4′-5-5′-6 → 4-4′-5-5′-6-6′ (Scheme 1).

Gibbs reaction energies and Gibbs energy barriers collected in Table 2 do not change their trends compared to enthalpies, although they are higher by roughly 5–6 kcal/mol. Not surprisingly for entropic reasons, the energy barrier increases and the exothermicity decreases when considering Gibbs energies. Although trends given by Gibbs energies are the same as enthalpies, i.e., we observe the same increase of the energy barriers and a decrease of the reaction energies with successive additions, there are some relevant differences. The most important one is that Gibbs reaction energies reach positive values after the fourth insertion, indicating that the formation of pentakis- and hexakisadducts is not thermodynamically favored (see Figure 5) and can therefore be assumed that the reaction will stop on the tetrakisadduct system. However, we expect a complex equilibrium between reactants and the different possible products. In particular, because the Gibbs energies of the pentakis- and hexakisadducts are 3.5 kcal/mol and 4.5 kcal/mol above their respective reactant complexes and their relative Gibbs energies with respect to separated reactants are −1.6 and 3.1 kcal/mol, respectively, it is likely that if the DA cycloaddition is carried out in the presence of a large excess of cyclopentadiene the reaction could afford the formation of minor quantities of the pentakis- and hexakisadducts. The increase in the energy barriers with successive additions is attributed mainly to the increase of the lowest unoccupied molecular orbital (LUMO) energy of the fullerenic cage (vide infra).

To unveil the effect of the presence of the [10]CPP, the same attacks studied for the [10]CPP⊃C_60_ species have also been studied removing the [10]CPP unit.

**Figure 6:**
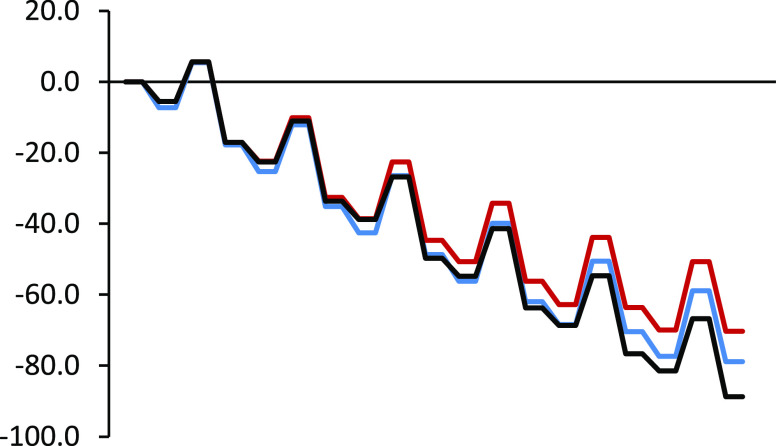
Enthalpy profile (kcal/mol) in toluene solution of the successive DA cycloadditions of cyclopentadiene to [10]CPP⊃C_60_ in blue color, C_60_ in red color, and C_60_ with the successive Diels–Alder cycloadditions studied by Solà et al.^36^ in black color.

**Figure 7:**
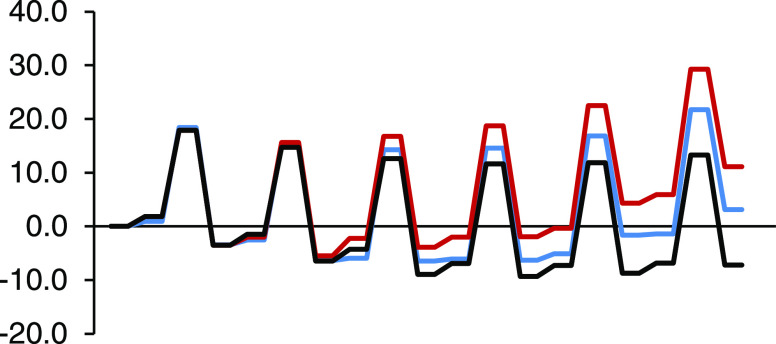
Gibbs energy profile (kcal/mol) in toluene solution of the successive DA cycloadditions of cyclopentadiene to [10]CPP⊃C_60_ in blue color, C_60_ in red color, and C_60_ with the successive Diels–Alder cycloadditions studied by Solà et al.^36^ in black color.

For the sake of comparison, the energy profiles for the six cycloadditions to the same [6,6] bonds of C_60_ and [10]CPP⊃C_60_ values have been compared in Figures 6 and 7. In comparison with [10]CPP⊃C_60_ values, relative energies of all RC, TSs, and products are lower in [10]CPP⊃C_60_ as compared to C_60_ because of better dispersion interactions (see Table S2). In Table 3, the activation energies and reaction energies for each individual DA reaction are included. Pang and Wilson^52^ found that the activation energy for the first DA reaction of C_60_ and cyclopentadiene in high-pressure liquid chromatography is 6.9 kcal/mol, whereas Giovane et al.^53^ reported an activation energy of 26.7 ± 2.2 kcal/mol for the corresponding retro-DA cycloaddition in tetrachloroethane. From the combination of these two numbers, one can estimate the reaction energy to be −19.8 ± 2.2 kcal/mol. These experimental values have to be compared with the Δ*H*^⧧^ = 11.2 kcal/mol and Δ*H*_r_ = −11.6 kcal/mol obtained in our study (Table 3). These results show that our calculated activation energies may be somewhat overestimated and the reaction energies somewhat underestimated. On the other hand, Ueno et al.^54^ found that the DA of 1,3-cyclohexadiene with C_60_ has an activation barrier of 16.1 kcal/mol in dichloromethane. Activation energies for the first additions over C_60_ are slightly lower than the activation energies for [10]CPP⊃C_60_, whereas for additions 5–6 are somewhat higher. In detail, this difference is only about 1 kcal/mol for additions 1–3 in favor of C_60_, while for additions 5 and 6, they are slightly higher for the C_60_ system, also around 1 kcal/mol. The fact that the fifth and sixth additions of cyclopentadiene to C_60_ are more favorable in [10]CPP⊃C_60_ than in pristine C_60_ is likely to be the result of an overestimation of the dispersion interactions by the D3(BJ) method. Reaction enthalpy values follow a similar trend, and thus, insertions 1 to 3 are less exothermic for [10]CPP⊃C_60_. However, the two last insertions become more exothermic for [10]CPP⊃C_60_. In general terms, although the differences found due to the presence of the [10]CPP are relatively small, the [10]CPP ring tends to difficult additions 1–3 and favor additions 4–6.

**Table 3:** Activation Energies and Reaction Energies for Each Addition over the C_60_ Structure^a^

	enthalpy		Gibbs energy	
addition^b^	activation energy	reaction energy	activation energy	reaction energy
1	11.15	–11.60	16.04	–5.34
2	12.19	–10.27	17.67	–3.43
3	15.90	–6.15	18.98	–1.67
4	16.44	–5.59	20.69	0.03
5	18.89	–0.82	22.86	4.70
6	19.35	–0.31	23.39	5.23

a (Enthalpies and Gibbs Energies in kcal/mol) in Toluene Solution.

b Order of addition is 4 → 4-4′ → 4-4′-5 → 4-4′-5-5′ → 4-4′-5-5′-6 → 4-4′-5-5′-6-6′ (Scheme 1).

Gibbs energy values again show that the reaction would stop at insertions 3-4 resulting in the formation of the tris- and tetrakisadducts in equilibrium. Interestingly, the increase in the Gibbs energy barriers and the destabilization of the products after the third or fourth addition is greater for C_60_ than for [10]CPP⊃C_60_. The higher destabilization for the last additions in C_60_ is due to the fact that the successive cycloadditions over the poles happen in a very reduced space. Surprisingly, this detrimental effect is less marked in [10]CPP⊃C_60_ despite one would expect a higher hindrance due to the presence of the [10]CPP ring, which should be translated into a destabilization of the TSs and final products. However, for the fifth and sixth additions, dispersion interactions previously discussed between the cyclopentadiene and the [10]CPP ring stabilize somewhat more the TSs than the RCs, favoring the additions to [10]CPP⊃C_60_ as compared to those to C_60_. The final conclusion is that the multiple cycloadditions to [10]CPP⊃C_60_ will stop at the fourth addition for thermodynamic reasons and that a mixture of bis-, tris-, and tetrakisadducts will be generated according to our calculations. The exact product distribution will depend on the initial concentration of cyclopentadiene and [10]CPP⊃C_60_. Let us mention here that the successive DA cycloadditions to free C_60_ do not follow the same path as in the case of [10]CPP⊃C_60_ (see Scheme 1). Following the most stable thermodynamic path according to the work by Solà et al.,^36^ i.e., the path 1 → 1-1′ → 1-1′-2 → 1-1′-2-2′ → 1-1′-2-2′-3 → 1-1′-2-2′-3-3′ in Scheme 1, one finds that the formation of the *T*_h_ hexakisadduct is possible although the most stable cycloadduct is the pentakisadduct (see Tables S2, S3 and Figures 6 and 7).

To get a deeper insight into the successive DA cycloadditions to [10]CPP⊃C_60_ and C_60_, the deformation and interaction energies have been studied for each RC and TS. One can consider the energy of a given complex as the sum of the deformation energy (energy required to deform the fragments to the geometry they have in the complex that is always positive) and interaction energy (energy usually released as a result of the interaction between deformed fragments in the formation of the complex). Deformation and interaction energies for the TSs of the cycloadditions to [10]CPP⊃C_60_ and C_60_ are collected in Table 4, whereas those of the RCs are given in the SI (Table S4). Two deformation energies have been considered, *i.e*., the deformation of [10]CPP⊃C_60_ or C_60_ (*E*_def1_) and the deformation of cyclopentadiene (*E*_def2_), being the total deformation energy the sum of both individual deformation energies (*E*_def_ = *E*_def1_ + *E*_def2_).

**Table 4:** Deformation and Interaction Energies (kcal/mol) in Toluene Solution of the Transition States of the Different Diels–Alder Cycloadditions of Cyclopentadiene to [10]CPP⊃C_60_ and C_60_

a	[10]CPP⊃C_60_ transition states					C_60_ transition states				
addition	*E*_def1_	*E*_def2_	*E*_def_	*E*_int_	*E*_def+Eint_	*E*_def1_	*E*_def2_	*E*_def_	*E*_int_	*E*_def+Eint_
1	8.7	18.5	27.2	–22.5	4.7	7.5	18.2	25.8	–20.9	4.9
2	8.0	19.0	27.0	–22.1	4.9	7.8	18.8	26.6	–20.4	6.2
3	8.3	20.2	28.5	–20.5	8.1	8.1	19.8	27.9	–18.6	9.3
4	8.3	20.4	28.7	–20.4	8.3	8.3	19.9	28.2	–18.4	9.8
5	9.4	18.5	28.0	–17.2	10.8	8.2	19.1	27.4	–15.6	11.8
6	9.3	19.0	28.3	–17.4	10.9	8.3	19.4	27.7	–15.5	12.3

a Order of addition is 4 → 4-4′ → 4-4′-5 → 4-4′-5-5′ → 4-4′-5-5′-6 → 4-4′-5-5′-6-6′ (Scheme 1).

The deformation of the TSs observed in fragments 1 is mainly due to changes in the attacked [6,6] bond. In [10]CPP⊃C_60_, this energy is slightly higher as the [10]CPP ring also shows a small deformation to allow the cyclopentadiene approximation. The main deformation is that of the cyclopentadiene; however, this is energetically similar for [10]CPP⊃C_60_ and C_60_ if we consider the same attacked bonds. The main difference between [10]CPP⊃C_60_ and C_60_ stems from the interaction energy, which is always around 2 kcal/mol more stable for the first one. As previously observed, the energy of the TSs increases after each addition. Thanks to the information in Table 4, this increase can be attributed to the fact that both systems have to be slightly more deformed and that the interaction energy is reduced after each addition. The increase of the energy barrier for successive cycloadditions is somewhat smoother for [10]CPP⊃C_60_ than for C_60_ (considering the same attacks) not because of the stabilization due to better π(HOMO_cyclopentadiene_)-π*(LUMO_cage_) (vide infra) but due to the dispersion interactions present in [10]CPP⊃C_60_ as compared to C_60_.

Finally, the frontier molecular orbitals (HOMO and LUMO) have been also studied for each reaction step and for both systems. As already indicated by Solà et al.,^36^ after each addition, LUMO orbitals become less stable explaining the increase of the energy barriers and the reduction of the reaction enthalpy. In both cases, highest occupied molecular orbital (HOMO) (located in the cyclopentadiene) and LUMO (located in the fullerenic cage) energy values become more positive as the cycloadditions proceed (see Table 5), obtaining less electrophilic and more stable products. The main difference between the two systems is that both HOMO and LUMO energies for [10]CPP⊃C_60_ structures have higher energies than C_60_ structures. Lower LUMO energies for C_60_ explain the lower barriers for the 1–3 additions. However, for the fourth and sixth additions, the lower barriers for [10]CPP⊃C_60_ can only be explained by the higher dispersion interactions present in [10]CPP⊃C_60_, which are probably somewhat overestimated using the D3(BJ) method. Finally, one can observe that the HOMO–LUMO gap in [10]CPP⊃C_60_ structures is more constant, starting from 2.1 eV in the [10]CPP⊃C_60_ geometry, and after the first adduct, all of the energy values oscillate between 2.2 and 2.3 eV. This trend is slightly different for C_60_. In this case, the HOMO–LUMO gap is reduced from 2.7 eV in C_60_ to 2.3 eV in the sixth insertion.

**Table 5:** HOMO, LUMO, and HOMO–LUMO Energy Gap in eV for All of the Reactant Complexes (RCs) in the Multiple DA Cycloadditions of Cyclopentadiene to [10]CPP⊃C_60_ and C_60_^a^

	[10]CPP⊃C_60_			C_60_		
structure	HOMO	LUMO	H–L gap	HOMO	LUMO	H–L gap
[10]CPP⊃C_60_/C_60_	–5.4	–3.4	2.1	–6.3	–3.6	2.7
RC 1	–5.5	–3.3	2.1	–6.1	–3.5	2.5
RC 2	–5.4	–3.2	2.2	–5.9	–3.4	2.5
RC 3	–5.4	–3.1	2.3	–5.7	–3.3	2.4
RC 4	–5.2	–2.9	2.3	–5.4	–3.1	2.3
RC 5	–5.0	–2.7	2.3	–5.1	–2.8	2.3
RC 6	–4.8	–2.5	2.3	–4.9	–2.7	2.3

a HOMO energy of the cyclopentadiene is −5.8 eV.

## Conclusions

We have studied the multiple Diels–Alder cycloadditions of cyclopentadiene to [10]CPP⊃C_60_. The [10]CPP ring divides the C_60_ of [10]CPP⊃C_60_ into two identical poles and the cycloadditions take place only over the poles of [10]CPP⊃C_60_ because the hindrance generated by the [10]CPP ring disfavors cycloadditions in the equator. The preferred sites of cycloaddition change when going from C_60_ to [10]CPP⊃C_60_. In C_60_, the final hexakisadduct has pseudooctahedral *T*_h_ symmetry, whereas in [10]CPP⊃C_60_, it has *C*_2h_ symmetry. In general, in successive additions, we have observed an increase in the energy barrier and a reduction in the exothermicity of the reaction. Based on Gibbs energies, we have determined that the formation of the [10]CPP⊃C_60_ pentakis- and hexakisadducts is thermodynamically unfavorable and can only be reached in minor quantities by adding an excess of cyclopentadiene. Our results favor the formation of the tris- and tetrakisadducts in [10]CPP⊃C_60_, at the variance of free C_60_, in which the formation of the pentakisadduct is thermodynamically favored. Analyzing the additions that lead to the *C*_2h_ symmetry hexakisadduct but without the [10]CPP ring, we have found that the formation of the tetrakis-, pentakis-, and hexakisadducts is more favored in [10]CPP⊃C_60_ than in free C_60_ because of dispersion interactions with the [10]CPP ring, which are slightly more intense in the TSs than in the RCs. Finally, frontier molecular orbitals show a decrease in the energy of the LUMO orbitals, explaining the increase of the energy barriers and the reduction of the reaction enthalpy after each addition.

## Computational Details

Theoretical calculations were performed by means of the Gaussian16 software package.^55^ Geometry optimizations and frequency calculations were carried out with the B3LYP hybrid functional^56−59^ using the standard 6-31G*^60^ together with the Grimme’s dispersion D3 correction to the electronic energy with the Becke–Johnson (BJ) damping.^61^ Dispersion corrections are essential for the study of chemical reactivity in fullerenes.^62^ For single-point energy refinements, the same B3LYP functional was used with the 6-311G** basis set.^63^ The ultrafine integration grid was employed in all calculations. To simulate solvent effects, calculations were carried out in toluene using the polarizable continuum model (PCM).^64,65^ On top of the B3LYP-D3(BJ)/6-311G**(toluene)//B3LYP-D3(BJ)/6-31G* electronic energies, we added the B3LYP-D3(BJ)/6-31G* thermal and entropy corrections obtained in the gas phase at 298.15 K and under atmospheric pressure conditions. It is likely that the errors in reaction energies for such a method are below 4 kcal/mol.^66,67^ Still, to get the product distributions, the important quantities are ΔΔ*G* for the different products. In this case, we expect an error in ΔΔ*G* much lower, in the order of 1 kcal/mol.

## Supporting Information Available

Tables with reaction energies and energy barriers for the different bisadditions, comparison between [10]CPP⊃C_60_ and C_60_ attacks, and deformation energies. All XYZ coordinates and absolute energies of all computed species (PDF)
