# Supplementary material for: Successive Diels–Alder Cycloadditions of Cyclopentadiene to [10]CPP⊃C60: A Computational Study
Source: J Org Chem. 2022 Mar 23;87(8):5149–57. doi: 10.1021/acs.joc.1c03116 (PMC9016765; doi:10.1021/acs.joc.1c03116)
Supplement: Supplementary file 1 — jo1c03116_si_001.pdf [file jo1c03116_si_001.pdf]

SUPPORTING INFORMATION

Successive Diels-Alder cycloadditions of cyclopentadiene to [10]CPP⊃C_{60}. A Computational Study

Gerard Pareras,[‡,§] Sílvia Simon,[‡] Albert Poater,[‡,*] and Miquel Solà[‡,*]

[‡]Institut de Química Computacional i Catàlisi and Departament de Química, Universitat de Girona, c/ Maria Aurèlia Capmany 69, 17003 Girona, Catalonia, Spain

[§]School of Chemistry, University College Cork, College Road, T12 YN60 Cork, Ireland

Corresponding authors: miquel.sola@udg.edu, albert.poater@udg.edu

Table of Contents

Table S1. Comparison between additions 4 to 4', 4 to 5' and 4 to 6' for the second cyclopentadiene attack, including enthalpies and Gibbs energies (kcal/mol) in a toluene solution. S2

Table S2. Comparison between [10]CPP⊃C_{60} and C_{60} with the same attacks as in [10]CPP⊃C_{60} and considering insertions studied by Sola et al.[1] of the relative energy values for all the reaction steps through the six cycloadditions, including enthalpies and Gibbs energies (kcal/mol) in a toluene solution. S2

Table S3. Activation energies and reaction enthalpies for each addition in kcal/mol considering toluene as a solvent, collected enthalpies and Gibbs energies for [10]CPP⊃C_{60} and C_{60} with the same attacks as in [10]CPP⊃C_{60} and considering insertions studied by Sola et al.[1] S2

Table S4. Deformation and interaction energies in kcal/mol considering toluene as a solvent of the reactant complexes of the different Diels-Alder cycloadditions over the [10]CPP⊃C_{60} and C_{60}. S3

Table S5. Coordinates data set, absolute energies (a.u.) for all DFT optimized [10]CPP⊃C_{60} complexes. S3

Table S6. Coordinates data set, absolute energies (a.u.) for all DFT optimized C_{60} complexes. S26

Table S7. Coordinates data set, absolute energies (a.u.) for all DFT optimized C_{60} complexes considering insertions studied by Sola et al.[1] S38

Table S8. Coordinates data set, absolute energies (a.u.) for all DFT optimized [10]CPP⊃C_{60} geometries considering insertions 4 to 5' and 4 to 6'. S49

References S56

Table S1. Comparison between additions 4 to 4', 4 to 5' and 4 to 6' for the second cyclopentadiene attack, including enthalpies and Gibbs energies (kcal/mol) in a toluene solution.

Structure	Addition 4 → 4-4'		Addition 4 → 4-5'		Addition 4 → 4-6'	
	ΔH	ΔG	ΔH	ΔG	ΔH	ΔG
[10]CPP⊃C_{60}	0.00	0.00	0.00	0.00	0.00	0.00
RC 1	-7.36	0.92	-7.36	0.92	-7.36	0.92
TS 1	5.44	18.40	5.44	18.40	5.44	18.40
Product 1	-17.79	-3.44	-17.79	-3.44	-17.79	-3.44
RC 2	-25.33	-2.52	-21.12	0.48	-21.02	1.13
TS 2	-12.10	15.28	-8.09	17.60	-8.01	18.24
Product 2	-35.12	-6.38	-30.89	-4.08	-32.64	-4.86

Table S2. Comparison between [10]CPP⊃C_{60} and C_{60} with the same attacks as in [10]CPP⊃C_{60} and considering insertions studied by Sola et al.[1] of the relative energy values for all the reaction steps through the six cycloadditions, including enthalpies and Gibbs energies (kcal/mol) in a toluene solution.

Structure	ΔH			ΔG		
	[10]CPP⊃C_{60}	C_{60}	C_{60} JACS	[10]CPP⊃C_{60}	C_{60}	C_{60} JACS
[10]CPP⊃C_{60}/C_{60}	0.00	0.00	0.00	0.00	0.00	0.00
RC 1	-7.36	-5.52	-5.52	0.92	1.82	1.83
TS 1	5.44	5.63	5.63	18.40	17.85	17.85
Product 1	-17.79	-17.11	-17.11	-3.44	-3.53	-3.52
RC 2	-25.33	-22.36	-22.55	-2.52	-2.04	-1.52
TS 2	-12.10	-10.18	-11.09	15.28	15.64	14.71
Product 2	-35.12	-32.63	-33.66	-6.38	-5.47	-6.46
RC 3	-42.63	-38.53	-38.78	-5.92	-2.26	-4.32
TS 3	-26.52	-22.63	-26.78	14.30	16.72	12.63
Product 3	-48.69	-44.67	-49.73	-6.50	-3.94	-8.93
Adduct 4	-56.21	-50.68	-54.76	-6.06	-2.01	-6.89
TS 4	-39.82	-34.24	-41.37	14.56	18.68	11.63
Product 4	-61.96	-56.27	-63.72	-6.30	-1.98	-9.36
RC 5	-68.46	-62.76	-68.67	-5.15	-0.39	-7.32
TS 5	-50.53	-43.87	-54.72	16.84	22.47	11.84
Product 5	-70.49	-63.58	-76.62	-1.63	4.31	-8.69
RC 6	-77.36	-70.02	-81.45	-1.44	5.90	-6.83
TS 6	-58.88	-50.68	-66.81	21.71	29.29	13.31
Product 6	-78.93	-70.33	-88.72	3.10	11.13	-7.21

Table S3. Activation energies and reaction enthalpies for each addition in kcal/mol considering toluene as a solvent, collected enthalpies and Gibbs energies for [10]CPP⊃C_{60} and C_{60} with the same attacks as in [10]CPP⊃C_{60} and considering insertions studied by Sola et al.[1]

Addition[a]	Enthalpy						Gibbs energy					
	Activation Energy			Reaction Energy			Activation Energy			Reaction Energy		
	[10]CPP⊃C_{60}	C_{60}	C_{60} (JACS)	[10]CPP⊃C_{60}	C_{60}	C_{60} (JACS)	[10]CPP⊃C_{60}	C_{60}	C_{60} (JACS)	[10]CPP⊃C_{60}	C_{60}	C_{60} (JACS)
1	12.80	11.15	11.15	-10.43	-11.60	-11.60	17.48	16.04	16.03	-4.36	-5.34	-5.35
2	13.23	12.19	11.46	-9.79	-10.27	-11.11	17.79	17.67	16.23	-3.86	-3.43	-4.94
3	16.10	15.90	12.00	-6.06	-6.15	-10.95	20.23	18.98	16.95	-0.58	-1.67	-4.62

4	16.38	16.44	13.38	-5.75	-5.59	-8.97	20.62	20.69	18.51	-0.24	0.03	-2.47
5	17.93	18.89	13.94	-2.04	-0.82	-7.96	21.98	22.86	19.16	3.52	4.70	-1.37
6	18.48	19.35	14.65	-1.57	-0.31	-7.27	23.15	23.39	20.15	4.54	5.23	-0.37

[a] Order of addition is 4→4-4'→4-4'-5→4-4'-5-5'→4-4'-5-5'-6→4-4'-5-5'-6-6' in [10]CPP⊃C_{60} and C_{60} and 1'→1-1'-2→1-1'-2-2'→1-1'-2-2'-3→1-1'-2-2'-3-3' in C_{60} (JACS) (see Scheme 1).

Table S4. Deformation and interaction energies in kcal/mol considering toluene as a solvent of the reactant complexes of the different Diels-Alder cycloadditions over the [10]CPP⊃C_{60} and C_{60}.

Addition[a]	[10]CPP⊃C_{60} Reactant Complexes					C_{60} Reactant Complexes				
	E_{def1}	E_{def2}	E_{def}	E_{int}	$E_{def+Eint}$	E_{def1}	E_{def2}	E_{def}	E_{int}	$E_{def+Eint}$
1	0.4	0.0	0.4	-9.1	-8.7	0.0	0.1	0.1	-6.8	-6.7
2	0.2	0.1	0.3	-9.1	-8.8	0.0	0.1	0.1	-6.6	-6.5
3	0.1	0.0	0.1	-8.8	-8.7	0.0	0.0	0.0	-6.6	-6.6
4	0.0	0.1	0.1	-8.8	-8.7	0.0	0.0	0.0	-7.3	-7.3
5	1.5	0.1	1.6	-9.4	-7.8	0.0	0.0	0.0	-7.8	-7.8
6	0.8	0.0	0.8	-9.0	-8.2	0.0	0.0	0.0	-7.7	-7.7

[a] Order of addition is 4→4-4’→4-4’-5→4-4’-5-5’→4-4’-5-5’-6→4-4’-5-5’-6-6’ (Scheme 1).

Table S5. Coordinates data set, absolute energies (a.u.) for all DFT optimized [10]CPP⊃C_{60} complexes.

C60CPP10	C60CPP10_1_Int
160	171
C60CPP10 SCF Done: -4598.27591548 A.U.	C60CPP10_1_Int_2 SCF Done: -4792.45929658 A.U.
C 5.448469 4.745340 -1.073917	C -6.351352 -3.598890 1.369339
C 6.271697 3.624487 -1.074559	C -5.542921 -4.727601 1.322204
C 6.345477 2.778126 0.045180	C -4.878797 -5.097330 0.138616
C 5.692169 3.200088 1.215323	C -5.212237 -4.390000 -1.028860
C 4.872602 4.319693 1.216657	C -6.022500 -3.262024 -0.982216
C 4.665324 5.070225 0.047425	C -6.534368 -2.786412 0.235484
H 5.370289 5.346066 -1.975459	H -6.800255 -3.309134 2.314794
H 6.820406 3.370530 -1.976899	H -5.377453 -5.297243 2.231887
H 5.710311 2.566991 2.095703	H -4.716010 -4.633352 -1.962298
H 4.276450 4.527835 2.098404	H -6.131015 -2.661369 -1.879521
C 6.816020 1.378370 -0.042326	C -3.680072 -5.964517 0.144481
C 6.597098 0.654030 -1.225121	C -2.836272 -5.965026 1.268263
C 7.214255 0.650243 1.093191	C -3.191129 -6.578888 -1.022484
C 6.588473 -0.735131 -1.225188	C -1.516435 -6.389998 1.179487
H 6.280708 1.177062 -2.121507	H -3.167182 -5.491120 2.186114
C 7.205626 -0.739255 1.093124	C -1.870947 -7.002132 -1.111898
H 7.471755 1.178568 2.006457	H -3.836115 -6.673259 -1.891104
C 6.798365 -1.462259 -0.042463	C -0.976888 -6.831619 -0.038969
H 6.265628 -1.254105 -2.121630	H -0.860938 -6.232842 2.029763
H 7.456530 -1.270833 2.006341	H -1.511790 -7.418179 -2.048477
C 3.477587 5.947858 -0.037184	C -6.970430 -1.376595 0.341731
C 2.911808 6.553226 1.099555	C -7.484029 -0.655392 -0.750310
C 2.722264 5.973691 -1.220269	C -6.616599 -0.643600 1.486699
C 1.594730 6.995042 1.098025	C -7.486874 0.735548 -0.755491
H 3.493244 6.625659 2.013933	H -7.834682 -1.188984 -1.629029
C 1.404133 6.412727 -1.220808	C -6.616999 0.743725 1.480647
H 3.116137 5.506396 -2.116958	H -6.199214 -1.159887 2.344164
H 1.173982 7.405121 2.011347	H -7.839407 1.260915 -1.638400
H 0.812162 6.274500 -2.119198	H -6.198953 1.267624 2.333176
C 6.310393 -2.856051 0.044916	C 0.490160 -6.827796 -0.231402
C 5.652026 -3.269956 1.215103	C 1.013103 -6.381390 -1.456498
C 6.225873 -3.701287 -1.074917	C 1.395652 -6.996823 0.830778
C 4.818494 -4.379204 1.216422	C 2.333727 -5.971420 -1.567558
H 5.678240 -2.637247 2.095557	H 0.345672 -6.218821 -2.295637
C 5.388683 -4.811752 -1.074285	C 2.720017 -6.586512 0.719091
H 6.777580 -3.454096 -1.977308	H 1.046039 -7.405335 1.774477
C 4.601701 -5.126951 0.047136	C 3.197621 -5.987993 -0.459455
H 4.219918 -4.579992 2.098232	H 2.656453 -5.501331 -2.489989

H	5.302848	-5.411350	-1.975883
C	3.403166	-5.989789	-0.037370
C	2.647393	-6.006098	-1.220332
C	2.830204	-6.588405	1.099350
C	1.323997	-6.428990	-1.220743
H	3.046836	-5.543499	-2.116994
C	1.507819	-7.014095	1.097943
H	3.410881	-6.668166	2.013600
C	0.695534	-6.859346	-0.039934
H	0.733611	-6.283377	-2.119012
H	1.082250	-7.419253	2.011228
C	-0.781574	-6.850576	0.039842
C	-1.404955	-6.412953	1.220674
C	-1.595531	-6.995281	-1.098155
C	-2.723143	-5.974090	1.220178
H	-0.812959	-6.274579	2.119024
C	-2.912659	-6.553603	-1.099654
H	-1.174733	-7.405259	-2.011501
C	-3.478470	-5.948294	0.037099
H	-3.117037	-5.506799	2.116861
H	-3.494092	-6.626031	-2.014034
C	-4.666144	-5.070583	-0.047525
C	-4.873241	-4.319978	-1.216738
C	-5.449275	-4.745611	1.073798
C	-5.692648	-3.200260	-1.215411
H	-4.277029	-4.528152	-2.098437
C	-6.272338	-3.624632	1.074437
H	-5.371168	-5.346341	1.975344
C	-6.345964	-2.778241	-0.045288
H	-5.710616	-2.567110	-2.095754
H	-6.821011	-3.370585	1.976775
C	-6.816341	-1.378420	0.042194
C	-6.597220	-0.654015	1.224913
C	-7.214705	-0.650349	-1.093317
C	-6.588506	0.735152	1.224886
H	-6.280767	-1.177019	2.121295
C	-7.206008	0.739146	-1.093341
H	-7.472446	-1.178729	-2.006483
C	-6.798544	1.462204	0.042142
H	-6.265554	1.254190	2.121256
H	-7.457118	1.270685	-2.006522
C	-6.310722	2.856043	-0.045345
C	-5.652213	3.269884	-1.215480
C	-6.226520	3.701438	1.074389
C	-4.818897	4.379293	-1.216828
H	-5.678160	2.637040	-2.095843
C	-5.389535	4.812057	1.073733
H	-6.778310	3.454250	1.976731
C	-4.602453	5.127252	-0.047615
H	-4.220207	4.580064	-2.098570
C	-3.404027	5.990214	0.036977
C	-2.648382	6.006576	1.220016
C	-2.830912	6.588687	-1.099735
C	-1.324940	6.429306	1.220497
H	-3.047922	5.544019	2.116656
C	-1.508491	7.014260	-1.098241
H	-3.411472	6.668347	-2.014069
C	-0.696322	6.859508	0.039714
H	-0.734630	6.283574	2.118794
H	3.378112	-6.681839	1.577981
C	4.418371	-5.152476	-0.495904
C	4.780800	-4.409391	0.639319
C	5.077448	-4.849332	-1.701033
C	5.614474	-3.302214	0.536717
H	4.287963	-4.604997	1.585916
C	5.909209	-3.741227	-1.804545
H	4.885533	-5.452093	-2.583746
C	6.121525	-2.889923	-0.706006
H	5.746785	-2.664011	1.404653
H	6.349808	-3.494746	-2.766284
C	6.567271	-1.489221	-0.870894
C	6.131639	-0.776825	-2.000446
C	7.166350	-0.753877	0.165745
C	6.130584	0.610984	-2.016382
H	5.652369	-1.309474	-2.814616
C	7.164215	0.637336	0.150055
H	7.584099	-1.277812	1.021097
C	6.563520	1.348439	-0.902578
H	5.651604	1.124963	-2.842770
H	7.579455	1.181979	0.993277
C	6.115569	2.751009	-0.763235
C	5.612448	3.182066	0.474052
C	5.906427	3.588086	-1.873216
C	4.794280	4.301424	0.564794
H	5.726962	2.546232	1.345140
C	5.090598	4.709111	-1.781106
H	6.340603	3.323987	-2.833253
C	4.443202	5.041343	-0.576989
H	4.304577	4.510863	1.510009
H	4.906253	5.302986	-2.671399
C	3.244140	5.907419	-0.541703
C	2.387336	5.941944	-1.654932
C	2.771730	6.491100	0.647518
C	1.073429	6.377958	-1.541103
H	2.703815	5.488921	-2.588270
C	1.456678	6.925100	0.762234
H	3.426530	6.555425	1.511561
C	0.551483	6.796967	-0.306919
H	0.407556	6.250140	-2.388145
H	1.110847	7.318418	1.713591
C	-0.914426	6.814418	-0.107359
C	-1.443999	6.342478	1.105137
C	-1.817451	7.028465	-1.163568
C	-2.769363	5.942813	1.202704
H	-0.779384	6.149342	1.940095
C	-3.145748	6.629057	-1.065489
H	-1.462352	7.461764	-2.094044
C	-3.629372	5.996537	0.092808
H	-3.098639	5.449295	2.110544
C	-4.848679	5.158475	0.093166
C	-5.193435	4.449394	-1.068913
C	-5.520513	4.812845	1.279449
C	-6.018105	3.332838	-1.011416
H	-4.692845	4.679352	-2.003580
C	-6.346080	3.696580	1.336708
H	-5.343931	5.388148	2.183545
C	-6.535668	2.874848	0.210864
H	-6.135669	2.725772	-1.902966

H	-1.082784	7.419240	-2.011540	H	-6.799134	3.421215	2.284522
C	0.780782	6.850440	-0.039994	C	-6.973606	1.466633	0.329945
C	1.891396	2.296043	-1.945849	C	-1.343389	-0.864554	3.080818
C	0.651584	3.028292	-1.735608	C	-1.903189	0.468594	2.913807
C	1.901619	1.166716	-2.766049	C	0.036691	-1.031383	3.206480
C	-0.525100	2.607528	-2.356473	C	-1.059845	1.580733	2.880083
C	-1.781461	2.617184	-1.619733	C	-1.297302	2.646613	1.917732
C	0.623193	3.473187	-0.350538	C	-3.020632	0.373765	1.986254
C	-0.577504	3.480742	0.353237	C	-3.247294	1.394996	1.061956
C	-1.805047	3.043685	-0.292545	C	-2.367556	2.553738	1.026671
C	3.352078	-0.021272	-1.162223	C	-0.033639	-3.006511	1.725945
C	3.344197	1.156596	-0.307460	C	-1.471916	-2.828154	1.592331
C	2.651116	-0.016318	-2.367084	C	0.705216	-2.124026	2.515533
C	2.629490	2.290493	-0.689629	C	-2.114680	-1.783823	2.257609
C	1.844935	3.019816	0.294915	C	-3.152169	-1.018300	1.580451
C	3.313831	0.705960	1.076047	C	-1.837098	-3.151138	0.221471
C	2.564307	1.409187	2.020379	C	-2.828197	-2.418719	-0.424967
C	1.815847	2.592775	1.621827	C	-3.499746	-1.329758	0.266811
C	1.861535	-2.318949	-1.947445	C	2.494817	-2.053653	0.818845
C	2.599626	-2.323822	-0.691223	C	1.725538	-2.972999	-0.005189
C	1.886369	-1.189272	-2.766854	C	1.996749	-1.638998	2.051785
C	3.328927	-1.199520	-0.308275	C	0.488555	-3.439750	0.437505
C	3.304396	-0.749479	1.075538	C	-0.627614	-3.530382	-0.491068
C	1.805721	-3.043637	0.292816	C	1.903631	-2.584016	-1.396346
C	1.782152	-2.617156	1.620015	C	0.833224	-2.676667	-2.287220
C	2.545842	-1.443609	2.019383	C	-0.459458	-3.162587	-1.825461
C	-0.531606	-1.420784	-3.211473	C	2.756407	0.671165	1.617000
C	-0.558782	-2.598836	-2.358280	C	3.278604	0.235747	0.326664
C	0.665275	-0.730772	-3.411310	C	2.126556	-0.246827	2.456560
C	0.612357	-3.035251	-1.737716	C	3.151765	-1.096666	-0.062667
C	0.578202	-3.480711	-0.352963	C	2.783905	-1.425317	-1.431304
C	-1.815155	-2.592745	-1.621556	C	3.035487	1.298106	-0.636887
C	-1.844247	-3.019798	-0.294650	C	2.687593	0.987040	-1.950816
C	-0.622495	-3.473164	0.350811	C	2.556429	-0.404174	-2.356324
C	-0.513163	1.429812	-3.210476	C	0.379018	1.407503	3.013015
C	-1.761146	0.710231	-3.002159	C	1.032030	2.366754	2.133258
C	0.674692	0.724505	-3.410806	C	0.914769	0.128064	3.168057
C	-1.770178	-0.685260	-3.002652	C	2.191730	2.001608	1.447054
C	-2.563613	-1.409154	-2.020106	C	2.363251	2.387516	0.054851
C	-2.545155	1.443643	-2.019106	C	-0.004264	3.132365	1.455788
C	-3.303722	0.749511	-1.075268	C	0.163615	3.500054	0.120730
C	-3.313148	-0.705930	-1.075779	C	1.372198	3.120520	-0.591685
C	-1.890705	-2.296012	1.946112	C	0.877514	0.833793	-3.450682
C	-2.628800	-2.290470	0.689891	C	1.648950	1.752532	-2.627861
C	-3.343510	-1.156570	0.307726	C	1.006499	2.796990	-1.963045
C	-1.900931	-1.166684	2.766312	C	-0.502635	1.000434	-3.578174
C	0.513854	-1.429775	3.210746	C	-0.844093	-1.437248	-3.382194
C	0.525791	-2.607488	2.356741	C	0.594951	-1.610770	-3.249324
C	-0.650891	-3.028257	1.735874	C	1.437988	-0.499167	-3.282936
C	-0.674004	-0.724471	3.411071	C	-1.381514	-0.158978	-3.542810
C	0.532297	1.420816	3.211741	C	-3.219193	-0.701553	-1.985662
C	1.770869	0.685294	3.002925	C	-2.656491	-2.032887	-1.817186
C	1.761837	-0.710195	3.002435	C	-1.495616	-2.395155	-2.502205
C	-0.664589	0.730806	3.411574	C	-2.593448	0.216188	-2.830655
C	-1.860856	2.318979	1.947706	C	-2.958049	2.021428	-1.188538
C	-0.611670	3.035273	1.737979	C	-3.612852	1.065590	-0.307402
C	0.559473	2.598862	2.358544	C	-3.741104	-0.266657	-0.696293
C	-1.885689	1.189305	2.767121	C	-2.463280	1.608700	-2.424679
C	-3.351412	0.021300	1.162492	C	-0.431320	2.974255	-2.096820

C -3.328281 1.199558 0.308543	C -0.952900 3.407865 -0.807535
C -2.598962 2.323863 0.691489	C -2.189507 2.942112 -0.364657
C -2.650439 0.016349 2.367353	C -1.170528 2.092947 -2.887595
H -5.303939 5.411790 1.975262	H -3.801877 6.756694 -1.921637
	C 3.931092 -0.397651 4.967910
	C 5.092736 1.360609 3.909341
	C 3.984807 1.104816 4.895341
	H 3.028963 1.536777 4.567365
	H 4.200387 1.553436 5.877102
	C 5.603482 0.178635 3.500927
	H 5.421595 2.349962 3.614702
	C 4.881756 -0.913677 4.159156
	H 6.405360 0.038515 2.787460
	H 3.227699 -0.948231 5.580712
	H 5.083345 -1.967398 3.997219
**C60CPP10_1_TS**	**C60CPP10_1_Prod**
171	171
C60CPP10_1_TS_2 SCF Done: -4792.43795844 A.U.	C60CPP10_1_Prod_2 SCF Done: -4792.47844483 A.U.
C 5.915318 -3.586558 -1.871975	C -6.464603 -3.507947 1.153358
C 5.082553 -4.694717 -1.785105	C -5.668684 -4.645982 1.116986
C 4.480833 -5.063424 -0.567381	C -4.969341 -5.007483 -0.048811
C 4.917986 -4.389547 0.585771	C -5.255205 -4.280197 -1.216558
C 5.762508 -3.289476 0.499272	C -6.051308 -3.141688 -1.180189
C 6.196527 -2.798566 -0.741928	C -6.597644 -2.676124 0.026561
H 6.304425 -3.292693 -2.842300	H -6.941359 -3.225985 2.087472
H 4.848023 -5.246965 -2.689772	H -5.540622 -5.230352 2.023398
H 4.486752 -4.639270 1.549599	H -4.731484 -4.517414 -2.136516
H 5.980364 -2.721638 1.396850	H -6.121498 -2.526643 -2.071611
C 3.266373 -5.908085 -0.519798	C -3.781443 -5.889201 -0.014943
C 2.400553 -5.931831 -1.626519	C -2.972982 -5.911117 1.134440
C 2.794894 -6.491123 0.670533	C -3.263753 -6.498553 -1.171969
C 1.086278 -6.362171 -1.507368	C -1.655716 -6.350025 1.082168
H 2.710240 -5.476576 -2.560564	H -3.326817 -5.442042 2.046169
C 1.476974 -6.916601 0.792055	C -1.946206 -6.936312 -1.224625
H 3.452294 -6.565186 1.531729	H -3.882494 -6.576972 -2.061064
C 0.567798 -6.785239 -0.272977	C -1.083311 -6.785570 -0.123455
H 0.417426 -6.228848 -2.350858	H -1.025405 -6.207863 1.953719
H 1.133630 -7.308429 1.745030	H -1.563395 -7.347873 -2.153780
C 6.639557 -1.391039 -0.854803	C -7.011586 -1.260022 0.140734
C 7.243184 -0.686985 0.202675	C -7.470762 -0.509532 -0.955996
C 6.195868 -0.639788 -1.955289	C -6.681631 -0.553017 1.308715
C 7.239416 0.705404 0.232491	C -7.438068 0.880935 -0.939161
H 7.670506 -1.234260 1.038544	H -7.803712 -1.020463 -1.854876
C 6.192970 0.747094 -1.925731	C -6.646889 0.833900 1.324873
H 5.710370 -1.142450 -2.784237	H -6.306998 -1.092068 2.171867
H 7.666104 1.222369 1.088138	H -7.746789 1.427944 -1.825307
H 5.705946 1.285425 -2.731247	H -6.245614 1.333172 2.199812
C -0.898880 -6.807664 -0.078505	C 0.389120 -6.794422 -0.272733
C -1.440855 -6.331403 1.126792	C 0.950446 -6.347115 -1.480496
C -1.791789 -7.029175 -1.141812	C 1.263325 -6.976503 0.813244
C -2.766802 -5.927261 1.207200	C 2.278218 -5.952679 -1.554244
H -0.784562 -6.135009 1.967717	H 0.308028 -6.172190 -2.336404
C -3.119729 -6.626511 -1.060574	C 2.594793 -6.579786 0.739645
H -1.427720 -7.469244 -2.065600	H 0.883554 -7.383754 1.745772
C -3.613463 -5.981951 0.086934	C 3.112108 -5.985188 -0.423938
H -3.104677 -5.427463 2.108480	H 2.631602 -5.482740 -2.465477
H -3.766996 -6.759126 -1.922615	H 3.228043 -6.684502 1.616042
C -4.828085 -5.136987 0.059367	C 4.348711 -5.172316 -0.429812
C -5.151625 -4.444020 -1.118715	C 4.694493 -4.428602 0.709984

C	-5.519904	-4.772526	1.228244
C	-5.979397	-3.328862	-1.093579
H	-4.633360	-4.686020	-2.040473
C	-6.350069	-3.657995	1.252742
H	-5.357917	-5.332386	2.144747
C	-6.523362	-2.854639	0.110944
H	-6.081154	-2.735887	-1.996419
H	-6.820556	-3.369884	2.188164
C	-6.973750	-1.447664	0.197910
C	-6.656674	-0.705898	1.347776
C	-7.459176	-0.734371	-0.912491
C	-6.662396	0.681434	1.332869
H	-6.261509	-1.214083	2.220363
C	-7.462410	0.656548	-0.928222
H	-7.783335	-1.273852	-1.797746
C	-6.982994	1.396410	0.167059
H	-6.272345	1.211592	2.194679
H	-7.788382	1.175001	-1.825359
C	-6.541235	2.804083	0.052578
C	-5.992078	3.261000	-1.156189
C	-6.385978	3.631337	1.179942
C	-5.173611	4.383539	-1.194679
H	-6.078528	2.648931	-2.048225
C	-5.567992	4.753519	1.141348
H	-6.863351	3.357306	2.116101
C	-4.867412	5.103187	-0.027315
H	-4.649671	4.611478	-2.116847
H	-5.423986	5.333559	2.048142
C	-3.661994	5.961079	0.002441
C	-2.849359	5.967120	1.149113
C	-3.135981	6.559996	-1.156309
C	-1.524016	6.380471	1.092707
H	-3.208981	5.504504	2.061849
C	-1.810280	6.971901	-1.213201
H	-3.755692	6.650262	-2.043595
C	-0.947010	6.804874	-0.114669
H	-0.893848	6.225836	1.962244
H	-1.422463	7.375527	-2.143757
C	0.524949	6.786796	-0.268283
C	1.074967	6.330849	-1.477990
C	1.405605	6.953507	0.815031
C	2.395834	5.914766	-1.556096
H	0.427064	6.166497	-2.331835
C	2.730286	6.535354	0.736923
H	1.035616	7.366488	1.748964
C	3.234219	5.933309	-0.428668
H	2.737987	5.438990	-2.468526
C	4.457327	5.100076	-0.439874
C	4.798492	4.352369	0.698755
C	5.146746	4.808675	-1.630943
C	5.642803	3.251670	0.608534
H	4.281654	4.538685	1.634346
C	5.987401	3.706278	-1.722322
H	4.971554	5.416047	-2.513937
C	6.180731	2.849859	-0.624356
H	5.761373	2.611149	1.475923
H	6.450017	3.467346	-2.675631
C	6.631875	1.450769	-0.792958
C	1.159882	-0.924760	-3.262812
C	5.050838	-4.895031	-1.616703
C	5.560873	-3.344893	0.625081
H	4.167364	-4.603173	1.642068
C	5.914657	-3.809983	-1.702969
H	4.869744	-5.499899	-2.500276
C	6.118177	-2.957464	-0.603998
H	5.681954	-2.705045	1.491968
H	6.389412	-3.581873	-2.652989
C	6.605330	-1.569893	-0.767524
C	6.194361	-0.853466	-1.902680
C	7.221812	-0.841777	0.264977
C	6.230005	0.533122	-1.928214
H	5.702914	-1.378377	-2.714443
C	7.258386	0.550021	0.239356
H	7.627525	-1.370910	1.123234
C	6.680320	1.270803	-0.821174
H	5.764249	1.049216	-2.760268
H	7.690323	1.084848	1.080829
C	6.266038	2.686986	-0.706066
C	5.833788	3.182305	0.534215
C	6.007882	3.484293	-1.835204
C	5.009633	4.298001	0.618533
H	6.033102	2.608473	1.432522
C	5.195747	4.607824	-1.750226
H	6.397901	3.186333	-2.803912
C	4.592891	4.983594	-0.535211
H	4.576931	4.552301	1.580450
H	4.977788	5.167469	-2.654524
C	3.393729	5.850018	-0.492251
C	2.534393	5.891642	-1.603498
C	2.926692	6.438900	0.696935
C	1.227281	6.344632	-1.490059
H	2.841319	5.433527	-2.537086
C	1.615910	6.887373	0.812605
H	3.580886	6.499372	1.561591
C	0.710105	6.774135	-0.257319
H	0.560587	6.225315	-2.337357
H	1.274742	7.283447	1.764601
C	-0.756863	6.822157	-0.070039
C	-1.313189	6.354270	1.132051
C	-1.640439	7.061155	-1.137289
C	-2.646761	5.974772	1.205781
H	-0.664599	6.145375	1.975966
C	-2.975914	6.682763	-1.062881
H	-1.264001	7.495637	-2.058746
C	-3.487022	6.046655	0.081667
H	-2.998463	5.480855	2.105038
C	-4.717809	5.225708	0.047863
C	-5.049453	4.539726	-1.132032
C	-5.422808	4.875477	1.213204
C	-5.900660	3.442237	-1.111411
H	-4.521779	4.771365	-2.051111
C	-6.276495	3.778789	1.233152
H	-5.253921	5.431954	2.130520
C	-6.461120	2.979749	0.090137
H	-6.010766	2.851823	-2.014955
H	-6.758018	3.500626	2.165961
C	-6.942475	1.582866	0.174031
C	-1.648617	-0.899908	3.009481

C	1.690433	0.420603	-3.088575	C	-2.141299	0.447998	2.836702
C	-0.204547	-1.113311	-3.491135	C	-0.283798	-1.106022	3.240223
C	0.832699	1.520573	-3.150973	C	-1.243615	1.519570	2.906142
C	0.980900	2.613896	-2.201201	C	-1.352500	2.617485	1.959018
C	2.732507	0.365904	-2.076275	C	-3.169253	0.424746	1.809227
C	2.870973	1.412268	-1.156735	C	-3.269404	1.479111	0.898811
C	1.975143	2.556606	-1.223231	C	-2.345137	2.601082	0.979350
C	-0.224824	-3.043312	-1.950880	C	-0.289603	-3.049433	1.714632
C	1.195532	-2.841213	-1.711416	C	-1.704199	-2.817866	1.457985
C	-0.911340	-2.195409	-2.821942	C	0.407738	-2.201440	2.581847
C	1.875208	-1.808866	-2.355255	C	-2.370628	-1.771743	2.092948
C	2.845921	-1.004963	-1.622179	C	-3.312457	-0.949212	1.348042
C	1.453247	-3.119619	-0.304863	C	-1.948130	-3.089894	0.050372
C	2.373017	-2.342075	0.398508	C	-2.848046	-2.302417	-0.663186
C	3.081972	-1.264580	-0.270308	C	-3.544827	-1.208973	-0.002045
C	-2.824162	-2.100764	-1.266948	C	2.336276	-2.149392	1.100331
C	-2.110666	-2.983731	-0.360122	C	1.621210	-3.005748	0.175043
C	-2.240713	-1.716340	-2.473832	C	1.749722	-1.771707	2.287225
C	-0.838572	-3.446262	-0.692466	C	0.334948	-3.461637	0.477022
C	0.203950	-3.497449	0.320025	C	-0.687738	-3.489543	-0.555424
C	-2.398913	-2.554766	1.001290	C	1.940293	-2.582054	-1.178451
C	-1.398374	-2.601863	1.972953	C	0.964655	-2.626403	-2.175026
C	-0.068081	-3.084192	1.628042	C	-0.380777	-3.084991	-1.855219
C	-3.057195	0.594439	-2.163406	C	2.889913	0.605298	2.132234
C	-3.674764	0.189519	-0.906386	C	3.266302	0.125024	0.729429
C	-2.355861	-0.337848	-2.929730	C	2.045866	-0.455749	2.999587
C	-3.558642	-1.128887	-0.467360	C	3.116400	-1.175349	0.299464
C	-3.297055	-1.411644	0.933775	C	2.857197	-1.457625	-1.098472
C	-3.523933	1.283523	0.039247	C	3.117533	1.199974	-0.216147
C	-3.277169	1.016632	1.385045	C	2.895895	0.950925	-1.573831
C	-3.158665	-0.360814	1.842909	C	2.759371	-0.414618	-2.026817
C	-0.587594	1.323724	-3.390761	C	0.161376	1.284472	3.172373
C	-1.318523	2.297517	-2.592359	C	0.937599	2.261204	2.371542
C	-1.096728	0.033958	-3.556359	C	0.629475	0.001134	3.350432
C	-2.527715	1.938909	-1.992066	C	2.134118	1.891491	1.796298
C	-2.814055	2.362446	-0.630851	C	2.420182	2.290410	0.444108
C	-0.350050	3.097877	-1.860188	C	-0.009167	3.071839	1.632798
C	-0.628532	3.504906	-0.555146	C	0.284794	3.491940	0.330971
C	-1.887255	3.129310	0.069585	C	1.530607	3.091569	-0.276265
C	-1.591979	0.930351	3.024353	C	1.233399	0.905999	-3.238575
C	-2.307442	1.817924	2.117314	C	1.953837	1.775117	-2.320667
C	-1.628382	2.851129	1.473383	C	1.287391	2.821309	-1.686439
C	-0.223369	1.113357	3.238655	C	-0.124082	1.122245	-3.485043
C	0.164274	-1.306797	3.163443	C	-0.557706	-1.306372	-3.388535
C	-1.252244	-1.506587	2.920410	C	0.853329	-1.531753	-3.128575
C	-2.118993	-0.410083	2.858071	C	1.733492	-0.449791	-3.055802
C	0.669973	-0.023190	3.316759	C	-1.038143	-0.006330	-3.561459
C	2.740146	-0.612746	2.006336	C	-3.029303	-0.527008	-2.195760
C	2.098780	-1.925267	1.756772	C	-2.531635	-1.883015	-2.018433
C	0.901821	-2.289891	2.358491	C	-1.320651	-2.265839	-2.600709
C	2.004269	0.365952	2.804586	C	-2.297674	0.390869	-2.951196
C	2.397036	2.111525	1.046731	C	-2.751176	2.162127	-1.293563
C	3.139792	1.127761	0.239661	C	-3.517639	1.204654	-0.504738
C	3.246277	-0.183013	0.675402	C	-3.656947	-0.110903	-0.947697
C	1.807787	1.722135	2.237267	C	-2.160160	1.766525	-2.493789
C	-0.205776	3.054600	1.710152	C	-0.125190	3.053070	-1.944887
C	0.409630	3.458636	0.461389	C	-0.746418	3.466589	-0.693614
C	1.684545	2.986715	0.136487	C	-2.031397	3.027930	-0.377639
C	0.481655	2.193515	2.570360	C	-0.817619	2.218841	-2.824892

H	3.367818	6.629517	1.611441
H	2.629437	1.037399	5.275484
C	3.275072	0.550736	4.553264
H	4.615124	2.215434	3.957807
C	4.404567	1.151913	3.960079
C	3.521894	-0.940297	4.535447
H	2.629509	-1.568235	4.520119
H	4.111506	-1.211317	5.426188
C	5.092953	0.191028	3.215108
C	4.413787	-1.039635	3.320156
H	5.913033	0.385725	2.536384
H	4.784980	-1.981681	2.938018

H	-3.616599	6.828326	-1.927747
C	2.960663	-0.617799	4.289629
C	4.131305	0.875081	3.087538
C	3.449210	0.831498	4.467328
H	2.639163	1.557967	4.581839
H	4.162394	0.936112	5.289356
C	4.938804	-0.407152	3.123729
H	4.663752	1.784987	2.814724
C	4.239450	-1.299333	3.838220
H	5.843568	-0.571893	2.553557
H	2.422478	-1.081301	5.117562
H	4.460968	-2.351278	3.979584

C60CPP10_2_Int

182

C60CPP10_2_Int_2 SCF Done: -4986.66211345 A.U.

C	6.146265	-3.511938	-1.923476
C	5.350277	-4.642872	-1.789676
C	4.753138	-4.971145	-0.558798
C	5.139599	-4.216381	0.561645
C	5.936709	-3.085546	0.428605
C	6.383586	-2.658057	-0.831668
H	6.539552	-3.252358	-2.902146
H	5.140143	-5.248093	-2.666571
H	4.690722	-4.424655	1.527235
H	6.078943	-2.441821	1.290347
C	3.566110	-5.850633	-0.470160
C	2.662173	-5.896130	-1.545292
C	3.151161	-6.437924	0.738370
C	1.357374	-6.341274	-1.373658
H	2.934003	-5.441700	-2.491884
C	1.845816	-6.882651	0.910268
H	3.843261	-6.495404	1.573420
C	0.892101	-6.760358	-0.117081
H	0.654788	-6.217930	-2.190992
H	1.546321	-7.278442	1.876134
C	6.793376	-1.247528	-1.009072
C	7.410115	-0.498601	0.007995
C	6.303616	-0.542927	-2.120532
C	7.371487	0.892567	-0.002392
H	7.870293	-1.013146	0.847003
C	6.264026	0.844344	-2.130514
H	5.811319	-1.085053	-2.920542
H	7.803815	1.443780	0.828206
H	5.740834	1.345294	-2.937559
C	-0.563326	-6.782587	0.152632
C	-1.031347	-6.329186	1.397229
C	-1.520120	-6.983330	-0.858057
C	-2.352328	-5.943257	1.572242
H	-0.325067	-6.140844	2.198146
C	-2.844471	-6.594679	-0.683282
H	-1.212824	-7.397814	-1.813771
C	-3.272267	-5.990379	0.511275
H	-2.635821	-5.466853	2.504300
H	-3.544052	-6.712164	-1.505981
C	-4.508135	-5.181483	0.605088
C	-4.942736	-4.449058	-0.511497
C	-5.118411	-4.894476	1.839489
C	-5.802630	-3.365958	-0.370401
H	-4.488316	-4.631184	-1.479690

C60CPP10_2_TS

182

C60CPP10_2_TS_2 SCF Done: -4986.64011946 A.U.

C	-6.122575	3.521132	-1.912920
C	-5.314986	4.644425	-1.788612
C	-4.751789	5.005600	-0.550609
C	-5.200332	4.302525	0.580500
C	-6.016098	3.184427	0.455982
C	-6.412192	2.707123	-0.803244
H	-6.482891	3.235976	-2.896864
H	-5.068372	5.215805	-2.678264
H	-4.794445	4.543838	1.557388
H	-6.235469	2.593504	1.338500
C	-3.554584	5.870844	-0.456418
C	-2.644190	5.904431	-1.526523
C	-3.140268	6.460630	0.751465
C	-1.338496	6.343271	-1.351370
H	-2.911494	5.446299	-2.472298
C	-1.832177	6.896402	0.928200
H	-3.834769	6.528862	1.583642
C	-0.875702	6.766314	-0.095194
H	-0.633303	6.212135	-2.164996
H	-1.533582	7.292371	1.894378
C	-6.822361	1.292786	-0.951663
C	-7.427968	0.554419	0.081294
C	-6.343930	0.572527	-2.058127
C	-7.393954	-0.837573	0.083875
H	-7.881787	1.075131	0.920138
C	-6.309212	-0.814332	-2.054759
H	-5.855254	1.101226	-2.868778
H	-7.825508	-1.379464	0.921354
H	-5.794489	-1.323946	-2.861603
C	0.580047	6.786003	0.171963
C	1.054061	6.322544	1.410551
C	1.530897	6.991957	-0.843121
C	2.374306	5.926540	1.572594
H	0.351717	6.131850	2.214524
C	2.853779	6.594607	-0.681024
H	1.218846	7.416296	-1.792957
C	3.285606	5.974805	0.504143
H	2.661775	5.440177	2.498334
H	3.548481	6.715837	-1.507241
C	4.515261	5.154153	0.573647
C	4.929439	4.435268	-0.559528
C	5.140629	4.844933	1.794816
C	5.787148	3.347547	-0.447605
H	4.460763	4.632375	-1.517873

C	-5.976989	-3.811071	1.981357
H	-4.868440	-5.490399	2.712305
C	-6.265856	-2.968094	0.893964
H	-5.990658	-2.734632	-1.231664
H	-6.379290	-3.576551	2.962705
C	-6.744508	-1.580740	1.083337
C	-6.266557	-0.858721	2.188304
C	-7.425912	-0.857141	0.088821
C	-6.305506	0.527841	2.211937
H	-5.723690	-1.378610	2.969939
C	-7.464697	0.534570	0.111893
H	-7.883311	-1.389904	-0.740675
C	-6.825123	1.261066	1.132681
H	-5.791145	1.047565	3.012519
H	-7.949405	1.064953	-0.703225
C	-6.423776	2.678456	0.988426
C	-6.075944	3.174259	-0.277978
C	-6.094209	3.477916	2.097525
C	-5.263785	4.293368	-0.417280
H	-6.329754	2.597827	-1.160781
C	-5.292057	4.603285	1.958622
H	-6.418556	3.179828	3.090104
C	-4.772918	4.980500	0.705990
H	-4.896090	4.548107	-1.405767
H	-5.014829	5.163517	2.846263
C	-3.579420	5.847185	0.582153
C	-2.640392	5.878240	1.627292
C	-3.199497	6.444527	-0.633465
C	-1.342647	6.325801	1.420428
H	-2.879899	5.414210	2.577586
C	-1.898762	6.888420	-0.842387
H	-3.915714	6.513830	-1.446845
C	-0.915562	6.760607	0.155512
H	-0.616021	6.196158	2.215244
H	-1.627969	7.291042	-1.813997
C	0.533314	6.798723	-0.144391
C	0.988582	6.351121	-1.395807
C	1.501586	7.009143	0.853269
C	2.309567	5.968564	-1.584881
H	0.273406	6.161263	-2.188751
C	2.824749	6.626415	0.663700
H	1.203817	7.425578	1.811095
C	3.239607	6.015754	-0.532328
H	2.584348	5.492562	-2.519824
C	4.471991	5.200987	-0.622989
C	4.912087	4.491241	0.506515
C	5.073632	4.885525	-1.854524
C	5.770435	3.405431	0.384378
H	4.463272	4.693742	1.473211
C	5.932359	3.799536	-1.976896
H	4.818347	5.461187	-2.739365
C	6.228452	2.980387	-0.873033
H	5.965006	2.791822	1.258076
H	6.329720	3.544713	-2.955120
C	6.713641	1.591363	-1.029696
C	1.207035	-0.903111	-3.288950
C	1.703607	0.447840	-3.154132
C	-0.169358	-1.116542	-3.426234
C	0.797912	1.514728	-3.166300
C	5.998275	3.757046	1.907293
H	4.904893	5.427661	2.680468
C	6.269683	2.929979	0.803152
H	5.959039	2.728699	-1.321135
H	6.414095	3.507094	2.879120
C	6.751924	1.540091	0.962634
C	6.308095	0.804130	2.072491
C	7.401428	0.828336	-0.061748
C	6.345536	-0.582735	2.076690
H	5.789661	1.313498	2.877214
C	7.438108	-0.563461	-0.058475
H	7.834082	1.370862	-0.898120
C	6.829088	-1.302497	0.972034
H	5.854789	-1.112035	2.885749
H	7.895761	-1.083661	-0.895499
C	6.419415	-2.716800	0.820793
C	6.029450	-3.193343	-0.440791
C	6.123178	-3.531084	1.928587
C	5.211429	-4.309424	-0.569653
H	6.253822	-2.603750	-1.323018
C	5.313953	-4.652670	1.799765
H	6.479241	-3.247175	2.914447
C	4.755411	-5.011653	0.558917
H	4.810466	-4.548927	-1.548969
H	5.062256	-5.224144	2.687933
C	3.556287	-5.873667	0.459156
C	2.643288	-5.907976	1.526993
C	3.143795	-6.459936	-0.751098
C	1.337795	-6.345462	1.348192
H	2.908884	-5.451851	2.474203
C	1.835588	-6.894041	-0.931650
H	3.839790	-6.526643	-1.582099
C	0.877490	-6.766514	0.090408
H	0.631012	-6.215764	2.160663
H	1.538590	-7.287234	-1.899467
C	-0.578048	-6.788572	-0.177530
C	-1.054249	-6.323988	-1.414881
C	-1.527738	-7.000695	0.837383
C	-2.376007	-5.931803	-1.574956
H	-0.352960	-6.129408	-2.218901
C	-2.851924	-6.607289	0.677232
H	-1.213754	-7.427095	1.785635
C	-3.286401	-5.985491	-0.505840
H	-2.665686	-5.444086	-2.499305
C	-4.517954	-5.167235	-0.570809
C	-4.933125	-4.454669	0.566052
C	-5.144929	-4.853287	-1.789973
C	-5.791144	-3.366751	0.459200
H	-4.463904	-4.656604	1.523132
C	-6.002657	-3.765076	-1.896976
H	-4.909483	-5.431753	-2.678460
C	-6.272895	-2.942112	-0.789289
H	-5.966004	-2.752440	1.335904
H	-6.418892	-3.510843	-2.867463
C	-6.750944	-1.550275	-0.944008
C	-1.340806	0.957457	-3.166671
C	-1.835847	-0.394740	-3.030708
C	0.031050	1.169473	-3.349163
C	-0.932874	-1.462462	-3.087845

C	0.965323	2.616736	-2.232654	C	-1.068066	-2.574787	-2.160977
C	2.799049	0.433370	-2.198823	C	-2.896280	-0.389983	-2.037051
C	2.955923	1.491878	-1.300352	C	-3.019095	-1.456628	-1.138827
C	2.022008	2.608629	-1.322107	C	-2.087972	-2.572908	-1.208590
C	-0.049955	-3.053589	-1.897330	C	-0.030007	3.084844	-1.789072
C	1.377433	-2.814487	-1.738168	C	-1.450574	2.845187	-1.582086
C	-0.808991	-2.212594	-2.718430	C	0.698019	2.254102	-2.648397
C	1.993993	-1.767898	-2.420601	C	-2.093423	1.810304	-2.257352
C	2.980401	-0.937503	-1.744231	C	-3.055131	0.968690	-1.558362
C	1.717238	-3.079934	-0.348735	C	-1.737044	3.090904	-0.176237
C	2.659003	-2.284810	0.299409	C	-2.647305	2.275558	0.497301
C	3.304348	-1.190176	-0.411524	C	-3.318148	1.194396	-0.204660
C	-2.632366	-2.163799	-1.109374	C	2.575331	2.177978	-1.104202
C	-1.852081	-3.013097	-0.231562	C	1.827950	3.014427	-0.187390
C	-2.130019	-1.788193	-2.334834	C	2.030087	1.821836	-2.317733
C	-0.586861	-3.463416	-0.618397	C	0.551909	3.474338	-0.523133
C	0.503409	-3.482892	0.342354	C	-0.504652	3.483025	0.474662
C	-2.080714	-2.585454	1.138901	C	2.102054	2.563438	1.167505
C	-1.040016	-2.621783	2.067812	C	1.092491	2.582528	2.131057
C	0.283218	-3.075072	1.659177	C	-0.242518	3.045869	1.777191
C	-3.269344	0.583687	-2.111067	C	3.172603	-0.555007	-2.168530
C	-3.546372	0.107236	-0.684294	C	3.501779	-0.100340	-0.746013
C	-2.480869	-0.476617	-3.030325	C	2.353906	0.520047	-3.044145
C	-3.361120	-1.190511	-0.260894	C	3.332455	1.191262	-0.296514
C	-3.006659	-1.466159	1.117273	C	3.028131	1.445969	1.096898
C	-3.339784	1.186634	0.244880	C	3.328456	-1.193080	0.173062
C	-3.025549	0.944066	1.585288	C	3.064378	-0.970855	1.528155
C	-2.851399	-0.419024	2.032996	C	2.907102	0.385929	2.002641
C	-0.620713	1.271941	-3.336290	C	0.478808	-1.220411	-3.306138
C	-1.345768	2.247976	-2.488189	C	1.232145	-2.209943	-2.498848
C	-1.093680	-0.014195	-3.478316	C	0.950272	0.066775	-3.449525
C	-2.499204	1.875144	-1.832376	C	2.411013	-1.849489	-1.881442
C	-2.694648	2.277683	-0.465272	C	2.656223	-2.272898	-0.529392
C	-0.355248	3.065991	-1.818148	C	0.265509	-3.035518	-1.804579
C	-0.562495	3.489495	-0.500888	C	0.521146	-3.481797	-0.503507
C	-1.762682	3.086092	0.190419	C	1.748435	-3.091200	0.148257
C	-1.255165	0.913716	3.134590	C	1.352003	-0.957908	3.138643
C	-2.040308	1.776138	2.263884	C	2.103353	-1.811523	2.228447
C	-1.423703	2.822716	1.581815	C	1.461173	-2.847283	1.553874
C	0.114780	1.137404	3.286496	C	-0.017110	-1.174387	3.322955
C	0.553756	-1.290650	3.172134	C	-0.457023	1.236512	3.277571
C	-0.870343	-1.523164	3.008192	C	0.956695	1.471509	3.062012
C	-1.759174	-0.445287	2.991259	C	1.851362	0.397142	3.000935
C	1.037435	0.013347	3.303004	C	-0.937523	-0.060836	3.403120
C	2.934202	-0.502947	1.809739	C	-2.998579	0.506341	2.063512
C	2.432468	-1.861213	1.671183	C	-2.384381	1.838306	1.849948
C	1.267285	-2.250279	2.337349	C	-1.204005	2.217849	2.476703
C	2.250471	0.414288	2.607479	C	-2.254203	-0.470866	2.859553
C	2.584294	2.183115	0.919872	C	-2.567593	-2.189697	1.058820
C	3.301704	1.224744	0.083647	C	-3.319632	-1.207966	0.255739
C	3.474325	-0.088505	0.520616	C	-3.468385	0.091145	0.715349
C	2.074698	1.786842	2.155494	C	-2.012665	-1.810607	2.270103
C	0.002198	3.063088	1.742852	C	0.040391	-3.088421	1.760057
C	0.535473	3.474132	0.451759	C	-0.539474	-3.480229	0.490304
C	1.798976	3.040001	0.051481	C	-1.816789	-3.027993	0.147921
C	0.756784	2.234658	2.576011	C	-0.684275	-2.258147	2.621305
H	3.533552	6.750737	1.477264	H	-3.545738	-6.733576	1.503459
C	-3.481443	-0.648563	-4.253595	C	3.312654	0.708600	-4.297976
C	-4.575381	0.842395	-2.979024	C	4.447432	-0.802474	-3.084420

C	-3.989921	0.797177	-4.402502	C	3.813519	-0.735294	-4.486063
H	-3.194012	1.527789	-4.575133	H	3.010683	-1.462203	-4.641558
H	-4.758635	0.894324	-5.173853	H	4.554919	-0.821725	-5.285015
C	-5.375869	-0.444604	-2.954877	C	5.250420	0.483005	-3.068390
H	-5.093744	1.750298	-2.673528	H	4.974471	-1.715490	-2.809717
C	-4.722071	-1.335626	-3.712644	C	4.572288	1.386128	-3.789611
H	-6.238476	-0.612684	-2.323428	H	6.133944	0.640590	-2.463474
H	-2.999025	-1.112294	-5.115103	H	2.802055	1.185800	-5.135577
H	-4.946495	-2.389386	-3.834541	H	4.793260	2.441721	-3.901767
H	3.664867	1.349729	5.578520	H	-2.918930	-1.199303	5.297485
C	4.299216	0.752770	4.934542	C	-3.558318	-0.716271	4.567244
H	5.377751	2.244504	3.774891	H	-4.843726	-2.403098	3.913782
C	5.178208	1.205217	4.014379	C	-4.660610	-1.334802	3.939706
C	4.327949	-0.752325	4.945094	C	-3.845297	0.768147	4.571996
H	3.340508	-1.185691	4.733148	H	-2.969855	1.419354	4.590775
H	4.630058	-1.147592	5.927024	H	-4.464610	1.005418	5.452073
C	5.820624	0.065099	3.355360	C	-5.355698	-0.378685	3.195817
C	5.334406	-1.082645	3.876069	C	-4.707968	0.866396	3.335022
H	6.547862	0.149865	2.558124	H	-6.155227	-0.581661	2.494813
H	5.613176	-2.092975	3.601313	H	-5.096165	1.805308	2.960629
**C60CPP10_2_Prod**				**C60CPP10_3_Int**			
182				193			
C60CPP10_2_Prod_2 SCF Done: -4986.68024206 A.U.				C60CPP10_3_Int_3 SCF Done: -5180.86371763 A.U.			
C	6.170647	3.477633	1.888963	C	5.531494	4.371620	1.532938
C	5.372022	4.607320	1.764575	C	4.566396	5.370716	1.534374
C	4.807293	4.969617	0.527567	C	3.863773	5.708400	0.362782
C	5.246129	4.260170	-0.603293	C	4.316876	5.135831	-0.837732
C	6.053208	3.135716	-0.478920	C	5.288418	4.142398	-0.839269
C	6.449149	2.658109	0.780422	C	5.845275	3.665470	0.357905
H	6.532086	3.192073	2.872383	H	6.004205	4.092460	2.469933
H	5.133429	5.183199	2.653534	H	4.310932	5.854453	2.472250
H	4.839301	4.502070	-1.579598	H	3.805367	5.363828	-1.766965
H	6.263625	2.541740	-1.361687	H	5.514910	3.633247	-1.769797
C	3.616573	5.843815	0.434975	C	2.552022	6.392458	0.403843
C	2.709827	5.886627	1.507802	C	1.727328	6.228778	1.529853
C	3.203279	6.433972	-0.773068	C	1.970329	6.982457	-0.732748
C	1.407125	6.335121	1.335585	C	0.362905	6.478457	1.468392
H	2.976759	5.428731	2.453819	H	2.126617	5.761355	2.423185
C	1.898042	6.879389	-0.946883	C	0.603777	7.229266	-0.795653
H	3.895772	6.494733	-1.607457	H	2.585322	7.199480	-1.601192
C	0.943998	6.759273	0.079876	C	-0.246267	6.900515	0.275876
H	0.703399	6.211688	2.151680	H	-0.254885	6.197557	2.314488
H	1.599484	7.275446	-1.913043	H	0.181231	7.631222	-1.711826
C	6.845837	1.240117	0.930996	C	6.462457	2.320807	0.389381
C	7.437588	0.493055	-0.103806	C	7.076729	1.733336	-0.731730
C	6.365348	0.527375	2.041489	C	6.187327	1.482666	1.482183
C	7.386416	-0.898217	-0.104261	C	7.240008	0.353919	-0.820299
H	7.892991	1.007057	-0.945861	H	7.378074	2.357553	-1.568405
C	6.313473	-0.859033	2.040139	C	6.351898	0.107894	1.394112
H	5.887956	1.063146	2.854280	H	5.697028	1.891231	2.358952
H	7.805783	-1.446585	-0.943576	H	7.667682	-0.075576	-1.722291
H	5.797619	-1.361612	2.850778	H	5.988666	-0.513194	2.205019
C	-0.512426	6.790883	-0.181932	C	-1.705458	6.720214	0.105767
C	-0.995821	6.328196	-1.417227	C	-2.199124	6.260167	-1.126439
C	-1.456920	7.008654	0.836549	C	-2.597394	6.727948	1.192557
C	-2.320365	5.943707	-1.572403	C	-3.456889	5.681814	-1.226085
H	-0.298597	6.129565	-2.223786	H	-1.538746	6.220536	-1.985765
C	-2.783885	6.622561	0.681457	C	-3.857253	6.147699	1.093018
H	-1.136904	7.433377	1.783543	H	-2.279521	7.142070	2.144961

C	-3.226306	6.003165	-0.499943	C	-4.282295	5.535390	-0.098440
H	-2.616814	5.458048	-2.495725	H	-3.740309	5.209708	-2.160453
H	-3.474023	6.752638	1.510147	H	-4.498457	6.119442	1.969362
C	-4.463540	5.193458	-0.560678	C	-5.381232	4.545026	-0.137199
C	-4.877146	4.480977	0.576752	C	-5.597459	3.712986	0.973415
C	-5.099509	4.887624	-1.777167	C	-6.048375	4.211943	-1.329582
C	-5.746214	3.401606	0.472862	C	-6.297167	2.518605	0.852132
H	-4.400422	4.675802	1.531587	H	-5.087891	3.925604	1.907248
C	-5.968820	3.808200	-1.881529	C	-6.748238	3.017246	-1.451401
H	-4.863718	5.466093	-2.665599	H	-5.967897	4.869182	-2.190459
C	-6.241397	2.986404	-0.773739	C	-6.812887	2.102388	-0.385804
H	-5.918694	2.786483	1.348920	H	-6.309948	1.835402	1.693996
H	-6.393661	3.560496	-2.850010	H	-7.198634	2.760888	-2.405989
C	-6.739355	1.601433	-0.926746	C	-7.107055	0.667848	-0.597506
C	-6.313565	0.859069	-2.039372	C	-6.657426	0.061439	-1.780862
C	-7.386829	0.898205	0.104857	C	-7.564552	-0.181683	0.425705
C	-6.365433	-0.527343	-2.040761	C	-6.498069	-1.314179	-1.867635
H	-5.797559	1.361633	-2.849922	H	-6.284765	0.680804	-2.589140
C	-7.437989	-0.493065	0.104371	C	-7.404392	-1.561754	0.339167
H	-7.806339	1.446568	0.944105	H	-7.999726	0.248990	1.323562
C	-6.846083	-1.240112	-0.930365	C	-6.781421	-2.151093	-0.776162
H	-5.887906	-1.063078	-2.853494	H	-6.005309	-1.724537	-2.741920
H	-7.893506	-1.007093	0.946350	H	-7.714685	-2.184496	1.173729
C	-6.449396	-2.658112	-0.779795	C	-6.160440	-3.494092	-0.737699
C	-6.053515	-3.135785	0.479533	C	-5.614653	-3.968504	0.465587
C	-6.170846	-3.477591	-1.888371	C	-5.830699	-4.200418	-1.908273
C	-5.246399	-4.260230	0.603870	C	-4.637949	-4.956686	0.475547
H	-6.264007	-2.541896	1.362340	H	-5.856217	-3.461926	1.393522
C	-5.372245	-4.607281	-1.764016	C	-4.862096	-5.196185	-1.898031
H	-6.532280	-3.191979	-2.871778	H	-6.293876	-3.924401	-2.850897
C	-4.807539	-4.969629	-0.527003	C	-4.170034	-5.530107	-0.719064
H	-4.839593	-4.502162	1.580177	H	-4.136963	-5.181618	1.411220
H	-5.133631	-5.183144	-2.652981	H	-4.596556	-5.680363	-2.832798
C	-3.616825	-5.843840	-0.434451	C	-2.857644	-6.214015	-0.745632
C	-2.710123	-5.886656	-1.507317	C	-2.029471	-6.074630	-1.872554
C	-3.203487	-6.434013	0.773568	C	-2.279406	-6.782991	0.403562
C	-1.407414	-6.335149	-1.335156	C	-0.665645	-6.325692	-1.802706
H	-2.977101	-5.428767	-2.453325	H	-2.424988	-5.625372	-2.776737
C	-1.898238	-6.879419	0.947328	C	-0.913396	-7.029575	0.475436
H	-3.895947	-6.494784	1.607984	H	-2.896575	-6.983588	1.274290
C	-0.944231	-6.759290	-0.079466	C	-0.060156	-6.723917	-0.600140
H	-0.703727	-6.211705	-2.151285	H	-0.045264	-6.063473	-2.652971
H	-1.599644	-7.275476	1.913478	H	-0.493899	-7.413741	1.400637
C	0.512193	-6.790859	0.182320	C	1.398724	-6.542714	-0.429066
C	0.995571	-6.328188	1.417623	C	1.888741	-6.054562	0.793912
C	1.456698	-7.008579	-0.836167	C	2.294465	-6.579761	-1.511997
C	2.320107	-5.943686	1.572821	C	3.149113	-5.480446	0.885286
H	0.298340	-6.129580	2.224183	H	1.224137	-5.990524	1.648546
C	2.783658	-6.622467	-0.681056	C	3.556243	-6.002146	-1.421338
H	1.136699	-7.433270	-1.783181	H	1.977856	-7.014409	-2.455609
C	3.226062	-6.003118	0.500376	C	3.979535	-5.365896	-0.242187
H	2.616541	-5.458037	2.496155	H	3.432498	-4.987674	1.809220
C	4.463280	-5.193405	0.561189	C	5.081469	-4.378688	-0.220826
C	4.876871	-4.480821	-0.576195	C	5.281574	-3.550553	-1.337463
C	5.099204	-4.887662	1.777718	C	5.765060	-4.042555	0.960809
C	5.745893	-3.401450	-0.472159	C	5.978185	-2.353209	-1.228657
H	4.400140	-4.675571	-1.531043	H	4.759589	-3.768241	-2.263183
C	5.968518	-3.808236	1.882209	C	6.465602	-2.846505	1.068789
H	4.863388	-5.466207	2.666094	H	5.694964	-4.696807	1.824993

C	6.241086	-2.986370	0.774491	C	6.509559	-1.933008	0.001356
H	5.918403	-2.786057	-1.348014	H	5.975258	-1.670505	-2.071260
H	6.393330	-3.560616	2.850727	H	6.929273	-2.586035	2.015760
C	6.739095	-1.601411	0.927452	C	6.796891	-0.496596	0.208117
C	1.387153	0.949183	3.139466	C	1.315483	1.052834	2.860359
C	1.867978	-0.407571	2.998053	C	1.982598	-0.201202	2.587009
C	0.018572	1.173874	3.334011	C	-0.051509	1.049349	3.164225
C	0.954946	-1.466409	3.062153	C	1.247559	-1.389637	2.633649
C	1.069874	-2.578414	2.132328	C	1.454955	-2.409419	1.619243
C	2.916668	-0.411070	1.991900	C	2.935516	0.021520	1.512767
C	3.015236	-1.473030	1.085781	C	3.124777	-0.954328	0.528253
C	2.075718	-2.578971	1.165107	C	2.372515	-2.197446	0.590518
C	0.084069	3.089049	1.772824	C	-0.399090	3.042676	1.743483
C	1.500191	2.835189	1.553077	C	1.017193	3.027783	1.410569
C	-0.644507	2.264782	2.638506	C	-0.924182	2.062946	2.594951
C	2.138882	1.794259	2.222495	C	1.855858	2.059866	1.957701
C	3.084881	0.943408	1.514566	C	2.861036	1.412033	1.126227
C	1.775708	3.075385	0.144805	C	1.142173	3.393763	0.007249
C	2.668382	2.246822	-0.541284	C	2.093182	2.758618	-0.796426
C	3.336991	1.160887	0.155989	C	2.970474	1.749117	-0.226810
C	-2.534921	2.204367	1.109532	C	-2.897773	1.801589	1.196779
C	-1.786742	3.031562	0.185940	C	-2.358026	2.790588	0.286775
C	-1.982573	1.844474	2.319302	C	-2.204076	1.458826	2.336645
C	-0.504517	3.481731	0.511024	C	-1.137249	3.414585	0.555986
C	0.545112	3.475945	-0.494543	C	-0.180946	3.634633	-0.516389
C	-2.075481	2.578956	-1.165600	C	-2.679464	2.382008	-1.071373
C	-1.069638	2.578391	-2.132821	C	-1.764083	2.598027	-2.102383
C	0.271312	3.028810	-1.791632	C	-0.484402	3.230765	-1.821330
C	-3.150009	-0.521619	2.181927	C	-3.008169	-1.047979	2.107699
C	-3.488480	-0.065558	0.762287	C	-3.521459	-0.564138	0.750964
C	-2.312625	0.546326	3.049242	C	-2.276269	0.083082	2.991666
C	-3.309403	1.224137	0.309617	C	-3.574658	0.763041	0.382072
C	-3.015001	1.473015	-1.086278	C	-3.431231	1.139951	-1.008885
C	-3.336753	-1.160911	-0.156495	C	-3.277784	-1.563888	-0.253415
C	-3.084640	-0.943427	-1.515070	C	-3.168327	-1.226669	-1.606660
C	-2.916434	0.411056	-1.992402	C	-3.244463	0.164145	-1.994288
C	-0.451712	-1.211808	3.294614	C	-0.157785	-1.373797	2.981602
C	-1.222430	-2.194777	2.494999	C	-0.831646	-2.414272	2.166435
C	-0.909704	0.080206	3.442458	C	-0.792728	-0.181626	3.255154
C	-2.404447	-1.823909	1.888899	C	-2.099858	-2.197532	1.670809
C	-2.668143	-2.246846	0.540783	C	-2.400844	-2.573214	0.316026
C	-0.271074	-3.028834	1.791138	C	0.176711	-3.044645	1.340112
C	-0.544871	-3.475961	0.494047	C	-0.126293	-3.449457	0.035906
C	-1.775469	-3.075406	-0.145305	C	-1.449644	-3.208253	-0.487755
C	-1.386907	-0.949196	-3.139965	C	-1.621419	-0.867378	-3.339104
C	-2.138638	-1.794273	-2.222998	C	-2.162776	-1.874060	-2.437274
C	-1.499948	-2.835204	-1.553575	C	-1.324151	-2.842392	-1.890359
C	-0.018328	-1.173892	-3.334511	C	-0.254017	-0.863219	-3.642057
C	0.451946	1.211785	-3.295104	C	-0.148551	1.559692	-3.461543
C	-0.954709	1.466384	-3.062640	C	-1.555160	1.576657	-3.116140
C	-1.867734	0.407556	-2.998545	C	-2.289369	0.386297	-3.066970
C	0.909946	-0.080225	-3.442951	C	0.486953	0.366805	-3.734002
C	3.150252	0.521586	-2.182439	C	2.701516	1.233240	-2.586515
C	2.404688	1.823883	-1.889397	C	1.792478	2.382966	-2.150767
C	1.222668	2.194756	-2.495496	C	0.524168	2.600617	-2.646928
C	2.312870	-0.546337	-3.049716	C	1.970783	0.102383	-3.470958
C	2.535161	-2.204384	-1.110025	C	2.591970	-1.617030	-1.676843
C	3.309645	-1.224155	-0.310114	C	3.268336	-0.578047	-0.862127
C	3.488736	0.065538	-0.762792	C	3.213958	0.749499	-1.230304

C	1.982814	-1.844494	-2.319794
C	-0.083825	-3.089064	-1.773318
C	0.504756	-3.481746	-0.511517
C	1.786978	-3.031574	-0.186430
C	0.644750	-2.264796	-2.639000
H	3.473799	-6.752489	-1.509752
C	-3.258652	0.745347	4.311195
C	-4.419061	-0.755576	3.109373
C	-3.772148	-0.693292	4.505232
H	-2.975322	-1.428079	4.654590
H	-4.507364	-0.771347	5.310705
C	-5.209357	0.537792	3.098912
H	-4.957422	-1.663569	2.839897
C	-4.515917	1.434863	3.813107
H	-6.096536	0.703484	2.501481
H	-2.735988	1.218343	5.143700
H	-4.725348	2.492713	3.926042
H	2.736151	-1.218371	-5.144179
C	3.258848	-0.745357	-4.311705
H	4.725590	-2.492703	-3.926696
C	4.516132	-1.434866	-3.813677
C	3.772304	0.693297	-4.505763
H	2.975450	1.428062	-4.655070
H	4.507478	0.771379	-5.311271
C	5.209652	-0.537774	-3.099593
C	4.419302	0.755581	-3.109943
H	6.096967	-0.703324	-2.502307
H	4.957660	1.663590	-2.840504

C	1.898075	-1.273883	-2.816585
C	0.092815	-2.857803	-2.223320
C	0.830806	-3.231181	-1.036388
C	2.051929	-2.606535	-0.767093
C	0.618496	-1.877987	-3.074510
H	4.200858	-5.994765	-2.295666
C	-3.140745	0.055599	4.325221
C	-4.150323	-1.533080	3.100427
C	-3.413224	-1.454635	4.450000
H	-2.504682	-2.062980	4.491461
H	-4.062885	-1.695694	5.295789
C	-5.126098	-0.381149	3.236176
H	-4.563981	-2.496317	2.804422
C	-4.523103	0.568612	3.964244
H	-6.071835	-0.320241	2.713192
H	-2.632462	0.553564	5.152088
H	-4.880958	1.572031	4.166174
H	2.332331	-0.367740	-5.631286
C	2.838601	0.129433	-4.802749
H	4.575605	-1.390236	-4.637981
C	4.219052	-0.386162	-4.437152
C	3.114609	1.639156	-4.927146
H	2.207516	2.249440	-4.971387
H	3.766914	1.878417	-5.771365
C	4.821676	0.562505	-3.707228
C	3.847575	1.716713	-3.575327
H	5.766003	0.498754	-3.181340
H	4.262463	2.679523	-3.278045
C	3.120379	-3.173946	4.601794
C	2.580099	-2.394670	5.562435
C	3.319877	-1.129839	5.633417
C	4.282034	-2.450726	3.975527
H	2.791130	-4.162180	4.302999
C	4.309165	-1.139960	4.715315
H	4.154389	-2.317714	2.893037
H	5.223322	-3.008333	4.098219
H	1.725592	-2.640429	6.183825
H	3.084618	-0.319696	6.315318
H	5.024873	-0.349590	4.521918

C60CPP10_3_TS

193

C60CPP10_3_TS_3 SCF Done: -5180.83693564 A.U.

C	5.932860	3.882522	1.564585
C	5.064542	4.966378	1.532229
C	4.397203	5.330853	0.348211
C	4.796478	4.683546	-0.832943
C	5.669632	3.603249	-0.800524
C	6.178396	3.113504	0.412833
H	6.378474	3.590529	2.511032
H	4.852978	5.500553	2.453649
H	4.307622	4.929292	-1.769803
H	5.843791	3.044565	-1.713693
C	3.151048	6.128672	0.363174
C	2.302918	6.049487	1.480547
C	2.636602	6.752370	-0.787523
C	0.966599	6.416702	1.399740
H	2.650516	5.557389	2.382226
C	1.297986	7.118291	-0.869274
H	3.278278	6.902086	-1.650972

C60CPP10_3_Prod

193

C60CPP10_3_Prod_3 SCF Done: -5180.87565382 A.U.

C	6.010382	3.766173	1.576003
C	5.163717	4.866759	1.535120
C	4.509783	5.239836	0.346324
C	4.902247	4.580286	-0.830329
C	5.753958	3.483287	-0.789420
C	6.246794	2.988118	0.428365
H	6.445449	3.469201	2.525784
H	4.958144	5.408563	2.453436
H	4.423348	4.832297	-1.770673
H	5.922268	2.918038	-1.699649
C	3.279690	6.062279	0.352015
C	2.425195	6.005215	1.465876
C	2.783042	6.691196	-0.803681
C	1.097035	6.399132	1.377493
H	2.758634	5.510142	2.371247
C	1.452465	7.083704	-0.893095
H	3.431444	6.824176	-1.664835

C	0.411043	6.878523	0.195856	C	0.556265	6.866878	0.169186
H	0.316260	6.199066	2.240028	H	0.438767	6.198646	2.215884
H	0.922097	7.544467	-1.794789	H	1.089344	7.513260	-1.822145
C	6.662612	1.717267	0.486625	C	6.702870	1.582823	0.510018
C	7.208399	1.038039	-0.617912	C	7.239644	0.888112	-0.589366
C	6.316960	0.943107	1.606204	C	6.336462	0.820440	1.631014
C	7.232426	-0.352097	-0.665235	C	7.233974	-0.502358	-0.631379
H	7.564395	1.603501	-1.474526	H	7.611924	1.442602	-1.446251
C	6.342651	-0.443311	1.559156	C	6.332111	-0.566425	1.589316
H	5.880746	1.426566	2.473768	H	5.907505	1.315759	2.495468
H	7.609316	-0.847397	-1.555843	H	7.604813	-1.009078	-1.518114
H	5.931185	-1.001505	2.392980	H	5.904327	-1.111563	2.423594
C	-1.056584	6.827880	0.012112	C	-0.911285	6.845069	-0.020440
C	-1.578156	6.397007	-1.219028	C	-1.436648	6.418671	-1.251525
C	-1.953734	6.931403	1.089575	C	-1.810306	6.971787	1.052999
C	-2.882168	5.933178	-1.323919	C	-2.749167	5.980030	-1.359143
H	-0.915998	6.285150	-2.070572	H	-0.773693	6.289955	-2.100068
C	-3.259538	6.465036	0.985049	C	-3.124618	6.530720	0.945739
H	-1.608055	7.328582	2.039520	H	-1.460446	7.366917	2.002261
C	-3.726892	5.876924	-0.202640	C	-3.598956	5.945924	-0.240820
H	-3.199097	5.475270	-2.254569	H	-3.071733	5.524093	-2.288848
H	-3.908644	6.505846	1.855089	H	-3.776151	6.588829	1.812977
C	-4.908482	4.986901	-0.238711	C	-4.797410	5.078833	-0.276567
C	-5.199609	4.186811	0.878165	C	-5.107451	4.290136	0.843296
C	-5.598588	4.703663	-1.430778	C	-5.489577	4.803493	-1.469271
C	-6.002418	3.058521	0.764829	C	-5.932543	3.177754	0.733218
H	-4.673956	4.360857	1.811049	H	-4.581040	4.458307	1.776843
C	-6.401872	3.574958	-1.544510	C	-6.315115	3.690628	-1.579744
H	-5.456608	5.343063	-2.297172	H	-5.332455	5.435685	-2.338338
C	-6.551076	2.679670	-0.470865	C	-6.485443	2.803901	-0.502097
H	-6.076806	2.385792	1.611800	H	-6.022968	2.511110	1.583372
H	-6.870518	3.350587	-2.498402	H	-6.785683	3.471138	-2.533817
C	-6.972877	1.275500	-0.669591	C	-6.935522	1.407762	-0.695053
C	-6.573981	0.617769	-1.843575	C	-6.548492	0.736537	-1.865370
C	-7.511180	0.482606	0.359745	C	-7.491615	0.630880	0.337058
C	-6.540541	-0.767551	-1.914931	C	-6.544195	-0.649471	-1.930602
H	-6.141025	1.191664	-2.655370	H	-6.102292	1.297541	-2.679009
C	-7.477883	-0.907047	0.288302	C	-7.487522	-0.759465	0.271793
H	-7.909327	0.961074	1.250562	H	-7.881108	1.121543	1.225060
C	-6.905472	-1.563123	-0.816877	C	-6.927854	-1.432342	-0.829816
H	-6.082062	-1.230415	-2.781556	H	-6.094216	-1.125777	-2.794404
H	-7.848731	-1.489387	1.127264	H	-7.871786	-1.330178	1.112698
C	-6.412166	-2.957428	-0.759589	C	-6.464952	-2.836749	-0.766187
C	-5.931706	-3.471352	0.455342	C	-5.995737	-3.355223	0.451177
C	-6.132911	-3.701384	-1.920057	C	-6.201470	-3.591685	-1.923289
C	-5.054499	-4.548193	0.487355	C	-5.141701	-4.450366	0.488283
H	-6.137573	-2.936894	1.376208	H	-6.189795	-2.811967	1.369428
C	-5.263717	-4.784644	-1.887956	C	-5.355495	-4.693034	-1.886101
H	-6.553082	-3.390426	-2.871860	H	-6.614862	-3.276068	-2.876537
C	-4.626321	-5.174325	-0.695627	C	-4.726768	-5.090640	-0.691791
H	-4.591110	-4.811880	1.432257	H	-4.683872	-4.719565	1.434347
H	-5.030338	-5.298948	-2.815242	H	-5.132650	-5.216398	-2.810928
C	-3.386693	-5.982793	-0.694067	C	-3.503960	-5.924168	-0.685816
C	-2.524791	-5.928270	-1.802759	C	-2.638259	-5.887824	-1.792258
C	-2.892153	-6.600666	0.468784	C	-3.024686	-6.549759	0.479255
C	-1.192425	-6.306337	-1.701056	C	-1.313634	-6.291074	-1.686302
H	-2.856198	-5.446958	-2.716159	H	-2.958055	-5.401550	-2.707202
C	-1.557647	-6.974376	0.572622	C	-1.697871	-6.948871	0.587253
H	-3.545906	-6.738820	1.324875	H	-3.683404	-6.673960	1.333700

C	-0.655210	-6.752768	-0.483135	C	-0.788371	-6.745725	-0.466228
H	-0.530035	-6.106325	-2.536506	H	-0.645248	-6.104980	-2.520195
H	-1.196881	-7.392935	1.507715	H	-1.347777	-7.373119	1.523822
C	0.808736	-6.698993	-0.274266	C	0.675661	-6.718678	-0.252976
C	1.303511	-6.235102	0.956061	C	1.175074	-6.263815	0.978856
C	1.728870	-6.830795	-1.328727	C	1.596381	-6.866717	-1.304767
C	2.605065	-5.769467	1.077808	C	2.484380	-5.821658	1.104861
H	0.621511	-6.100601	1.788544	H	0.493522	-6.118008	1.809833
C	3.032364	-6.361315	-1.207429	C	2.907758	-6.420884	-1.179300
H	1.403012	-7.250890	-2.275816	H	1.266055	-7.281071	-2.252846
C	3.474154	-5.742670	-0.025343	C	3.357090	-5.810408	0.004254
H	2.903017	-5.288763	2.003433	H	2.787461	-5.346414	2.031522
C	4.652104	-4.848333	0.013295	C	4.552012	-4.939060	0.046789
C	4.962471	-4.074437	-1.116694	C	4.885411	-4.175421	-1.083655
C	5.318869	-4.533509	1.210920	C	5.217589	-4.633917	1.247583
C	5.758737	-2.940574	-1.015868	C	5.705646	-3.058793	-0.981712
H	4.455485	-4.273024	-2.054798	H	4.380373	-4.366442	-2.024426
C	6.119542	-3.401078	1.311129	C	6.041511	-3.518460	1.349150
H	5.164640	-5.153647	2.089374	H	5.044684	-5.247012	2.127383
C	6.286442	-2.530649	0.219212	C	6.234840	-2.656124	0.255154
H	5.844412	-2.287833	-1.877776	H	5.811158	-2.411439	-1.845438
H	6.575703	-3.157285	2.266416	H	6.495886	-3.281060	2.306788
C	6.712824	-1.124669	0.389243	C	6.692038	-1.259613	0.423114
C	1.438419	0.907298	2.838365	C	1.461352	0.894906	2.859421
C	2.073957	-0.424671	2.690325	C	2.171322	-0.453779	2.832383
C	0.089834	1.034058	3.139427	C	0.116479	1.033473	3.134584
C	1.183629	-1.591612	2.733059	C	1.177107	-1.718041	2.895450
C	1.249056	-2.553218	1.613448	C	1.193319	-2.590554	1.645395
C	2.951836	-0.280271	1.501397	C	2.957368	-0.332163	1.527489
C	3.051997	-1.267395	0.528220	C	3.025321	-1.319997	0.565783
C	2.182166	-2.443622	0.594794	C	2.121536	-2.485165	0.635008
C	-0.073395	3.042893	1.703910	C	-0.010864	3.040875	1.691348
C	1.328311	2.898205	1.376832	C	1.386660	2.872534	1.373737
C	-0.684218	2.111980	2.559753	C	-0.638374	2.117854	2.544370
C	2.055366	1.840868	1.924144	C	2.085343	1.800677	1.931672
C	2.994491	1.096926	1.100258	C	3.012122	1.032300	1.114249
C	1.494572	3.244517	-0.029950	C	1.566974	3.211157	-0.033452
C	2.381836	2.517298	-0.826806	C	2.443104	2.463769	-0.822364
C	3.154105	1.426486	-0.251107	C	3.188182	1.358924	-0.238016
C	-2.673946	2.034329	1.157206	C	-2.627877	2.082032	1.138182
C	-2.043674	2.966288	0.243878	C	-1.976315	2.998924	0.224765
C	-2.013784	1.627512	2.296008	C	-1.975054	1.659821	2.275308
C	-0.772060	3.478046	0.513363	C	-0.696509	3.488714	0.498342
C	0.202284	3.605696	-0.558365	C	0.284525	3.594987	-0.569379
C	-2.400412	2.586483	-1.112438	C	-2.335481	2.623146	-1.130764
C	-1.466109	2.711116	-2.142954	C	-1.393323	2.724937	-2.157348
C	-0.134966	3.225301	-1.864005	C	-0.054352	3.215667	-1.875580
C	-3.067163	-0.783950	2.079126	C	-3.088335	-0.721608	2.069840
C	-3.528199	-0.259579	0.718850	C	-3.533319	-0.192991	0.705593
C	-2.223569	0.271624	2.959944	C	-2.216163	0.314590	2.943356
C	-3.447753	1.064540	0.344696	C	-3.421245	1.127727	0.326559
C	-3.265859	1.420937	-1.047089	C	-3.225093	1.475214	-1.065153
C	-3.377504	-1.281655	-0.281863	C	-3.398939	-1.221164	-0.290735
C	-3.229395	-0.961553	-1.635475	C	-3.235898	-0.909264	-1.644703
C	-3.171585	0.428199	-2.028761	C	-3.147001	0.477262	-2.042890
C	-0.261492	-1.370562	2.965464	C	-0.307542	-1.373752	2.991987
C	-1.025381	-2.332224	2.143424	C	-1.080360	-2.305284	2.153114
C	-0.775899	-0.139015	3.238223	C	-0.780496	-0.130813	3.232230
C	-2.273063	-2.014392	1.648914	C	-2.320523	-1.969807	1.649292

C	-2.601700	-2.367052	0.294950	C	-2.647298	-2.319207	0.294743
C	-0.079053	-3.055281	1.325639	C	-0.140689	-3.044769	1.342779
C	-0.418013	-3.449071	0.025836	C	-0.485366	-3.441573	0.042814
C	-1.712789	-3.093008	-0.503149	C	-1.770221	-3.066401	-0.495692
C	-1.648927	-0.759114	-3.363100	C	-1.641192	-0.746592	-3.363109
C	-2.286754	-1.705350	-2.460301	C	-2.304140	-1.675749	-2.461794
C	-1.546903	-2.748065	-1.906405	C	-1.589170	-2.731527	-1.899118
C	-0.285409	-0.885715	-3.660463	C	-0.278057	-0.901966	-3.651445
C	0.047635	1.518274	-3.492686	C	0.102711	1.495885	-3.493755
C	-1.352261	1.668861	-3.150581	C	-1.295805	1.675646	-3.159216
C	-2.195575	0.553779	-3.098553	C	-2.162090	0.578502	-3.107366
C	0.568834	0.269660	-3.756301	C	0.600151	0.235984	-3.747561
C	2.850663	0.929776	-2.604641	C	2.888732	0.856917	-2.588175
C	2.053256	2.165218	-2.180731	C	2.114579	2.111455	-2.176429
C	0.812138	2.498648	-2.682884	C	0.882415	2.466173	-2.685997
C	2.019639	-0.132816	-3.483880	C	2.040798	-0.194026	-3.464695
C	2.464587	-1.890359	-1.673163	C	2.432986	-1.944362	-1.635362
C	3.231189	-0.916004	-0.862290	C	3.213835	-0.983526	-0.826390
C	3.309172	0.408548	-1.243895	C	3.328307	0.335520	-1.221014
C	1.812882	-1.491304	-2.820222	C	1.801895	-1.543033	-2.792669
C	-0.135462	-2.898305	-2.232543	C	-0.178791	-2.910601	-2.215118
C	0.557866	-3.332575	-1.040694	C	0.496603	-3.347860	-1.013927
C	1.829650	-2.820188	-0.761125	C	1.773100	-2.854101	-0.722345
C	0.484132	-1.974215	-3.084743	C	0.465060	-2.001426	-3.065101
H	3.699919	-6.423133	-2.062107	H	3.576836	-6.495263	-2.031782
C	-3.090518	0.340161	4.290494	C	-3.078076	0.411292	4.276550
C	-4.252441	-1.146485	3.072706	C	-4.282618	-1.046611	3.065609
C	-3.512543	-1.134402	4.423142	C	-3.540980	-1.050090	4.415205
H	-2.668058	-1.828833	4.467870	H	-2.716153	-1.767381	4.462338
H	-4.183579	-1.305664	5.269415	H	-4.215550	-1.198797	5.262893
C	-5.108515	0.097738	3.201146	C	-5.103169	0.221456	3.190278
H	-4.760034	-2.065310	2.781438	H	-4.815984	-1.951993	2.778421
C	-4.414142	0.986729	3.924752	C	-4.383323	1.093518	3.909729
H	-6.043164	0.250303	2.676570	H	-6.034168	0.398011	2.666705
H	-2.536349	0.789285	5.116047	H	-2.510124	0.847727	5.099544
H	-4.670296	2.022177	4.119544	H	-4.610367	2.136368	4.100988
H	2.348350	-0.655797	-5.637678	H	2.370092	-0.738952	-5.612949
C	2.894539	-0.201972	-4.809594	C	2.921061	-0.290613	-4.785039
H	4.473394	-1.882374	-4.615225	H	4.463732	-2.001886	-4.570224
C	4.215779	-0.845953	-4.428020	C	4.226509	-0.959134	-4.391430
C	3.316891	1.272427	-4.945428	C	3.374465	1.173775	-4.928625
H	2.473259	1.967037	-5.001338	H	2.545684	1.885245	-4.994129
H	3.994740	1.439379	-5.786997	H	4.060256	1.320785	-5.767461
C	4.902533	0.046528	-3.701514	C	4.927511	-0.075900	-3.667273
C	4.044059	1.291561	-3.588473	C	4.094435	1.187371	-3.567763
H	5.832025	-0.105560	-3.166368	H	5.850686	-0.242882	-3.125823
H	4.548817	2.212608	-3.296757	H	4.616611	2.099676	-3.279386
C	2.021132	-2.671376	4.431594	C	1.731067	-2.494058	4.168847
C	1.657564	-1.783827	5.465331	C	1.433069	-1.623509	5.374603
C	2.471851	-0.653454	5.399741	C	2.258671	-0.569737	5.320941
C	3.413043	-2.275585	3.993986	C	3.245382	-2.261191	4.003800
H	1.624136	-3.673621	4.314500	H	1.374767	-3.524950	4.206084
C	3.364596	-0.794829	4.310577	C	3.115449	-0.727710	4.079277
H	3.680329	-2.533831	2.967853	H	3.647460	-2.624485	3.053011
H	4.144136	-2.754842	4.665215	H	3.823845	-2.673191	4.835562
H	0.786029	-1.890943	6.101041	H	0.609336	-1.780699	6.061420
H	2.332239	0.253925	5.976054	H	2.247448	0.309470	5.955024
H	4.197747	-0.126159	4.122374	H	4.030585	-0.134733	4.036228
C60CPP10_4_Int				C60CPP10_4_TS			

204

C60CPP10_4_Int_3 SCF Done: -5375.05917517 A.U.

C	5.449187	-4.694308	-1.564663
C	4.404498	-5.604580	-1.664985
C	3.594983	-5.911298	-0.555630
C	4.010182	-5.414937	0.691407
C	5.059829	-4.510331	0.791914
C	5.737976	-4.050356	-0.348101
H	6.009610	-4.433127	-2.457542
H	4.174644	-6.037968	-2.633670
H	3.416212	-5.622076	1.575357
H	5.260477	-4.047456	1.751889
C	2.234515	-6.473188	-0.707765
C	1.506241	-6.197487	-1.877420
C	1.528185	-7.046752	0.364715
C	0.124733	-6.327919	-1.915211
H	2.004729	-5.735226	-2.722205
C	0.144402	-7.174096	0.328421
H	2.061700	-7.345665	1.262248
C	-0.598135	-6.736703	-0.783076
H	-0.407949	-5.963403	-2.786908
H	-0.373416	-7.567108	1.198481
C	6.464671	-2.762558	-0.290476
C	7.028486	-2.256483	0.895112
C	6.352378	-1.876283	-1.373916
C	7.287320	-0.897779	1.043260
H	7.210757	-2.924731	1.732072
C	6.613040	-0.521370	-1.226714
H	5.911986	-2.222423	-2.302779
H	7.669454	-0.529406	1.991356
H	6.373724	0.147583	-2.046107
C	-2.045239	-6.437063	-0.704430
C	-2.583043	-5.968861	0.506045
C	-2.856068	-6.341285	-1.848873
C	-3.793062	-5.289291	0.539036
H	-1.981726	-6.006104	1.407835
C	-4.066395	-5.657584	-1.816636
H	-2.506810	-6.755748	-2.790053
C	-4.521181	-5.044272	-0.637134
H	-4.103013	-4.819172	1.466246
H	-4.639735	-5.548632	-2.732868
C	-5.529486	-3.961556	-0.641877
C	-5.561700	-3.062567	-1.720530
C	-6.276172	-3.624624	0.500477
C	-6.159200	-1.814319	-1.598023
H	-4.983810	-3.279423	-2.612602
C	-6.877116	-2.376631	0.622107
H	-6.334186	-4.325338	1.328397
C	-6.754591	-1.411074	-0.391887
H	-6.026285	-1.092554	-2.396393
H	-7.392997	-2.122646	1.543558
C	-6.937762	0.033057	-0.129003
C	-6.539802	0.539857	1.117824
C	-7.226967	0.966397	-1.140234
C	-6.269004	1.889499	1.291586
H	-6.293596	-0.148163	1.918637
C	-6.958407	2.320623	-0.965212
H	-7.615553	0.618674	-2.093685
C	-6.386518	2.798980	0.228062

204

C60CPP10_4_TS_3 SCF Done: -5375.03201820 A.U.

C	5.752805	-4.285277	-1.570811
C	4.786128	-5.279926	-1.652579
C	4.019413	-5.645612	-0.531053
C	4.403909	-5.104196	0.707058
C	5.374958	-4.114306	0.788740
C	6.001638	-3.610439	-0.362158
H	6.279322	-3.985694	-2.472199
H	4.580958	-5.737877	-2.615568
H	3.838273	-5.352187	1.599019
H	5.545327	-3.627391	1.742633
C	2.710632	-6.323895	-0.659173
C	1.938777	-6.113891	-1.814357
C	2.078083	-6.954325	0.427269
C	0.572322	-6.359788	-1.823442
H	2.379263	-5.611835	-2.668667
C	0.709588	-7.197151	0.419836
H	2.654012	-7.205791	1.313104
C	-0.089624	-6.823872	-0.675527
H	-0.007548	-6.041414	-2.683086
H	0.244198	-7.630791	1.300261
C	6.625170	-2.269120	-0.320739
C	7.152140	-1.709718	0.857867
C	6.440182	-1.404326	-1.411459
C	7.303406	-0.333704	0.993345
H	7.389500	-2.353845	1.699866
C	6.594254	-0.031838	-1.277200
H	6.025253	-1.792282	-2.335498
H	7.657735	0.072172	1.936921
H	6.300222	0.608951	-2.101274
C	-1.553066	-6.637260	-0.561791
C	-2.090867	-6.196813	0.658944
C	-2.400363	-6.616183	-1.683137
C	-3.346405	-5.608508	0.720120
H	-1.462853	-6.179284	1.542992
C	-3.656879	-6.023635	-1.622431
H	-2.046839	-7.013370	-2.630195
C	-4.122648	-5.430171	-0.436728
H	-3.664719	-5.152592	1.651523
H	-4.261568	-5.968204	-2.523112
C	-5.205130	-4.421904	-0.425093
C	-5.343288	-3.554924	-1.521192
C	-5.933499	-4.111220	0.737000
C	-6.032524	-2.354445	-1.406637
H	-4.780553	-3.748128	-2.428100
C	-6.627066	-2.911226	0.850652
H	-5.911530	-4.793751	1.581859
C	-6.620286	-1.967161	-0.191522
H	-5.982366	-1.646262	-2.226553
H	-7.133994	-2.677953	1.782727
C	-6.925562	-0.537934	0.035174
C	-6.584924	0.038878	1.268448
C	-7.286875	0.338041	-1.004456
C	-6.430234	1.411309	1.401752
H	-6.294620	-0.601802	2.093909
C	-7.135227	1.714150	-0.870087
H	-7.638113	-0.068668	-1.948802
C	-6.612449	2.275009	0.309632

H	-5.816538	2.211145	2.223065
H	-7.141207	3.007129	-1.787190
C	-5.657970	4.084548	0.311029
C	-4.989254	4.574182	-0.822166
C	-5.361414	4.699406	1.540659
C	-3.942934	5.480833	-0.707437
H	-5.197254	4.136157	-1.792193
C	-4.321043	5.612914	1.655272
H	-5.912340	4.412992	2.431547
C	-3.522586	5.952614	0.547664
H	-3.357202	5.711987	-1.590935
H	-4.086937	6.023532	2.632752
C	-2.167575	6.527317	0.701173
C	-1.428115	6.248800	1.863292
C	-1.474536	7.119653	-0.369893
C	-0.046813	6.388488	1.890968
H	-1.916187	5.776702	2.708720
C	-0.091598	7.255286	-0.344268
H	-2.017645	7.425944	-1.259086
C	0.663488	6.809012	0.755232
H	0.495757	6.020040	2.755006
H	0.416217	7.661532	-1.214215
C	2.108611	6.507586	0.652890
C	2.621834	6.043180	-0.569648
C	2.940868	6.402849	1.780852
C	3.827533	5.358630	-0.629636
H	2.003712	6.088909	-1.459666
C	4.147841	5.714602	1.721180
H	2.611209	6.812960	2.731006
C	4.577139	5.103478	0.530518
H	4.116691	4.892412	-1.565276
C	5.579655	4.015564	0.511131
C	5.652900	3.131105	1.599526
C	6.281275	3.659619	-0.654661
C	6.247934	1.882146	1.473517
H	5.109399	3.359524	2.510015
C	6.879829	2.410752	-0.780097
H	6.311711	4.348648	-1.493798
C	6.801753	1.460710	0.253800
H	6.145928	1.173596	2.288296
H	7.365657	2.147652	-1.715334
C	6.995667	0.014451	0.012824
C	1.708383	-0.880660	-3.018320
C	2.657500	0.295276	-2.818855
C	0.394738	-0.730759	-3.412994
C	1.934909	1.732646	-2.854635
C	1.997658	2.497909	-1.537685
C	3.276276	-0.067892	-1.469471
C	3.439565	0.819330	-0.424748
C	2.786768	2.142492	-0.467804
C	-0.252834	-2.766317	-2.160752
C	1.114101	-2.900577	-1.717644
C	-0.606936	-1.681428	-2.980662
C	2.054975	-1.952621	-2.123431
C	3.028866	-1.440756	-1.171043
C	1.090311	-3.362159	-0.334346
C	2.013110	-2.858679	0.584040
C	3.008560	-1.886680	0.158457
C	-2.679027	-1.346593	-1.742567

H	-6.017514	1.800772	2.326167
H	-7.369439	2.357307	-1.713677
C	-5.990834	3.617119	0.352130
C	-5.360383	4.120602	-0.796835
C	-5.747561	4.293292	1.561143
C	-4.390730	5.111644	-0.712278
H	-5.526500	3.632616	-1.750927
C	-4.782241	5.288955	1.645816
H	-6.277292	3.993934	2.460738
C	-4.011436	5.654179	0.526925
H	-3.821714	5.359386	-1.602153
H	-4.580967	5.747899	2.609158
C	-2.703269	6.332702	0.659455
C	-1.934089	6.119565	1.815827
C	-2.068038	6.965055	-0.424241
C	-0.567407	6.364177	1.828315
H	-2.376967	5.615980	2.668038
C	-0.699403	7.206992	-0.413213
H	-2.641944	7.218774	-1.310775
C	0.097375	6.830050	0.682776
H	0.010319	6.043154	2.688406
H	-0.231892	7.642549	-1.291540
C	1.560789	6.641940	0.570904
C	2.100243	6.207036	-0.651105
C	2.406055	6.613672	1.693623
C	3.355225	5.617771	-0.713552
H	1.474269	6.195429	-1.536724
C	3.661987	6.019955	1.631782
H	2.051622	7.006441	2.642193
C	4.129246	5.432296	0.443710
H	3.674004	5.166669	-1.646991
C	5.211863	4.424169	0.428230
C	5.353515	3.554919	1.522119
C	5.937728	4.116416	-0.736264
C	6.044443	2.355686	1.403637
H	4.792775	3.745410	2.430871
C	6.632224	2.917412	-0.854171
H	5.912758	4.799956	-1.580099
C	6.629943	1.971574	0.186409
H	5.997487	1.645945	2.222398
H	7.136045	2.686496	-1.788415
C	6.937357	0.543363	-0.043828
C	1.736113	-0.782937	-3.040795
C	2.588174	0.466360	-2.848386
C	0.413509	-0.740103	-3.434354
C	1.753947	1.841250	-2.891544
C	1.755393	2.617398	-1.579000
C	3.234867	0.161101	-1.497724
C	3.324992	1.063578	-0.457059
C	2.570290	2.330944	-0.506532
C	-0.067070	-2.815341	-2.171747
C	1.307738	-2.838217	-1.730562
C	-0.508055	-1.764759	-2.995554
C	2.168663	-1.818675	-2.142162
C	3.099551	-1.225677	-1.192691
C	1.323276	-3.291730	-0.345201
C	2.203153	-2.711578	0.570312
C	3.118052	-1.665694	0.138745
C	-2.595230	-1.586481	-1.748181

C	-2.305889	-2.434456	-0.860540	C	-2.135552	-2.636952	-0.861293
C	-1.850332	-0.986253	-2.782451	C	-1.801214	-1.168812	-2.794531
C	-1.123197	-3.149443	-1.070115	C	-0.901366	-3.259707	-1.077244
C	-0.287455	-3.518755	0.061072	C	-0.038341	-3.549187	0.052106
C	-2.713992	-2.086883	0.488063	C	-2.568880	-2.322452	0.485035
C	-1.909553	-2.438949	1.573723	C	-1.740288	-2.592868	1.564233
C	-0.670824	-3.168307	1.362752	C	-0.449208	-3.209774	1.349293
C	-2.501515	1.549787	-2.465359	C	-2.653257	1.309536	-2.496447
C	-3.167902	1.029257	-1.191905	C	-3.273820	0.746233	-1.218761
C	-1.764683	0.422269	-3.350906	C	-1.830318	0.237914	-3.374181
C	-3.348284	-0.310288	-0.919494	C	-3.342120	-0.602630	-0.931432
C	-3.357014	-0.782500	0.448497	C	-3.315517	-1.063116	0.438011
C	-2.935512	1.940423	-0.104617	C	-3.110285	1.674046	-0.142072
C	-2.965784	1.511430	1.226576	C	-3.092432	1.239651	1.189119
C	-3.180810	0.111431	1.510048	C	-3.209671	-0.158474	1.487811
C	0.428054	1.697536	-3.100829	C	0.254487	1.685548	-3.136815
C	-0.228494	2.705789	-2.251538	C	-0.478429	2.645842	-2.294320
C	-0.249123	0.592536	-3.484310	C	-0.333447	0.527952	-3.512778
C	-1.552651	2.589971	-1.880016	C	-1.789861	2.428235	-1.920949
C	-1.934428	2.904642	-0.531528	C	-2.192545	2.718043	-0.573466
C	0.753022	3.186835	-1.307256	C	0.462079	3.209226	-1.354065
C	0.367466	3.555225	-0.010190	C	0.048001	3.553391	-0.057273
C	-1.010995	3.407637	0.388344	C	-1.313229	3.299320	0.342369
C	-1.606009	0.919782	3.050196	C	-1.709761	0.782803	3.021653
C	-1.987693	2.020833	2.179697	C	-2.159971	1.830663	2.137331
C	-1.033503	2.949777	1.768934	C	-1.296193	2.848069	1.727706
C	-0.273010	0.782360	3.461642	C	-0.383363	0.755009	3.433848
C	-0.344423	-1.627377	3.129960	C	-0.246214	-1.657434	3.113940
C	-1.711220	-1.501651	2.666380	C	-1.681280	-1.717126	2.753159
C	-2.342893	-0.255442	2.639737	C	-2.451076	-0.467391	2.726745
C	0.357879	-0.512276	3.531066	C	0.357470	-0.503603	3.513514
C	2.586346	-1.495438	2.511362	C	2.666008	-1.298288	2.490845
C	1.629984	-2.540931	1.932595	C	1.799124	-2.418197	1.917535
C	0.309568	-2.676526	2.308288	C	0.488547	-2.639643	2.288812
C	1.874540	-0.358161	3.402444	C	1.855167	-0.221026	3.374616
C	2.769262	1.398735	1.766487	C	2.606275	1.594600	1.730628
C	3.425306	0.358728	0.944541	C	3.347383	0.609486	0.914187
C	3.247198	-0.980517	1.233487	C	3.283231	-0.738356	1.210048
C	1.964024	1.053418	2.830009	C	1.825806	1.187202	2.790922
C	0.347494	2.823190	2.212306	C	0.082451	2.829848	2.168976
C	1.206727	3.197166	1.111160	C	0.911366	3.265781	1.066497
C	2.386888	2.480786	0.883281	C	2.142657	2.640858	0.842132
C	0.719886	1.748825	3.031893	C	0.531706	1.785015	2.994823
H	4.738074	5.600096	2.625850	H	4.265128	5.959438	2.533172
C	-2.500719	0.587197	-4.751256	C	-2.583200	0.328740	-4.772587
C	-3.513609	2.169550	-3.521122	C	-3.716283	1.835037	-3.552270
C	-2.662266	2.116961	-4.803214	C	-2.870049	1.839406	-4.838718
H	-1.715172	2.659157	-4.724570	H	-1.970065	2.457911	-4.769883
H	-3.213567	2.451944	-5.686176	H	-3.450746	2.119269	-5.722101
C	-4.545284	1.098432	-3.814770	C	-4.658312	0.679534	-3.828591
H	-3.889523	3.141258	-3.202309	H	-4.169272	2.776256	-3.241099
C	-3.940421	0.152011	-4.546135	C	-3.981486	-0.221221	-4.554632
H	-5.537478	1.071953	-3.381570	H	-5.642155	0.573756	-3.387267
H	-1.952135	0.103524	-5.560871	H	-2.000885	-0.116751	-5.580577
H	-4.342455	-0.811023	-4.840367	H	-4.303698	-1.217688	-4.835376
H	2.093865	-0.033665	5.608036	H	2.046298	0.138049	5.577458
C	2.625971	-0.526343	4.793047	C	2.619058	-0.313111	4.765787
H	4.481488	0.854932	4.849482	H	4.350265	1.221790	4.812281
C	4.067138	-0.105820	4.565093	C	4.019411	0.226887	4.535978

C	2.773608	-2.057771	4.848905	C	2.896222	-1.825813	4.832793
H	1.820985	-2.592534	4.785736	H	1.991659	-2.438675	4.771364
H	3.334009	-2.394435	5.725423	H	3.481670	-2.108360	5.712203
C	4.652284	-1.060395	3.828679	C	4.684636	-0.679764	3.806742
C	3.605668	-2.123288	3.555095	C	3.732610	-1.829560	3.539986
H	5.637954	-1.043798	3.379785	H	5.665447	-0.581772	3.357162
H	3.968107	-3.099959	3.234964	H	4.177221	-2.774259	3.227303
C	2.747477	2.467813	-4.007966	C	2.505146	2.632293	-4.049521
C	2.406047	1.757619	-5.303851	C	2.221008	1.890274	-5.341414
C	3.005103	0.559654	-5.273268	C	2.913262	0.743804	-5.304672
C	4.165991	1.928371	-3.740724	C	3.962037	2.208702	-3.780302
H	2.600457	3.549006	-3.985798	H	2.272531	3.698461	-4.033044
C	3.752424	0.459895	-3.957137	C	3.666191	0.710904	-3.988449
H	4.536451	2.139269	-2.732803	H	4.314368	2.453552	-2.773636
H	4.890226	2.272735	-4.484447	H	4.656688	2.605489	-4.526203
H	1.697993	2.121411	-6.039570	H	1.485572	2.192256	-6.078264
H	2.886647	-0.254780	-5.978855	H	2.858980	-0.081658	-6.005312
H	4.528416	-0.304603	-3.890646	H	4.500392	0.010708	-3.918317
C	-3.869751	-3.207868	4.387790	C	-2.754064	-2.817329	4.292900
C	-3.356633	-2.517941	5.428329	C	-2.399446	-2.042333	5.416854
H	-2.578303	-2.857846	6.103138	H	-1.598114	-2.277299	6.108132
H	-3.590205	-4.203991	4.065489	H	-2.443767	-3.843913	4.132513
C	-4.003700	-1.203674	5.504628	C	-3.097038	-0.835492	5.373681
C	-4.909454	-1.094315	4.509932	C	-3.903461	-0.814344	4.210731
H	-5.545120	-0.242095	4.300261	H	-4.651421	-0.055163	4.008443
H	-3.767447	-0.446205	6.244286	H	-2.922675	0.012923	6.025456
H	-4.685429	-2.194606	2.647616	H	-4.266003	-2.419103	2.721189
C	-4.916420	-2.367714	3.707107	C	-4.062460	-2.258465	3.781156
H	-5.903629	-2.854707	3.721478	H	-4.887955	-2.707864	4.356807
C60CPP10_4_Prod				C60CPP10_5_Int			
204				215			
C60CPP10_4_Prod_3 SCF Done: -5375.07063626 A.U.				C60CPP10_5_Int_3 SCF Done: -5569.25270891 A.U.			
C	5.834444	-4.178361	-1.572784	C	-4.448620	-4.909170	1.199746
C	4.889175	-5.193844	-1.648488	C	-3.314709	-5.690147	1.026386
C	4.131903	-5.570410	-0.524138	C	-2.898938	-6.103418	-0.250296
C	4.506351	-5.015118	0.710896	C	-3.773120	-5.834470	-1.317095
C	5.455890	-4.004101	0.786455	C	-4.894418	-5.031985	-1.145221
C	6.070101	-3.492466	-0.367692	C	-5.214826	-4.469462	0.104469
H	6.353319	-3.871849	-2.476280	H	-4.678068	-4.563210	2.200868
H	4.692535	-5.660730	-2.608971	H	-2.679206	-5.878963	1.885173
H	3.947383	-5.271003	1.604832	H	-3.535014	-6.195649	-2.313022
H	5.616602	-3.508792	1.737693	H	-5.476474	-4.772635	-2.022789
C	2.837431	-6.276374	-0.647075	C	-1.501619	-6.532335	-0.467634
C	2.058530	-6.085345	-1.800804	C	-0.703107	-7.071533	0.557097
C	2.220586	-6.916326	0.442796	C	-0.847331	-6.160965	-1.653207
C	0.697214	-6.358391	-1.805431	C	0.684309	-7.069179	0.466718
H	2.486753	-5.577378	-2.657884	H	-1.170156	-7.448640	1.462137
C	0.857248	-7.186639	0.439676	C	0.536207	-6.154873	-1.739638
H	2.803841	-7.153678	1.327747	H	-1.415041	-5.723746	-2.466975
C	0.047663	-6.832044	-0.654362	C	1.344582	-6.519990	-0.648560
H	0.108910	-6.054344	-2.664538	H	1.260049	-7.450280	1.304558
H	0.403075	-7.627327	1.322435	H	0.984710	-5.718456	-2.623596
C	6.664037	-2.137655	-0.333565	C	-6.102857	-3.285810	0.207638
C	7.181526	-1.561738	0.841273	C	-6.830191	-2.791267	-0.892876
C	6.457280	-1.281949	-1.427534	C	-6.037982	-2.452357	1.337621
C	7.303421	-0.182207	0.970234	C	-7.266433	-1.471667	-0.937760
H	7.434835	-2.196865	1.685453	H	-6.998757	-3.419685	-1.761392
C	6.581964	0.094121	-1.299746	C	-6.478422	-1.138098	1.297032
H	6.048439	-1.682985	-2.348710	H	-5.510231	-2.777748	2.225352

H	7.651469	0.235428	1.911008
H	6.272068	0.724709	-2.125887
C	-1.418706	-6.673408	-0.536477
C	-1.961165	-6.242472	0.685601
C	-2.269507	-6.668789	-1.655345
C	-3.227456	-5.678196	0.750600
H	-1.331387	-6.213192	1.568111
C	-3.536916	-6.100285	-1.590960
H	-1.911581	-7.059890	-2.603291
C	-4.010425	-5.515334	-0.404034
H	-3.550913	-5.227854	1.682820
H	-4.145427	-6.057277	-2.489749
C	-5.113076	-4.529231	-0.389226
C	-5.275762	-3.668082	-1.486574
C	-5.841006	-4.230794	0.776393
C	-5.990357	-2.482511	-1.371201
H	-4.714483	-3.851687	-2.396425
C	-6.559252	-3.045551	0.891128
H	-5.799384	-4.909839	1.623200
C	-6.579658	-2.104771	-0.153831
H	-5.960707	-1.775943	-2.193484
H	-7.064784	-2.820635	1.825907
C	-6.916182	-0.682297	0.070803
C	-6.581909	-0.094350	1.300585
C	-7.303782	0.181993	-0.969266
C	-6.457316	1.281724	1.428393
H	-6.271817	-0.724950	2.126644
C	-7.182020	1.561533	-0.840269
H	-7.651921	-0.235647	-1.910004
C	-6.664351	2.137451	0.334494
H	-6.048330	1.682772	2.349501
H	-7.435572	2.196657	-1.684378
C	-6.070410	3.492270	0.368564
C	-5.456620	4.004005	-0.785777
C	-5.834294	4.178028	1.573633
C	-4.506943	5.014907	-0.710463
H	-5.618154	3.508958	-1.737013
C	-4.888956	5.193461	1.649077
H	-6.352864	3.871457	2.477284
C	-4.132021	5.570050	0.524507
H	-3.948334	5.270907	-1.604590
H	-4.691978	5.660300	2.609512
C	-2.837500	6.275991	0.647168
C	-2.058559	6.085266	1.800932
C	-2.220689	6.915699	-0.442857
C	-0.697265	6.358373	1.805484
H	-2.486743	5.577486	2.658139
C	-0.857352	7.186068	-0.439817
H	-2.803945	7.152828	-1.327866
C	-0.047739	6.831790	0.654295
H	-0.108943	6.054556	2.664658
H	-0.403217	7.626542	-1.322702
C	1.418645	6.673227	0.536479
C	1.961211	6.242198	-0.685510
C	2.269392	6.668778	1.655403
C	3.227537	5.677968	-0.750366
H	1.331511	6.212815	-1.568074
C	3.536817	6.100304	1.591168
H	1.911403	7.059999	2.603276
H	-7.758765	-1.105634	-1.834653
H	-6.270278	-0.493506	2.144024
C	2.756849	-6.076222	-0.599822
C	3.418487	-5.604376	-1.748013
C	3.402652	-5.855320	0.630240
C	4.532442	-4.782438	-1.653772
H	3.010039	-5.807875	-2.732094
C	4.537194	-5.059277	0.722094
H	2.960515	-6.224579	1.548743
C	5.069681	-4.415341	-0.407575
H	4.920560	-4.333245	-2.561844
H	4.960447	-4.867582	1.702780
C	5.936772	-3.221306	-0.297337
C	5.869665	-2.416020	0.852716
C	6.639520	-2.701049	-1.400071
C	6.313729	-1.100222	0.839979
H	5.331824	-2.766728	1.725880
C	7.077311	-1.381832	-1.416775
H	6.793690	-3.316354	-2.281250
C	6.843741	-0.526171	-0.326416
H	6.103221	-0.470580	1.698065
H	7.555080	-0.991935	-2.311156
C	6.848203	0.945472	-0.463651
C	6.364782	1.506327	-1.656007
C	7.052253	1.815401	0.621820
C	5.939065	2.825826	-1.707126
H	6.184591	0.863511	-2.511142
C	6.633902	3.140866	0.567249
H	7.496532	1.433028	1.536863
C	5.983215	3.650715	-0.571448
H	5.431892	3.173830	-2.600491
H	6.760819	3.771978	1.442242
C	5.120408	4.852113	-0.529611
C	4.453055	5.180845	0.660754
C	4.709285	5.520859	-1.696752
C	3.312652	5.973580	0.653655
H	4.745461	4.695410	1.584977
C	3.571848	6.318584	-1.704326
H	5.250844	5.365550	-2.625157
C	2.790438	6.484100	-0.546381
H	2.742279	6.073065	1.571209
H	3.250511	6.770993	-2.637752
C	1.373991	6.905955	-0.606502
C	0.612643	6.588012	-1.743815
C	0.676930	7.371115	0.522668
C	-0.774961	6.573127	-1.701123
H	1.107700	6.203624	-2.628834
C	-0.712080	7.352927	0.566801
H	1.229399	7.698427	1.398653
C	-1.467724	6.871136	-0.516881
H	-1.317902	6.177594	-2.552910
H	-1.217363	7.664076	1.476555
C	-2.866195	6.413186	-0.365942
C	-3.266251	5.836968	0.850967
C	-3.737324	6.277374	-1.460540
C	-4.389314	5.025071	0.926133
H	-2.612055	5.903991	1.713447
C	-4.860892	5.461777	-1.385939
H	-3.498966	6.767732	-2.399924

C	4.010424	5.515226	0.404334	C	-5.163479	4.749525	-0.212751
H	3.551082	5.227566	-1.682528	H	-4.581891	4.483957	1.846206
C	5.113089	4.529136	0.389712	C	-6.042866	3.560281	-0.211387
C	5.275695	3.668070	1.487136	C	-6.065737	2.726578	-1.341317
C	5.841111	4.230587	-0.775827	C	-6.653982	3.076685	0.959195
C	5.990277	2.482473	1.371906	C	-6.524078	1.418029	-1.260578
H	4.714348	3.851740	2.396932	H	-5.584380	3.055104	-2.256173
C	6.559339	3.045325	-0.890418	C	-7.113595	1.766767	1.040603
H	5.799556	4.909561	-1.622695	H	-6.719435	3.717400	1.833897
C	6.579644	2.104607	0.154605	C	-6.982505	0.883593	-0.045519
H	5.960541	1.775985	2.194253	H	-6.382174	0.762429	-2.113085
H	7.064937	2.820334	-1.825144	H	-7.527227	1.406423	1.978267
C	6.916081	0.682088	-0.069924	C	-7.007036	-0.584710	0.121512
C	1.729144	-0.766441	-3.043376	C	-1.515184	-0.984918	3.016257
C	2.556624	0.500850	-2.861073	C	-2.591132	0.086507	2.876690
C	0.402954	-0.751593	-3.429235	C	-0.208369	-0.697299	3.354890
C	1.694748	1.858574	-2.905381	C	-2.026410	1.593355	2.911619
C	1.687202	2.640536	-1.596214	C	-2.224734	2.368251	1.613475
C	3.217301	0.214124	-1.513157	C	-3.223067	-0.320480	1.546116
C	3.293883	1.122262	-0.476132	C	-3.522308	0.559261	0.525197
C	2.513531	2.374277	-0.526681	C	-3.015325	1.944944	0.567722
C	-0.026800	-2.830957	-2.156122	C	0.595733	-2.628266	2.035266
C	1.351098	-2.825207	-1.724052	C	-0.767369	-2.913910	1.653407
C	-0.494467	-1.791682	-2.979214	C	0.867401	-1.522558	2.855114
C	2.188540	-1.789406	-2.144798	C	-1.784513	-2.075035	2.116208
C	3.112594	-1.173761	-1.202299	C	-2.844195	-1.654652	1.212226
C	1.384069	-3.271862	-0.337320	C	-0.754919	-3.346573	0.262066
C	2.256036	-2.670225	0.571231	C	-1.763601	-2.931559	-0.610334
C	3.148364	-1.608571	0.130595	C	-2.836603	-2.078841	-0.125646
C	-2.571471	-1.644090	-1.711575	C	2.829671	-0.941605	1.528220
C	-2.084723	-2.678882	-0.822546	C	2.534155	-2.046650	0.639655
C	-1.797842	-1.219595	-2.770065	C	2.014751	-0.693407	2.611377
C	-0.843356	-3.282536	-1.050785	C	1.449186	-2.892413	0.899108
C	0.030165	-3.550526	0.069848	C	0.613320	-3.328457	-0.193560
C	-2.513451	-2.374131	0.526304	C	2.846417	-1.652124	-0.716901
C	-1.687117	-2.640370	1.595838	C	2.052721	-2.074557	-1.760724
C	-0.384565	-3.209106	1.367764	C	0.903894	-2.898113	-1.497547
C	-2.698727	1.242278	-2.483688	C	2.383428	1.900902	2.339852
C	-3.299489	0.674587	-1.198197	C	3.044739	1.481246	1.026298
C	-1.859425	0.182134	-3.359050	C	1.809012	0.682211	3.222274
C	-3.332395	-0.673354	-0.896644	C	3.344407	0.171433	0.703343
C	-3.293810	-1.122107	0.475742	C	3.352809	-0.265940	-0.673905
C	-3.148302	1.608718	-0.130965	C	2.666184	2.370966	-0.022096
C	-3.112524	1.173899	1.201938	C	2.675125	1.947787	-1.359976
C	-3.217206	-0.213977	1.512774	C	3.054776	0.614345	-1.694942
C	0.197450	1.672110	-3.141731	C	-0.515749	1.719542	3.097423
C	-0.549694	2.622816	-2.300754	C	-0.009254	2.809461	2.245578
C	-0.369622	0.501229	-3.508326	C	0.291794	0.687445	3.427404
C	-1.854990	2.382271	-1.920117	C	1.303969	2.847306	1.820226
C	-2.255963	2.670382	-0.571585	C	1.589350	3.221267	0.462723
C	0.384643	3.209268	-1.368124	C	-1.076293	3.195584	1.351442
C	-0.030089	3.550677	-0.070203	C	-0.786748	3.625172	0.046078
C	-1.383986	3.272004	0.336964	C	0.580869	3.635673	-0.408730
C	-1.729056	0.766581	3.043004	C	1.341690	1.275485	-3.160654
C	-2.188459	1.789539	2.144435	C	1.610827	2.364762	-2.262478
C	-1.351016	2.825340	1.723697	C	0.592597	3.202195	-1.800061
C	-0.402870	0.751751	3.428869	C	0.034215	0.989392	-3.502690
C	-0.197363	-1.671953	3.141362	C	0.343188	-1.426601	-3.244881
C	-1.694698	-1.858393	2.905012	C	1.854402	-1.300893	-3.058641

C	-2.556520	-0.500745	2.860708	C	2.419256	0.205541	-3.024258
C	0.369709	-0.501083	3.507960	C	-0.465665	-0.395543	-3.574983
C	2.698831	-1.242095	2.483317	C	-2.556355	-1.609490	-2.485197
C	1.855088	-2.382125	1.919780	C	-1.476627	-2.556412	-1.967466
C	0.549778	-2.622650	2.300396	C	-0.163248	-2.516736	-2.393152
C	1.859509	-0.181986	3.358703	C	-1.983260	-0.393869	-3.373508
C	2.571548	1.644236	1.711208	C	-3.000194	1.234948	-1.677821
C	3.332470	0.673503	0.896267	C	-3.515970	0.121940	-0.852082
C	3.299541	-0.674419	1.197817	C	-3.214622	-1.187241	-1.173228
C	1.797927	1.219743	2.769711	C	-2.192513	0.986803	-2.767046
C	0.026877	2.831098	2.155762	C	-0.771204	2.925424	-2.186721
C	0.843434	3.282677	1.050424	C	-1.624775	3.190794	-1.049373
C	2.084798	2.679030	0.822178	C	-2.706391	2.341519	-0.790008
C	0.494554	1.791824	2.978856	C	-1.042608	1.817596	-3.008699
H	4.145279	6.057439	2.489997	H	-5.480249	5.329339	-2.268427
C	-2.622211	0.247845	-4.753478	C	2.591481	0.879565	4.592325
C	-3.778359	1.738949	-3.536192	C	3.375132	2.592523	3.369232
C	-2.940275	1.751849	-4.827937	C	2.599693	2.415425	4.688149
H	-2.052713	2.389075	-4.768884	H	1.599356	2.858608	4.671207
H	-3.531979	2.013829	-5.709488	H	3.157419	2.781262	5.554799
C	-4.698179	0.562523	-3.798742	C	4.523808	1.623777	3.575884
H	-4.248788	2.672705	-3.228488	H	3.634461	3.607229	3.068523
C	-4.007580	-0.329164	-4.522979	C	4.055204	0.600187	4.303518
H	-5.676821	0.440083	-3.350356	H	5.489391	1.706660	3.091300
H	-2.035743	-0.191294	-5.561952	H	2.135534	0.313956	5.406100
H	-4.311117	-1.333823	-4.795230	H	4.565591	-0.326512	4.540901
H	2.035728	0.191255	5.561648	H	-2.302621	-0.061124	-5.567068
C	2.622268	-0.247736	4.753148	C	-2.761429	-0.611400	-4.744143
H	4.310959	1.334158	4.795025	H	-4.735389	0.595212	-4.717516
C	4.007553	0.329484	4.522691	C	-4.226166	-0.328125	-4.464829
C	2.940537	-1.751716	4.827518	C	-2.770823	-2.149271	-4.821843
H	2.053055	-2.389050	4.768422	H	-1.770885	-2.593320	-4.797306
H	3.532265	-2.013655	5.709064	H	-3.325244	-2.522408	-5.687552
C	4.698245	-0.562056	3.798371	C	-4.696787	-1.342407	-3.725865
C	3.778606	-1.738604	3.535761	C	-3.551091	-2.310854	-3.503926
H	5.676865	-0.439476	3.349987	H	-5.663283	-1.420207	-3.243500
H	4.249333	-2.672156	3.227881	H	-3.814237	-3.318043	-3.181089
C	2.423848	2.659628	-4.070600	C	-2.865071	2.218731	4.109939
C	2.147672	1.906759	-5.357948	C	-2.395701	1.530161	5.377288
C	2.862897	0.774512	-5.320387	C	-2.863355	0.275055	5.347132
C	3.890283	2.266298	-3.807567	C	-4.227331	1.533380	3.888965
H	2.169977	3.720964	-4.057141	H	-2.835735	3.309706	4.103160
C	3.623292	0.762036	-4.008142	C	-3.649599	0.115249	4.059898
H	4.243088	2.522303	-2.803824	H	-4.659905	1.717847	2.900760
H	4.572848	2.673705	-4.558851	H	-4.952843	1.786090	4.667476
H	1.402587	2.191125	-6.092099	H	-1.699615	1.956745	6.090374
H	2.821610	-0.054649	-6.017534	H	-2.627104	-0.532917	6.030229
H	4.471609	0.078869	-3.939541	H	-4.341462	-0.727495	4.009535
C	-2.423784	-2.659495	4.070215	C	2.693932	-1.928956	-4.254869
C	-2.147600	-1.906619	5.357553	C	2.224019	-1.244303	-5.523906
H	-1.402497	-2.190918	6.091713	H	1.528670	-1.672978	-6.236525
H	-2.169940	-3.720836	4.056746	H	2.667006	-3.019974	-4.242265
C	-2.862828	-0.774371	5.319993	C	2.689549	0.011763	-5.496022
C	-3.623235	-0.761902	4.007761	C	3.475887	0.175805	-4.209387
H	-4.471564	-0.078755	3.939145	H	4.166410	1.019518	-4.160741
H	-2.821495	0.054765	6.017167	H	2.452658	0.817612	-6.181420
H	-4.242996	-2.522158	2.803435	H	4.487996	-1.424366	-3.047054
C	-3.890209	-2.266161	3.807189	C	4.054974	-1.241577	-4.034756
H	-4.572801	-2.673588	4.558450	H	4.780968	-1.494813	-4.812852

				C	1.510820	-4.880919	3.964360
				C	2.827874	-4.151430	3.992568
				H	3.033900	-3.646844	3.039510
				H	3.672570	-4.836645	4.165867
				C	2.657547	-3.179492	5.129721
				C	1.434132	-3.348037	5.675264
				H	1.020901	-2.791908	6.510192
				C	0.721825	-4.406485	4.951130
				H	3.419945	-2.477106	5.445150
				H	1.253634	-5.639368	3.235001
				H	-0.291145	-4.725243	5.172342
C60CPP10_5_TS				C60CPP10_5_Prod			
215				215			
C60CPP10_5_TS_3 SCF Done: -5569.22303644 A.U.				C60CPP10_5_Prod_3 SCF Done: -5569.25820410 A.U.			
C	-4.880879	-4.452939	1.231021	C	-5.027725	-4.276466	1.243185
C	-3.827435	-5.344476	1.086225	C	-4.009169	-5.207824	1.100136
C	-3.436497	-5.815748	-0.177804	C	-3.639194	-5.698518	-0.162729
C	-4.263530	-5.477587	-1.262352	C	-4.454318	-5.333535	-1.247572
C	-5.302479	-4.566459	-1.119248	C	-5.457615	-4.383062	-1.106298
C	-5.583805	-3.956783	0.117675	C	-5.713380	-3.758816	0.128960
H	-5.089469	-4.070325	2.223265	H	-5.219301	-3.881900	2.234144
H	-3.226528	-5.584114	1.957411	H	-3.415157	-5.465717	1.970808
H	-4.046658	-5.874642	-2.249414	H	-4.253621	-5.742363	-2.233300
H	-5.842618	-4.263155	-2.009393	H	-5.987820	-4.062819	-1.996499
C	-2.085593	-6.383129	-0.367544	C	-2.310633	-6.316435	-0.351391
C	-1.361002	-6.994082	0.671643	C	-1.612539	-6.957421	0.687587
C	-1.378710	-6.083742	-1.543081	C	-1.589935	-6.038196	-1.523795
C	0.021809	-7.130081	0.603566	C	-0.235516	-7.143860	0.623174
H	-1.877987	-7.320006	1.569554	H	-2.144239	-7.266424	1.582903
C	-0.000274	-6.213568	-1.606517	C	-0.217008	-6.218592	-1.583673
H	-1.886731	-5.595001	-2.366816	H	-2.077496	-5.527329	-2.346521
C	0.751155	-6.652187	-0.501794	C	0.514019	-6.689012	-0.478198
H	0.543553	-7.564719	1.450941	H	0.267750	-7.598662	1.471005
H	0.501599	-5.824937	-2.483666	H	0.301979	-5.845520	-2.457629
C	-6.354416	-2.691663	0.191453	C	-6.435843	-2.465547	0.199236
C	-7.016676	-2.146593	-0.926047	C	-7.079831	-1.900635	-0.919026
C	-6.224328	-1.851773	1.310906	C	-6.272372	-1.626968	1.315353
C	-7.326257	-0.792718	-0.994144	C	-7.339419	-0.536571	-0.991594
H	-7.232298	-2.768633	-1.788724	H	-7.320061	-2.517567	-1.778860
C	-6.538561	-0.502746	1.247048	C	-6.537020	-0.267603	1.247119
H	-5.739090	-2.211896	2.209407	H	-5.799317	-2.001555	2.214444
H	-7.769891	-0.394674	-1.902713	H	-7.769929	-0.125785	-1.900798
H	-6.280337	0.131684	2.087810	H	-6.254378	0.359941	2.085168
C	2.198676	-6.345110	-0.433240	C	1.971256	-6.433787	-0.403665
C	2.912378	-5.934945	-1.573624	C	2.703323	-6.048756	-1.541108
C	2.850283	-6.185352	0.803630	C	2.621704	-6.295437	0.836370
C	4.094085	-5.217014	-1.466770	C	3.907215	-5.369630	-1.428770
H	2.497814	-6.099285	-2.562196	H	2.287990	-6.200063	-2.531470
C	4.048960	-5.490459	0.909217	C	3.841758	-5.639776	0.947602
H	2.370569	-6.521311	1.716461	H	2.125797	-6.612814	1.747100
C	4.648620	-4.898265	-0.215160	C	4.465081	-5.068191	-0.174343
H	4.529880	-4.803688	-2.370042	H	4.360888	-4.970869	-2.329821
H	4.478408	-5.337099	1.894461	H	4.270379	-5.498320	1.934967
C	5.621786	-3.789552	-0.103504	C	5.471479	-3.990078	-0.059463
C	5.611928	-2.964936	1.034140	C	5.474853	-3.156957	1.072219
C	6.387391	-3.354068	-1.201157	C	6.262684	-3.588118	-1.151468
C	6.178022	-1.696699	1.011765	C	6.084079	-1.908693	1.048113
H	5.030273	-3.252623	1.902433	H	4.874630	-3.420150	1.935887
C	6.948033	-2.082669	-1.227603	C	6.865605	-2.336254	-1.180102

H	6.497068	-3.994529	-2.070971	H	6.360533	-4.238468	-2.015353
C	6.778736	-1.192902	-0.152740	C	6.715333	-1.434300	-0.112791
H	6.013262	-1.037208	1.857210	H	5.933563	-1.238229	1.887629
H	7.475078	-1.752586	-2.118334	H	7.413543	-2.030121	-2.066781
C	6.926845	0.269138	-0.310826	C	6.918780	0.020086	-0.279256
C	6.515369	0.854056	-1.518470	C	6.539233	0.611393	-1.494239
C	7.201034	1.133061	0.763770	C	7.219304	0.880745	0.790736
C	6.221585	2.207701	-1.596275	C	6.299709	1.974889	-1.584314
H	6.283103	0.217744	-2.365824	H	6.288647	-0.021927	-2.338627
C	6.914498	2.491916	0.682771	C	6.988123	2.249347	0.697201
H	7.594883	0.724554	1.690586	H	7.590394	0.464039	1.723249
C	6.331574	3.043405	-0.473075	C	6.435130	2.814505	-0.466733
H	5.761796	2.587931	-2.501960	H	5.861673	2.365580	-2.496329
H	7.090865	3.122326	1.549682	H	7.183871	2.878773	1.560621
C	5.590174	4.323981	-0.463657	C	5.743729	4.122872	-0.471108
C	4.940844	4.736847	0.710318	C	5.109375	4.571873	0.697839
C	5.262892	5.008717	-1.647847	C	5.443642	4.807889	-1.662360
C	3.882612	5.635571	0.671905	C	4.085097	5.508484	0.649094
H	5.171650	4.242116	1.646953	H	5.321422	4.079323	1.639959
C	4.209075	5.913137	-1.686600	C	4.423789	5.750005	-1.711595
H	5.800034	4.785060	-2.564790	H	5.973127	4.555724	-2.576335
C	3.430339	6.173592	-0.544418	C	3.653911	6.050209	-0.573202
H	3.310565	5.804932	1.578096	H	3.519356	5.708474	1.552991
H	3.947052	6.378156	-2.632272	H	4.180074	6.214934	-2.662198
C	2.062848	6.730099	-0.634331	C	2.307555	6.654893	-0.670100
C	1.291804	6.469789	-1.779643	C	1.528034	6.410103	-1.813059
C	1.397086	7.277923	0.476523	C	1.661730	7.237710	0.434675
C	-0.091337	6.588103	-1.759288	C	0.149937	6.577388	-1.794514
H	1.760764	6.026113	-2.650985	H	1.981254	5.940983	-2.679411
C	0.012236	7.393188	0.498349	C	0.281809	7.401836	0.454846
H	1.965315	7.564167	1.356810	H	2.239754	7.512874	1.312110
C	-0.769710	6.968369	-0.590480	C	-0.514394	6.993366	-0.629821
H	-0.657115	6.232689	-2.613876	H	-0.428287	6.233229	-2.645394
H	-0.474601	7.764902	1.395357	H	-0.191913	7.799527	1.347743
C	-2.207073	6.646253	-0.455157	C	-1.961825	6.722075	-0.491445
C	-2.675517	6.125183	0.762186	C	-2.445662	6.223303	0.729161
C	-3.072249	6.579492	-1.560746	C	-2.830128	6.679030	-1.595716
C	-3.871400	5.424263	0.830392	C	-3.664286	5.563260	0.802387
H	-2.028931	6.140270	1.632838	H	-1.797516	6.220912	1.598790
C	-4.269013	5.874981	-1.493062	C	-4.050127	6.016191	-1.522771
H	-2.775603	7.032749	-2.502181	H	-2.519340	7.117099	-2.539798
C	-4.653380	5.209765	-0.316256	C	-4.454857	5.370077	-0.342121
H	-4.126474	4.914917	1.753392	H	-3.934766	5.067015	1.728130
C	-5.642302	4.110004	-0.313598	C	-5.481063	4.305248	-0.332766
C	-5.728585	3.266677	-1.433185	C	-5.599793	3.461397	-1.448941
C	-6.313423	3.703650	0.853466	C	-6.162256	3.926678	0.837792
C	-6.310255	2.008781	-1.342774	C	-6.225108	2.225098	-1.352409
H	-5.204441	3.534569	-2.344275	H	-5.069156	3.707454	-2.362441
C	-6.896383	2.444498	0.944588	C	-6.788785	2.689211	0.935118
H	-6.329595	4.360035	1.718792	H	-6.152932	4.586345	1.700705
C	-6.834046	1.537662	-0.128050	C	-6.761815	1.777156	-0.134612
H	-6.218006	1.330190	-2.183973	H	-6.159347	1.540646	-2.191373
H	-7.355213	2.138055	1.880401	H	-7.255505	2.402370	1.873231
C	-6.998706	0.080745	0.057399	C	-6.978218	0.327887	0.056264
C	-1.598771	-0.844118	3.006370	C	-1.594829	-0.787651	2.997985
C	-2.567356	0.323466	2.843641	C	-2.528905	0.405854	2.832687
C	-0.282386	-0.680430	3.340639	C	-0.295331	-0.668107	3.364812
C	-1.871359	1.776905	2.879242	C	-1.800018	1.839345	2.872544
C	-1.983412	2.563657	1.577461	C	-1.889762	2.630607	1.571760

C	-3.215202	-0.026219	1.505269	C	-3.190397	0.076080	1.500310
C	-3.425687	0.873853	0.480542	C	-3.380066	0.981263	0.477097
C	-2.795239	2.207716	0.523357	C	-2.710629	2.296305	0.517571
C	0.363182	-2.820794	2.144802	C	0.270420	-2.979527	2.215200
C	-1.037591	-2.837361	1.642478	C	-1.136161	-2.828415	1.652538
C	0.741457	-1.657245	2.959454	C	0.735772	-1.773462	3.180073
C	-1.966994	-1.918192	2.091469	C	-2.015439	-1.866548	2.092455
C	-2.963198	-1.392694	1.178108	C	-2.986923	-1.299567	1.178678
C	-1.043253	-3.246893	0.260072	C	-1.150199	-3.217711	0.268219
C	-2.005913	-2.764377	-0.631746	C	-2.092734	-2.704103	-0.629173
C	-2.992532	-1.817919	-0.161344	C	-3.041602	-1.724423	-0.160600
C	2.727298	-1.210673	1.565565	C	2.648378	-1.290548	1.588112
C	2.333245	-2.295330	0.670301	C	2.235639	-2.382791	0.684751
C	1.929296	-0.888410	2.629485	C	1.918548	-0.958501	2.674796
C	1.183255	-3.032957	0.920565	C	1.079484	-3.091909	0.927511
C	0.324856	-3.362601	-0.176868	C	0.215043	-3.378840	-0.167776
C	2.696948	-1.932517	-0.680541	C	2.616536	-2.028195	-0.660466
C	1.886941	-2.283624	-1.741014	C	1.810058	-2.359773	-1.731873
C	0.665930	-2.983873	-1.486531	C	0.568512	-3.013896	-1.480605
C	2.549066	1.661922	2.352590	C	2.606029	1.579498	2.356315
C	3.182797	1.176892	1.050565	C	3.215005	1.067894	1.057035
C	1.846567	0.507184	3.225369	C	1.870612	0.451748	3.242670
C	3.352009	-0.159848	0.739742	C	3.321300	-0.273750	0.753292
C	3.334220	-0.603263	-0.635251	C	3.296354	-0.722422	-0.616712
C	2.904103	2.092768	-0.003982	C	2.978776	1.991445	-0.001106
C	2.886013	1.663132	-1.341153	C	2.944889	1.554667	-1.336066
C	3.137136	0.297124	-1.664467	C	3.146802	0.178456	-1.653247
C	-0.360281	1.761242	3.076735	C	-0.292692	1.775283	3.070288
C	0.257756	2.796548	2.230802	C	0.356716	2.788147	2.225867
C	0.338714	0.651140	3.401377	C	0.366255	0.636871	3.393803
C	1.573120	2.708396	1.819978	C	1.668952	2.658713	1.818909
C	1.908345	3.045408	0.464929	C	2.016946	2.980051	0.463579
C	-0.758573	3.278044	1.323858	C	-0.642272	3.303674	1.316915
C	-0.414865	3.670726	0.020153	C	-0.284321	3.680592	0.012602
C	0.953008	3.548919	-0.419707	C	1.079732	3.511668	-0.425154
C	1.515800	1.114027	-3.155912	C	1.562182	1.047142	-3.152535
C	1.878218	2.176155	-2.256320	C	1.960063	2.099740	-2.255065
C	0.938809	3.108805	-1.808120	C	1.052081	3.065631	-1.810492
C	0.191716	0.952866	-3.512550	C	0.234782	0.931080	-3.511845
C	0.264365	-1.480949	-3.247895	C	0.222688	-1.504114	-3.245712
C	1.779375	-1.499219	-3.043471	C	1.735976	-1.574007	-3.036156
C	2.483914	-0.053301	-3.002134	C	2.488677	-0.152410	-2.993454
C	-0.438502	-0.378367	-3.590122	C	-0.441131	-0.378098	-3.589207
C	-2.647956	-1.384637	-2.520004	C	-2.685671	-1.306804	-2.520055
C	-1.668169	-2.428367	-1.990086	C	-1.742937	-2.383317	-1.988627
C	-0.354713	-2.515531	-2.400079	C	-0.434050	-2.516029	-2.397807
C	-1.951540	-0.230934	-3.405138	C	-1.948553	-0.178482	-3.406657
C	-2.823752	1.490766	-1.720044	C	-2.761650	1.574484	-1.723881
C	-3.450106	0.434701	-0.897723	C	-3.421851	0.541518	-0.900534
C	-3.275940	-0.896844	-1.214987	C	-3.295753	-0.795685	-1.215606
C	-2.033346	1.164637	-2.802154	C	-1.982558	1.220190	-2.805801
C	-0.440696	2.961126	-2.209743	C	-0.331150	2.963645	-2.214735
C	-1.277714	3.311298	-1.083027	C	-1.157358	3.345524	-1.090430
C	-2.436585	2.568083	-0.832378	C	-2.340183	2.641237	-0.838702
C	-0.807170	1.881210	-3.031697	C	-0.732649	1.894190	-3.033957
H	-4.886383	5.790372	-2.382806	H	-4.671598	5.948437	-2.411093
C	2.614588	0.646621	4.613454	C	2.631985	0.607005	4.636631
C	3.587245	2.260249	3.393762	C	3.665615	2.157656	3.386497
C	2.771108	2.174621	4.696133	C	2.840286	2.129516	4.684586

H	1.819545	2.712808	4.653768	H	1.908292	2.699221	4.625181
H	3.343248	2.491916	5.572525	H	3.418362	2.445714	5.557479
C	4.629950	1.185519	3.633579	C	4.668074	1.052157	3.654333
H	3.951892	3.240787	3.089597	H	4.066095	3.117654	3.062846
C	4.049438	0.221265	4.361935	C	4.052141	0.126158	4.403000
H	5.608426	1.167722	3.168929	H	5.647466	0.990356	3.195531
H	2.085016	0.148725	5.426643	H	2.084498	0.153393	5.463198
H	4.463064	-0.747750	4.618816	H	4.429916	-0.850563	4.684444
H	-2.217554	0.124940	-5.601587	H	-2.201142	0.182281	-5.603809
C	-2.734244	-0.376897	-4.781979	C	-2.735244	-0.299825	-4.783509
H	-4.584617	1.012393	-4.777009	H	-4.536346	1.152887	-4.782192
C	-4.168150	0.045328	-4.517957	C	-4.153817	0.172442	-4.521084
C	-2.889620	-1.907144	-4.857766	C	-2.943904	-1.823876	-4.857002
H	-1.936822	-2.444241	-4.821797	H	-2.010519	-2.393955	-4.819652
H	-3.468310	-2.227799	-5.728673	H	-3.532912	-2.125360	-5.727820
C	-4.741005	-0.918015	-3.783035	C	-4.760322	-0.769103	-3.784894
C	-3.694797	-1.990348	-3.547601	C	-3.752302	-1.876923	-3.547262
H	-5.715804	-0.902210	-3.311311	H	-5.734316	-0.718150	-3.313901
H	-4.055885	-2.967593	-3.226487	H	-4.147195	-2.840518	-3.224596
C	-2.661933	2.477138	4.067488	C	-2.572065	2.555539	4.063686
C	-2.269851	1.752074	5.340969	C	-2.193357	1.820094	5.335265
C	-2.849863	0.544862	5.308515	C	-2.799617	0.625771	5.302230
C	-4.078359	1.917333	3.833271	C	-4.000856	2.027417	3.832338
H	-2.534536	3.561063	4.060108	H	-2.420297	3.636292	4.057354
C	-3.633410	0.453371	4.012821	C	-3.587456	0.553647	4.008199
H	-4.481262	2.137598	2.839862	H	-4.401646	2.258608	2.840506
H	-4.786317	2.237314	4.603242	H	-4.699325	2.360876	4.605172
H	-1.545079	2.115722	6.060423	H	-1.459879	2.166955	6.054195
H	-2.695179	-0.279195	5.995891	H	-2.662731	-0.202079	5.988726
H	-4.398532	-0.323163	3.956613	H	-4.369110	-0.206146	3.951677
C	2.570699	-2.205515	-4.227619	C	2.507712	-2.307666	-4.215878
C	2.187736	-1.480836	-5.503669	C	2.155910	-1.572201	-5.494747
H	1.464532	-1.841462	-6.226195	H	1.424601	-1.909137	-6.220552
H	2.438651	-3.288907	-4.216185	H	2.338869	-3.385907	-4.203813
C	2.771842	-0.275575	-5.469362	C	2.780656	-0.387554	-5.459254
C	3.550831	-0.185898	-4.170980	C	3.555851	-0.322557	-4.157158
H	4.318376	0.587760	-4.111846	H	4.348916	0.424693	-4.095092
H	2.623318	0.548521	-6.157911	H	2.663807	0.440083	-6.149661
H	4.387304	-1.874168	-2.993403	H	4.328531	-2.036626	-2.973119
C	3.988662	-1.652161	-3.987946	C	3.942621	-1.802628	-3.970065
H	4.698255	-1.975007	-4.755360	H	4.644661	-2.150559	-4.733423
C	0.630152	-4.392181	3.316798	C	0.440017	-4.243367	3.159265
C	1.934643	-3.895588	3.915564	C	1.710101	-3.849277	3.931850
H	2.669978	-3.530313	3.196273	H	2.570198	-3.653321	3.284054
H	2.394596	-4.720704	4.483712	H	1.978498	-4.576347	4.703669
C	1.403818	-2.869547	4.880088	C	1.077782	-2.572678	4.518364
C	0.111869	-3.261088	5.241159	C	-0.232393	-3.116011	5.053622
H	-0.483269	-2.805325	6.024772	H	-0.799823	-2.672234	5.863769
C	-0.349811	-4.216020	4.329739	C	-0.611336	-4.114371	4.244138
H	-1.360428	-4.605351	4.288342	H	-1.552793	-4.652113	4.257546
H	0.596292	-5.212620	2.608534	H	0.445818	-5.172206	2.590080
H	2.031206	-2.185130	5.437169	H	1.680835	-1.988772	5.213136
**C60CPP10_6_Int**				**C60CPP10_6_TS**			
226				226			
C60CPP10_6_Int_3 SCF Done: -5763.44083016 A.U.				C60CPP10_6_TS_3 SCF Done: -5763.41040820 A.U.			
C	-2.055363	6.062661	-1.434302	C	-3.258230	-5.588236	1.236570
C	-0.725258	6.416218	-1.266671	C	-2.015340	-6.184191	1.085499
C	-0.250579	6.894580	-0.035743	C	-1.605317	-6.704661	-0.151476
C	-1.203544	7.163174	0.960616	C	-2.564485	-6.756893	-1.176133

C	-2.540844	6.824503	0.784249	C	-3.816496	-6.173323	-1.017300
C	-2.985107	6.180097	-0.386134	C	-4.162590	-5.489706	0.164109
H	-2.332737	5.560955	-2.353064	H	-3.460098	-5.075307	2.168557
H	-0.017987	6.175891	-2.053196	H	-1.298083	-6.108502	1.895877
H	-0.884344	7.595326	1.905005	H	-2.307324	-7.212854	-2.128209
H	-3.234822	7.014074	1.596914	H	-4.508207	-6.200801	-1.852946
C	1.198213	6.812365	0.238467	C	-0.160923	-6.888835	-0.396527
C	2.167210	6.997933	-0.761616	C	0.732657	-7.288069	0.611195
C	1.627754	6.253767	1.452005	C	0.392513	-6.383804	-1.583191
C	3.452914	6.488606	-0.618287	C	2.095531	-7.035386	0.502532
H	1.892922	7.495363	-1.687738	H	0.348069	-7.750909	1.515788
C	2.906007	5.731429	1.586430	C	1.750102	-6.116570	-1.682375
H	0.907470	6.082130	2.245244	H	-0.265163	-6.048777	-2.378470
C	3.824111	5.748601	0.520630	C	2.626017	-6.349936	-0.606939
H	4.154494	6.615272	-1.436700	H	2.743675	-7.321535	1.324865
H	3.130959	5.177386	2.489257	H	2.097494	-5.585457	-2.559953
C	-4.254068	5.417153	-0.437784	C	-5.260036	-4.494325	0.207346
C	-4.917378	5.040400	0.743979	C	-5.839728	-4.003590	-0.977139
C	-4.704985	4.818369	-1.628077	C	-5.583753	-3.809067	1.392369
C	-5.833652	3.997844	0.759249	C	-6.529310	-2.799189	-1.001376
H	-4.643387	5.497473	1.688564	H	-5.669476	-4.515305	-1.917957
C	-5.595130	3.753318	-1.609028	C	-6.245050	-2.588949	1.364741
H	-4.287229	5.122584	-2.581602	H	-5.231042	-4.178290	2.348847
H	-6.277369	3.700256	1.704315	H	-6.908024	-2.426878	-1.948449
H	-5.801245	3.230123	-2.537302	H	-6.339805	-2.027013	2.288324
C	4.966020	4.803857	0.509130	C	3.932686	-5.653757	-0.553239
C	5.329611	4.080274	1.658781	C	4.470792	-5.011632	-1.682712
C	5.552598	4.390966	-0.701872	C	4.549702	-5.375445	0.680390
C	6.052329	2.899305	1.570988	C	5.412312	-3.999674	-1.557878
H	4.967464	4.388525	2.633221	H	4.085777	-5.237195	-2.671209
C	6.298628	3.222725	-0.786886	C	5.512025	-4.382480	0.802771
H	5.347465	4.932877	-1.618303	H	4.206778	-5.868721	1.583031
C	6.480989	2.393450	0.332283	C	5.891620	-3.598400	-0.299558
H	6.185194	2.306036	2.469538	H	5.691187	-3.437919	-2.443403
H	6.675394	2.912171	-1.756559	H	5.910686	-4.158820	1.787454
C	6.840948	0.965254	0.204646	C	6.513049	-2.267459	-0.135970
C	6.496448	0.267680	-0.965560	C	6.252724	-1.530382	1.031446
C	7.282827	0.200983	1.299864	C	7.137884	-1.586306	-1.196105
C	6.431082	-1.118774	-0.985840	C	6.438914	-0.156860	1.073183
H	6.137530	0.813682	-1.830781	H	5.760764	-2.008616	1.870619
C	7.211605	-1.187396	1.283255	C	7.325344	-0.208765	-1.155113
H	7.633740	0.699564	2.198407	H	7.429670	-2.131051	-2.089151
C	6.704832	-1.873602	0.166468	C	6.897915	0.549928	-0.051345
H	6.015496	-1.608769	-1.859971	H	6.077619	0.385636	1.939455
H	7.500116	-1.745011	2.169841	H	7.757486	0.290963	-2.017278
C	6.180978	-3.251746	0.252888	C	6.650241	2.004117	-0.128168
C	5.513524	-3.650904	1.420419	C	6.261468	2.588685	-1.344645
C	6.065604	-4.094010	-0.865332	C	6.517992	2.795366	1.024977
C	4.622356	-4.713766	1.411025	C	5.605031	3.811372	-1.378904
H	5.584156	-3.037000	2.312176	H	6.365922	2.027607	-2.267729
C	5.171968	-5.158380	-0.872532	C	5.832483	4.001909	0.994032
H	6.633388	-3.870745	-1.764482	H	6.884363	2.420606	1.975833
C	4.343034	-5.429977	0.233655	C	5.269265	4.495965	-0.196904
H	4.049513	-4.896002	2.311773	H	5.265442	4.183952	-2.338964
H	5.067654	-5.732118	-1.787057	H	5.650497	4.512174	1.933456
C	3.082166	-6.201288	0.098061	C	4.172825	5.492672	-0.163693
C	2.624431	-6.645066	-1.157209	C	3.825521	6.186810	1.011125
C	2.164637	-6.289321	1.161486	C	3.269352	5.580491	-1.237847
C	1.290958	-6.971710	-1.372374	C	2.572266	6.769388	1.164121

H	3.296525	-6.663000	-2.008048	H	4.517563	6.223402	1.846221
C	0.830523	-6.604664	0.947176	C	2.025194	6.175500	-1.092660
H	2.458678	-6.004315	2.164272	H	3.473339	5.058809	-2.164587
C	0.341658	-6.868036	-0.342401	C	1.613510	6.705653	0.139695
H	0.970206	-7.245441	-2.373322	H	2.313846	7.233692	2.111805
H	0.134886	-6.514973	1.775060	H	1.308198	6.092318	-1.902643
C	-1.104809	-6.744406	-0.612883	C	0.168593	6.887723	0.383442
C	-2.086768	-7.000624	0.359809	C	-0.726960	7.276761	-0.626583
C	-1.520689	-6.097782	-1.787516	C	-0.382657	6.390941	1.574500
C	-3.372813	-6.487334	0.234883	C	-2.089301	7.022283	-0.514094
H	-1.826086	-7.563647	1.251432	H	-0.344646	7.733685	-1.535155
C	-2.800323	-5.577660	-1.905427	C	-1.739430	6.121263	1.677252
H	-0.792784	-5.861646	-2.555878	H	0.276591	6.063737	2.371752
C	-3.734692	-5.680442	-0.860245	C	-2.617291	6.344312	0.601343
H	-4.086592	-6.671508	1.031961	H	-2.739004	7.302499	-1.337332
H	-3.018452	-4.958732	-2.767222	H	-2.084160	5.596399	2.559587
C	-4.890912	-4.756528	-0.818739	C	-3.923523	5.646608	0.555178
C	-5.302541	-4.037195	-1.955286	C	-4.455060	5.005792	1.688503
C	-5.447865	-4.361987	0.410924	C	-4.548653	5.366886	-0.674089
C	-6.046418	-2.870583	-1.840943	C	-5.398562	3.994834	1.571046
H	-4.965368	-4.341094	-2.940734	H	-4.063847	5.231752	2.674384
C	-6.219607	-3.213561	0.521580	C	-5.513066	4.374955	-0.789008
H	-5.194117	-4.898861	1.317969	H	-4.212990	5.860752	-1.579267
C	-6.449179	-2.380622	-0.586605	C	-5.887224	3.592736	0.316692
H	-6.220512	-2.276506	-2.732241	H	-5.672156	3.434556	2.459109
C	-6.817731	-0.957600	-0.427640	C	-6.513613	2.263377	0.159089
C	-6.456817	-0.285418	0.752391	C	-6.267035	1.526405	-1.011133
C	-7.273869	-0.166328	-1.497659	C	-7.128522	1.582676	1.225494
C	-6.373439	1.097812	0.797355	C	-6.454154	0.153077	-1.050953
H	-6.090416	-0.847946	1.602934	H	-5.782519	2.003802	-1.854888
C	-7.191429	1.221382	-1.453046	C	-7.317853	0.205401	1.186137
H	-7.641951	-0.642022	-2.401857	H	-7.410510	2.127404	2.121688
C	-6.650436	1.881565	-0.335537	C	-6.901460	-0.553495	0.078121
H	-5.938956	1.557800	1.677545	H	-6.101007	-0.389771	-1.920326
H	-7.495434	1.795647	-2.323421	H	-7.741395	-0.294199	2.052623
C	-6.110815	3.255418	-0.400713	C	-6.650493	-2.007194	0.152525
C	-0.814689	1.619220	-2.991566	C	-1.227915	-1.478562	3.010473
C	-2.218619	1.039854	-2.857826	C	-2.490363	-0.638585	2.853934
C	0.263781	0.880697	-3.352121	C	-0.039500	-0.966192	3.414732
C	-2.287753	-0.566831	-2.921691	C	-2.255905	0.949694	2.956993
C	-2.773196	-1.229221	-1.637395	C	-2.568944	1.716914	1.676913
C	-2.654790	1.635855	-1.523694	C	-2.991120	-1.114109	1.494178
C	-3.278348	0.924742	-0.519636	C	-3.434642	-0.278092	0.489232
C	-3.341122	-0.548959	-0.583290	C	-3.216690	1.177246	0.585316
C	1.877569	2.596073	-2.152553	C	1.251173	-2.936483	2.214700
C	0.569934	3.147677	-1.601344	C	-0.121274	-3.219118	1.619176
C	1.702550	1.331699	-3.138813	C	1.292462	-1.680065	3.226150
C	-0.662696	2.750964	-2.066138	C	-1.268567	-2.601429	2.062571
C	-1.802593	2.725098	-1.171038	C	-2.356098	-2.341857	1.140978
C	0.728209	3.474100	-0.209774	C	0.012022	-3.546297	0.225652
C	-0.358706	3.480932	0.671358	C	-1.030984	-3.329839	-0.682067
C	-1.659897	3.101675	0.176925	C	-2.249787	-2.716656	-0.211584
C	3.106235	-0.053053	-1.546380	C	2.974898	-0.559011	1.698707
C	3.270567	1.087744	-0.623069	C	2.945938	-1.693459	0.753593
C	2.324131	0.032244	-2.644474	C	2.163588	-0.513865	2.778246
C	2.617763	2.277819	-0.857922	C	2.074623	-2.742630	0.946020
C	1.990068	2.938317	0.238069	C	1.365338	-3.254161	-0.179636
C	3.406521	0.572054	0.717476	C	3.214228	-1.189578	-0.571771
C	2.852747	1.242458	1.790606	C	2.578749	-1.726835	-1.673564

C	2.098309	2.428225	1.545532	C	1.606282	-2.752257	-1.472440
C	1.670153	-2.518316	-2.371715	C	2.015618	2.120031	2.548548
C	2.430216	-2.390539	-1.059029	C	2.771622	1.871683	1.250928
C	1.595954	-1.163560	-3.241015	C	1.660645	0.788400	3.385755
C	3.180024	-1.281202	-0.727890	C	3.301164	0.646564	0.909988
C	3.357424	-0.898452	0.651437	C	3.437303	0.262663	-0.474932
C	1.753350	-3.094767	-0.022720	C	2.268069	2.711094	0.215332
C	1.919119	-2.717743	1.321627	C	2.385833	2.332837	-1.135488
C	2.768067	-1.624144	1.668331	C	3.020183	1.103458	-1.487893
C	-0.940776	-1.250464	-3.109434	C	-0.808586	1.360833	3.194497
C	-0.885139	-2.463587	-2.281346	C	-0.499106	2.557505	2.397928
C	0.198380	-0.579854	-3.404066	C	0.173292	0.479068	3.501015
C	0.314439	-3.005478	-1.864586	C	0.791127	2.866527	2.022881
C	0.439180	-3.477715	-0.514018	C	1.044499	3.325169	0.683403
C	-2.020456	-2.430000	-1.387633	C	-1.596501	2.747848	1.471741
C	-1.914581	-2.956225	-0.091185	C	-1.353358	3.248058	0.181556
C	-0.652555	-3.493811	0.358892	C	-0.000247	3.538279	-0.222827
C	0.939188	-1.623954	3.141555	C	1.269778	1.478270	-3.014032
C	0.781460	-2.723838	2.223624	C	1.300287	2.582757	-2.060702
C	-0.477469	-3.111901	1.753000	C	0.137213	3.184330	-1.612431
C	-0.163678	-0.873585	3.492228	C	0.061798	0.960186	-3.390648
C	1.027559	1.257290	3.276644	C	0.827029	-1.367298	-3.198664
C	2.381730	0.575769	3.078236	C	2.277267	-0.958837	-2.956354
C	2.337937	-1.032519	3.011359	C	2.527687	0.630268	-2.855652
C	-0.108727	0.597462	3.591419	C	-0.153817	-0.490277	-3.507788
C	-1.591501	2.526856	2.529576	C	-2.004771	-2.124862	-2.544947
C	-0.232616	3.010055	2.027003	C	-0.778604	-2.870646	-2.022450
C	0.965984	2.474370	2.445786	C	0.511513	-2.565868	-2.400436
C	-1.516055	1.167630	3.394702	C	-1.643046	-0.789595	-3.370286
C	-3.063149	0.070974	1.672919	C	-3.000198	0.550338	-1.681109
C	-3.116357	1.306211	0.866837	C	-3.309624	-0.661130	-0.899949
C	-2.352704	2.402504	1.210724	C	-2.766349	-1.881775	-1.245474
C	-2.222612	-0.022124	2.762780	C	-2.130972	0.505157	-2.737609
C	-1.632710	-2.339558	2.146084	C	-1.190305	2.786507	-2.152936
C	-2.521790	-2.248087	1.010564	C	-2.059165	2.717767	-0.946838
C	-3.205202	-1.050831	0.766876	C	-2.951007	1.672785	-0.746347
C	-1.471147	-1.226139	2.984706	C	-1.207174	1.594567	-3.015412
H	-6.573531	-2.916026	1.503296	H	-5.919749	4.151682	-1.770541
C	2.197458	-1.658707	-4.634438	C	2.306613	1.136899	4.802659
C	2.329628	-3.531103	-3.399905	C	2.829197	2.970164	3.613066
C	1.637482	-3.090244	-4.701928	C	2.029299	2.648568	4.888401
H	0.545202	-3.133538	-4.655704	H	0.967409	2.901503	4.816005
H	1.997492	-3.638059	-5.577461	H	2.462925	3.101889	5.784367
C	3.745909	-3.048874	-3.647654	C	4.119489	2.221620	3.886680
H	2.211724	-4.565501	-3.077103	H	2.921215	4.014433	3.310024
C	3.668475	-1.934153	-4.388181	C	3.809519	1.129534	4.599346
H	4.625859	-3.473717	-3.181417	H	5.079911	2.477811	3.457838
H	1.948737	-0.987882	-5.457304	H	1.911909	0.512836	5.605077
H	4.477561	-1.262740	-4.653932	H	4.468142	0.311936	4.870755
H	-1.944365	0.934740	5.580034	H	-1.924115	-0.460305	-5.571369
C	-2.162572	1.632882	4.770547	C	-2.310812	-1.105079	-4.781033
H	-4.442468	1.242697	4.727706	H	-4.473827	-0.280534	-4.811841
C	-3.626446	1.916364	4.491251	C	-3.811736	-1.102282	-4.561954
C	-1.602761	3.064203	4.876670	C	-2.034603	-2.615585	-4.897841
H	-0.509489	3.106884	4.856391	H	-0.971893	-2.869339	-4.840368
H	-1.983100	3.600243	5.750806	H	-2.477297	-3.053758	-5.796881
C	-3.685795	3.046126	3.772988	C	-4.114952	-2.207963	-3.867658
C	-2.262274	3.527183	3.563349	C	-2.821126	-2.959830	-3.619333
H	-4.557421	3.482410	3.301472	H	-5.070892	-2.472214	-3.433501

H	-2.134555	4.567748	3.260271
C	-3.295518	-0.794052	-4.130086
C	-2.585282	-0.323282	-5.385151
C	-2.525421	1.014359	-5.332806
C	-4.281606	0.365675	-3.896101
H	-3.696361	-1.809131	-4.139898
C	-3.195000	1.447756	-4.042757
H	-4.758624	0.349058	-2.912514
H	-5.043450	0.429967	-4.678667
H	-2.105807	-0.975954	-6.105891
H	-1.987772	1.676061	-6.002598
H	-3.503558	2.492213	-3.972949
C	3.395223	0.816362	4.278380
C	2.706194	0.329840	5.538989
H	2.222775	0.971943	6.266409
H	3.778320	1.838366	4.288533
C	2.668272	-1.008189	5.482714
C	3.332588	-1.426242	4.184749
H	3.657192	-2.465876	4.112237
H	2.147454	-1.681482	6.154088
H	4.866053	-0.296822	3.042125
C	4.399895	-0.325414	4.031609
H	5.170270	-0.379407	4.806403
C	2.664049	3.625202	-3.069755
C	3.584549	2.666145	-3.843341
H	4.226108	2.061143	-3.195075
H	4.188401	3.177306	-4.598632
C	2.412787	1.877772	-4.458973
C	1.550147	3.005790	-4.990038
H	0.850207	2.912973	-5.812679
C	1.703183	4.049303	-4.163348
H	1.154069	4.984321	-4.174791
H	3.120840	4.420405	-2.482021
H	2.658004	1.082545	-5.162544
C	-3.919656	-2.234514	5.083956
C	-2.877285	-2.883019	5.645019
C	-2.606856	-4.111315	4.889150
C	-4.413746	-3.025846	3.902029
H	-4.370101	-2.450401	2.967965
H	-5.464579	-3.331405	4.026138
C	-3.484095	-4.209820	3.868587
H	-3.515910	-4.984286	3.111721
H	-4.358745	-1.299931	5.412484
H	-2.308882	-2.558374	6.510254
H	-1.805102	-4.805969	5.115866

H	-2.909910	-4.009233	-3.333645
C	-3.236801	1.339581	4.144786
C	-2.668250	0.716169	5.405559
C	-2.862672	-0.606813	5.320048
C	-4.418843	0.394698	3.855871
H	-3.436764	2.412270	4.171898
C	-3.562365	-0.877521	4.001895
H	-4.853753	0.522296	2.860452
H	-5.201947	0.460875	4.617021
H	-2.095250	1.250193	6.155117
H	-2.481358	-1.372888	5.985827
H	-4.060927	-1.842767	3.897310
C	3.260566	-1.359910	-4.137519
C	2.708146	-0.729943	-5.402240
H	2.133377	-1.257083	-6.155307
H	3.448385	-2.434838	-4.163375
C	2.918493	0.590444	-5.315859
C	3.612614	0.853218	-3.992868
H	4.122622	1.812245	-3.885649
H	2.550254	1.360985	-5.983933
H	4.877900	-0.561908	-2.841606
C	4.452185	-0.429367	-3.840376
H	5.240477	-0.505783	-4.595251
C	1.801156	-4.114108	3.125890
C	2.865021	-3.363091	3.944458
H	3.628470	-2.879401	3.326971
H	3.338496	-3.994051	4.702151
C	1.847562	-2.376820	4.549509
C	0.771170	-3.329132	5.032257
H	0.077694	-3.119172	5.838644
C	0.746373	-4.367310	4.185347
H	0.028177	-5.179399	4.160249
H	2.114992	-4.971064	2.530205
H	2.219586	-1.656434	5.277731
C	-2.135622	2.621221	-4.901164
H	-2.522080	1.803309	-5.496396
C	-1.011988	3.392124	-5.218645
H	-0.291311	3.156259	-5.993839
C	-0.875339	4.413676	-4.274490
H	-0.027853	5.084995	-4.196672
C	-2.970818	3.421128	-3.936727
H	-3.584809	2.836929	-3.248659
H	-3.637182	4.089943	-4.505556
C	-1.885245	4.261359	-3.287468
H	-2.110102	5.029626	-2.555606

C60CPP10_6_Prod

226

C60CPP10_6_Prod_3 SCF Done: -5763.44561845 A.U.

C	-3.567967	-5.378063	1.287418
C	-2.360850	-6.046469	1.149580
C	-1.981050	-6.612849	-0.076797
C	-2.941283	-6.630759	-1.101723
C	-4.158327	-5.974423	-0.955748
C	-4.464588	-5.249388	0.211796
H	-3.740922	-4.835308	2.208329
H	-1.640305	-5.995713	1.958985
H	-2.709963	-7.119254	-2.044244
H	-4.851469	-5.979155	-1.790734
C	-0.549257	-6.882621	-0.316017

C	0.319140	-7.316109	0.699360
C	0.032936	-6.426524	-1.508672
C	1.694148	-7.141547	0.589811
H	-0.091978	-7.742846	1.610026
C	1.403280	-6.236546	-1.608805
H	-0.604102	-6.067281	-2.310160
C	2.263541	-6.503016	-0.528365
H	2.323985	-7.451091	1.417847
H	1.780291	-5.738456	-2.493440
C	-5.501411	-4.190809	0.234678
C	-6.032919	-3.674413	-0.961271
C	-5.800185	-3.479825	1.410992
C	-6.649541	-2.431588	-1.002650
H	-5.877434	-4.201052	-1.896421
C	-6.387523	-2.222946	1.366487
H	-5.484469	-3.864433	2.374453
H	-6.990882	-2.043242	-1.957397
H	-6.462749	-1.650553	2.285441
C	3.606679	-5.879342	-0.481675
C	4.174914	-5.274586	-1.616926
C	4.241369	-5.625700	0.748355
C	5.168048	-4.312365	-1.501557
H	3.775969	-5.486086	-2.602892
C	5.254088	-4.682914	0.861553
H	3.877116	-6.095590	1.655097
C	5.671128	-3.927796	-0.247400
H	5.473649	-3.772452	-2.391782
H	5.667134	-4.473894	1.843537
C	6.364282	-2.631432	-0.096886
C	6.148452	-1.870985	1.064572
C	7.021678	-1.995530	-1.165344
C	6.410754	-0.509497	1.093587
H	5.634838	-2.314943	1.909633
C	7.285598	-0.630458	-1.137249
H	7.279903	-2.563760	-2.054047
C	6.905503	0.159869	-0.038504
H	6.083571	0.061082	1.955550
H	7.742011	-0.162444	-2.004703
C	6.741414	1.624779	-0.127198
C	6.365456	2.215632	-1.344348
C	6.675724	2.435749	1.017753
C	5.779301	3.473109	-1.383328
H	6.419827	1.637648	-2.261250
C	6.062639	3.680567	0.981905
H	7.036097	2.051319	1.967221
C	5.505882	4.191339	-0.205239
H	5.442535	3.850141	-2.342382
H	5.932591	4.214110	1.916913
C	4.469742	5.250820	-0.168526
C	4.158992	5.953141	1.011755
C	3.576852	5.402581	-1.244507
C	2.941546	6.606419	1.166791
H	4.847376	5.940705	1.850392
C	2.368518	6.066680	-1.096929
H	3.753634	4.882470	-2.177587
C	1.983884	6.607546	0.139474
H	2.707454	7.075776	2.118292
H	1.651019	6.032015	-1.909969
C	0.551449	6.869945	0.381866

C	-0.316739	7.317056	-0.627679
C	-0.030874	6.395798	1.567489
C	-1.691506	7.140567	-0.520843
H	0.094335	7.757100	-1.531984
C	-1.401326	6.204839	1.664909
H	0.605906	6.024406	2.363540
C	-2.261380	6.487908	0.588642
H	-2.320691	7.460704	-1.344870
H	-1.779091	5.693351	2.541615
C	-3.605826	5.868084	0.534910
C	-4.183040	5.264313	1.666144
C	-4.233927	5.618574	-0.699195
C	-5.179466	4.306135	1.543474
H	-3.788971	5.473976	2.654608
C	-5.249990	4.680463	-0.819670
H	-3.860991	6.087315	-1.603037
C	-5.676465	3.925187	0.285624
H	-5.492927	3.766465	2.431163
C	-6.371571	2.630611	0.127512
C	-6.151673	1.874076	-1.035804
C	-7.034033	1.990783	1.190492
C	-6.411966	0.512418	-1.070027
H	-5.635320	2.320804	-1.877652
C	-7.296507	0.625481	1.156848
H	-7.296666	2.555698	2.080032
C	-6.909138	-0.161773	0.058263
H	-6.080707	-0.053949	-1.933099
H	-7.756680	0.155006	2.020935
C	-6.741079	-1.626658	0.144888
C	-1.304454	-1.387188	3.003291
C	-2.525302	-0.489649	2.836990
C	-0.093938	-0.928230	3.405512
C	-2.217551	1.086586	2.930802
C	-2.488793	1.859337	1.644088
C	-3.041844	-0.950903	1.478331
C	-3.429839	-0.101974	0.461247
C	-3.145523	1.340293	0.546187
C	1.104091	-2.967057	2.223678
C	-0.279092	-3.190282	1.627646
C	1.203061	-1.705335	3.224679
C	-1.397297	-2.515623	2.064994
C	-2.467751	-2.212182	1.138617
C	-0.159625	-3.538478	0.239197
C	-1.191012	-3.283103	-0.673053
C	-2.379326	-2.607889	-0.209615
C	2.936272	-0.676255	1.689977
C	2.855234	-1.816273	0.754241
C	2.128014	-0.584532	2.768881
C	1.937432	-2.823595	0.954770
C	1.206897	-3.313334	-0.167281
C	3.145789	-1.336701	-0.575202
C	2.489167	-1.856021	-1.673139
C	1.471834	-2.835352	-1.464079
C	2.104813	2.052543	2.519103
C	2.847091	1.758544	1.222993
C	1.686946	0.744549	3.366271
C	3.316432	0.506854	0.891645
C	3.430021	0.105703	-0.490211
C	2.379151	2.611391	0.180769

C	2.467493	2.216165	-1.167545
C	3.041454	0.954664	-1.507095
C	-0.752755	1.431264	3.169645
C	-0.385967	2.606758	2.365064
C	0.186534	0.506238	3.482098
C	0.916448	2.851741	1.987781
C	1.190750	3.286245	0.644132
C	-1.471311	2.838234	1.434872
C	-1.206766	3.316993	0.138274
C	0.159559	3.542690	-0.268230
C	1.304601	1.391124	-3.032037
C	1.396976	2.520046	-2.093995
C	0.278864	3.195891	-1.657404
C	0.094258	0.931493	-3.433472
C	0.752852	-1.427980	-3.198391
C	2.217571	-1.083002	-2.959623
C	2.525282	0.493437	-2.865683
C	-0.186389	-0.502912	-3.510793
C	-2.104529	-2.049024	-2.547810
C	-0.916321	-2.848649	-2.016779
C	0.386164	-2.603784	-2.394103
C	-1.686826	-0.741169	-3.395180
C	-2.935901	0.679930	-1.718927
C	-3.315990	-0.503130	-0.920549
C	-2.846507	-1.754751	-1.251789
C	-2.127751	0.588082	-2.797920
C	-1.105227	2.972144	-2.253199
C	-1.937466	2.827550	-0.983814
C	-2.854994	1.819977	-0.783250
C	-1.202443	1.708569	-3.253696
H	-5.657491	4.474157	-1.804510
C	2.348386	1.072928	4.780719
C	2.957910	2.870348	3.577752
C	2.143488	2.596678	4.855012
H	1.094847	2.899388	4.780336
H	2.598146	3.035380	5.747814
C	4.210932	2.062739	3.856977
H	3.100082	3.906642	3.267094
C	3.849209	0.992329	4.578043
H	5.182056	2.268922	3.425458
H	1.924274	0.474499	5.587653
H	4.468002	0.146227	4.855804
H	-1.925291	-0.471815	-5.616679
C	-2.348998	-1.069837	-4.809263
H	-4.468435	-0.142502	-4.883224
C	-3.849741	-0.988743	-4.605743
C	-2.144571	-2.593630	-4.883515
H	-1.095998	-2.896649	-4.809261
H	-2.599808	-3.032311	-5.776022
C	-4.211380	-2.059043	-3.884478
C	-2.958362	-2.866828	-3.605798
H	-5.182706	-2.265020	-3.453025
H	-3.100466	-3.903128	-3.294953
C	-3.182993	1.529271	4.112127
C	-2.649260	0.888072	5.379223
C	-2.905153	-0.424859	5.301328
C	-4.406875	0.638874	3.824147
H	-3.332826	2.610254	4.131550
C	-3.611502	-0.671068	3.981882

H	-4.831062	0.780514	2.825958
H	-5.189313	0.746629	4.581217
H	-2.055192	1.399570	6.128101
H	-2.562948	-1.203573	5.973669
H	-4.154361	-1.612535	3.880967
C	3.183052	-1.525438	-4.141031
C	2.649660	-0.883989	-5.408118
H	2.055789	-1.395366	-6.157225
H	3.332746	-2.606434	-4.160855
C	2.905705	0.428878	-5.329982
C	3.611730	0.674795	-4.010314
H	4.154497	1.616364	-3.909536
H	2.563855	1.207781	-6.002275
H	4.830730	-0.777289	-2.854132
C	4.407047	-0.635244	-3.852645
H	5.189635	-0.742948	-4.609531
C	1.596575	-4.161384	3.145798
C	2.694042	-3.454133	3.958952
H	3.479532	-3.012100	3.337925
H	3.137039	-4.100172	4.722340
C	1.723712	-2.416134	4.554596
C	0.603506	-3.312723	5.044416
H	-0.079544	-3.063754	5.848615
C	0.530468	-4.355751	4.206269
H	-0.224759	-5.133710	4.187445
H	1.870319	-5.036983	2.557862
H	2.128678	-1.707836	5.277038
C	-1.720853	2.416657	-4.585399
H	-2.121979	1.706611	-5.308216
C	-0.601821	3.315859	-5.073245
H	0.084023	3.067858	-5.875361
C	-0.533796	4.360150	-4.236261
H	0.219690	5.139739	-4.215990
C	-2.694948	3.451820	-3.992791
H	-3.480485	3.007684	-3.373236
H	-3.138241	4.096597	-4.757058
C	-1.601488	4.163458	-3.177628
H	-1.886544	5.041133	-2.597644

Table S6. Coordinates data set, absolute energies (a.u.) for all DFT optimized C_{60} complexes.

C60	C60_1
60	71
C60 SCF Done: -2286.90700177 A.U.	C60_1_Int SCF Done: -2481.08737916 A.U.
C -1.525707 3.205322 -0.113602	C 0.544878 -0.232214 3.355268
C -1.852114 2.673096 -1.427756	C 0.067187 1.140570 3.297783
C -0.207256 3.540206 0.195336	C 1.688940 -0.602094 2.648340
C -0.846865 2.497783 -2.378635	C 0.754379 2.087099 2.536686
C -0.824967 1.298031 -3.201352	C 0.015774 3.042977 1.725391
C -2.878856 1.656179 -1.258482	C -1.386991 1.109507 3.279571
C -2.857783 0.505994 -2.047231	C -2.095121 2.026302 2.502468
C -1.809260 0.323211 -3.039236	C -1.378839 3.013131 1.708751
C -0.447861 2.541988 2.439638	C 0.613969 -2.712003 1.949546
C -1.822934 2.192631 2.117380	C -0.580369 -2.325179 2.685867
C 0.342993 3.201629 1.498758	C 1.724261 -1.867517 1.930300
C -2.350573 2.517362 0.867775	C -0.614210 -1.112351 3.373806
C -3.186933 1.559875 0.160164	C -1.808207 -0.282527 3.326852
C -2.108996 0.896537 2.713334	C -1.739130 -2.760663 1.921727

C	-2.910804	-0.021296	2.035024	C	-2.884221	-1.965392	1.877131
C	-3.461182	0.317373	0.731454	C	-2.919531	-0.700133	2.594615
C	2.272112	1.774008	2.074839	C	2.061945	-2.310855	-0.472984
C	1.447280	1.085877	3.056185	C	0.902221	-3.188194	-0.454512
C	1.731532	2.809420	1.312633	C	2.462031	-1.662280	0.694099
C	0.116026	1.461836	3.234668	C	0.193800	-3.386501	0.731701
C	-0.910737	0.444968	3.403972	C	-1.260594	-3.416023	0.713779
C	1.809221	-0.323176	3.039117	C	0.185388	-3.013200	-1.708770
C	0.825025	-1.298052	3.201463	C	-1.209120	-3.043031	-1.725401
C	-0.563685	-0.905822	3.387525	C	-1.947435	-3.248646	-0.488620
C	2.875285	1.962693	-0.703823	C	2.885332	0.417338	-0.565500
C	3.439222	0.882394	0.091274	C	2.461375	-0.257194	-1.783585
C	2.039549	2.905636	-0.106130	C	2.885547	-0.270515	0.647460
C	3.144030	0.790123	1.451275	C	2.061697	-1.591796	-1.740951
C	2.857864	-0.505973	2.047266	C	0.901822	-2.026482	-2.503012
C	3.460973	-0.317267	-0.731460	C	1.723404	0.698206	-2.594260
C	3.186923	-1.559984	-0.160136	C	0.612911	0.281060	-3.328458
C	2.878849	-1.656159	1.258530	C	0.192897	-1.110250	-3.281538
C	0.528234	2.847219	-2.056344	C	1.949178	1.702223	1.801812
C	1.400105	1.863275	-2.679914	C	1.949034	2.420682	0.535002
C	0.841188	3.357471	-0.796607	C	2.404513	0.384693	1.854024
C	2.548952	1.430378	-2.018048	C	2.404180	1.789105	-0.622515
C	2.910686	0.021345	-2.034834	C	1.688077	1.963984	-1.876883
C	0.563719	0.905874	-3.387576	C	0.753934	3.248630	0.488377
C	0.910700	-0.444924	-3.404003	C	0.066400	3.415930	-0.714368
C	2.108945	-0.896614	-2.713369	C	0.543763	2.760213	-1.921925
C	1.525605	-3.205347	0.113597	C	-1.740249	0.231793	-3.355528
C	2.350557	-2.517366	-0.867770	C	-0.581589	1.111397	-3.374417
C	1.822922	-2.192637	-2.117377	C	-0.615429	2.324140	-2.686331
C	0.207354	-3.540405	-0.195308	C	-2.885091	0.601567	-2.649654
C	-0.528174	-2.847200	2.056456	C	-3.141961	-1.701546	-1.800635
C	0.846886	-2.497713	2.378583	C	-1.947885	-2.087332	-2.536545
C	1.852124	-2.673115	1.427699	C	-1.261390	-1.141149	-3.297547
C	-0.841197	-3.357422	0.796639	C	-3.600903	-0.385471	-1.855983
C	-2.875308	-1.962573	0.703852	C	-4.078628	-0.418313	0.565158
C	-2.548892	-1.430387	2.018037	C	-3.600547	-1.791181	0.622871
C	-1.400002	-1.863277	2.679997	C	-3.141817	-2.419345	-0.535001
C	-2.039479	-2.905692	0.106072	C	-4.078935	0.269729	-0.648147
C	-2.272146	-1.773931	-2.074940	C	-3.254400	2.308284	0.473015
C	-3.144025	-0.790092	-1.451405	C	-3.254143	1.590495	1.738749
C	-3.439175	-0.882426	-0.091302	C	-3.657602	0.256090	1.783752
C	-1.731566	-2.809379	-1.312613	C	-3.658295	1.661856	-0.695119
C	0.447787	-2.542018	-2.439561	C	-1.809259	2.709833	-1.949936
C	-0.115980	-1.461863	-3.234648	C	-1.387890	3.384401	-0.731294
C	-1.447220	-1.085836	-3.056150	C	-2.095522	3.187613	0.454663
C	-0.342972	-3.201786	-1.498928	C	-2.920408	1.866823	-1.932120
				C	5.954765	-0.331431	1.167307
				C	5.955278	0.830921	-0.884498
				C	5.946824	1.063672	0.602494
				H	5.074559	1.645086	0.931642
				H	6.830781	1.634621	0.926155
				C	6.004240	-0.498371	-1.123912
				H	5.939866	1.624983	-1.621241
				C	6.003890	-1.220167	0.150212
				H	6.023780	-0.974441	-2.098240
				H	5.939091	-0.555119	2.227157
				H	6.023186	-2.300629	0.242783
**C60_1_TS**				**C60_1_Prod**			
71				71			

C60_1_TS SCF Done: -2481.06877894 A.U.				C60_1_Prod SCF Done: -2481.10842974 A.U.			
C	0.565383	3.032874	-1.473999	C	0.559549	3.037563	-1.483522
C	0.070290	2.310873	-2.628632	C	0.052422	2.316975	-2.628061
C	1.716340	2.585914	-0.821179	C	1.719347	2.582861	-0.847368
C	0.754311	1.177692	-3.079770	C	0.733273	1.180331	-3.076979
C	0.008735	-0.000767	-3.494539	C	-0.016052	0.002949	-3.483185
C	-1.381986	2.308040	-2.567175	C	-1.398945	2.313986	-2.552892
C	-2.095704	1.177064	-2.965869	C	-2.115459	1.181032	-2.942155
C	-1.385345	-0.000750	-3.441582	C	-1.409242	0.002904	-3.424720
C	0.686311	3.035487	1.378375	C	0.717085	3.036908	1.364283
C	-0.519549	3.487672	0.698200	C	-0.497278	3.490810	0.701369
C	1.777490	2.586146	0.629938	C	1.799515	2.583159	0.603352
C	-0.578529	3.486656	-0.694721	C	-0.574179	3.491701	-0.690137
C	-1.783990	3.036438	-1.372236	C	-1.787709	3.041751	-1.353092
C	-1.663007	3.037496	1.475805	C	-1.629972	3.039252	1.493566
C	-2.819453	2.603815	0.826943	C	-2.794463	2.604642	0.858962
C	-2.881324	2.603372	-0.627206	C	-2.875114	2.606069	-0.594539
C	2.158113	0.736858	2.188975	C	2.202451	0.739335	2.136821
C	1.012862	1.179434	2.959799	C	1.066779	1.175135	2.923960
C	2.530344	1.424067	1.045099	C	2.571460	1.439153	1.009165
C	0.292234	2.313943	2.570247	C	0.340259	2.313059	2.556083
C	-1.160038	2.309696	2.632453	C	-1.110612	2.309455	2.641402
C	0.305232	0.000751	3.436534	C	0.366542	-0.002942	3.408425
C	-1.088067	0.000771	3.503657	C	-1.024420	-0.003040	3.504747
C	-1.836755	1.178603	3.090479	C	-1.779685	1.175834	3.105273
C	3.066106	-0.730661	-0.151132	C	3.247520	-0.804494	-0.204214
C	2.530293	-1.423619	1.045712	C	2.571648	-1.440938	1.006805
C	3.066151	0.730570	-0.151461	C	3.247430	0.804766	-0.202805
C	2.158133	-0.735930	2.189326	C	2.202397	-0.742911	2.135328
C	1.012871	-1.178156	2.960345	C	1.066667	-1.180110	2.921981
C	1.777457	-2.585887	0.631065	C	1.799470	-2.584350	0.598935
C	0.686269	-3.034866	1.379697	C	0.717070	-3.039394	1.359038
C	0.292212	-2.312812	2.571268	C	0.340247	-2.317517	2.552264
C	1.960476	0.733866	-2.407238	C	1.947713	0.743034	-2.419504
C	1.960471	-0.734935	-2.406915	C	1.947779	-0.738969	-2.420756
C	2.432766	1.421782	-1.298619	C	2.440793	1.437401	-1.337068
C	2.432779	-1.422387	-1.298004	C	2.440694	-1.435093	-1.339438
C	1.716320	-2.586296	-0.820054	C	1.719331	-2.581539	-0.851589
C	0.754299	-1.179047	-3.079235	C	0.733201	-1.175051	-3.078964
C	0.070270	-2.312033	-2.627613	C	0.052345	-2.312447	-2.632027
C	0.565357	-3.033536	-1.472673	C	0.559478	-3.035133	-1.488617
C	-1.663037	-3.036829	1.477136	C	-1.630028	-3.041693	1.488401
C	-0.519589	-3.487330	0.699723	C	-0.497207	-3.492076	0.695363
C	-0.578565	-3.486989	-0.693199	C	-0.574109	-3.490519	-0.696154
C	-2.819480	-2.603433	0.828081	C	-2.794490	-2.606033	0.854635
C	-3.042296	-0.727569	2.415900	C	-2.992957	-0.730753	2.446129
C	-1.836770	-1.177239	3.091014	C	-1.779733	-1.181161	3.103342
C	-1.160060	-2.308528	2.633472	C	-1.110644	-2.314036	2.637461
C	-3.524383	-1.425169	1.307518	C	-3.490773	-1.427442	1.343661
C	-4.022670	0.697572	0.150484	C	-4.002951	0.697459	0.195301
C	-3.524370	1.425768	1.306894	C	-3.490768	1.425141	1.346079
C	-3.042293	0.728646	2.415585	C	-2.992979	0.726583	2.447315
C	-4.022678	-0.697477	0.150791	C	-4.002955	-0.697742	0.194117
C	-3.239628	-0.728660	-2.190229	C	-3.248725	-0.726767	-2.155928
C	-3.239640	0.727728	-2.190565	C	-3.248743	0.730443	-2.154706
C	-3.624494	1.425138	-1.044955	C	-3.621092	1.427014	-1.003818
C	-3.624530	-1.425578	-1.044336	C	-3.621008	-1.425187	-1.006165
C	-1.784026	-3.037048	-1.370910	C	-1.787756	-3.039380	-1.358317
C	-1.382005	-2.309167	-2.566158	C	-1.398861	-2.309511	-2.556758

C -2.095697 -1.178344 -2.965326	C -2.115427 -1.176011 -2.944079
C -2.881353 -2.603628 -0.626069	C -2.875106 -2.604994 -0.599036
C 5.191438 1.156889 -0.298132	C 4.802213 1.124223 -0.310467
C 5.191542 -1.157100 -0.297394	C 4.802371 -1.123590 -0.312492
C 5.370573 -0.000408 -1.253375	C 5.256007 0.001158 -1.261566
H 4.715831 -0.000723 -2.126413	H 4.736250 0.002013 -2.224517
H 6.412344 -0.000464 -1.612343	H 6.337112 0.001310 -1.422595
C 5.600557 -0.698207 0.971225	C 5.428857 -0.670631 0.992933
H 5.212237 -2.194930 -0.609852	H 4.988450 -2.154622 -0.616007
C 5.600523 0.698831 0.970775	C 5.428805 0.669097 0.994087
H 5.744165 -1.324082 1.844324	H 5.701317 -1.330940 1.808117
H 5.212160 2.194512 -0.611276	H 4.988095 2.155818 -0.612218
H 5.744098 1.325276 1.843471	H 5.701119 1.328032 1.810431
**C60_2_Int**	**C60_2_TS**
82	82
C60_2_Int_3 SCF Done: -2675.28832287 A.U.	C60_2_TS_3 SCF Done: -2675.26808271 A.U.
C 1.288009 3.039451 -1.363527	C -1.253150 -3.041723 -1.361043
C 0.892884 2.317294 -2.550738	C -0.865011 -2.318239 -2.550689
C 2.382121 2.584577 -0.619985	C -2.340957 -2.585253 -0.608301
C 1.614067 1.180189 -2.931505	C -1.586812 -1.178896 -2.923284
C 0.906735 0.002864 -3.407145	C -0.880709 -0.001092 -3.400109
C -0.558924 2.314121 -2.614632	C 0.586065 -2.317736 -2.625143
C -1.235349 1.180789 -3.070387	C 1.263635 -1.180488 -3.076567
C -0.485745 0.002562 -3.482659	C 0.511963 -0.001120 -3.479881
C 1.174086 3.035605 1.486180	C -1.116682 -3.039911 1.488471
C 0.028587 3.490829 0.711086	C 0.021539 -3.494230 0.703593
C 2.324199 2.582050 0.831617	C -2.271457 -2.583593 0.843031
C 0.084205 3.492254 -0.681490	C -0.044908 -3.494349 -0.688397
C -1.060498 3.041587 -1.457637	C 1.092736 -3.040938 -1.475102
C -1.174743 3.039690 1.392524	C 1.229453 -3.041723 1.377654
C -2.272763 2.603021 0.649356	C 2.315973 -2.588925 0.622621
C -2.214446 2.604666 -0.805609	C 2.246703 -2.589487 -0.828556
C 2.576279 0.739422 2.396830	C -2.505746 -0.740362 2.409679
C 1.371795 1.175262 3.073505	C -1.296031 -1.177268 3.075198
C 3.053096 1.435737 1.308405	C -2.994318 -1.436715 1.325844
C 0.683979 2.312560 2.637080	C -0.614343 -2.316632 2.635019
C -0.768247 2.310301 2.584432	C 0.837216 -2.317765 2.569758
C 0.628793 -0.003043 3.488452	C -0.547270 0.001122 3.480280
C -0.765228 -0.003377 3.450843	C 0.846531 0.001100 3.423812
C -1.479264 1.175668 2.981533	C 1.555817 -1.178951 2.951223
C 3.842962 -0.803965 0.159583	C -3.792619 0.804946 0.184753
C 3.053846 -1.436858 1.305976	C -2.994334 1.437616 1.324937
C 3.842537 0.805195 0.160938	C -3.792623 -0.804792 0.185276
C 2.576670 -0.742640 2.395580	C -2.505743 0.741934 2.409202
C 1.372421 -1.180255 3.071524	C -1.296011 1.179251 3.074405
C 2.325549 -2.582741 0.827254	C -2.271445 2.584170 0.841385
C 1.175685 -3.038016 1.481055	C -1.116648 3.040876 1.486521
C 0.685200 -2.317179 2.633178	C -0.614321 2.318338 2.633525
C 2.761704 0.743456 -2.162493	C -2.728189 -0.741983 -2.145974
C 2.762091 -0.738861 -2.163744	C -2.728184 0.740616 -2.146448
C 3.148128 1.441568 -1.039549	C -3.108203 -1.441240 -1.020938
C 3.148890 -1.438679 -1.041986	C -3.108139 1.440580 -1.021832
C 2.383472 -2.582785 -0.624350	C -2.340923 2.584879 -0.609948
C 1.614684 -1.174896 -2.933496	C -1.586802 1.177013 -2.924022
C 0.894099 -2.313019 -2.554650	C -0.864974 2.316563 -2.552142
C 1.289599 -3.036969 -1.368660	C -1.253107 3.040837 -1.362980
C -1.173140 -3.043172 1.387397	C 1.229494 3.042615 1.375713
C 0.030424 -3.492523 0.705190	C 0.021580 3.494699 0.701357
C 0.086034 -3.491555 -0.687385	C -0.044866 3.493889 -0.690633

C	-2.271395	-2.605823	0.644964	C	2.315999	2.589299	0.620964
C	-2.624741	-0.732722	2.211235	C	2.696346	0.737733	2.176400
C	-1.478641	-1.182006	2.979549	C	1.555825	1.180851	2.950476
C	-0.767024	-2.315596	2.580542	C	0.837251	2.319450	2.568300
C	-3.010862	-1.427477	1.064893	C	3.067019	1.425824	1.031410
C	-3.413999	0.696288	-0.126927	C	3.597585	-0.731843	-0.166943
C	-3.011640	1.423590	1.067290	C	3.066965	-1.425172	1.032315
C	-2.625125	0.727067	2.212442	C	2.696345	-0.736349	2.176875
C	-3.413696	-0.698344	-0.128059	C	3.597581	0.731697	-0.167419
C	-2.438640	-0.728156	-2.397165	C	2.472674	0.734341	-2.414728
C	-2.439027	0.730431	-2.395946	C	2.472666	-0.735905	-2.414247
C	-2.917465	1.425192	-1.283518	C	2.956204	-1.424155	-1.309562
C	-2.916721	-1.425045	-1.285899	C	2.956190	1.423269	-1.310464
C	-1.058907	-3.040172	-1.462767	C	1.092771	3.039985	-1.477054
C	-0.557715	-2.310488	-2.618527	C	0.586089	2.316043	-2.626640
C	-1.234732	-1.176752	-3.072370	C	1.263655	1.178512	-3.077364
C	-2.213089	-2.604969	-0.809998	C	2.246716	2.588926	-0.830208
C	5.399452	-1.122904	0.243733	C	-5.348227	1.123867	0.281431
C	5.398865	1.124811	0.245614	C	-5.348230	-1.123635	0.282250
C	5.866966	0.000285	1.187899	C	-5.808592	0.000462	1.228779
H	5.361308	-0.000666	2.158318	H	-5.294863	0.000816	2.194961
H	6.950402	0.000438	1.332982	H	-6.890900	0.000516	1.382538
C	6.005991	0.672090	-1.068914	C	-5.966007	-0.670245	-1.026999
H	5.588932	2.155941	0.546377	H	-5.536044	-2.154893	0.584054
C	6.006358	-0.667651	-1.070024	C	-5.965992	0.669515	-1.027492
H	6.265294	1.332368	-1.888425	H	-6.230920	-1.329945	-1.845216
H	5.590060	-2.154429	0.542770	H	-5.536046	2.155352	0.582463
H	6.266023	-1.326417	-1.890636	H	-6.230890	1.328615	-1.846198
H	-6.478693	2.208874	-0.692105	H	5.731984	-2.194644	-0.639235
C	-6.497996	1.178571	-0.357920	C	5.712452	-1.156737	-0.326709
H	-6.579076	1.358314	1.809456	H	6.285920	-1.323618	1.810264
C	-6.554869	0.739777	0.918819	C	6.135821	-0.697783	0.938176
C	-6.481823	-0.006455	-1.285806	C	5.888916	-0.000559	-1.284421
H	-5.601782	-0.010974	-1.943752	H	5.230314	-0.000889	-2.154468
H	-7.358402	-0.010251	-1.951933	H	6.929471	-0.000768	-1.646701
C	-6.555559	-0.725042	0.927983	C	6.135872	0.698564	0.937575
C	-6.498966	-1.179791	-0.343163	C	5.712571	1.156449	-0.327706
H	-6.580315	-1.332374	1.826281	H	6.286017	1.325147	1.809119
H	-6.480637	-2.214215	-0.664442	H	5.732073	2.194076	-0.641145
**C60_2_Prod**				**C60_3_Int**			
82				93			
C60_2_Prod_3 SCF Done: -2675.30722677 A.U.				C60_3_Int_3 SCF Done: -2869.48726635 A.U.			
C	-1.250177	3.044600	1.360477	C	-1.519379	-2.097840	1.779726
C	-0.868140	2.319868	2.551344	C	-1.220275	-2.802463	0.553127
C	-2.332604	2.586647	0.600154	C	-2.370731	-0.987850	1.738121
C	-1.590118	1.178450	2.917146	C	-1.791804	-2.360845	-0.645454
C	-0.885504	-0.000066	3.394706	C	-0.989710	-2.314928	-1.857523
C	0.582811	2.319397	2.630892	C	0.179019	-3.193148	0.588250
C	1.259822	1.178529	3.074547	C	0.954463	-3.128111	-0.574919
C	0.507193	-0.000087	3.471756	C	0.353239	-2.689881	-1.822759
C	-1.094253	3.043206	-1.488588	C	-0.904657	0.232225	3.309147
C	0.037958	3.497382	-0.695549	C	-0.006436	-0.911852	3.329469
C	-2.253295	2.585332	-0.851031	C	-2.059421	0.197295	2.518684
C	-0.037886	3.497359	0.695718	C	-0.306827	-2.049221	2.581968
C	1.094320	3.043092	1.488737	C	0.741298	-2.730720	1.837459
C	1.250250	3.044654	-1.360337	C	1.355324	-0.400642	3.365475
C	2.332668	2.586628	-0.600035	C	2.360822	-1.041297	2.631911
C	2.253351	2.585228	0.851152	C	2.047813	-2.228680	1.854230
C	-2.474199	0.741451	-2.419166	C	-1.720867	2.491194	1.786622

C	-1.259793	1.178730	-3.074497	C	-0.504053	2.538005	2.570434
C	-2.971031	1.437950	-1.338598	C	-2.492557	1.349087	1.774852
C	-0.582768	2.319547	-2.630792	C	-0.104188	1.435694	3.334064
C	0.868203	2.319971	-2.551220	C	1.294501	1.043842	3.369736
C	-0.507193	0.000087	-3.471756	C	0.486714	3.300381	1.828629
C	0.885504	0.000066	-3.394706	C	1.829711	2.924782	1.863695
C	1.590142	1.178553	-2.917076	C	2.243949	1.768809	2.641881
C	-3.776633	-0.805018	-0.203793	C	-3.087134	1.737565	-0.768504
C	-2.971022	-1.437778	-1.338622	C	-2.017909	2.820936	-0.659433
C	-3.776577	0.805104	-0.203751	C	-3.350817	0.914870	0.589908
C	-2.474201	-0.741250	-2.419189	C	-1.476403	3.247730	0.534587
C	-1.259822	-1.178529	-3.074547	C	-0.115800	3.740803	0.580843
C	-2.253352	-2.585229	-0.851152	C	-1.208095	2.838337	-1.847098
C	-1.094321	-3.043092	-1.488737	C	0.098533	3.340419	-1.830425
C	-0.582811	-2.319397	-2.630892	C	0.660153	3.803662	-0.581815
C	-2.726692	0.741495	2.133972	C	-2.695167	-1.231754	-0.657982
C	-2.726691	-0.741528	2.133924	C	-2.453375	-0.476635	-1.909461
C	-3.100575	1.441735	1.006908	C	-2.990799	-0.567459	0.511370
C	-3.100630	-1.441742	1.006856	C	-2.520840	0.899984	-1.915290
C	-2.332668	-2.586628	0.600035	C	-1.520513	1.651049	-2.625080
C	-1.590142	-1.178553	2.917076	C	-1.404017	-1.159219	-2.636706
C	-0.868204	-2.319972	2.551220	C	-0.453934	-0.433897	-3.363530
C	-1.250250	-3.044654	1.360337	C	-0.514932	1.010562	-3.359007
C	1.250176	-3.044601	-1.360477	C	2.358674	2.706381	-1.771046
C	0.037886	-3.497359	-0.695718	C	1.146590	2.659053	-2.574732
C	-0.037959	-3.497382	0.695549	C	0.846828	1.521613	-3.321488
C	2.332604	-2.586647	-0.600153	C	3.213000	1.598124	-1.731582
C	2.726691	-0.741495	-2.133972	C	3.538096	1.842905	0.664685
C	1.590118	-1.178451	-2.917146	C	2.632211	2.971938	0.652448
C	0.868140	-2.319869	-2.551344	C	2.058999	3.411709	-0.545329
C	3.100575	-1.441734	-1.006908	C	3.835422	1.177291	-0.504999
C	3.776632	0.805017	0.203793	C	3.927342	-1.129253	0.775454
C	3.100630	1.441741	-1.006856	C	3.361302	-0.290612	1.921682
C	2.726690	0.741528	-2.133924	C	3.294057	1.086139	1.916169
C	3.776576	-0.805104	0.203751	C	4.192754	-0.307191	-0.583288
C	2.474199	-0.741452	2.419167	C	2.561105	-1.881679	-1.779057
C	2.474200	0.741249	2.419188	C	2.316561	-2.639877	-0.527125
C	2.971021	1.437776	1.338621	C	2.857165	-2.211681	0.666626
C	2.971031	-1.437951	1.338598	C	3.332517	-0.739195	-1.767100
C	1.094253	-3.043206	1.488588	C	1.745527	0.377664	-3.301289
C	0.582768	-2.319547	2.630792	C	0.945153	-0.825515	-3.327286
C	1.259792	-1.178730	3.074497	C	1.344906	-1.927893	-2.563815
C	2.253294	-2.585333	0.851032	C	2.900649	0.412214	-2.511508
C	-5.331705	-1.123693	-0.311058	C	-4.909884	1.144664	0.804798
C	-5.331651	1.123753	-0.310909	C	-4.541888	2.295275	-1.090149
C	-5.785573	0.000094	-1.261009	C	-5.404572	1.096662	-0.652672
H	-5.265628	0.000079	-2.223872	H	-5.159457	0.164060	-1.169208
H	-6.866836	0.000083	-1.421749	H	-6.475561	1.300151	-0.737048
C	-5.957954	0.669844	0.994066	C	-4.871839	3.301937	-0.004567
H	-5.517241	2.155179	-0.613434	H	-4.628996	2.637898	-2.122057
C	-5.958474	-0.669918	0.993729	C	-5.092009	2.615943	1.125046
H	-6.228524	1.329201	1.810702	H	-4.807741	4.377698	-0.120889
H	-5.517345	-2.155066	-0.613747	H	-5.333645	0.430052	1.511644
H	-6.229440	-1.329413	1.810116	H	-5.244048	3.016589	2.120667
H	5.517344	2.155067	0.613752	H	6.177391	0.164292	-1.510270
C	5.331707	1.123694	0.311061	C	5.750444	-0.546265	-0.801290
H	6.229456	1.329419	-1.810106	H	6.070660	-2.421374	-2.116165
C	5.958484	0.669922	-0.993723	C	5.922736	-2.019220	-1.120557
C	5.785578	-0.000095	1.261008	C	6.249695	-0.501252	0.654768

H	5.265636	-0.000080	2.223872	H	6.012653	0.432771	1.173267
H	6.866841	-0.000085	1.421744	H	7.319361	-0.711360	0.736488
C	5.957947	-0.669840	-0.994071	C	5.702012	-2.702968	0.010241
C	5.331651	-1.123752	0.310907	C	5.380093	-1.693264	1.095599
H	6.228510	-1.329194	-1.810711	H	5.632659	-3.778244	0.127841
H	5.517242	-2.155179	0.613428	H	5.467025	-2.035421	2.127678
				C	-5.267614	-3.731971	-1.227967
				C	-5.784602	-2.755492	-0.453947
				C	-5.314818	-2.930453	0.927208
				C	-4.416633	-4.636578	-0.375861
				H	-5.426062	-3.873910	-2.290403
				C	-4.517313	-4.016428	0.992745
				H	-3.376036	-4.680572	-0.726671
				H	-4.787049	-5.673165	-0.385430
				H	-6.443313	-1.957519	-0.779897
				H	-5.574726	-2.274613	1.751170
				H	-4.008502	-4.398438	1.869199
**C60_3_TS**				**C60_3_Prod**			
93				93			
C60_3_TS_3 SCF Done: -2869.46189621 A.U.				C60_3_Prod_3 SCF Done: -2869.50036897 A.U.			
C	1.466073	2.199267	1.752354	C	1.442646	2.268227	1.732466
C	1.148291	2.998837	0.545748	C	1.151780	3.189087	0.554835
C	2.423203	1.195833	1.722717	C	2.397767	1.273853	1.694758
C	1.779373	2.577308	-0.710231	C	1.858277	2.734861	-0.818105
C	0.905050	2.327930	-1.874570	C	0.883965	2.335358	-1.921136
C	-0.333307	3.109434	0.561474	C	-0.376762	3.136168	0.528398
C	-1.102447	2.963575	-0.588717	C	-1.130367	2.952783	-0.615286
C	-0.459442	2.574021	-1.847117	C	-0.475500	2.550214	-1.877491
C	1.091296	-0.161668	3.303925	C	1.093561	-0.090686	3.294834
C	0.087558	0.878582	3.323048	C	0.078358	0.933366	3.307679
C	2.233645	-0.009091	2.501675	C	2.227120	0.072419	2.481606
C	0.272680	2.022228	2.544811	C	0.251848	2.068118	2.512559
C	-0.839153	2.589361	1.800432	C	-0.868951	2.609678	1.760326
C	-1.220188	0.235387	3.371209	C	-1.220573	0.272922	3.367581
C	-2.285860	0.765314	2.636817	C	-2.293855	0.778720	2.629131
C	-2.095921	1.969526	1.840928	C	-2.119579	1.973845	1.816528
C	2.124374	-2.335492	1.791530	C	2.155753	-2.267593	1.808926
C	0.922600	-2.500829	2.583731	C	0.958608	-2.440597	2.606125
C	2.771622	-1.118914	1.761092	C	2.777955	-1.039082	1.755152
C	0.417416	-1.440549	3.343475	C	0.440045	-1.378913	3.355451
C	-1.014393	-1.194033	3.386806	C	-0.994890	-1.152966	3.400119
C	0.011846	-3.367445	1.856470	C	0.060178	-3.329997	1.893630
C	-1.363156	-3.129916	1.895959	C	-1.318417	-3.110587	1.932501
C	-1.890155	-2.018387	2.668638	C	-1.860480	-1.998651	2.693284
C	3.402822	-1.484366	-0.775105	C	3.422575	-1.437469	-0.768827
C	2.446941	-2.667215	-0.650898	C	2.483892	-2.632371	-0.627684
C	3.585023	-0.617967	0.572719	C	3.582809	-0.544071	0.562446
C	1.953166	-3.128638	0.550597	C	1.996384	-3.082571	0.580885
C	0.649758	-3.755859	0.609104	C	0.701934	-3.726187	0.650027
C	1.636941	-2.777696	-1.833763	C	1.673765	-2.769936	-1.807230
C	0.386133	-3.405286	-1.804530	C	0.430706	-3.412515	-1.767401
C	-0.120692	-3.909523	-0.548881	C	-0.067873	-3.906269	-0.504528
C	2.716378	1.433559	-0.682132	C	2.718968	1.478807	-0.721797
C	2.533412	0.640291	-1.914032	C	2.525981	0.659100	-1.928365
C	3.091447	0.825971	0.477312	C	3.072761	0.892639	0.442736
C	2.750560	-0.721128	-1.924674	C	2.761152	-0.700192	-1.927753
C	1.825383	-1.571500	-2.623207	C	1.844760	-1.570658	-2.611476
C	1.420154	1.209238	-2.637483	C	1.401143	1.205292	-2.650978
C	0.550421	0.388985	-3.365850	C	0.544173	0.361632	-3.372405

C	0.757934	-1.041644	-3.357573	C	0.770119	-1.065459	-3.351411
C	-1.925460	-2.997198	-1.738949	C	-1.885415	-3.031944	-1.703529
C	-0.728890	-2.837961	-2.549391	C	-0.692990	-2.869576	-2.518173
C	-0.546525	-1.683246	-3.308148	C	-0.525850	-1.722488	-3.292527
C	-2.886000	-1.978300	-1.704319	C	-2.858903	-2.024143	-1.680069
C	-3.176356	-2.235662	0.696051	C	-3.144491	-2.255810	0.723823
C	-2.160992	-3.267929	0.688349	C	-2.114845	-3.274248	0.727317
C	-1.551713	-3.658035	-0.508444	C	-1.502044	-3.671957	-0.464957
C	-3.542143	-1.612136	-0.476799	C	-3.518631	-1.651011	-0.455816
C	-3.859740	0.683031	0.786426	C	-3.866171	0.654352	0.780421
C	-3.209702	-0.086346	1.937840	C	-3.207286	-0.093379	1.941415
C	-3.005233	-1.450087	1.942489	C	-2.983882	-1.454037	1.961553
C	-4.044158	-0.171008	-0.565008	C	-4.038543	-0.217548	-0.560938
C	-2.575327	1.547053	-1.777017	C	-2.582836	1.500307	-1.788134
C	-2.403331	2.334343	-0.534334	C	-2.420516	2.303914	-0.557519
C	-2.903733	1.866571	0.665311	C	-2.923896	1.847631	0.646260
C	-3.233073	0.334937	-1.754661	C	-3.231674	0.284125	-1.755119
C	-1.553846	-0.632767	-3.291538	C	-1.545612	-0.684072	-3.286154
C	-0.876674	0.641963	-3.330561	C	-0.880943	0.597039	-3.336023
C	-1.372764	1.707591	-2.568316	C	-1.382323	1.664684	-2.580013
C	-2.696570	-0.775026	-2.494050	C	-2.684004	-0.829115	-2.483205
C	5.158275	-0.700270	0.792632	C	5.157624	-0.591476	0.789155
C	4.904782	-1.902317	-1.089129	C	4.932561	-1.835749	-1.068547
C	5.649839	-0.623120	-0.664385	C	5.653249	-0.535147	-0.667131
H	5.316524	0.275577	-1.192536	H	5.306805	0.346963	-1.214160
H	6.735357	-0.724410	-0.746741	H	6.740686	-0.618969	-0.743064
C	5.327485	-2.860468	0.008221	C	5.367866	-2.763641	0.049536
H	5.026547	-2.246780	-2.116982	H	5.064243	-2.198700	-2.088766
C	5.478545	-2.143741	1.130157	C	5.501980	-2.021796	1.157309
H	5.364510	-3.938867	-0.094946	H	5.424960	-3.843060	-0.031379
H	5.512132	0.058711	1.491909	H	5.494731	0.188028	1.473965
H	5.664567	-2.517381	2.130510	H	5.691268	-2.371720	2.165589
H	-5.972966	-0.844289	-1.485304	H	-5.957828	-0.924428	-1.475968
C	-5.618118	-0.089974	-0.781751	C	-5.612975	-0.157884	-0.780715
H	-6.124699	1.735097	-2.108466	H	-6.139447	1.645447	-2.129360
C	-5.936974	1.356256	-1.110385	C	-5.948242	1.280653	-1.126709
C	-6.108334	-0.174763	0.675583	C	-6.104160	-0.231501	0.676866
H	-5.778043	-1.076982	1.199329	H	-5.764100	-1.123644	1.211554
H	-7.193575	-0.071888	0.758475	H	-7.190625	-0.140361	0.757207
C	-5.783635	2.066036	0.015505	C	-5.804843	2.005205	-0.008977
C	-5.360655	1.100604	1.106580	C	-5.372074	1.057503	1.093902
H	-5.820423	3.143738	0.125713	H	-5.854865	3.083556	0.088841
H	-5.479723	1.439448	2.136595	H	-5.496667	1.407032	2.119663
C	2.914257	4.376756	-1.214401	C	2.694588	4.036734	-1.191724
C	3.874137	4.326984	-0.183578	C	3.784214	4.168219	-0.144543
C	3.266266	4.683376	1.020449	C	3.196613	4.543318	0.999558
C	1.777885	5.230725	-0.703436	C	1.718920	5.138700	-0.739186
H	3.135829	4.249505	-2.267934	H	3.012590	4.033124	-2.235330
C	1.898829	4.956027	0.780462	C	1.709392	4.667097	0.726835
H	0.799841	5.029190	-1.143643	H	0.739735	5.085853	-1.224886
H	2.021447	6.289612	-0.885567	H	2.138383	6.141749	-0.853787
H	4.876765	3.927291	-0.283831	H	4.819006	3.880566	-0.290534
H	3.722655	4.604959	2.000462	H	3.653049	4.625505	1.979344
H	1.230421	5.391071	1.515327	H	1.124500	5.243390	1.444932
**C60_4_Int**				**C60_4_TS**			
104				104			
C60_4_Int_3 SCF Done: -3063.68153752 A.U.				C60_4_TS_3 SCF Done: -3063.65431622 A.U.			
C	2.638214	-1.887855	-1.634903	C	-2.644655	1.663283	-1.761507
C	2.626662	-2.816895	-0.428143	C	-2.791729	2.631477	-0.594899

C	3.216813	-0.635925	-1.617652	C	-3.073511	0.352465	-1.712759
C	3.105529	-2.115835	0.939278	C	-3.243235	1.931407	0.781871
C	2.021438	-2.022018	2.007796	C	-2.202001	2.005092	1.893984
C	1.163777	-3.263832	-0.432251	C	-1.391774	3.247470	-0.563559
C	0.357316	-3.301190	0.689339	C	-0.641729	3.418507	0.584619
C	0.807443	-2.669119	1.947138	C	-1.067935	2.786702	1.850558
C	1.589108	0.180097	-3.293361	C	-1.294092	-0.327030	-3.291939
C	0.962356	-1.118735	-3.285609	C	-0.824784	1.037245	-3.316460
C	2.689035	0.419857	-2.453337	C	-2.392190	-0.663914	-2.482384
C	1.471069	-2.110307	-2.444187	C	-1.480225	1.992222	-2.535722
C	0.564721	-2.964039	-1.692503	C	-0.710681	2.975207	-1.787592
C	-0.478272	-0.919545	-3.395228	C	0.631641	1.007395	-3.364337
C	-1.351022	-1.724582	-2.657843	C	1.373848	1.936260	-2.629428
C	-0.822312	-2.772271	-1.796505	C	0.692348	2.947057	-1.833413
C	1.841533	2.628523	-1.873208	C	-1.310038	-2.729634	-1.772918
C	0.677674	2.377973	-2.698756	C	-0.150233	-2.374186	-2.565499
C	2.826948	1.671782	-1.760360	C	-2.407283	-1.894891	-1.740266
C	0.554767	1.182823	-3.415828	C	-0.145791	-1.202679	-3.332978
C	-0.727028	0.500400	-3.478345	C	1.044819	-0.376177	-3.371723
C	-0.481884	2.947743	-2.037821	C	1.041083	-2.780062	-1.847390
C	-1.712715	2.289521	-2.093132	C	2.177223	-1.982842	-1.874207
C	-1.841411	1.039110	-2.821063	C	2.179602	-0.755145	-2.640055
C	3.230630	2.334944	0.758004	C	-2.843257	-2.506445	0.785548
C	1.959676	3.154435	0.553162	C	-1.477872	-3.178286	0.665231
C	3.712350	1.502362	-0.534455	C	-3.359952	-1.785882	-0.558384
C	1.389212	3.384374	-0.680771	C	-0.824670	-3.381034	-0.535185
C	-0.041398	3.569267	-0.799166	C	0.619793	-3.404084	-0.589188
C	1.113252	3.056186	1.711215	C	-0.701389	-2.933468	1.841300
C	-0.269143	3.257359	1.623670	C	0.699743	-2.961211	1.800641
C	-0.862649	3.522152	0.333132	C	1.378450	-3.216294	0.562113
C	3.513864	-0.651473	0.810583	C	-3.473867	0.425474	0.700346
C	3.029012	0.097028	1.980929	C	-2.957809	-0.214842	1.920646
C	3.693096	-0.017780	-0.368891	C	-3.526696	-0.269253	-0.456946
C	2.809770	1.458119	1.932047	C	-2.579424	-1.542161	1.937885
C	1.640108	2.003180	2.564354	C	-1.382095	-1.920007	2.635332
C	2.121128	-0.763352	2.702864	C	-2.187691	0.772915	2.639029
C	1.015082	-0.223242	3.374886	C	-1.054515	0.391089	3.375065
C	0.765812	1.198328	3.302976	C	-0.640040	-0.991730	3.371825
C	-2.334183	2.141791	1.531051	C	2.622903	-1.631565	1.755416
C	-1.178304	2.400898	2.373288	C	1.468493	-1.977097	2.546661
C	-0.671047	1.395374	3.195025	C	0.815783	-1.019745	3.326129
C	-2.926389	0.871523	1.527754	C	3.065943	-0.315800	1.725919
C	-3.198437	0.923681	-0.886507	C	3.440263	-0.409349	-0.678834
C	-2.554542	2.221047	-0.910323	C	3.072440	-1.841905	-0.706949
C	-2.141060	2.833903	0.276182	C	2.674697	-2.489490	0.548253
C	-3.393096	0.266162	0.307430	C	3.518008	0.300677	0.480035
C	-2.933352	-2.063102	-0.845042	C	2.831350	2.525313	-0.775946
C	-2.519012	-1.177543	-2.021879	C	2.569860	1.557335	-1.928413
C	-2.748923	0.180766	-2.089570	C	2.947214	0.230506	-1.915443
C	-3.420571	-1.254782	0.458420	C	3.352758	1.817522	0.574134
C	-1.522976	-2.365900	1.777891	C	1.287429	2.746570	1.776364
C	-1.071253	-3.109936	0.582492	C	0.802526	3.391945	0.537876
C	-1.658903	-2.879211	-0.647242	C	1.463813	3.193452	-0.659487
C	-2.529944	-1.428716	1.685148	C	2.395488	1.926158	1.756163
C	-1.297642	0.081902	3.209573	C	1.283975	0.348763	3.305412
C	-0.254554	-0.910880	3.321177	C	0.130123	1.218112	3.336371
C	-0.358752	-2.106220	2.597905	C	0.127004	2.386621	2.564671
C	-2.396468	-0.175756	2.379624	C	2.382705	0.694528	2.501135
C	5.192553	2.052724	-0.733431	C	-4.755507	-2.514303	-0.793903

C	4.519972	3.211744	1.070506	C	-4.034625	-3.516013	1.083659
C	5.635538	2.204936	0.732944	C	-5.243883	-2.662225	0.658380
H	5.577858	1.275144	1.307001	H	-5.320763	-1.710863	1.193470
H	6.634116	2.640333	0.825814	H	-6.188192	-3.208836	0.729410
C	4.663958	4.196426	-0.074057	C	-4.011205	-4.553822	-0.022288
H	4.495803	3.628394	2.078356	H	-4.007624	-3.888466	2.108640
C	5.065005	3.505396	-1.149771	C	-4.440932	-3.956453	-1.142094
H	4.364779	5.237513	-0.033769	H	-3.593919	-5.549593	0.074631
H	5.784845	1.404944	-1.381365	H	-5.389073	-1.966022	-1.492693
H	5.160479	3.867177	-2.167070	H	-4.446416	-4.364876	-2.146152
H	-5.488983	-1.186350	1.307465	H	5.377435	2.019918	1.510597
C	-4.893155	-1.822395	0.654666	C	4.741515	2.557646	0.805757
H	-4.836521	-3.658836	2.058592	H	4.419117	4.418239	2.140978
C	-4.747835	-3.280142	1.046729	C	4.417687	4.001078	1.140489
C	-5.339090	-1.955947	-0.812427	C	5.231391	2.695570	-0.647287
H	-5.294605	-1.016511	-1.370489	H	5.314049	1.739977	-1.174276
H	-6.334249	-2.398828	-0.908466	H	6.172599	3.247009	-0.722341
C	-4.345994	-3.951063	-0.041233	C	3.986337	4.585945	0.014852
C	-4.215668	-2.946148	-1.170185	C	4.017721	3.538254	-1.081847
H	-4.039227	-4.989054	-0.100748	H	3.563175	5.578201	-0.091965
H	-4.189529	-3.346338	-2.184682	H	3.990255	3.901672	-2.110049
C	4.307973	-3.062239	1.377961	C	-4.564292	2.746018	1.135017
C	5.411921	-2.863035	0.356799	C	-5.594122	2.380293	0.082953
C	5.012752	-3.443840	-0.782625	C	-5.217473	2.960354	-1.064477
C	3.757326	-4.435126	0.950243	C	-4.158942	4.156911	0.670206
H	4.576210	-2.923600	2.426336	H	-4.858218	2.616462	2.177649
C	3.639046	-4.037137	-0.532722	C	-3.933282	3.719598	-0.789077
H	2.800008	-4.689440	1.415402	H	-3.257625	4.538812	1.159354
H	4.476343	-5.242818	1.111066	H	-4.972688	4.880274	0.769786
H	6.291953	-2.249194	0.510453	H	-6.402218	1.672885	0.229476
H	5.500058	-3.402182	-1.750035	H	-5.655049	2.824201	-2.046882
H	3.294875	-4.794043	-1.238790	H	-3.649621	4.484324	-1.513427
C	-6.972460	1.092588	0.113099	C	4.857089	-2.993227	-1.207780
C	-6.536298	1.538416	1.310318	C	5.707211	-2.544779	-0.176488
H	-6.715186	1.072396	2.273917	H	6.447504	-1.759182	-0.276049
H	-7.567688	0.207106	-0.075298	H	5.005351	-2.784442	-2.261196
C	-5.770983	2.777712	1.120066	C	5.305819	-3.124669	1.027039
C	-5.743724	3.084931	-0.192854	C	4.180502	-3.947712	0.786274
H	-5.246064	3.929466	-0.652730	H	3.756561	-4.623590	1.520814
H	-5.297344	3.333906	1.920846	H	5.685554	-2.860791	2.007393
H	-5.866415	1.536871	-1.702169	H	3.216912	-4.478740	-1.138548
C	-6.506558	2.038839	-0.961793	C	4.188144	-4.248041	-0.697670
H	-7.348082	2.468691	-1.525746	H	4.857001	-5.104458	-0.879253
**C60_4_Prod**				**C60_5_Int**			
104				115			
C60_4_Prod_3 SCF Done: -3063.69275352 A.U.				C60_5_Int_3 SCF Done: -3257.87467349 A.U.			
C	2.600985	1.729130	1.748783	C	2.536555	1.728221	0.912778
C	2.721582	2.693360	0.575813	C	2.400332	2.695240	-0.255392
C	3.061354	0.428093	1.706014	C	2.971405	0.427506	0.765191
C	3.186484	1.996416	-0.798079	C	2.557820	2.001532	-1.699573
C	2.140937	2.037228	-1.907982	C	1.296337	2.043174	-2.556493
C	1.307077	3.275296	0.545224	C	1.012309	3.275809	0.022706
C	0.549266	3.419791	-0.601689	C	0.023965	3.421049	-0.932317
C	0.987516	2.790875	-1.865238	C	0.178612	2.794570	-2.262085
C	1.303840	-0.283166	3.294716	C	1.608636	-0.288040	2.699007
C	0.802624	1.069759	3.314246	C	1.123681	1.065229	2.832693
C	2.405616	-0.599024	2.482102	C	2.501609	-0.600483	1.661454
C	1.432821	2.035420	2.525082	C	1.565782	2.032284	1.926275
C	0.636682	2.994637	1.772958	C	0.624475	2.991471	1.366548

C	-0.652042	1.005232	3.365915	C	-0.285716	0.997658	3.196749
C	-1.419016	1.909932	2.627076	C	-1.196039	1.902782	2.644230
C	-0.765206	2.933539	1.824020	C	-0.732970	2.928332	1.720232
C	1.364682	-2.688076	1.780157	C	1.342254	-2.693176	1.203587
C	0.198752	-2.354683	2.571303	C	0.375593	-2.361208	2.229085
C	2.445887	-1.833154	1.744837	C	2.386805	-1.833779	0.934187
C	0.174301	-1.183272	3.338356	C	0.516929	-1.190471	2.985796
C	-1.031226	-0.387292	3.377963	C	-0.652220	-0.395992	3.287812
C	-0.987515	-2.790876	1.865238	C	-0.935423	-2.797218	1.796028
C	-2.140936	-2.037228	1.907981	C	-2.052566	-2.044793	2.089499
C	-2.156931	-0.802099	2.647132	C	-1.909080	-0.811021	2.817320
C	2.896125	-2.449896	-0.776638	C	2.283177	-2.446302	-1.624626
C	1.546575	-3.152687	-0.651400	C	0.993359	-3.151575	-1.211327
C	3.395850	-1.709178	0.562426	C	3.058378	-1.705538	-0.425120
C	0.893514	-3.357547	0.549585	C	0.616066	-3.360111	0.102519
C	-0.549265	-3.419793	0.601689	C	-0.781130	-3.423763	0.466288
C	0.765206	-2.933540	-1.824019	C	-0.024141	-2.930086	-2.185581
C	-0.636683	-2.994638	-1.772958	C	-1.381608	-2.993159	-1.831912
C	-1.307076	-3.275295	-0.545224	C	-1.769605	-3.277089	-0.488776
C	3.454503	0.497170	-0.708737	C	2.840452	0.502366	-1.675403
C	2.953066	-0.162380	-1.925022	C	2.087284	-0.156016	-2.754494
C	3.526249	-0.189642	0.452200	C	3.159114	-0.186341	-0.558106
C	2.608465	-1.498968	-1.934701	C	1.750394	-1.493582	-2.691630
C	1.419015	-1.909932	-2.627075	C	0.439030	-1.904412	-3.109602
C	2.156931	0.802098	-2.647132	C	1.152415	0.809025	-3.284058
C	1.031225	0.387291	-3.377962	C	-0.104757	0.393859	-3.753573
C	0.652042	-1.005233	-3.365915	C	-0.470842	-0.999297	-3.662483
C	-2.600984	-1.729128	-1.748782	C	-3.295193	-1.730347	-1.379757
C	-1.432822	-2.035420	-2.525081	C	-2.322791	-2.033548	-2.391424
C	-0.802626	-1.069759	-3.314246	C	-1.879701	-1.065754	-3.296508
C	-3.061354	-0.428092	-1.706015	C	-3.736799	-0.430088	-1.235394
C	-3.454501	-0.497168	0.708736	C	-3.596534	-0.504120	1.207032
C	-3.186480	-1.996415	0.798075	C	-3.314005	-2.003176	1.232866
C	-2.721581	-2.693355	-0.575809	C	-3.157481	-2.696870	-0.210594
C	-3.526249	0.189643	-0.452201	C	-3.919195	0.184674	0.090709
C	-2.896126	2.449896	0.776638	C	-3.039594	2.443283	1.158355
C	-2.608466	1.498968	1.934701	C	-2.506651	1.490674	2.224628
C	-2.953066	0.162380	1.925022	C	-2.843848	0.153754	2.287034
C	-3.395851	1.709178	-0.562428	C	-3.817139	1.704566	-0.041978
C	-1.364682	2.688075	-1.780157	C	-2.099288	2.688351	-1.669033
C	-0.893514	3.357545	-0.549585	C	-1.373292	3.356350	-0.568502
C	-1.546575	3.152686	0.651399	C	-1.750187	3.148540	0.745048
C	-2.445888	1.833155	-1.744839	C	-3.146401	1.832047	-1.402042
C	-1.303840	0.283165	-3.294717	C	-2.366181	0.286370	-3.165941
C	-0.174301	1.183272	-3.338356	C	-1.273740	1.188037	-3.451467
C	-0.198752	2.354682	-2.571303	C	-1.132240	2.357894	-2.694769
C	-2.405618	0.599024	-2.482104	C	-3.265824	0.599310	-2.133049
C	4.807986	-2.403135	0.802245	C	4.492454	-2.391025	-0.496534
C	4.110412	-3.432723	-1.068961	C	3.408853	-3.426392	-2.171559
C	5.299651	-2.548490	-0.649136	C	4.657644	-2.537471	-2.019695
H	5.354423	-1.598851	-1.190033	H	4.590534	-1.588654	-2.560696
H	6.256411	-3.073391	-0.716941	H	5.578739	-3.059389	-2.293364
C	4.110816	-4.464115	0.043407	C	3.654209	-4.455489	-1.083949
H	4.092081	-3.812280	-2.091523	H	3.170977	-3.808334	-3.165367
C	4.527163	-3.850245	1.159259	C	4.300726	-3.837789	-0.085725
H	3.716968	-5.469936	-0.047744	H	3.252684	-5.462443	-1.085758
H	5.428620	-1.836076	1.497655	H	5.239121	-1.817446	0.052027
H	4.543010	-4.252353	2.165746	H	4.533644	-4.237038	0.894912
H	-5.428620	1.836077	-1.497656	H	-6.004423	1.830267	-0.513927

C	-4.807986	2.403135	-0.802245	C	-5.248404	2.396948	0.031656
H	-4.543008	4.252351	-2.165746	H	-5.286870	4.249302	-1.352721
C	-4.527162	3.850244	-1.159259	C	-5.053189	3.845124	-0.374443
C	-5.299651	2.548490	0.649137	C	-5.413693	2.538562	1.555534
H	-5.354424	1.598850	1.190033	H	-5.348729	1.587720	2.093182
H	-6.256410	3.073391	0.716943	H	-6.333499	3.061874	1.830592
C	-4.110819	4.464115	-0.043407	C	-4.405782	4.457420	0.626280
C	-4.110413	3.432722	1.068962	C	-4.162711	3.423737	1.709507
H	-3.716972	5.469937	0.047747	H	-4.002609	5.463655	0.631933
H	-4.092085	3.812276	2.091525	H	-3.923329	3.800961	2.704708
C	4.486169	2.841273	-1.159677	C	3.748990	2.847456	-2.329696
C	5.527694	2.506992	-0.108613	C	4.993009	2.512319	-1.527794
C	5.140178	3.084170	1.036615	C	4.861656	3.088881	-0.325642
C	4.047743	4.244419	-0.701627	C	3.419505	4.249917	-1.785605
H	4.780224	2.712960	-2.202389	H	3.813074	2.721227	-3.411439
C	3.836959	3.810025	0.760731	C	3.528281	3.812593	-0.312806
H	3.135967	4.601413	-1.190227	H	2.423790	4.607036	-2.065834
H	4.843281	4.986862	-0.807581	H	4.173347	4.993046	-2.059142
H	6.352688	1.819071	-0.253633	H	5.768541	1.826378	-1.848734
H	5.583895	2.964731	2.018433	H	5.511123	2.974971	0.534392
H	3.536560	4.571629	1.481574	H	3.389419	4.572657	0.457318
C	-4.486169	-2.841272	1.159677	C	-4.503494	-2.850393	1.866012
C	-5.527693	-2.506991	0.108614	C	-5.748438	-2.515635	1.066463
H	-6.352686	-1.819069	0.253631	H	-6.522871	-1.828841	1.388145
H	-4.780220	-2.712957	2.202390	H	-4.564536	-2.724159	2.947948
C	-5.140177	-3.084169	-1.036614	C	-5.617811	-3.090182	-0.136763
C	-3.836960	-3.810025	-0.760731	C	-4.285155	-3.814811	-0.151443
H	-3.536557	-4.571626	-1.481575	H	-4.147488	-4.574758	-0.921809
H	-5.583896	-2.964728	-2.018431	H	-6.263830	-2.969389	-0.998850
H	-3.135965	-4.601412	1.190227	H	-3.177228	-4.608890	1.599647
C	-4.047742	-4.244419	0.701627	C	-4.173503	-4.252117	1.320992
H	-4.843280	-4.986862	0.807582	H	-4.926530	-4.995787	1.595323
				C	4.590307	0.493599	4.116477
				C	5.017655	-0.942338	3.963434
				H	4.152619	-1.610240	3.843739
				H	5.569545	-1.306934	4.842937
				C	5.877398	-0.915060	2.726861
				C	5.926924	0.347229	2.251687
				H	6.459730	0.680506	1.367343
				C	5.126846	1.223852	3.117472
				H	6.372229	-1.786584	2.314495
				H	3.939588	0.849865	4.905425
				H	4.986045	2.286204	2.953370
**C60_5_TS**				**C60_5_Prod**			
115				115			
C60_5_TS_3 SCF Done: -3257.84356095 A.U.				C60_5_Prod_3 SCF Done: -3257.87826462 A.U.			
C	2.552958	1.774281	1.090475	C	2.551343	1.750178	1.074819
C	2.397264	2.828410	0.000961	C	2.416577	2.779293	-0.039553
C	3.023420	0.514069	0.848244	C	3.043646	0.503161	0.878445
C	2.613825	2.271734	-1.497009	C	2.652694	2.195539	-1.520697
C	1.373464	2.340007	-2.382476	C	1.426152	2.253715	-2.425777
C	0.979718	3.325870	0.286359	C	1.004706	3.298513	0.214091
C	0.011314	3.521362	-0.679509	C	0.052284	3.484838	-0.768236
C	0.220390	3.019176	-2.053501	C	0.274185	2.948673	-2.127393
C	1.736245	-0.427340	2.819540	C	1.752425	-0.375688	3.009060
C	1.100433	0.914813	2.905112	C	1.076688	0.986104	2.924120
C	2.675710	-0.624104	1.703973	C	2.858561	-0.639612	1.864752
C	1.527065	1.959807	2.108068	C	1.517664	1.986537	2.090739
C	0.568571	2.919049	1.592270	C	0.575987	2.933629	1.526251

C	-0.304932	0.762974	3.196224	C	-0.333401	0.850514	3.179080
C	-1.244837	1.681422	2.717452	C	-1.263681	1.759234	2.662233
C	-0.797866	2.788763	1.899004	C	-0.796575	2.834325	1.818659
C	1.515883	-2.677199	0.975880	C	1.500991	-2.624889	1.066756
C	0.503332	-2.463893	2.007747	C	0.467274	-2.393561	2.095893
C	2.520756	-1.762838	0.812206	C	2.540754	-1.777645	0.905819
C	0.583412	-1.367684	2.858541	C	0.538067	-1.294715	2.925634
C	-0.617091	-0.645031	3.165000	C	-0.656236	-0.557041	3.180833
C	-0.774933	-2.909968	1.499568	C	-0.800959	-2.848024	1.578084
C	-1.935177	-2.234922	1.823232	C	-1.969578	-2.170420	1.871257
C	-1.854040	-1.063900	2.643086	C	-1.892432	-0.980108	2.654686
C	2.504120	-2.169624	-1.793330	C	2.518104	-2.242362	-1.698885
C	1.235197	-2.953752	-1.475900	C	1.239218	-2.999021	-1.372155
C	3.212659	-1.498926	-0.516062	C	3.227675	-1.546749	-0.432096
C	0.838130	-3.275143	-0.190114	C	0.826376	-3.264136	-0.081587
C	-0.563475	-3.419660	0.130197	C	-0.575959	-3.396140	0.227910
C	0.235164	-2.691443	-2.454786	C	0.253212	-2.767205	-2.372319
C	-1.128369	-2.832183	-2.143257	C	-1.114604	-2.886990	-2.068521
C	-1.535033	-3.236214	-0.837127	C	-1.538087	-3.248626	-0.754771
C	2.948761	0.789334	-1.589335	C	2.976607	0.710978	-1.569916
C	2.251323	0.194778	-2.740331	C	2.291338	0.093736	-2.712544
C	3.248807	0.023959	-0.517649	C	3.253139	-0.025513	-0.468213
C	1.964701	-1.154474	-2.797546	C	1.997869	-1.254188	-2.739504
C	0.680512	-1.577428	-3.280571	C	0.716409	-1.681325	-3.225217
C	1.293202	1.166183	-3.213786	C	1.346472	1.059001	-3.226170
C	0.064279	0.745589	-3.748213	C	0.120959	0.632581	-3.763037
C	-0.250634	-0.663211	-3.781102	C	-0.203720	-0.774863	-3.760819
C	-3.100627	-1.682660	-1.643314	C	-3.085368	-1.713551	-1.625222
C	-2.090801	-1.866387	-2.648730	C	-2.064554	-1.933039	-2.613666
C	-1.663213	-0.811059	-3.459565	C	-1.619457	-0.903668	-3.449229
C	-3.595333	-0.416455	-1.406161	C	-3.574740	-0.438622	-1.430939
C	-3.516301	-0.684992	1.025292	C	-3.532049	-0.637791	1.008538
C	-3.175578	-2.170434	0.937152	C	-3.198084	-2.127270	0.967200
C	-2.952706	-2.735308	-0.552815	C	-2.957407	-2.734802	-0.503225
C	-3.837638	0.080819	-0.040211	C	-3.832228	0.099472	-0.082856
C	-3.071559	2.274709	1.233861	C	-3.066561	2.323868	1.139184
C	-2.532161	1.256068	2.233394	C	-2.549083	1.330111	2.174738
C	-2.818362	-0.089894	2.176975	C	-2.845473	-0.013694	2.152438
C	-3.790113	1.609504	-0.045700	C	-3.773439	1.627055	-0.131315
C	-2.065835	2.783164	-1.534914	C	-2.019797	2.744407	-1.630029
C	-1.393622	3.381322	-0.363430	C	-1.358003	3.367752	-0.467008
C	-1.798023	3.056032	0.916138	C	-1.781910	3.084455	0.816026
C	-3.088284	1.871901	-1.370278	C	-3.052139	1.846031	-1.453411
C	-2.204595	0.507147	-3.232014	C	-2.154962	0.423952	-3.265312
C	-1.141013	1.468586	-3.412568	C	-1.082757	1.372731	-3.460535
C	-1.062150	2.574642	-2.557201	C	-1.005598	2.501042	-2.634436
C	-3.140584	0.700600	-2.202445	C	-3.101433	0.652434	-2.252699
C	4.679319	-2.115817	-0.607109	C	4.697359	-2.165374	-0.520979
C	3.681264	-3.058567	-2.384418	C	3.686547	-3.159657	-2.262456
C	4.885701	-2.133374	-2.131770	C	4.893979	-2.230637	-2.044881
H	4.788717	-1.148011	-2.597176	H	4.797864	-1.260059	-2.540373
H	5.835497	-2.592132	-2.420176	H	5.841159	-2.700937	-2.323115
C	3.946012	-4.159091	-1.374573	C	3.951361	-4.228235	-1.219175
H	3.482863	-3.370321	-3.410736	H	3.482324	-3.502969	-3.277507
C	4.544101	-3.597363	-0.314893	C	4.558874	-3.636419	-0.181427
H	3.586780	-5.177935	-1.463998	H	3.587495	-5.247887	-1.274599
H	5.397817	-1.554119	-0.008987	H	5.426081	-1.589514	0.050043
H	4.770604	-4.065661	0.636412	H	4.788998	-4.074441	0.783266
H	-5.966918	1.697987	-0.568664	H	-5.942191	1.716969	-0.685661

C	-5.246592	2.243739	0.042661	C	-5.225660	2.274669	-0.080692
H	-5.316854	4.201026	-1.187692	H	-5.264323	4.195951	-1.367980
C	-5.094882	3.726357	-0.238762	C	-5.058468	3.747365	-0.402859
C	-5.456960	2.253930	1.567629	C	-5.456312	2.330804	1.440348
H	-5.369770	1.265054	2.027962	H	-5.383281	1.355113	1.930309
H	-6.402748	2.720044	1.857373	H	-6.402114	2.812367	1.703503
C	-4.497383	4.276757	0.826845	C	-4.470804	4.323902	0.654329
C	-4.244443	3.166791	1.829179	C	-4.240197	3.241659	1.691843
H	-4.131398	5.292391	0.925243	H	-4.098441	5.339272	0.728363
H	-4.045665	3.469234	2.858329	H	-4.053109	3.572563	2.714395
C	3.784479	3.213512	-2.018713	C	3.839640	3.117973	-2.040923
C	5.021116	2.855329	-1.215777	C	5.061331	2.761468	-1.214777
C	4.838656	3.318811	0.027899	C	4.865485	3.246037	0.018883
C	3.386974	4.549475	-1.364405	C	3.445980	4.467699	-1.413524
H	3.878820	3.185454	-3.105283	H	3.948858	3.071069	-3.125420
C	3.478532	3.990029	0.067128	C	3.511430	3.929899	0.027809
H	2.384455	4.891662	-1.638905	H	2.451025	4.815611	-1.707724
H	4.116774	5.341533	-1.553039	H	4.186563	5.249339	-1.603281
H	5.828729	2.226767	-1.573239	H	5.868578	2.120751	-1.550857
H	5.467090	3.146389	0.894400	H	5.480547	3.082981	0.896630
H	3.292446	4.674968	0.895638	H	3.319139	4.628995	0.842905
C	-4.345485	-3.111404	1.462788	C	-4.378910	-3.047926	1.502813
C	-5.581830	-2.761830	0.656215	C	-5.602935	-2.717218	0.669942
H	-6.392193	-2.134045	1.008579	H	-6.415788	-2.077206	0.993387
H	-4.440591	-3.076949	2.549116	H	-4.488485	-2.982189	2.586320
C	-5.395707	-3.230703	-0.584904	C	-5.402045	-3.222010	-0.554665
C	-4.033830	-3.898235	-0.618282	C	-4.042457	-3.894985	-0.550553
H	-3.843914	-4.586296	-1.443237	H	-3.844017	-4.606859	-1.353018
H	-6.022623	-3.064512	-1.453542	H	-6.016711	-3.078801	-1.436052
H	-2.940495	-4.788392	1.095027	H	-2.975799	-4.739784	1.202146
C	-3.943322	-4.450029	0.816647	C	-3.973557	-4.405987	0.900617
H	-4.671594	-5.242985	1.007353	H	-4.707340	-5.190671	1.103898
C	2.830417	-0.741043	4.456481	C	2.600660	-0.587828	4.336393
C	3.601179	-1.914617	3.878494	C	3.523633	-1.740312	3.903913
H	2.996436	-2.686271	3.398450	H	2.985532	-2.633317	3.571516
H	4.173971	-2.387911	4.692167	H	4.251984	-2.006420	4.674881
C	4.550097	-1.177894	2.972731	C	4.151964	-0.937310	2.751000
C	4.743922	0.096842	3.517221	C	4.509962	0.355581	3.455918
H	5.480897	0.816174	3.177879	H	5.297029	1.032255	3.143259
C	3.733829	0.355627	4.446916	C	3.586454	0.562993	4.404319
H	3.559689	1.307341	4.934893	H	3.464183	1.444200	5.023653
H	2.080125	-0.875434	5.229030	H	1.964005	-0.742497	5.208519
H	5.217676	-1.668137	2.275463	H	4.947746	-1.416382	2.181569
**C60_6_Int**				**C60_6_TS**			
126				126			
C60_6_Int_3 SCF Done: -3452.06010259 A.U.				C60_6_TS_3 SCF Done: -3452.02826381 A.U.			
C	3.117315	-1.753484	-0.712477	C	2.977239	-1.677199	-1.036873
C	2.748378	-2.794650	0.335864	C	2.555241	-2.865524	-0.183413
C	3.564337	-0.512725	-0.401449	C	3.470509	-0.527530	-0.516542
C	2.673744	-2.230329	1.841223	C	2.492937	-2.566751	1.396163
C	1.285541	-2.290215	2.470816	C	1.097355	-2.675476	2.003842
C	1.417701	-3.300299	-0.212729	C	1.208778	-3.211514	-0.812550
C	0.280921	-3.491830	0.547677	C	0.055859	-3.478302	-0.098474
C	0.217712	-2.973364	1.930344	C	0.000935	-3.201605	1.350596
C	2.748705	0.401078	-2.742058	C	2.713778	0.806822	-2.667127
C	2.063454	-0.956540	-2.817837	C	1.975563	-0.488449	-2.976918
C	3.594230	0.643051	-1.389628	C	3.555359	0.778389	-1.291301
C	2.316322	-1.969849	-1.924063	C	2.179000	-1.649996	-2.269229
C	1.272963	-2.916400	-1.580208	C	1.095935	-2.597435	-2.095827

C	0.737696	-0.807157	-3.358436	C	0.663567	-0.194258	-3.488690
C	-0.284282	-1.714918	-3.058570	C	-0.397160	-1.097628	-3.353627
C	-0.008246	-2.803582	-2.150491	C	-0.174409	-2.336319	-2.643889
C	2.109703	2.628496	-0.864716	C	2.144339	2.701220	-0.435541
C	1.311602	2.417556	-2.089321	C	1.350375	2.736696	-1.680309
C	3.089853	1.772199	-0.502716	C	3.088426	1.758050	-0.224307
C	1.548024	1.328054	-2.900472	C	1.552496	1.795686	-2.667404
C	0.429417	0.602677	-3.408500	C	0.411505	1.215139	-3.297411
C	-0.034429	2.875922	-1.841129	C	0.020485	3.198003	-1.360385
C	-1.119905	2.211259	-2.380632	C	-1.084382	2.685124	-2.013099
C	-0.887121	1.029253	-3.146400	C	-0.889613	1.644266	-2.971384
C	2.527447	2.205628	2.046169	C	2.522038	1.771529	2.360883
C	1.348390	2.976070	1.470903	C	1.377980	2.675320	1.923186
C	3.481903	1.519942	0.945785	C	3.459191	1.246325	1.159857
C	1.214374	3.259410	0.126391	C	1.264474	3.186070	0.647266
C	-0.092297	3.406240	-0.466377	C	-0.032575	3.479818	0.085708
C	0.174838	2.740663	2.241773	C	0.188246	2.354188	2.638955
C	-1.099319	2.874363	1.661670	C	-1.078623	2.626214	2.087319
C	-1.238280	3.255377	0.292953	C	-1.190391	3.239811	0.802615
C	2.987571	-0.748947	1.976243	C	2.865781	-1.143981	1.784602
C	2.083080	-0.140297	2.959867	C	1.978645	-0.675576	2.857047
C	3.491696	-0.001768	0.966196	C	3.408548	-0.255704	0.918512
C	1.797022	1.209291	2.942821	C	1.746719	0.665002	3.070832
C	0.444950	1.641240	3.158008	C	0.408065	1.110997	3.348981
C	1.047143	-1.104375	3.252966	C	0.897970	-1.633976	2.967054
C	-0.261007	-0.674883	3.528989	C	-0.400218	-1.203465	3.291995
C	-0.571400	0.735410	3.477932	C	-0.650998	0.205380	3.481012
C	-2.938977	1.720769	0.801412	C	-2.967687	1.719438	1.035397
C	-2.146770	1.921451	1.984751	C	-2.163683	1.680829	2.250899
C	-1.891515	0.879573	2.882058	C	-1.955418	0.501934	2.943900
C	-3.376431	0.451539	0.490583	C	-3.435444	0.546790	0.513524
C	-2.835113	0.680218	-1.883302	C	-2.859151	1.163052	-1.791486
C	-2.509857	2.166518	-1.752079	C	-2.481098	2.587947	-1.405252
C	-2.576802	2.754732	-0.255096	C	-2.543716	2.902801	0.174394
C	-3.356481	-0.067471	-0.886659	C	-3.403117	0.275838	-0.930094
C	-2.367708	-2.284238	-1.951381	C	-2.509521	-1.760807	-2.365097
C	-1.641066	-1.282138	-2.844290	C	-1.736872	-0.652171	-3.074786
C	-1.928665	0.064025	-2.866988	C	-1.971197	0.688423	-2.865538
C	-3.317835	-1.595283	-0.847147	C	-3.434292	-1.230136	-1.158991
C	-1.921545	-2.747291	0.969448	C	-2.122654	-2.728378	0.431011
C	-1.035293	-3.361017	-0.038900	C	-1.246875	-3.195127	-0.659365
C	-1.181902	-3.058657	-1.378363	C	-1.363854	-2.670742	-1.932105
C	-2.887607	-1.836308	0.592460	C	-3.036789	-1.731669	0.220647
C	-2.381559	-0.442513	2.570254	C	-2.619436	-0.761956	2.526831
C	-1.379155	-1.402409	2.972651	C	-1.541532	-1.781478	2.643512
C	-1.137610	-2.522281	2.165824	C	-1.334757	-2.743628	1.662037
C	-3.090777	-0.651186	1.376803	C	-3.321254	-0.725938	1.233200
C	4.903884	2.125971	1.347231	C	4.899165	1.718484	1.661993
C	3.557492	3.107090	2.853049	C	3.579433	2.479955	3.311545
C	4.778930	2.171303	2.879391	C	4.763473	1.505995	3.179198
H	4.576556	1.195585	3.330813	H	4.519274	0.476294	3.456320
H	5.649758	2.630728	3.355172	H	5.647577	1.842409	3.727948
C	4.039318	4.186041	1.902274	C	4.110651	3.685843	2.560605
H	3.147858	3.439642	3.807797	H	3.175204	2.660219	4.308614
C	4.846873	3.602041	1.006149	C	4.902286	3.232314	1.578833
H	3.676929	5.207709	1.894149	H	3.787509	4.707285	2.726369
H	5.732557	1.551320	0.932799	H	5.708861	1.191123	1.156943
H	5.275057	4.049820	0.116433	H	5.354085	3.808502	0.779223
H	-5.549100	-1.661757	-0.751426	H	-5.676671	-1.199666	-1.102050

C	-4.733822	-2.224186	-1.205790
H	-5.054368	-4.154635	0.025963
C	-4.649426	-3.700643	-0.871432
C	-4.642639	-2.265157	-2.741750
H	-4.461215	-1.286159	-3.195688
H	-5.516798	-2.734670	-3.201402
C	-3.858609	-4.274603	-1.788544
C	-3.408173	-3.186256	-2.744842
H	-3.486589	-5.292841	-1.793367
H	-3.015363	-3.511110	-3.709421
C	3.721887	-3.167827	2.584951
C	5.090672	-2.810913	2.036168
C	5.153471	-3.278825	0.782303
C	3.460893	-4.506979	1.871525
H	3.602983	-3.134634	3.668944
C	3.827636	-3.952365	0.482646
H	2.424836	-4.850936	1.947541
H	4.142006	-5.296347	2.200831
H	5.813364	-2.180437	2.541487
H	5.938488	-3.109615	0.053990
H	3.806041	-4.640246	-0.363794
C	-3.551179	3.100863	-2.506961
C	-4.922839	2.768721	-1.949127
H	-5.654631	2.139291	-2.442409
H	-3.436032	3.048976	-3.590630
C	-4.977767	3.259001	-0.703592
C	-3.643042	3.921364	-0.417614
H	-3.611549	4.622227	0.417867
H	-5.765729	3.116304	0.026699
H	-2.234039	4.779950	-1.901127
C	-3.273961	4.449409	-1.816619
H	-3.946687	5.241276	-2.157068
C	3.855233	0.622725	-3.861311
C	4.674263	1.762680	-3.231265
H	4.083550	2.655919	-3.006100
H	5.548335	2.032156	-3.830502
C	5.045062	0.941039	-1.983742
C	5.534926	-0.346157	-2.616061
H	6.236264	-1.032749	-2.155803
C	4.827660	-0.534880	-3.738344
H	4.832087	-1.407707	-4.381154
H	3.414447	0.792710	-4.844524
H	5.707639	1.406996	-1.255063
C	-6.601683	-0.916412	1.836628
C	-6.537645	0.376004	1.453126
C	-5.877745	1.162336	2.504039
C	-5.968296	-1.058728	3.195891
H	-5.111316	-1.747129	3.175369
H	-6.671203	-1.462756	3.940032
C	-5.539388	0.347184	3.523858
H	-5.025251	0.625168	4.435561
H	-7.038209	-1.739557	1.283193
H	-6.900785	0.787503	0.517285
H	-5.684289	2.227362	2.443633

C	-4.875637	-1.720130	-1.628389
H	-5.299458	-3.816493	-0.743574
C	-4.865695	-3.234250	-1.548860
C	-4.769421	-1.503966	-3.148186
H	-4.537858	-0.471815	-3.427326
H	-5.659872	-1.846455	-3.682700
C	-4.089665	-3.680077	-2.546425
C	-3.579248	-2.468197	-3.302657
H	-3.762892	-4.698891	-2.720711
H	-3.190794	-2.643364	-4.306865
C	3.495328	-3.660879	1.967210
C	4.882152	-3.274782	1.487661
C	4.934100	-3.523297	0.172148
C	3.182893	-4.845571	1.034706
H	3.370174	-3.809118	3.040865
C	3.582789	-4.077205	-0.238324
H	2.132346	-5.151745	1.050734
H	3.827149	-5.708691	1.223370
H	5.627121	-2.771168	2.093116
H	5.730353	-3.264813	-0.516716
H	3.537726	-4.607814	-1.190489
C	-3.475724	3.682773	-1.986437
C	-4.865023	3.312939	-1.501138
H	-5.614387	2.808005	-2.100029
H	-3.350870	3.818756	-3.061765
C	-4.913638	3.577021	-0.188640
C	-3.557388	4.124925	0.214478
H	-3.507683	4.666373	1.160370
H	-5.710859	3.331729	0.504039
H	-2.099199	5.172060	-1.088241
C	-3.152493	4.875475	-1.067518
H	-3.788999	5.742268	-1.265628
C	3.838622	1.171835	-3.729000
C	4.696284	2.152526	-2.910387
H	4.139245	3.017007	-2.536361
H	5.585958	2.485145	-3.452295
C	5.022370	1.114505	-1.821826
C	5.467084	-0.063656	-2.664941
H	6.135867	-0.847330	-2.327625
C	4.763536	-0.027998	-3.804726
H	4.738991	-0.777353	-4.587647
H	3.414063	1.525947	-4.669376
H	5.695952	1.421119	-1.022213
C	-5.430049	-1.312170	2.007015
H	-5.962172	-1.645398	1.124967
C	-5.678152	-0.119613	2.697701
H	-6.297439	0.694223	2.337215
C	-4.871375	-0.074529	3.836915
H	-4.759752	0.781006	4.492568
C	-4.723671	-2.241757	2.956458
H	-4.067864	-2.979852	2.491000
H	-5.473301	-2.781778	3.556949
C	-4.037029	-1.224699	3.850725
H	-3.466862	-1.524906	4.724030

C60_6_Prod

126

C60_6_Prod_3 SCF Done: -3452.06283321 A.U.

C	-2.973677	-1.707148	0.977733
C	-2.569025	-2.854773	0.062549

C	-3.462859	-0.529759	0.519679
C	-2.522373	-2.480511	-1.501314
C	-1.133793	-2.566446	-2.129164
C	-1.217994	-3.237741	0.659796
C	-0.071139	-3.463389	-0.077960
C	-0.028084	-3.118018	-1.510398
C	-2.674294	0.691221	2.725681
C	-1.943553	-0.623161	2.964886
C	-3.529192	0.737078	1.358418
C	-2.164221	-1.747744	2.203007
C	-1.090163	-2.691317	1.972185
C	-0.626782	-0.365068	3.480487
C	0.426000	-1.269855	3.293259
C	0.186109	-2.469764	2.524998
C	-2.109087	2.686678	0.582963
C	-1.303217	2.654841	1.820423
C	-3.064312	1.763699	0.335477
C	-1.505063	1.669814	2.763749
C	-0.363954	1.050433	3.355523
C	0.028079	3.118038	1.510391
C	1.133783	2.566462	2.129162
C	0.937783	1.483705	3.039103
C	-2.523681	1.899948	-2.252944
C	-1.366054	2.769600	-1.782852
C	-3.453844	1.324220	-1.068335
C	-1.234561	3.213201	-0.484220
C	0.071130	3.463411	0.077948
C	-0.186115	2.469754	-2.524997
C	1.090158	2.691292	-1.972189
C	1.217989	3.237720	-0.659807
C	-2.889277	-1.037701	-1.816135
C	-2.009603	-0.523843	-2.874478
C	-3.413994	-0.188343	-0.901257
C	-1.766538	0.822533	-3.024585
C	-0.426011	1.269852	-3.293280
C	-0.937797	-1.483690	-3.039105
C	0.363947	-1.050423	-3.355517
C	0.626772	0.365073	-3.480497
C	2.973702	1.707158	-0.977753
C	2.164216	1.747709	-2.203010
C	1.943572	0.623171	-2.964931
C	3.462856	0.529753	-0.519678
C	2.889242	1.037696	1.816117
C	2.522361	2.480503	1.501302
C	2.569026	2.854786	-0.062569
C	3.413994	0.188335	0.901258
C	2.523666	-1.899937	2.252935
C	1.766523	-0.822527	3.024576
C	2.009583	0.523852	2.874473
C	3.453851	-1.324220	1.068347
C	2.109081	-2.686652	-0.582964
C	1.234559	-3.213192	0.484212
C	1.366047	-2.769589	1.782842
C	3.064314	-1.763693	-0.335479
C	2.674326	-0.691250	-2.725799
C	1.505066	-1.669813	-2.763766
C	1.303221	-2.654832	-1.820430
C	3.529244	-0.737074	-1.358387
C	-4.893919	1.833334	-1.531666

C	-3.584171	2.663060	-3.156178
C	-4.775781	1.694303	-3.058883
H	-4.544121	0.677314	-3.388672
H	-5.662280	2.065064	-3.580941
C	-4.096453	3.835393	-2.341604
H	-3.188914	2.888524	-4.147641
C	-4.882067	3.341347	-1.374684
H	-3.765907	4.860900	-2.461241
H	-5.703552	1.289242	-1.044597
H	-5.320699	3.881688	-0.543344
H	5.703551	-1.289257	1.044609
C	4.893903	-1.833337	1.531674
H	5.320692	-3.881693	0.543368
C	4.882061	-3.341353	1.374710
C	4.775771	-1.694302	3.058892
H	4.544121	-0.677311	3.388682
H	5.662271	-2.065067	3.580947
C	4.096394	-3.835387	2.341593
C	3.584153	-2.663055	3.156188
H	3.765823	-4.860890	2.461201
H	3.188877	-2.888512	4.147643
C	-3.537870	-3.539516	-2.112792
C	-4.916972	-3.169710	-1.599323
C	-4.955857	-3.481478	-0.296931
C	-3.222328	-4.769863	-1.242394
H	-3.425602	-3.636372	-3.193693
C	-3.603364	-4.061836	0.070826
H	-2.173713	-5.080417	-1.285204
H	-3.873627	-5.619435	-1.465227
H	-5.665856	-2.633693	-2.171192
H	-5.743062	-3.252791	0.412552
H	-3.550748	-4.638099	0.995691
C	3.537867	3.539484	2.112795
C	4.916970	3.169692	1.599315
H	5.665856	2.633673	2.171177
H	3.425600	3.636324	3.193696
C	4.955854	3.481484	0.296930
C	3.603351	4.061849	-0.070812
H	3.550741	4.638135	-0.995663
H	5.743055	3.252822	-0.412563
H	2.173715	5.080412	1.285245
C	3.222326	4.769851	1.242419
H	3.873632	5.619415	1.465267
C	-3.786099	1.012104	3.815445
C	-4.642776	2.038854	3.054395
H	-4.081825	2.916118	2.717903
H	-5.524353	2.351550	3.620816
C	-4.988017	1.058473	1.919317
C	-5.435122	-0.156046	2.707892
H	-6.114077	-0.916449	2.339129
C	-4.720608	-0.182344	3.841091
H	-4.695531	-0.969301	4.586191
H	-3.349623	1.316020	4.767856
H	-5.666580	1.409666	1.142623
C	4.988047	-1.058526	-1.919304
H	5.666595	-1.409720	-1.142592
C	5.435276	0.155952	-2.707916
H	6.114435	0.916209	-2.339230
C	4.720582	0.182440	-3.840985

H	4.695474	0.969455	-4.586024
C	4.642809	-2.038872	-3.054365
H	4.081842	-2.916130	-2.717879
H	5.524360	-2.351563	-3.620817
C	3.786063	-1.012050	-3.815364
H	3.349636	-1.315896	-4.767827

Table S7. Coordinates data set, absolute energies (a.u.) for all DFT optimized C_{60} complexes considering insertions studied by Sola et al.[1]

C60_1_Int				C60_1_TS			
71				71			
C60_1_Int_J SCF Done: -2481.08736049 A.U.				C60_1_TS_J SCF Done: -2481.06877893 A.U.			
C	0.543506	0.535076	3.320742	C	0.565383	3.032874	-1.473999
C	0.066941	1.859342	2.952981	C	0.070290	2.310873	-2.628632
C	1.687585	0.013568	2.716962	C	1.716340	2.585914	-0.821179
C	0.755124	2.607842	1.997180	C	0.754311	1.177692	-3.079770
C	0.017586	3.354958	0.989660	C	0.008735	-0.000767	-3.494539
C	-1.387321	1.825974	2.941732	C	-1.381986	2.308040	-2.567175
C	-2.094374	2.542712	1.976237	C	-2.095704	1.177064	-2.965869
C	-1.377039	3.322943	0.979517	C	-1.385345	-0.000750	-3.441582
C	0.611207	-2.199205	2.515177	C	0.686311	3.035487	1.378375
C	-0.583114	-1.654485	3.143821	C	-0.519549	3.487672	0.698200
C	1.722092	-1.381822	2.305055	C	1.777490	2.586146	0.629938
C	-0.616242	-0.317029	3.537875	C	-0.578529	3.486656	-0.694721
C	-1.809513	0.481179	3.303231	C	-1.783990	3.036438	-1.372236
C	-1.742077	-2.251437	2.498012	C	-1.663007	3.037496	1.475805
C	-2.886562	-1.486275	2.273389	C	-2.819453	2.603815	0.826943
C	-2.920996	-0.091064	2.684627	C	-2.881324	2.603372	-0.627206
C	2.060081	-2.359446	0.065407	C	2.158113	0.736858	2.188975
C	0.899649	-3.208849	0.282097	C	1.012862	1.179434	2.959799
C	2.460316	-1.463122	1.054846	C	2.530344	1.424067	1.045099
C	0.190742	-3.132080	1.481941	C	0.292234	2.313943	2.570247
C	-1.263618	-3.164189	1.470550	C	-1.160038	2.309696	2.632453
C	0.183452	-3.322954	-0.979542	C	0.305232	0.000751	3.436534
C	-1.211066	-3.354971	-0.989691	C	-1.088067	0.000771	3.503657
C	-1.949865	-3.273849	0.261159	C	-1.836755	1.178603	3.090479
C	2.885789	0.275744	-0.643855	C	3.066106	-0.730661	-0.151132
C	2.461713	-0.657486	-1.677070	C	2.530293	-1.423619	1.045712
C	2.885177	-0.118657	0.693653	C	3.066151	0.730570	-0.151461
C	2.060880	-1.947304	-1.332683	C	2.158133	-0.735930	2.189326
C	0.900923	-2.542926	-1.976731	C	1.012871	-1.178156	2.960345
C	1.724689	0.089369	-2.683791	C	1.777457	-2.585887	0.631065
C	0.614088	-0.482884	-3.304468	C	0.686269	-3.034866	1.379697
C	0.193098	-1.827070	-2.943504	C	0.292212	-2.312812	2.571268
C	1.949982	2.065377	1.369463	C	1.960476	0.733866	-2.407238
C	1.950757	2.477320	-0.027451	C	1.960471	-0.734935	-2.406915
C	2.404256	0.793797	1.719693	C	2.432766	1.421782	-1.298619
C	2.405811	1.599057	-1.011130	C	2.432779	-1.422387	-1.298004
C	1.690281	1.485045	-2.272803	C	1.716320	-2.586296	-0.820054
C	0.756228	3.273842	-0.261414	C	0.754299	-1.179047	-3.079235
C	0.069282	3.164037	-1.471123	C	0.070270	-2.312033	-2.627613
C	0.546587	2.251033	-2.498116	C	0.565357	-3.033536	-1.472673
C	-1.738998	-0.535477	-3.320956	C	-1.663037	-3.036829	1.477136
C	-0.579678	0.316042	-3.538293	C	-0.519589	-3.487330	0.699723
C	-0.612801	1.653454	-3.144067	C	-0.578565	-3.486989	-0.693199
C	-2.883842	-0.014291	-2.718160	C	-2.819480	-2.603433	0.828081
C	-3.142907	-2.064417	-1.368515	C	-3.042296	-0.727569	2.415900
C	-1.948764	-2.607995	-1.997037	C	-1.836770	-1.177239	3.091014

C	-1.261274	-1.859785	-2.952652	C	-1.160060	-2.308528	2.633472
C	-3.600652	-0.794914	-1.721418	C	-3.524383	-1.425169	1.307518
C	-4.079212	-0.276690	0.643763	C	-4.022670	0.697572	0.150484
C	-3.602264	-1.600913	1.011944	C	-3.524370	1.425768	1.306894
C	-3.143686	-2.475970	0.027134	C	-3.042293	0.728646	2.415585
C	-4.078474	0.117793	-0.694154	C	-4.022678	-0.697477	0.150791
C	-3.252715	2.357070	-0.064820	C	-3.239628	-0.728660	-2.190229
C	-3.253524	1.945651	1.330868	C	-3.239640	0.727728	-2.190565
C	-3.658151	0.656574	1.677539	C	-3.624494	1.425138	-1.044955
C	-3.656677	1.462584	-1.055778	C	-3.624530	-1.425578	-1.044336
C	-1.806624	2.197113	-2.515107	C	-1.784026	-3.037048	-1.370910
C	-1.384971	3.130221	-1.481092	C	-1.382005	-2.309167	-2.566158
C	-2.093093	3.208407	-0.281840	C	-2.095697	-1.178344	-2.965326
C	-2.918369	1.380839	-2.306713	C	-2.881353	-2.603628	-0.626069
C	5.955518	-0.060103	1.212088	C	5.191438	1.156889	-0.298132
C	5.955798	0.609753	-1.049030	C	5.191542	-1.157100	-0.297394
C	5.947306	1.171692	0.347228	C	5.370573	-0.000408	-1.253375
H	5.074961	1.812167	0.536789	H	4.715831	-0.000723	-2.126413
H	6.831188	1.801034	0.533842	H	6.412344	-0.000464	-1.612343
C	6.004608	-0.739349	-0.982552	C	5.600557	-0.698207	0.971225
H	5.940518	1.217218	-1.945886	H	5.212237	-2.194930	-0.609852
C	6.004833	-1.155296	0.421538	C	5.600523	0.698831	0.970775
H	6.024155	-1.422853	-1.824449	H	5.744165	-1.324082	1.844324
H	5.940131	-0.039121	2.295099	H	5.212160	2.194512	-0.611276
H	6.024513	-2.187078	0.755283	H	5.744098	1.325276	1.843471
C60_1_Prod				C60_2_Int			
71				82			
C60_1_Prod_J SCF Done: -2481.10842974 A.U.				C60_2_Int_J SCF Done: -2675.28868420 A.U.			
C	-0.559537	-3.036471	-1.486010	C	0.520338	0.950926	-3.064771
C	-0.052347	-2.314668	-2.630080	C	1.783726	0.999202	-2.366057
C	-1.719371	-2.582400	-0.849486	C	-0.550460	1.721585	-2.598289
C	-0.733253	-1.177683	-3.078009	C	1.908477	1.814216	-1.235564
C	0.015966	-0.000001	-3.483143	C	2.622362	1.328374	-0.066530
C	1.398822	-2.311676	-2.554763	C	2.348442	-0.341119	-2.361421
C	2.115363	-1.178465	-2.943024	C	3.026361	-0.806918	-1.233333
C	1.409173	-0.000001	-3.424642	C	3.175619	0.048586	-0.065120
C	-0.717011	-3.038157	1.361595	C	-2.111556	-0.147933	-3.010545
C	0.497288	-3.491384	0.698347	C	-0.993576	-0.956018	-3.476527
C	-1.799416	-2.583744	0.601079	C	-1.890537	1.161453	-2.571053
C	0.574145	-3.491066	-0.693156	C	0.292543	-0.419155	-3.503307
C	1.787705	-3.040625	-1.355730	C	1.425334	-1.219354	-3.065713
C	1.630024	-3.040563	1.490886	C	-1.205864	-2.316997	-3.010539
C	2.794571	-2.605495	0.856724	C	-0.120200	-3.085329	-2.587430
C	2.875089	-2.605510	-0.596786	C	1.223061	-2.525597	-2.616041
C	-2.202331	-0.741105	2.136001	C	-3.415844	0.871374	-0.699411
C	-1.066583	-1.177561	2.922769	C	-3.636903	-0.497293	-1.120406
C	-2.571543	-1.440107	1.007888	C	-2.573586	1.689149	-1.420130
C	-0.340161	-2.315216	2.554086	C	-3.008099	-1.000230	-2.264831
C	1.110617	-2.311673	2.639426	C	-2.453076	-2.343441	-2.259696
C	-0.366438	-0.000001	3.408364	C	-3.748858	-1.328958	0.066426
C	1.024548	-0.000001	3.504669	C	-3.229740	-2.623240	0.068415
C	1.779816	-1.178501	3.104364	C	-2.560736	-3.138647	-1.117578
C	-3.247574	0.804805	-0.203494	C	-1.739040	2.844144	0.800917
C	-2.571545	1.440107	1.007888	C	-2.523884	1.713641	1.459862
C	-3.247576	-0.804804	-0.203494	C	-1.766737	2.830360	-0.807987
C	-2.202335	0.741104	2.136001	C	-3.390380	0.884037	0.782748
C	-1.066582	1.177564	2.922768	C	-3.596262	-0.477243	1.234267
C	-1.799417	2.583745	0.601082	C	-1.801072	1.205476	2.595255
C	-0.717013	3.038156	1.361594	C	-2.006187	-0.096151	3.064234

C	-0.340167	2.315216	2.554088	C	-2.927574	-0.960800	2.364412
C	-1.947887	-0.740978	-2.420102	C	0.795227	2.625329	-0.786882
C	-1.947890	0.740977	-2.420102	C	0.821064	2.637602	0.694979
C	-2.440856	-1.436253	-1.338280	C	-0.405076	2.592852	-1.461722
C	-2.440858	1.436252	-1.338281	C	-0.355616	2.617500	1.411273
C	-1.719372	2.582401	-0.849489	C	-0.461037	1.766006	2.566327
C	-0.733252	1.177686	-3.078009	C	1.949029	1.834609	1.118382
C	-0.052353	2.314667	-2.630083	C	1.864442	1.039288	2.265340
C	-0.559540	3.036471	-1.486009	C	0.626056	1.002713	3.007440
C	1.630023	3.040564	1.490887	C	-1.100235	-2.265298	3.069438
C	0.497292	3.491377	0.698348	C	-0.872188	-0.896528	3.504441
C	0.574149	3.491059	-0.693157	C	0.413803	-0.359747	3.476770
C	2.794576	2.605495	0.856720	C	-0.029843	-3.041223	2.622262
C	2.993150	0.728659	2.446671	C	-1.407559	-3.928533	0.776669
C	1.779816	1.178503	3.104362	C	-2.520012	-3.118784	1.238964
C	1.110623	2.311673	2.639423	C	-2.372623	-2.303973	2.362762
C	3.490908	1.426296	1.344831	C	-0.186921	-3.890890	1.452854
C	4.002931	-0.697541	0.194691	C	1.034462	-3.869618	-0.692586
C	3.490906	-1.426295	1.344832	C	-0.236228	-3.914814	-1.398936
C	2.993149	-0.728659	2.446672	C	-1.432750	-3.940838	-0.680428
C	4.002937	0.697541	0.194691	C	1.058606	-3.857833	0.702259
C	3.248666	0.728676	-2.155335	C	2.844368	-2.153880	0.688436
C	3.248666	-0.728676	-2.155336	C	2.819282	-2.166592	-0.768968
C	3.621075	-1.426188	-1.005032	C	1.935274	-3.009243	-1.444829
C	3.621076	1.426190	-1.005031	C	1.984781	-2.985571	1.408538
C	1.787704	3.040626	-1.355732	C	1.531025	-1.167631	3.013786
C	1.398827	2.311676	-2.554760	C	2.429349	-0.302060	2.263833
C	2.115363	1.178467	-2.943022	C	3.067538	-0.787297	1.122138
C	2.875093	2.605510	-0.596783	C	1.313651	-2.481868	2.594840
C	-4.802405	-1.123864	-0.311236	C	-2.351438	4.274500	-1.129643
C	-4.802408	1.123865	-0.311237	C	-2.313018	4.293634	1.117667
C	-5.256174	0.000000	-1.261325	C	-1.673935	5.105651	-0.024237
H	-4.736367	0.000000	-2.224262	H	-0.580944	5.055291	-0.042414
H	-6.337307	-0.000001	-1.422353	H	-1.998536	6.149462	-0.027599
C	-5.428993	0.669851	0.993799	C	-3.768163	4.287119	0.688515
H	-4.988528	2.155156	-0.613884	H	-2.103594	4.601362	2.142985
C	-5.428991	-0.669848	0.993801	C	-3.791078	4.275700	-0.650955
H	-5.701269	1.329464	1.809613	H	-4.609825	4.182772	1.363435
H	-4.988529	-2.155154	-0.613881	H	-2.177336	4.564653	-2.166641
H	-5.701264	-1.329460	1.809617	H	-4.655272	4.160101	-1.294856
				C	5.247684	2.989537	-0.310623
				C	6.188851	0.827841	-0.266876
				C	5.711679	1.886923	-1.224050
				H	6.522406	2.226436	-1.887094
				H	4.911986	1.525247	-1.884984
				C	5.468169	2.623966	0.971616
				H	4.820963	3.922902	-0.657502
				C	6.052817	1.281482	0.998803
				H	6.586169	-0.131535	-0.575501
				H	6.312337	0.744080	1.904429
				H	5.238461	3.210428	1.854615
**C60_2_TS**				**C60_2_Prod**			
82				82			
C60_2_TS_J SCF Done: -2675.26960883 A.U.				C60_2_Prod_J SCF Done: -2675.30888628 A.U.			
C	0.225305	1.042036	-3.086147	C	-0.083891	-1.043049	-3.084128
C	1.435321	1.445945	-2.416280	C	-1.236064	-1.601755	-2.427370
C	-1.012429	1.499677	-2.613273	C	1.200681	-1.351851	-2.613982
C	1.356762	2.275977	-1.301806	C	-1.066808	-2.438760	-1.341395
C	2.279728	2.133489	-0.154136	C	-2.079130	-2.577919	-0.207088

C	2.351778	0.301754	-2.411014	C	-2.294808	-0.569261	-2.423512
C	3.144902	0.060412	-1.296678	C	-3.130746	-0.436221	-1.335235
C	3.201059	0.998207	-0.150870	C	-3.235667	-1.457126	-0.202253
C	-2.007462	-0.732553	-2.972509	C	1.917602	0.987199	-2.950562
C	-0.717405	-1.212071	-3.449605	C	0.580629	1.310425	-3.432809
C	-2.147082	0.594607	-2.553675	C	2.217523	-0.316513	-2.543214
C	0.373122	-0.345566	-3.502975	C	-0.396272	0.318363	-3.491513
C	1.688625	-0.800960	-3.083741	C	-1.759444	0.607284	-3.081403
C	-0.543766	-2.573449	-2.968245	C	0.239939	2.638057	-2.944820
C	0.717899	-3.013550	-2.563094	C	-1.069432	2.920904	-2.549312
C	1.857336	-2.112031	-2.625354	C	-2.090541	1.888692	-2.625624
C	-3.502123	-0.071259	-0.646381	C	3.466214	0.492110	-0.618619
C	-3.346208	-1.455027	-1.047350	C	3.143679	1.850245	-1.009096
C	-2.929284	0.933679	-1.394114	C	3.027818	-0.568522	-1.380280
C	-2.623115	-1.784615	-2.199396	C	2.394132	2.099397	-2.164932
C	-1.722138	-2.926033	-2.191941	C	1.360236	3.122916	-2.156326
C	-3.205169	-2.266007	0.150061	C	2.893646	2.625918	0.193244
C	-2.352472	-3.370623	0.154604	C	1.913337	3.618913	0.199126
C	-1.590330	-3.703967	-1.039648	C	1.126690	3.870125	-0.998826
C	-2.402204	2.307769	0.796830	C	2.652919	-2.017778	0.793146
C	-2.837341	1.017125	1.485151	C	2.922018	-0.690384	1.497016
C	-2.452892	2.261027	-0.810854	C	2.711338	-1.949686	-0.812732
C	-3.454947	-0.028315	0.835219	C	3.411907	0.429466	0.862004
C	-3.270658	-1.386968	1.304663	C	3.057194	1.750753	1.340882
C	-1.980943	0.744644	2.609598	C	2.027243	-0.535102	2.614051
C	-1.811507	-0.556104	3.094334	C	1.694040	0.730390	3.106832
C	-2.473338	-1.650152	2.424635	C	2.223538	1.903515	2.455155
C	0.073549	2.745292	-0.839509	C	0.256153	-2.733812	-0.869282
C	0.120633	2.787588	0.635352	C	0.203131	-2.797109	0.603491
C	-1.089603	2.390023	-1.492079	C	1.378569	-2.236202	-1.505685
C	-0.998782	2.473936	1.381066	C	1.274424	-2.358338	1.360041
C	-0.847047	1.650471	2.543186	C	1.011424	-1.570077	2.522341
C	1.429301	2.343758	1.040177	C	-1.150905	-2.541259	1.000841
C	1.584298	1.582279	2.192520	C	-1.405653	-1.796609	2.136564
C	0.421049	1.218281	2.959223	C	-0.305878	-1.297813	2.919773
C	-0.347317	-2.397175	3.102236	C	0.016143	2.381131	3.117242
C	-0.491934	-1.009478	3.513630	C	0.324057	1.015770	3.514937
C	0.597951	-0.143599	3.444636	C	-0.651257	0.025475	3.416326
C	0.886291	-2.863109	2.643417	C	-1.261438	2.700568	2.651934
C	-0.232929	-4.121996	0.837381	C	-0.286454	4.104247	0.868698
C	-1.514492	-3.635912	1.314080	C	1.039973	3.770696	1.352875
C	-1.572525	-2.791492	2.425204	C	1.189841	2.927272	2.456981
C	0.943887	-3.744829	1.487167	C	-1.415160	3.581723	1.504074
C	2.073542	-3.425637	-0.684649	C	-2.479500	3.149714	-0.680863
C	0.851942	-3.826258	-1.363776	C	-1.310151	3.702192	-1.345458
C	-0.279826	-4.163900	-0.619062	C	-0.232838	4.165798	-0.587041
C	2.118575	-3.386210	0.708849	C	-2.530840	3.091160	0.711072
C	3.354872	-1.250542	0.637153	C	-3.494998	0.817307	0.609834
C	3.308848	-1.292213	-0.815722	C	-3.440724	0.877600	-0.842372
C	2.688939	-2.362572	-1.466674	C	-2.954218	2.026907	-1.476736
C	2.781415	-2.281833	1.387684	C	-3.060129	1.907655	1.372743
C	1.884908	-0.625203	2.969477	C	-1.981547	0.351979	2.933059
C	2.502705	0.434787	2.194381	C	-2.464516	-0.763769	2.141947
C	3.219715	0.125501	1.048428	C	-3.220774	-0.533448	1.012149
C	2.026435	-1.962550	2.580008	C	-2.282662	1.668981	2.565601
C	-3.413383	3.486144	-1.135683	C	3.815736	-3.046051	-1.138999
C	-3.343147	3.550910	1.109746	C	3.734404	-3.140894	1.104779
C	-2.968442	4.487860	-0.053770	C	3.485756	-4.104818	-0.070341
H	-1.902874	4.734391	-0.094186	H	2.458183	-4.478048	-0.123353

H	-3.563736	5.404764	-0.061657	H	4.187432	-4.943037	-0.080382
C	-4.749373	3.144042	0.711110	C	5.084806	-2.563683	0.723580
H	-3.207414	3.920065	2.127226	H	3.635204	-3.533507	2.117701
C	-4.791284	3.105483	-0.627298	C	5.133326	-2.507217	-0.613963
H	-5.519079	2.825210	1.404478	H	5.804667	-2.161806	1.427257
H	-3.342126	3.795788	-2.179252	H	3.791172	-3.351649	-2.185902
H	-5.602209	2.748665	-1.251747	H	5.900911	-2.049686	-1.227429
C	3.667123	3.800278	-0.303438	C	-2.934224	-3.915791	-0.317163
C	5.119031	1.998964	-0.297109	C	-4.547751	-2.351158	-0.310737
C	4.534271	3.010250	-1.256195	C	-4.058189	-3.456642	-1.264457
H	5.346084	3.662626	-1.616245	H	-4.811448	-4.232025	-1.426904
H	4.025179	2.596895	-2.128518	H	-3.697409	-3.080609	-2.226613
C	4.271185	3.703106	0.966436	C	-3.694502	-4.053268	0.988217
H	3.031294	4.619465	-0.619286	H	-2.323860	-4.766478	-0.623291
C	5.148729	2.616201	0.970321	C	-4.656422	-3.120684	0.992023
H	5.787642	1.204049	-0.607407	H	-5.418110	-1.766012	-0.610626
H	5.650826	2.219875	1.845205	H	-5.317202	-2.858004	1.810074
H	3.987642	4.281731	1.837908	H	-3.408726	-4.708790	1.802753
C60_3_Int				C60_3_TS			
93				93			
C60_3_Int_J SCF Done: -2869.48864842 A.U.				C60_3_TS_J SCF Done: -2869.46872144 A.U.			
C	-1.956834	-0.237133	-2.004192	C	-1.580043	-0.164297	-2.369780
C	-1.040968	-1.308956	-2.292315	C	-0.679810	-1.275149	-2.457764
C	-1.521827	1.092566	-2.099581	C	-1.065737	1.133082	-2.389953
C	0.259120	-1.019362	-2.659517	C	0.682779	-1.065120	-2.581780
C	1.447653	-1.935047	-2.376470	C	1.743679	-2.037790	-2.070967
C	-1.329312	-2.410258	-1.348066	C	-1.199946	-2.343070	-1.585257
C	-0.300068	-3.167326	-0.828914	C	-0.331237	-3.155964	-0.881890
C	1.136314	-3.137725	-1.351835	C	1.178472	-3.207385	-1.119676
C	-2.765174	1.672018	-0.045914	C	-2.647561	1.811473	-0.618499
C	-3.215238	0.289564	0.053729	C	-3.304742	0.482569	-0.647964
C	-1.931963	2.064275	-1.098334	C	-1.613720	2.147497	-1.488089
C	-2.814653	-0.641780	-0.901047	C	-2.754284	-0.529249	-1.544934
C	-2.421566	-1.980828	-0.497618	C	-2.411660	-1.853148	-0.977907
C	-3.227203	-0.073242	1.462482	C	-3.478886	0.122809	0.779757
C	-2.856229	-1.360118	1.855892	C	-3.265549	-1.166941	1.243164
C	-2.451779	-2.337039	0.854945	C	-2.721343	-2.187802	0.335272
C	-0.564868	3.417479	0.391482	C	-0.483823	3.454735	0.229218
C	-1.402670	3.003920	1.499188	C	-1.535311	3.101473	1.153679
C	-0.834914	2.970410	-0.883580	C	-0.531596	2.994528	-1.071328
C	-2.496448	2.156530	1.286143	C	-2.600966	2.294421	0.732865
C	-2.778673	1.079733	2.223426	C	-3.114374	1.251048	1.603534
C	-0.559081	2.810929	2.666294	C	-0.946742	2.885273	2.465443
C	-0.837439	1.788768	3.575276	C	-1.448530	1.894043	3.309741
C	-1.968661	0.901842	3.348340	C	-2.558418	1.058517	2.873062
C	1.745851	2.828586	-1.416403	C	2.093681	2.717648	-1.097138
C	1.878146	3.089596	0.081715	C	1.951436	2.997356	0.396775
C	0.232418	2.761213	-1.954918	C	0.708991	2.715069	-1.915262
C	0.831206	3.478078	0.888120	C	0.791088	3.453217	0.984056
C	0.812721	3.100980	2.287472	C	0.484950	3.100469	2.357044
C	2.930711	2.276803	0.632071	C	2.833114	2.141847	1.143971
C	2.943845	1.924497	1.985172	C	2.567682	1.810562	2.477141
C	1.858459	2.348462	2.835353	C	1.364224	2.303410	3.100311
C	0.688535	0.347500	-2.738214	C	1.188250	0.271893	-2.584989
C	2.076897	0.410054	-2.244148	C	2.456954	0.274501	-1.833814
C	-0.184087	1.394200	-2.500221	C	0.335142	1.362347	-2.522426
C	2.518140	1.514674	-1.538628	C	2.808539	1.366455	-1.061157
C	3.320885	1.305408	-0.375617	C	3.358747	1.135716	0.235973
C	2.468138	-0.919671	-1.876729	C	2.701207	-1.067602	-1.389658

C	3.261816	-1.120673	-0.763567	C	3.251985	-1.290633	-0.142460
C	3.702717	0.011431	0.007685	C	3.592165	-0.169603	0.693775
C	2.485972	0.178568	3.495623	C	1.735886	0.114490	3.879644
C	3.334643	0.578068	2.383674	C	2.801658	0.452672	2.950349
C	3.698571	-0.355256	1.415652	C	3.296058	-0.513499	2.075506
C	2.049134	-1.144039	3.599421	C	1.215824	-1.182580	3.902168
C	-0.206830	-0.373585	4.244782	C	-1.077565	-0.287623	4.098187
C	0.248475	0.999674	4.135832	C	-0.535628	1.059405	4.074064
C	1.569498	1.271956	3.770447	C	0.843782	1.257424	3.967896
C	0.674107	-1.425204	3.983143	C	-0.218913	-1.386845	4.013570
C	-1.090045	-2.631623	2.747615	C	-1.770445	-2.512962	2.457996
C	-2.012943	-1.543224	3.026836	C	-2.671628	-1.376424	2.550219
C	-1.579580	-0.433863	3.757726	C	-2.331249	-0.283387	3.357446
C	0.222307	-2.574110	3.215299	C	-0.574287	-2.520441	3.175445
C	1.061063	-3.438287	1.057069	C	0.621497	-3.466428	1.229787
C	-0.307532	-3.498674	0.568368	C	-0.626248	-3.465256	0.482791
C	-1.365560	-3.114213	1.401964	C	-1.806078	-3.004976	1.088450
C	1.321307	-2.996702	2.359758	C	0.646141	-3.015491	2.554758
C	3.247994	-1.732174	1.518580	C	2.763557	-1.863693	2.097302
C	2.975263	-2.222487	0.181228	C	2.730989	-2.362178	0.735490
C	1.912271	-3.072453	-0.039704	C	1.688615	-3.161746	0.317708
C	2.443588	-2.120366	2.596800	C	1.744989	-2.193607	3.000126
C	0.242704	3.886751	-3.078333	C	0.991531	3.820799	-3.023076
C	2.357769	3.981080	-2.325250	C	2.925847	3.824134	-1.879766
C	1.653489	3.687961	-3.663215	C	2.475976	3.544112	-3.325851
H	1.833745	2.677390	-4.042978	H	2.673552	2.519837	-3.657107
H	1.895173	4.424429	-4.434177	H	2.897878	4.255240	-4.041167
C	1.672022	5.270608	-1.914737	C	2.241182	5.152134	-1.616896
H	3.448511	3.977122	-2.310125	H	3.992037	3.767289	-1.656140
C	0.411115	5.214413	-2.363723	C	1.087965	5.150186	-2.298615
H	2.108736	6.022464	-1.267535	H	2.583415	5.891633	-0.902087
H	-0.608428	3.796202	-3.754690	H	0.281758	3.760737	-3.849403
H	-0.393598	5.910939	-2.158323	H	0.295035	5.887950	-2.254549
C	-5.528150	-0.961021	-2.425375	C	-4.401436	-0.793752	-2.941717
C	-5.689778	0.516466	-2.187883	C	-4.685971	0.690263	-2.904619
H	-4.743904	1.063759	-2.302013	H	-3.836435	1.340659	-3.119615
C	-6.221192	0.581074	-0.781004	C	-5.266429	0.808170	-1.513786
C	-6.372506	-0.675114	-0.307316	C	-5.859306	-0.436440	-1.219436
H	-6.735562	-0.950356	0.676943	H	-6.495503	-0.637455	-0.365302
C	-5.942036	-1.633002	-1.328739	C	-5.335772	-1.403776	-2.081191
H	-5.944733	-2.709815	-1.198914	H	-5.504028	-2.471387	-1.999445
H	-6.390837	0.968091	-2.906773	H	-5.478923	0.915014	-3.636251
H	-5.146290	-1.387544	-3.345069	H	-3.928038	-1.287066	-3.782968
H	-6.445816	1.504508	-0.261106	H	-5.571949	1.751688	-1.076026
C	1.919733	-2.733800	-3.669658	C	2.412052	-2.878605	-3.244778
C	1.485130	-4.411967	-2.239227	C	1.623494	-4.510507	-1.916186
C	0.972519	-3.946788	-3.614797	C	1.409012	-4.040756	-3.367030
H	1.177345	-4.673979	-4.404991	H	1.721710	-4.789252	-4.100087
H	-0.088163	-3.677948	-3.621593	H	0.384901	-3.717351	-3.577499
C	3.243872	-3.384941	-3.318258	C	3.608239	-3.591959	-2.643568
H	1.899855	-2.110155	-4.564429	H	2.596717	-2.267941	-4.129689
C	2.984820	-4.385244	-2.465480	C	3.138146	-4.564761	-1.851482
H	1.066735	-5.328460	-1.821098	H	1.084548	-5.397857	-1.581320
H	3.701047	-4.992168	-1.923699	H	3.704244	-5.198793	-1.178822
H	4.215390	-3.007523	-3.615863	H	4.637332	-3.268742	-2.750503

C60_3_Prod	C60_4_Int
93<br>C60_3_Prod_J SCF Done: -2869.50869823 A.U.<br>C 0.348738 1.369470 -2.508528	104<br>C60_4_Int_J SCF Done: -3063.68822482 A.U.<br>C 1.114674 -1.116217 2.642803

C	-1.015747	0.960687	-2.489047	C	0.670189	0.212094	2.902737
C	1.339920	0.399605	-2.488874	C	2.262863	-1.303847	1.887876
C	-1.360103	-0.382646	-2.508834	C	1.397522	1.308084	2.463377
C	-2.642163	-0.948129	-1.902210	C	0.784749	2.667402	2.134466
C	-1.763019	1.911591	-1.646971	C	-0.802424	0.218838	2.843737
C	-2.816707	1.470881	-0.866636	C	-1.473681	1.321544	2.345026
C	-3.458283	0.087902	-0.981330	C	-0.822151	2.676512	2.066141
C	2.682232	1.704016	-0.866273	C	1.216146	-3.163318	0.629435
C	1.805290	2.951082	-0.980850	C	-0.006499	-3.236020	1.544389
C	2.537019	0.571233	-1.646727	C	2.314932	-2.353922	0.855184
C	0.500083	2.762406	-1.901815	C	-0.060914	-2.090086	2.672065
C	-0.827255	2.886063	-1.162858	C	-1.227014	-1.117216	2.540054
C	1.511926	3.222440	0.492037	C	-1.130435	-3.170680	0.513916
C	0.260499	3.534519	0.979046	C	-2.246850	-2.372754	0.648428
C	-0.938608	3.365179	0.127910	C	-2.296933	-1.321995	1.689953
C	3.383657	-0.869762	0.127976	C	2.758947	-2.137725	-1.531969
C	3.508696	0.291104	0.970217	C	1.597695	-2.948436	-1.791315
C	2.913092	-0.726466	-1.162786	C	3.113681	-1.854443	-0.227425
C	3.150355	1.553586	0.473456	C	0.838308	-3.440108	-0.718707
C	2.430115	2.491037	1.320056	C	-0.613569	-3.446939	-0.797401
C	3.145484	-0.078452	2.328150	C	0.926112	-2.433996	-2.973156
C	2.464172	0.823716	3.145602	C	-0.466332	-2.449618	-3.055663
C	2.101964	2.137979	2.634359	C	-1.254619	-2.970532	-1.947653
C	1.653193	-3.038827	-0.981199	C	3.905156	0.559017	-0.945749
C	2.034744	-2.920622	0.491726	C	3.232285	0.185836	-2.263718
C	2.142450	-1.814026	-1.901996	C	3.836928	-0.579108	0.188907
C	2.930739	-1.993028	0.978953	C	2.815727	-1.090629	-2.576715
C	2.787837	-1.485159	2.330247	C	1.677149	-1.288704	-3.453505
C	0.942149	-3.350143	1.319574	C	2.508553	1.312504	-2.783573
C	0.800404	-2.889561	2.633896	C	1.422117	1.142698	-3.650073
C	1.747502	-1.934507	3.153247	C	0.996271	-0.191099	-3.995214
C	-0.323806	-1.359932	-2.489262	C	2.573917	1.089363	1.690255
C	-0.773729	-2.482487	-1.647129	C	2.636519	2.133648	0.652405
C	1.011768	-0.986488	-2.508747	C	3.026991	-0.194282	1.424823
C	0.134678	-3.174670	-0.866708	C	3.148845	1.841714	-0.598950
C	-0.229755	-3.505124	0.472906	C	2.460635	2.332686	-1.748673
C	-2.085622	-2.159421	-1.163198	C	1.482633	2.971472	0.814049
C	-2.445074	-2.495534	0.127478	C	0.827209	3.462163	-0.298359
C	-1.502267	-3.184314	0.969678	C	1.321775	3.141098	-1.611943
C	-0.377420	-1.374105	3.993028	C	-0.926815	1.143466	-3.765425
C	-0.518887	-2.546290	3.145145	C	0.236641	1.974491	-3.500676
C	-1.640743	-2.685103	2.327642	C	0.188089	2.944852	-2.499921
C	-1.371267	-0.390928	3.993400	C	-2.095722	1.325031	-3.020139
C	0.346904	1.382577	3.993815	C	-2.390052	-1.120516	-2.836676
C	1.378126	0.360080	3.993251	C	-1.174856	-1.307264	-3.609393
C	1.023651	-0.992215	3.993566	C	-0.457212	-0.197194	-4.066417
C	-1.001518	1.013417	3.993269	C	-2.839043	0.171215	-2.545164
C	-1.505121	2.763106	2.327967	C	-3.372625	-0.504680	-0.233833
C	-0.108049	3.156647	2.330210	C	-2.911089	-1.848505	-0.529775
C	0.801165	2.480536	3.153448	C	-2.435139	-2.154013	-1.811287
C	-1.945957	1.722101	3.145393	C	-3.345610	0.481944	-1.218704
C	-3.372572	0.858966	1.319555	C	-2.528829	2.138545	0.425111
C	-2.920681	1.951480	0.473064	C	-2.568088	1.109882	1.454540
C	-2.006576	2.893007	0.970001	C	-2.986844	-0.189179	1.131559
C	-2.902891	0.751249	2.633906	C	-2.917886	1.832545	-0.883672
C	-2.680143	-1.672043	2.329791	C	-1.025666	3.144950	-1.728865
C	-3.191457	-1.541759	0.978539	C	-0.651522	3.468099	-0.365732
C	-3.546811	-0.301907	0.491509	C	-1.400073	2.986628	0.687945
C	-2.549206	-0.546546	3.152998	C	-2.149730	2.350220	-1.988222

C	3.003353	-2.570119	-3.005824	C	5.378990	-0.795582	0.515256
C	2.320270	-4.280576	-1.718489	C	5.473561	0.793658	-1.070487
C	2.166500	-3.849868	-3.189057	C	5.897005	0.650415	0.403203
H	1.130300	-3.659137	-3.485353	H	5.392681	1.351482	1.075540
H	2.628234	-4.557243	-3.883124	H	6.979953	0.719670	0.536613
C	3.819654	-4.143800	-1.533218	C	6.058694	-0.474176	-1.663238
H	1.870780	-5.226490	-1.413215	H	5.707123	1.729071	-1.580826
C	4.226920	-3.124150	-2.300802	C	6.002416	-1.421640	-0.717785
H	4.404520	-4.698225	-0.808314	H	6.340864	-0.595765	-2.702736
H	3.180625	-1.945573	-3.882510	H	5.525644	-1.318984	1.461097
H	5.212992	-2.675071	-2.331377	H	6.229467	-2.475915	-0.826945
C	0.724687	3.885985	-3.005724	H	-6.386957	1.153023	-2.771324
C	2.251427	3.800958	-3.188685	C	-6.434697	0.642015	-1.817323
H	2.604353	2.808172	-3.484859	H	-6.588489	2.272629	-0.384846
C	2.547295	4.149577	-1.718077	C	-6.542233	1.209768	-0.596336
C	1.679194	5.379754	-1.533000	C	-6.399304	-0.854814	-1.663431
H	1.866827	6.163493	-0.808095	H	-5.493512	-1.297633	-2.099964
C	0.592788	5.222828	-2.301005	H	-7.248349	-1.335035	-2.174067
H	-0.289040	5.852406	-2.331908	C	-6.562799	0.159911	0.425777
H	2.633351	4.554438	-3.882737	C	-6.468025	-1.047630	-0.172289
H	0.095346	3.727219	-3.882529	H	-6.626023	0.344135	1.492865
H	3.591178	4.233210	-1.412627	H	-6.448862	-2.015584	0.313792
C	-3.727538	-1.315667	-3.005916	C	-0.099048	-2.957568	4.005157
C	-4.867129	0.131404	-1.718754	C	0.845502	-4.111058	3.619121
C	-4.417076	0.049088	-3.189274	H	1.848257	-3.780506	3.330901
H	-5.260467	0.003080	-3.883449	C	-0.024294	-4.557687	2.429345
H	-3.733561	0.850939	-3.485487	C	-1.391915	-4.627209	3.082003
C	-4.819278	-2.098026	-2.300756	H	-2.206599	-5.255725	2.741054
H	-3.275473	-1.781636	-3.882600	C	-1.436464	-3.673289	4.021561
C	-5.498771	-1.235279	-1.533518	H	-2.295310	-3.362906	4.605543
H	-5.461343	0.993805	-1.413538	H	0.914629	-4.875393	4.397854
H	-6.271522	-1.464329	-0.808721	H	0.152521	-2.363777	4.885039
H	-4.923580	-3.176515	-2.331172	H	0.295642	-5.433090	1.862485
				C	1.061183	3.749164	3.267999
				C	-1.183662	3.762253	3.171208
				C	-0.106481	3.456347	4.228478
				H	-0.139183	4.150022	5.072968
				H	-0.128348	2.424345	4.592280
				C	0.639371	5.091900	2.702168
				H	2.076327	3.677246	3.660922
				C	-0.699040	5.099648	2.644360
				H	-2.229666	3.702627	3.475142
				H	-1.335082	5.850711	2.190125
				H	1.320494	5.835683	2.304894
**C60_4_TS**				**C60_4_Prod**			
104				104			
C60_4_TS_J SCF Done: -3063.66600122 A.U.				C60_4_Prod_J SCF Done: -3063.70495285 A.U.			
C	1.023779	-1.171193	2.638427	C	0.992383	-1.242823	2.614380
C	0.535026	0.137838	2.918646	C	0.482665	0.051572	2.924260
C	2.198068	-1.308754	1.913792	C	2.178331	-1.344439	1.902852
C	1.244029	1.263320	2.528967	C	1.180904	1.196647	2.576180
C	0.603263	2.613006	2.214210	C	0.525770	2.545445	2.289914
C	-0.935602	0.105566	2.812240	C	-0.985489	0.002610	2.786752
C	-1.624007	1.201330	2.314144	C	-1.681338	1.100335	2.304767
C	-1.001154	2.579629	2.092209	C	-1.075823	2.491243	2.137394
C	1.239169	-3.159602	0.574267	C	1.261009	-3.166722	0.496247
C	-0.008753	-3.286898	1.448356	C	0.002285	-3.334327	1.348096
C	2.308668	-2.328728	0.855234	C	2.315994	-2.331585	0.816255
C	-0.126731	-2.174580	2.603954	C	-0.145371	-2.258023	2.532701

C	-1.311791	-1.226081	2.461772	C	-1.339158	-1.320511	2.398195
C	-1.101152	-3.221280	0.383886	C	-1.076370	-3.248437	0.271463
C	-2.236849	-2.443221	0.497947	C	-2.224554	-2.485663	0.386310
C	-2.344596	-1.425640	1.560148	C	-2.359589	-1.500629	1.475784
C	2.815906	-2.033917	-1.510963	C	2.850206	-1.957960	-1.533449
C	1.683872	-2.866274	-1.826930	C	1.731578	-2.792937	-1.888688
C	3.125311	-1.778718	-0.189216	C	3.139326	-1.740107	-0.200523
C	0.907936	-3.408489	-0.791910	C	0.950583	-3.377008	-0.881367
C	-0.541162	-3.449687	-0.912773	C	-0.497335	-3.426609	-1.020236
C	1.033163	-2.333694	-3.012310	C	1.090057	-2.230462	-3.064850
C	-0.356880	-2.376933	-3.132843	C	-0.299556	-2.278081	-3.197687
C	-1.163369	-2.949850	-2.065512	C	-1.111632	-2.890811	-2.160524
C	3.874721	0.673413	-0.818910	C	3.866973	0.739439	-0.747740
C	3.252499	0.320039	-2.166820	C	3.268846	0.419393	-2.114576
C	3.801641	-0.497227	0.282962	C	3.792593	-0.465077	0.318652
C	2.876783	-0.957702	-2.525503	C	2.911943	-0.851714	-2.514682
C	1.770423	-1.159681	-3.441264	C	1.820734	-1.038334	-3.451417
C	2.517272	1.441960	-2.679003	C	2.528492	1.547563	-2.604411
C	1.462461	1.269570	-3.584146	C	1.488930	1.390542	-3.530216
C	1.079846	-0.064946	-3.977016	C	1.126441	0.063913	-3.966604
C	2.449067	1.096360	1.786998	C	2.399508	1.067107	1.848592
C	2.515642	2.170107	0.779977	C	2.466763	2.171123	0.874684
C	2.945285	-0.167192	1.503066	C	2.915456	-0.181528	1.535426
C	3.075368	1.926805	-0.462204	C	3.047411	1.971941	-0.366036
C	2.411489	2.432041	-1.619218	C	2.395718	2.504369	-1.517073
C	1.335851	2.973014	0.927141	C	1.274956	2.954742	1.027735
C	0.703834	3.479800	-0.191529	C	0.653921	3.488478	-0.084634
C	1.248671	3.209072	-1.497290	C	1.221872	3.264367	-1.389556
C	-0.879481	1.210854	-3.770480	C	-0.848700	1.310592	-3.750956
C	0.252717	2.066339	-3.448205	C	0.268393	2.168202	-3.387417
C	0.148812	3.007553	-2.424282	C	0.138648	3.076925	-2.337682
C	-2.070106	1.333463	-3.044885	C	-2.051351	1.391485	-3.037007
C	-2.319441	-1.102402	-2.946086	C	-2.279804	-1.028266	-3.006695
C	-1.076487	-1.239650	-3.680092	C	-1.023248	-1.135538	-3.722490
C	-0.369595	-0.103651	-4.094634	C	-0.320966	0.014608	-4.103565
C	-2.807209	0.161081	-2.633006	C	-2.791664	0.211502	-2.685042
C	-3.533314	-0.622057	-0.347820	C	-3.701872	-0.715162	-0.386335
C	-2.888283	-1.912111	-0.676202	C	-2.877536	-1.940761	-0.772040
C	-2.368534	-2.171642	-1.941552	C	-2.329195	-2.134327	-2.024960
C	-3.497943	0.442407	-1.352682	C	-3.656673	0.488257	-1.455324
C	-2.643273	2.050784	0.383690	C	-2.687484	1.996117	0.386034
C	-2.686522	0.984286	1.390775	C	-2.730755	0.893103	1.363284
C	-3.055633	-0.308248	1.019279	C	-3.090582	-0.388164	0.970314
C	-2.978146	1.769057	-0.937043	C	-3.008869	1.770413	-0.941572
C	-1.091670	3.155622	-1.683515	C	-1.113394	3.186649	-1.611530
C	-0.771551	3.449737	-0.306813	C	-0.818355	3.439305	-0.224531
C	-1.543645	2.912553	0.705552	C	-1.600164	2.856530	0.754321
C	-2.180837	2.325360	-1.989067	C	-2.188929	2.350433	-1.952100
C	5.338352	-0.683177	0.651017	C	5.326046	-0.643288	0.702798
C	5.439544	0.950584	-0.888116	C	5.428988	1.036754	-0.785498
C	5.821700	0.777868	0.593639	C	5.792006	0.824335	0.695837
H	5.279002	1.447251	1.268378	H	5.231962	1.466909	1.382395
H	6.898014	0.870646	0.762280	H	6.864623	0.924589	0.882446
C	6.075527	-0.285168	-1.495794	C	6.088602	-0.172682	-1.420413
H	5.663851	1.905233	-1.365972	H	5.648380	2.007827	-1.231503
C	6.015279	-1.259282	-0.578023	C	6.027228	-1.174429	-0.532994
H	6.391233	-0.371470	-2.529183	H	6.420085	-0.224187	-2.451173
H	5.469643	-1.228403	1.586743	H	5.450912	-1.214728	1.623651
H	6.272023	-2.304300	-0.708508	H	6.298672	-2.211958	-0.690597

H -5.566615 1.180239 -2.783847	H -5.341517 1.219105 -2.738799
C -5.584116 0.640663 -1.843648	C -5.196335 0.649711 -1.819686
H -6.215605 2.230905 -0.430168	H -6.161121 2.212529 -0.414614
C -6.060976 1.176432 -0.627811	C -5.882035 1.177149 -0.573396
C -5.771054 -0.857156 -1.753595	C -5.648654 -0.822071 -1.802992
H -5.089433 -1.456523 -2.359216	H -5.093798 -1.460729 -2.497011
H -6.801457 -1.101671 -2.058366	H -6.723124 -0.931687 -1.973231
C -6.100517 0.161390 0.329626	C -5.920133 0.176429 0.316631
C -5.652614 -1.040656 -0.257806	C -5.260309 -1.028258 -0.327315
H -6.292020 0.304555 1.386889	H -6.237045 0.226380 1.352088
H -5.691309 -2.009995 0.226119	H -5.464030 -2.000272 0.124122
C -0.183482 -3.079021 3.910853	C -0.211884 -3.200533 3.812045
C 0.800819 -4.198707 3.524660	C 0.790882 -4.297707 3.409248
H 1.803371 -3.836115 3.277157	H 1.793026 -3.917231 3.188164
C -0.020065 -4.632269 2.295940	C -0.006112 -4.703946 2.155794
C -1.405382 -4.752915 2.902634	C -1.399352 -4.857123 2.736595
H -2.193025 -5.391121 2.518644	H -2.173629 -5.492560 2.322065
C -1.502821 -3.826751 3.865644	C -1.522062 -3.960498 3.724362
H -2.386758 -3.552881 4.430263	H -2.417474 -3.712970 4.283020
H 0.864827 -4.982576 4.284337	H 0.851996 -5.102662 4.146780
H 0.025307 -2.503906 4.814083	H -0.023702 -2.649412 4.734559
H 0.338614 -5.483754 1.715898	H 0.370815 -5.534053 1.556654
C 0.811861 3.670780 3.384526	C 0.696678 3.570924 3.494954
C -1.427984 3.624934 3.213176	C -1.539016 3.495775 3.281696
C -0.377749 3.320079 4.297480	C -0.503215 3.174408 4.374906
H -0.457323 3.989250 5.158581	H -0.608518 3.815971 5.254008
H -0.382174 2.278264 4.632688	H -0.496941 2.123118 4.679066
C 0.371014 5.016378 2.840720	C 0.244008 4.925095 2.983089
H 1.815200 3.616019 3.809296	H 1.693046 3.519121 3.936179
C -0.964492 4.988978 2.738417	C -1.088917 4.880251 2.855914
H -2.481228 3.528400 3.480679	H -2.595107 3.374857 3.526918
H -1.605819 5.734064 2.281693	H -1.733938 5.628846 2.410299
H 1.043476 5.788481 2.484814	H 0.910344 5.717749 2.662602
**C60_5_Int**	**C60_5_TS**
115	115
C60_5_Int_J SCF Done: -3257.88434959 A.U.	C60_5_TS_J SCF Done: -3257.86119595 A.U.
C -0.992628 1.460896 2.751618	C -0.432377 1.718219 2.735632
C -2.212973 1.471697 2.015696	C -1.568500 2.135949 1.982992
C 0.039370 2.296716 2.352159	C 0.825985 2.150081 2.346668
C -2.398984 2.340495 0.952337	C -1.436505 3.005404 0.912027
C -3.317558 2.058060 -0.233777	C -2.383085 3.038836 -0.284851
C -2.705356 0.083012 1.939109	C -2.504054 0.997524 1.909623
C -3.356290 -0.368266 0.801423	C -3.259158 0.782339 0.767019
C -3.852599 0.542185 -0.318401	C -3.402649 1.794117 -0.366267
C 1.719046 0.508880 2.703337	C 1.794572 -0.103159 2.724272
C 0.729605 -0.431074 3.393671	C 0.537601 -0.641943 3.407540
C 1.430616 1.809024 2.327997	C 1.968914 1.217178 2.342618
C -0.785652 0.103822 3.420710	C -0.705512 0.377944 3.414453
C -1.776352 -0.743808 2.631729	C -1.919852 -0.088819 2.620815
C 0.941138 -1.705830 2.580366	C 0.310190 -1.920572 2.606151
C -0.088344 -2.489754 2.089824	C -0.924408 -2.312073 2.113822
C -1.478390 -1.997466 2.117664	C -2.064065 -1.374401 2.121627
C 2.983448 1.705830 0.450840	C 3.396943 0.559990 0.488604
C 3.267527 0.337608 0.800793	C 3.211633 -0.814584 0.851607
C 2.083900 2.428730 1.209988	C 2.802025 1.563437 1.233938
C 2.635354 -0.244201 1.908996	C 2.411797 -1.129385 1.950623
C 2.152430 -1.616146 1.832217	C 1.485105 -2.262511 1.876364
C 3.434355 -0.428526 -0.422429	C 3.252838 -1.667237 -0.358155
C 2.971409 -1.743214 -0.495806	C 2.325366 -2.800958 -0.432230

C	2.320244	-2.352431	0.651152	C	1.407084	-3.020447	0.708005
C	1.216729	3.658126	-0.961266	C	2.432060	2.991144	-0.958645
C	2.073308	2.615147	-1.673593	C	2.887936	1.710393	-1.652328
C	1.222660	3.551987	0.645333	C	2.383563	2.907005	0.648051
C	2.976649	1.796183	-1.026960	C	3.438156	0.631282	-0.984030
C	3.257520	0.472238	-1.545636	C	3.272090	-0.708739	-1.485853
C	1.391674	2.130421	-2.839245	C	2.100605	1.474222	-2.820305
C	1.669798	0.863980	-3.371787	C	1.936347	0.176074	-3.329531
C	2.630380	0.014144	-2.711145	C	2.546880	-0.937875	-2.650294
C	-1.325319	3.192577	0.562354	C	-0.132644	3.437659	0.531592
C	-1.333734	3.290064	-0.908136	C	-0.091144	3.515408	-0.939882
C	-0.128343	3.200264	1.262958	C	0.987314	3.045447	1.249335
C	-0.142712	3.390768	-1.607038	C	1.070511	3.196903	-1.622613
C	0.020628	2.613992	-2.791561	C	0.973631	2.395082	-2.798075
C	-2.418507	2.484126	-1.387842	C	-1.380271	3.120368	-1.428954
C	-2.269152	1.747410	-2.546827	C	-1.476910	2.360535	-2.578691
C	-1.025719	1.809743	-3.271784	C	-0.277776	1.986389	-3.285126
C	0.887794	-1.348743	-3.493231	C	0.447173	-1.640145	-3.446154
C	0.582383	0.027033	-3.854662	C	0.634392	-0.247208	-3.818755
C	-0.732164	0.488932	-3.798572	C	-0.444910	0.636594	-3.793317
C	-0.140811	-2.205815	-3.078095	C	-0.822354	-2.090741	-3.050092
C	1.241753	-3.115995	-1.299005	C	0.153337	-3.376579	-1.243760
C	2.308624	-2.218687	-1.695340	C	1.463178	-2.917917	-1.629619
C	2.146009	-1.357090	-2.786925	C	1.615831	-2.073666	-2.723636
C	0.045650	-3.120563	-1.987184	C	-0.971064	-2.995152	-1.953273
C	-1.289041	-3.537811	0.252728	C	-2.391229	-2.907525	0.270982
C	0.070895	-3.265116	0.890794	C	-1.026552	-3.104816	0.927381
C	1.252838	-3.195883	0.179095	C	0.121919	-3.436684	0.229324
C	-1.304795	-3.459260	-1.355246	C	-2.362699	-2.847571	-1.337198
C	-3.056608	-1.587278	-1.178579	C	-3.373504	-0.489140	-1.199828
C	-3.040987	-1.661877	0.293811	C	-3.399869	-0.547232	0.273556
C	-2.137455	-2.483607	0.952784	C	-2.838289	-1.619840	0.951601
C	-2.169030	-2.340409	-1.928721	C	-2.788674	-1.508613	-1.932130
C	-1.806810	-0.400187	-3.395581	C	-1.764599	0.173181	-3.404200
C	-2.761580	0.353972	-2.624821	C	-2.414039	1.217587	-2.653714
C	-3.378660	-0.237012	-1.538851	C	-3.210274	0.885947	-1.574096
C	-1.512872	-1.724822	-3.033476	C	-1.947449	-1.167474	-3.030694
C	1.718865	5.006596	1.057447	C	3.342001	4.108571	1.057718
C	1.710446	5.153757	-1.184477	C	3.409582	4.225037	-1.184591
C	1.009352	5.860533	-0.009608	C	2.980412	5.142475	-0.024650
H	-0.078943	5.744589	-0.013141	H	1.918314	5.405945	-0.043052
H	1.271457	6.919866	0.058536	H	3.588825	6.048771	0.040373
C	3.157078	5.208175	-0.731787	C	4.782441	3.785794	-0.711801
H	1.497169	5.506895	-2.194319	H	3.341751	4.618403	-2.199835
C	3.162082	5.120522	0.605046	C	4.742188	3.716439	0.625497
H	4.012547	5.187927	-1.397213	H	5.586404	3.466476	-1.365153
H	1.512872	5.224581	2.106315	H	3.211661	4.395046	2.102281
H	4.022870	5.014322	1.255273	H	5.506928	3.329012	1.289014
H	-1.609617	-5.154980	-2.787779	H	-3.210169	-4.354864	-2.760979
C	-1.806919	-4.918359	-1.741318	C	-3.326790	-4.053209	-1.719105
H	-4.111881	-4.916396	-1.926317	H	-5.492253	-3.271806	-1.936406
C	-3.248408	-5.016033	-1.278548	C	-4.721007	-3.651442	-1.275930
C	-1.095601	-5.758301	-0.664506	C	-2.954441	-5.072075	-0.626180
H	-0.006897	-5.648314	-0.669278	H	-1.892694	-5.337259	-0.616513
H	-1.363905	-6.817143	-0.714433	H	-3.565388	-5.977896	-0.671685
C	-3.235859	-5.081626	0.059551	C	-4.745528	-3.701268	0.062614
C	-1.785897	-5.028330	0.502371	C	-3.367958	-4.136914	0.525186
H	-4.087271	-5.046745	0.729614	H	-5.541309	-3.370820	0.720413
H	-1.568975	-5.365866	1.516813	H	-3.288866	-4.515598	1.545241

C	-1.080583	0.145229	4.982868	C	-0.981774	0.532861	4.973054
C	0.285618	0.612822	5.518073	C	0.457409	0.511924	5.520597
H	0.616070	1.569675	5.101980	H	1.097714	1.294429	5.101635
C	1.037055	-0.602767	4.944214	C	0.753988	-0.892548	4.962990
C	0.163216	-1.741012	5.436940	C	-0.459657	-1.659741	5.453121
H	0.508151	-2.754843	5.605211	H	-0.483140	-2.728945	5.630510
C	-1.099765	-1.294931	5.459850	C	-1.494956	-0.809539	5.459150
H	-1.998872	-1.869526	5.651139	H	-2.537865	-1.041540	5.642726
H	0.319031	0.649644	6.610428	H	0.492244	0.546362	6.613070
H	-1.956403	0.752322	5.216487	H	-1.599900	1.404509	5.193061
H	2.106894	-0.683024	5.142326	H	1.730675	-1.330664	5.173960
C	-4.682045	2.873479	-0.152260	C	-3.389244	4.270781	-0.228018
C	-5.428822	0.757557	-0.270908	C	-4.811126	2.534927	-0.342381
C	-5.523580	1.928222	0.725051	C	-4.511973	3.678894	0.643943
H	-6.549173	2.282767	0.859479	H	-5.356718	4.363264	0.761323
H	-5.072203	1.714263	1.698830	H	-4.171027	3.334888	1.625349
C	-5.369365	2.709623	-1.494319	C	-4.076857	4.335975	-1.578233
H	-4.526435	3.894153	0.200055	H	-2.898838	5.181262	0.119356
C	-5.814597	1.448022	-1.565049	C	-4.924703	3.300844	-1.646408
H	-5.958667	-0.164494	-0.027651	H	-5.626154	1.851456	-0.100081
H	-6.251214	0.950496	-2.423453	H	-5.494644	2.971694	-2.507740
H	-5.368009	3.453177	-2.283084	H	-3.812951	5.024908	-2.372462
C	5.756250	-3.224255	-0.385331	C	3.690036	-4.465357	-0.417173
C	6.526735	-0.999906	-0.237935	C	5.152819	-2.678704	-0.299046
C	5.951334	-2.735949	-1.629364	C	4.298691	-4.287313	-1.678210
C	6.430053	-1.353795	-1.537767	C	5.181630	-3.208746	-1.606931
H	5.769176	-3.260771	-2.560927	H	4.007463	-4.798605	-2.588483
H	6.647705	-0.722893	-2.392760	H	5.682081	-2.752632	-2.453293
C	6.123982	-2.167849	0.622034	C	4.572878	-3.758482	0.586820
H	5.398104	-4.212164	-0.122024	H	3.052228	-5.303083	-0.158647
H	6.842041	-0.040604	0.154339	H	5.827308	-1.912177	0.065535
H	6.947925	-2.496511	1.274145	H	5.383495	-4.441925	0.886895
H	5.286846	-1.922110	1.290034	H	4.073102	-3.409140	1.491831
**C60_5_Prod**				**C60_6_Int**			
115				126			
C60_5_Prod_J SCF Done: -3257.89937708 A.U.				C60_6_Int_J SCF Done: -3452.07859523 A.U.			
C	-0.170321	1.766713	2.730278	C	-2.865712	0.498033	-2.154866
C	-1.230633	2.347017	1.974430	C	-2.331698	-0.744854	-2.604976
C	1.138623	2.007485	2.344488	C	-2.138056	1.657599	-2.369640
C	-0.967887	3.183997	0.901479	C	-1.131289	-0.798353	-3.295542
C	-1.895768	3.352076	-0.298510	C	-0.189722	-1.999204	-3.281217
C	-2.325330	1.360435	1.902421	C	-2.658622	-1.768539	-1.593981
C	-3.102606	1.255556	0.759054	C	-1.767888	-2.795149	-1.319699
C	-3.089864	2.272403	-0.378313	C	-0.545634	-3.116156	-2.174609
C	1.758700	-0.368674	2.716575	C	-2.637572	2.478612	-0.079181
C	0.438043	-0.711399	3.400815	C	-3.666921	1.385822	0.197022
C	2.128202	0.912364	2.338610	C	-2.022000	2.673664	-1.305337
C	-0.639580	0.482294	3.409170	C	-3.794918	0.279580	-0.963274
C	-1.910241	0.200966	2.617143	C	-3.386289	-1.128940	-0.550509
C	0.024773	-1.940922	2.601119	C	-3.155422	0.857111	1.531512
C	-1.256796	-2.145458	2.114509	C	-3.032924	-0.491463	1.827237
C	-2.246723	-1.049916	2.123643	C	-3.152086	-1.507301	0.762315
C	3.444647	0.038408	0.500529	C	-0.037592	3.546511	-0.221329
C	3.076426	-1.288219	0.870411	C	-0.650554	3.353001	1.051272
C	3.005896	1.128455	1.238102	C	-0.719407	3.242986	-1.390748
C	2.225167	-1.474956	1.948583	C	-1.921604	2.802613	1.109855
C	1.132896	-2.464151	1.875346	C	-2.247188	1.780949	2.123339
C	3.142297	-2.219284	-0.336910	C	0.409047	3.172633	2.134525
C	1.948986	-3.299859	-0.416718	C	0.053107	2.056277	3.241761

C	0.943845	-3.218743	0.728187
C	2.860185	2.587189	-0.957005
C	3.122259	1.249401	-1.642465
C	2.795352	2.517655	0.648644
C	3.503772	0.099065	-0.971930
C	3.162881	-1.201973	-1.470421
C	2.310022	1.125502	-2.806586
C	1.954548	-0.138615	-3.301642
C	2.395999	-1.325148	-2.613648
C	0.386628	3.417743	0.523586
C	0.442922	3.481766	-0.948385
C	1.434306	2.866297	1.246019
C	1.546325	2.990966	-1.626070
C	1.333908	2.205892	-2.797588
C	-0.889299	3.279014	-1.439611
C	-1.095417	2.536205	-2.586255
C	0.037112	1.983506	-3.285496
C	0.213836	-1.710622	-3.415732
C	0.607510	-0.365180	-3.792777
C	-0.327520	0.670279	-3.785464
C	-1.111783	-1.964923	-3.031797
C	-0.341627	-3.375402	-1.226673
C	1.019685	-3.140855	-1.613242
C	1.299166	-2.316058	-2.685909
C	-1.399088	-2.833503	-1.937788
C	-2.795552	-2.524214	0.278685
C	-1.476205	-2.918879	0.937387
C	-0.382307	-3.419869	0.246683
C	-2.754859	-2.476255	-1.328342
C	-3.402463	0.007505	-1.203488
C	-3.441234	-0.039733	0.270302
C	-3.047943	-1.181777	0.953732
C	-2.974690	-1.091373	-1.929859
C	-1.703386	0.411605	-3.402263
C	-2.192339	1.545507	-2.659831
C	-3.032844	1.340799	-1.581991
C	-2.087910	-0.884556	-3.025983
C	3.922467	3.562912	1.057550
C	4.012136	3.659740	-1.184546
C	3.723053	4.635569	-0.029021
H	2.712673	5.055740	-0.051267
H	4.460686	5.440280	0.034813
C	5.302166	3.020413	-0.706518
H	4.006498	4.054615	-2.201451
C	5.248749	2.962740	0.630861
H	6.050696	2.581925	-1.356595
H	3.834135	3.869097	2.100906
H	5.944998	2.467473	1.298032
H	-3.813584	-3.846051	-2.749301
C	-3.886831	-3.526459	-1.708838
H	-5.912378	-2.434158	-1.934972
C	-5.207050	-2.920818	-1.271006
C	-3.671844	-4.584841	-0.611236
H	-2.661199	-5.004739	-0.597907
H	-4.410201	-5.390245	-0.654976
C	-5.241395	-2.960954	0.067645
C	-3.944562	-3.593955	0.535389
H	-5.980985	-2.513938	0.722136
H	-3.924006	-3.975496	1.557215
C	-1.287109	1.358601	3.029479
C	1.534274	2.485343	-2.537360
C	2.091404	2.644643	-1.125557
C	-0.032186	2.819036	-2.683045
C	1.399034	3.241818	-0.084488
C	1.629955	2.860892	1.278496
C	2.992239	1.581511	-0.829225
C	3.218319	1.183922	0.497008
C	2.520491	1.844586	1.570120
C	-0.400899	0.407750	-3.505347
C	1.034745	0.098658	-3.373773
C	-0.902138	1.628547	-3.079199
C	1.899791	1.027696	-2.819132
C	2.874705	0.578836	-1.880573
C	1.148486	-1.299794	-3.078261
C	2.105110	-1.738661	-2.182694
C	2.989462	-0.783229	-1.565189
C	2.697491	-0.443134	2.106612
C	3.326964	-0.227425	0.816632
C	3.216013	-1.187021	-0.189728
C	1.968178	-1.619570	2.338320
C	0.250167	-0.355319	3.475700
C	0.990017	0.855364	3.265363
C	2.192504	0.819073	2.585869
C	0.737113	-1.582511	3.057199
C	-1.701219	-2.409435	2.482782
C	-2.054051	-0.949574	2.756560
C	-1.180264	-0.032222	3.321813
C	-0.142005	-2.765606	2.650432
C	-0.113642	-3.498044	0.186960
C	-1.544303	-3.171891	0.036667
C	-2.245146	-2.562032	1.067608
C	0.553837	-3.201441	1.364028
C	2.471154	-2.410337	0.045121
C	1.778289	-2.765197	-1.167611
C	0.508227	-3.304832	-1.091160
C	1.853625	-2.622668	1.287403
C	-0.013044	3.952983	-3.798518
C	2.174378	3.486321	-3.594534
C	1.114643	3.435721	-4.710557
H	0.936418	2.429109	-5.101467
H	1.337959	4.121459	-5.532578
C	1.957503	4.887175	-3.054221
H	3.198817	3.210493	-3.848640
C	0.652685	5.165521	-3.175827
H	2.713602	5.476735	-2.548329
H	-0.998760	4.105780	-4.240313
H	0.123854	6.029557	-2.789883
H	0.787263	-4.063082	4.221986
C	-0.190313	-3.897117	3.767021
H	-0.342441	-5.972993	2.759605
C	-0.864669	-5.101366	3.137424
C	-1.322746	-3.362778	4.663241
H	-1.135706	-2.358070	5.054921
H	-1.565681	-4.044162	5.483324
C	-2.163729	-4.805072	2.997906
C	-2.368130	-3.400518	3.533262
H	-2.920925	-5.385332	2.483001
H	-3.391830	-3.109827	3.773387

C	-0.888517	0.676399	4.968507	C	-5.330144	0.409245	-1.359603
C	0.532014	0.441606	5.514456	C	-5.529075	1.930810	-1.231455
H	1.281199	1.120498	5.095341	H	-4.854606	2.516428	-1.863889
C	0.616162	-0.991353	4.956789	C	-5.151468	1.953981	0.261239
C	-0.697582	-1.569619	5.447686	C	-6.023082	0.833843	0.796869
H	-0.879519	-2.623416	5.625419	H	-6.370522	0.758579	1.820989
C	-1.594984	-0.574913	5.454803	C	-6.129651	-0.087515	-0.169880
H	-2.660597	-0.649280	5.639505	H	-6.581985	-1.070049	-0.097617
H	0.572468	0.470429	6.606880	H	-6.564344	2.235726	-1.407906
H	-1.369975	1.630195	5.189196	H	-5.543257	-0.054919	-2.323651
H	1.517092	-1.569701	5.166718	H	-5.200347	2.909085	0.786259
C	-2.707754	4.720458	-0.249589	C	6.326959	-0.763494	1.666139
C	-4.372234	3.215422	-0.361569	H	6.302019	-0.283844	2.637131
C	-3.907972	4.305986	0.621314	C	5.990944	-2.212634	1.436225
H	-4.641549	5.109088	0.733353	H	6.668840	-2.880696	1.989727
H	-3.624591	3.919224	1.605100	H	4.975497	-2.461345	1.774015
C	-3.374706	4.881680	-1.601991	C	6.148138	-2.368890	-0.052643
H	-2.087853	5.549069	0.095650	C	6.653824	-0.192982	0.486577
C	-4.367190	3.984168	-1.668758	C	6.542741	-1.190675	-0.581554
H	-5.280530	2.662014	-0.119167	H	6.936551	0.842621	0.331145
H	-4.978036	3.740207	-2.530384	H	6.732644	-0.989631	-1.630412
H	-3.009306	5.520499	-2.397880	H	5.966823	-3.294276	-0.585854
C	2.760266	-4.669491	-0.415201	C	-0.269204	-2.847786	-4.625610
C	4.423676	-3.163228	-0.304007	C	-0.764037	-4.404838	-3.083344
C	3.516572	-4.731380	-1.728168	C	-1.456402	-3.774696	-4.305769
C	4.508468	-3.833166	-1.661882	H	-1.646903	-4.501258	-5.100481
H	3.207222	-5.308889	-2.591789	H	-2.378649	-3.238911	-4.060660
H	5.174710	-3.527177	-2.460346	C	0.883406	-3.832629	-4.589492
C	3.898310	-4.322717	0.562276	H	-0.343092	-2.208417	-5.506418
H	2.121325	-5.520265	-0.174374	C	0.588347	-4.761050	-3.669719
H	5.312010	-2.630924	0.038837	H	-1.292206	-5.194999	-2.547852
H	4.625366	-5.133166	0.663390	H	1.233330	-5.549451	-3.298885
H	3.548518	-4.009405	1.550815	H	1.818606	-3.707746	-5.123241
				C	0.083268	2.916402	4.580843
				C	0.578801	4.472426	3.037748
				C	1.525079	3.331194	4.801905
				C	1.820571	4.259024	3.881672
				H	2.205556	2.851695	5.496258
				H	2.791709	4.692074	3.670845
				C	-0.528466	4.238343	4.081756
				H	-0.413319	2.402848	5.405319
				H	0.537411	5.387447	2.445241
				H	-0.545259	5.010887	4.855478
				H	-1.525962	4.124215	3.646256
**C60_6_TS**				**C60_6_Prod**			
126				126			
C60_6_TS_J SCF Done: -3452.05430246 A.U.				C60_6_Prod_J SCF Done: -3452.09255210 A.U.			
C	1.687681	-1.448887	-2.810985	C	1.416421	-1.143803	-3.078848
C	2.016044	-0.065774	-2.921578	C	1.927242	0.183086	-2.971616
C	0.376260	-1.845418	-3.019202	C	0.059103	-1.325748	-3.292064
C	1.059016	0.868002	-3.285672	C	1.097716	1.281691	-3.131507
C	1.087527	2.332146	-2.855982	C	1.332998	2.633367	-2.463090
C	3.005380	0.247527	-1.872084	C	2.972480	0.180441	-1.929219
C	2.988436	1.478636	-1.234574	C	3.135576	1.275499	-1.094402
C	2.165806	2.673182	-1.707935	C	2.470730	2.629430	-1.323925
C	0.458303	-3.389715	-1.074909	C	-0.027097	-3.179783	-1.639683
C	1.965237	-3.236087	-0.888719	C	1.490076	-3.255854	-1.496557
C	-0.253358	-2.840975	-2.130034	C	-0.680060	-2.369421	-2.555290
C	2.652414	-2.151964	-1.859691	C	2.296885	-2.118533	-2.301779

C	3.260791	-0.951064	-1.146875	C	3.077389	-1.147526	-1.425863
C	2.026332	-2.895772	0.595139	C	1.628311	-3.187615	0.018835
C	2.805533	-1.875166	1.117295	C	2.548891	-2.380751	0.669504
C	3.437493	-0.881603	0.226441	C	3.290070	-1.338907	-0.069212
C	-2.234425	-2.637429	-0.748841	C	-2.585357	-2.153671	-1.073098
C	-1.521209	-3.198107	0.351385	C	-1.931803	-2.983909	-0.115700
C	-1.625216	-2.485950	-1.985235	C	-1.987244	-1.869135	-2.290928
C	-0.188100	-3.540691	0.186462	C	-0.660848	-3.462473	-0.394607
C	0.799413	-3.228238	1.237849	C	0.382099	-3.466346	0.649994
C	-2.124980	-2.730848	1.672786	C	-2.437756	-2.676423	1.290785
C	-1.046468	-2.389509	2.820479	C	-1.299921	-2.680347	2.429968
C	0.407586	-2.588805	2.403424	C	0.104888	-2.992124	1.922566
C	-2.991744	-0.266664	-2.412973	C	-3.043780	0.550744	-2.269483
C	-3.390142	-0.445060	-0.951469	C	-3.427061	0.176737	-0.843896
C	-2.008585	-1.404148	-2.987491	C	-2.241710	-0.588827	-3.075857
C	-3.138521	-1.597264	-0.220244	C	-3.322705	-1.108950	-0.334302
C	-2.950548	-1.543493	1.193802	C	-3.103213	-1.326978	1.055776
C	-3.396448	0.810742	-0.282202	C	-3.253768	1.286436	0.031761
C	-3.195206	0.884620	1.095481	C	-3.039916	1.093740	1.388433
C	-2.960986	-0.316674	1.837509	C	-2.936955	-0.233931	1.893150
C	-0.283611	0.436196	-3.496347	C	-0.295237	1.070114	-3.350920
C	-1.183802	1.481625	-2.973255	C	-1.031431	2.115433	-2.613103
C	-0.625446	-0.903375	-3.395715	C	-0.813626	-0.210646	-3.459200
C	-2.383200	1.134566	-2.368424	C	-2.249429	1.828513	-2.015865
C	-2.767400	1.799620	-1.168968	C	-2.512445	2.328559	-0.707135
C	-0.367239	2.527969	-2.447910	C	-0.071691	2.941834	-1.962038
C	-0.749178	3.168102	-1.280605	C	-0.344616	3.415727	-0.688046
C	-1.972723	2.809877	-0.629354	C	-1.590353	3.134614	-0.056792
C	-1.627585	1.381394	2.758219	C	-1.379128	1.090015	3.041292
C	-2.467998	2.014380	1.717527	C	-2.258863	2.064817	2.263646
C	-1.842273	3.002096	0.833223	C	-1.452234	3.201902	1.458580
C	-0.310891	1.783103	2.988231	C	-0.021794	1.273265	3.255691
C	0.335295	-0.499794	3.467129	C	0.332716	-1.121535	3.315975
C	-1.010939	-0.929555	3.255179	C	-1.059212	-1.333212	3.099085
C	-1.972708	-0.004536	2.887972	C	-1.891324	-0.236598	2.936003
C	0.685231	0.838013	3.369199	C	0.851572	0.159200	3.423890
C	3.038517	0.192660	2.363599	C	3.079507	-0.602733	2.231834
C	2.422846	-1.203376	2.315146	C	2.284273	-1.880398	1.978133
C	1.225335	-1.544474	2.925658	C	1.066327	-2.166752	2.575429
C	2.069141	1.334503	2.952373	C	2.279235	0.536815	3.039962
C	2.288655	2.577936	0.715703	C	2.622876	2.102338	1.038077
C	3.176781	1.529027	0.177634	C	3.357535	1.057544	0.296714
C	3.427496	0.372575	0.901536	C	3.462290	-0.228153	0.805848
C	1.687193	2.423958	1.955534	C	2.024819	1.817213	2.255932
C	-0.408382	3.306210	1.036639	C	0.064890	3.126731	1.601583
C	0.238811	3.482065	-0.230483	C	0.698736	3.411790	0.356993
C	1.571400	3.139931	-0.385026	C	1.971103	2.933556	0.082382
C	0.314739	2.772373	2.104457	C	0.717538	2.317189	2.518714
C	-2.843861	-1.950117	-4.226674	C	-3.175320	-0.795256	-4.348018
C	-4.215582	-0.363029	-3.424671	C	-4.294380	0.794437	-3.223161
C	-3.472698	-0.643262	-4.743950	C	-3.629968	0.653187	-4.604906
H	-2.737530	0.124203	-5.005262	H	-2.802552	1.350787	-4.767510
H	-4.154500	-0.808557	-5.582906	H	-4.348468	0.728204	-5.425951
C	-4.880718	-1.703798	-3.177890	C	-5.131751	-0.469305	-3.175180
H	-4.855054	0.519522	-3.375250	H	-4.804194	1.731277	-2.993986
C	-4.062368	-2.650592	-3.656207	C	-4.464157	-1.417615	-3.846031
H	-5.786369	-1.845821	-2.599198	H	-6.033900	-0.589454	-2.586202
H	-2.222639	-2.525906	-4.914247	H	-2.657055	-1.318937	-5.152595
H	-4.162505	-3.724662	-3.548686	H	-4.709131	-2.471223	-3.917540

H	2.307316	2.445088	4.882796	H	2.697498	1.265068	5.116671
C	2.918350	1.867966	4.187190	C	3.214511	0.741532	4.311206
H	4.244908	3.635799	3.507181	H	4.749180	2.416536	3.880146
C	4.137593	2.561973	3.610294	C	4.503250	1.363181	3.808196
C	3.541418	0.553737	4.692679	C	3.668275	-0.707438	4.566527
H	2.802527	-0.209413	4.956129	H	2.840463	-1.404452	4.729608
H	4.231169	0.709708	5.526905	H	4.387738	-0.783739	5.386611
C	4.944731	1.611481	3.120414	C	5.169373	0.414856	3.135897
C	4.271361	0.274628	3.365941	C	4.331004	-0.848240	3.183911
H	5.846872	1.749591	2.535362	H	6.070948	0.534686	2.545978
H	4.903421	-0.612588	3.306691	H	4.839825	-1.785329	2.953535
C	3.719737	-3.045740	-2.629872	C	3.216994	-2.997750	-3.257260
C	2.928993	-4.357033	-2.791601	C	2.252809	-4.147854	-3.601275
H	1.981401	-4.235061	-3.325454	H	1.319200	-3.814360	-4.064859
C	2.761335	-4.558495	-1.274267	C	2.092251	-4.584121	-2.133430
C	4.188022	-4.362557	-0.797726	C	3.542070	-4.659675	-1.694512
H	4.598394	-4.786808	0.111584	H	3.910835	-5.283901	-0.888547
C	4.759735	-3.460265	-1.606408	C	4.213021	-3.713412	-2.364924
H	5.732956	-2.996289	-1.493214	H	5.242272	-3.405996	-2.218880
H	3.520698	-5.149773	-3.257915	H	2.722027	-4.918379	-4.219378
H	4.086490	-2.555114	-3.532660	H	3.627189	-2.410710	-4.080226
H	2.247573	-5.457859	-0.931556	H	1.468922	-5.454560	-1.923802
C	-3.883609	3.235558	2.780023	C	-3.179042	2.943945	3.219270
H	-4.136712	2.626468	3.640497	H	-3.589206	2.356769	4.042146
C	-2.891182	4.375265	2.837453	C	-2.214902	4.094109	3.563309
H	-3.323821	5.190932	3.439168	H	-2.684167	4.864643	4.181409
H	-1.913239	4.129190	3.254213	H	-1.281306	3.760603	4.026881
C	-2.896985	4.790519	1.383041	C	-2.054272	4.530356	2.095461
C	-4.775861	3.537638	1.728480	C	-4.175069	3.659711	2.327016
C	-4.180573	4.476591	0.885263	C	-3.504105	4.606010	1.656661
H	-5.698191	3.006621	1.522689	H	-5.204230	3.352101	2.180705
H	-4.568337	4.788607	-0.077697	H	-3.872816	5.230094	0.850548
H	-2.265431	5.579201	0.989817	H	-1.430847	5.400687	1.885689
C	1.557217	3.298683	-4.029608	C	1.902123	3.719705	-3.477283
C	3.059751	3.774789	-2.429052	C	3.488842	3.715103	-1.887670
C	3.086370	3.222612	-3.866148	C	3.410494	3.421310	-3.397043
H	3.615911	3.882104	-4.559431	H	4.008284	4.117609	-3.991853
H	3.485701	2.206263	-3.938644	H	3.668661	2.390234	-3.657709
C	1.287438	4.715959	-3.561676	C	1.834472	5.057450	-2.765645
H	1.130885	3.008614	-4.990999	H	1.419614	3.657192	-4.453719
C	2.183633	4.999907	-2.607115	C	2.780843	5.054567	-1.817276
H	4.013405	3.921667	-1.920012	H	4.464084	3.648168	-1.403352
H	2.214663	5.877986	-1.972090	H	2.943190	5.798264	-1.045416
H	0.436354	5.314503	-3.866034	H	1.065164	5.803922	-2.927345
C	-1.486432	-3.389767	3.977310	C	-1.847017	-3.797690	3.422111
C	-2.989396	-3.866190	2.377277	C	-3.433979	-3.792982	1.832794
C	-2.840091	-2.917189	4.471833	C	-3.112869	-3.243436	4.047404
C	-3.736526	-3.201430	3.517569	C	-4.059524	-3.240703	3.099322
H	-2.999474	-2.339363	5.375135	H	-3.172856	-2.813115	5.040591
H	-4.778146	-2.903143	3.481598	H	-5.051488	-2.807504	3.159105
C	-1.891732	-4.622263	3.148083	C	-2.433901	-4.806787	2.418107
H	-0.704252	-3.513366	4.727714	H	-1.073260	-4.148431	4.106550
H	-3.587773	-4.427081	1.657916	H	-4.118178	-4.139035	1.056749
H	-2.293331	-5.430233	3.766046	H	-2.928882	-5.648838	2.909936
H	-1.090012	-5.004217	2.508542	H	-1.705309	-5.175594	1.689490

Table S8. Coordinates data set, absolute energies (a.u.) for all DFT optimized [10]CPP⊃C_{60} geometries considering insertions 4 to 5’ and 4 to 6’.

C60CPP10_4_5_Int	C60CPP10_4_5_TS
182	182
C60CPP10_4_5_Int SCF Done: -4985.61623095 A.U.	C60CPP10_4_5_TS SCF Done: -4985.59625293 A.U.
C -4.382756 5.508850 -1.425577	C -4.852910 5.125178 -1.370839
C -3.174405 6.193501 -1.415218	C -3.713771 5.918764 -1.321102
C -2.525826 6.520649 -0.211211	C -3.119548 6.275254 -0.097458
C -3.241001 6.272863 0.973922	C -3.826845 5.930642 1.067814
C -4.430165 5.557089 0.964762	C -4.943271 5.108010 1.019013
C -4.993341 5.080533 -0.232636	C -5.440925 4.613228 -0.199880
H -4.809714 5.231019 -2.383337	H -5.235657 4.831491 -2.342604
H -2.679918 6.379825 -2.362705	H -3.221562 6.172884 -2.253971
H -2.824340 6.569574 1.930541	H -3.455452 6.240212 2.038937
H -4.869502 5.280870 1.916984	H -5.369598 4.766257 1.955664
C -1.079330 6.835732 -0.190669	C -1.710804 6.727148 -0.040449
C -0.365332 7.223287 -1.340005	C -1.019408 7.221845 -1.162371
C -0.315883 6.502027 0.941094	C -0.937553 6.432789 1.095337
C 1.018811 7.102705 -1.407131	C 0.370509 7.239769 -1.204949
H -0.901066 7.569771 -2.218388	H -1.572312 7.543425 -2.039615
C 1.063409 6.385022 0.874850	C 0.447833 6.453405 1.053627
H -0.807903 6.187254 1.853219	H -1.412553 6.041395 1.986410
C 1.761017 6.591715 -0.326876	C 1.140530 6.766914 -0.126490
H 1.521480 7.356512 -2.335696	H 0.862404 7.572235 -2.114251
H 1.583100 5.983947 1.737113	H 0.991100 6.077179 1.913063
C -5.986311 3.980937 -0.210156	C -6.328274 3.426695 -0.217138
C -6.674782 3.612355 0.961402	C -7.002147 2.977252 0.934545
C -6.071864 3.094445 -1.297664	C -6.304925 2.551693 -1.316691
C -7.237224 2.348750 1.101388	C -7.433639 1.661218 1.050043
H -6.717328 4.296510 1.802915	H -7.132007 3.643801 1.781271
C -6.642992 1.837218 -1.162997	C -6.745556 1.240210 -1.206519
H -5.562179 3.324561 -2.225464	H -5.802786 2.844522 -2.230931
H -7.694513 2.074010 2.047745	H -7.879485 1.329233 1.983401
H -6.555542 1.134630 -1.984645	H -6.569583 0.560122 -2.032960
C 3.115531 6.015835 -0.480609	C 2.544898 6.326270 -0.274638
C 3.915923 5.699407 0.631986	C 3.371227 6.075447 0.835773
C 3.536325 5.510301 -1.722566	C 3.012785 5.880240 -1.522136
C 4.942338 4.769576 0.540217	C 4.487129 5.254106 0.733044
H 3.683380 6.124644 1.603345	H 3.095983 6.464750 1.811453
C 4.584152 4.603010 -1.818875	C 4.145799 5.085193 -1.628881
H 2.965724 5.737743 -2.616865	H 2.421501 6.060865 -2.413430
C 5.247684 4.127814 -0.674625	C 4.854787 4.663980 -0.490445
H 5.447683 4.470734 1.451987	H 5.022213 4.994068 1.640477
H 4.824702 4.193759 -2.794406	H 4.426616 4.717091 -2.609638
C 6.004998 2.853772 -0.679956	C 5.731665 3.470652 -0.522648
C 5.772471 1.901076 -1.688199	C 5.574420 2.522483 -1.549063
C 6.763395 2.430642 0.427730	C 6.541508 3.093403 0.565167
C 6.104645 0.564100 -1.511522	C 6.021696 1.218199 -1.403911
H 5.195747 2.170689 -2.564746	H 4.963402 2.754759 -2.412361
C 7.080901 1.090220 0.615219	C 6.986047 1.783523 0.715088
H 7.049544 3.145538 1.192454	H 6.773336 3.814547 1.343037
C 6.677774 0.114879 -0.311393	C 6.651091 0.791262 -0.222738
H 5.773813 -0.161123 -2.248365	H 5.736931 0.488917 -2.154256
H 7.591828 0.785518 1.524427	H 7.547833 1.515154 1.605435
C 6.542106 -1.312936 0.046741	C 6.662284 -0.652510 0.091772
C 5.996609 -1.629114 1.300027	C 6.396145 -1.086043 1.401360
C 6.656965 -2.358110 -0.883755	C 6.649449 -1.632813 -0.914242
C 5.419999 -2.867391 1.541028	C 5.957959 -2.378410 1.658968

H	5.891816	-0.848479	2.046029	H	6.422405	-0.370524	2.217386
C	6.094903	-3.605767	-0.632389	C	6.191058	-2.917922	-0.658357
H	7.140552	-2.173469	-1.839383	H	6.924824	-1.363087	-1.929538
C	5.367219	-3.860158	0.546792	C	5.736093	-3.297953	0.617680
H	4.893064	-3.002245	2.477198	H	5.700531	-2.639610	2.679525
H	6.167924	-4.367633	-1.401632	H	6.098989	-3.605257	-1.491683
C	4.381687	-4.965240	0.646390	C	4.842757	-4.468824	0.788928
C	3.983523	-5.692318	-0.490877	C	4.617642	-5.397725	-0.245153
C	3.615656	-5.161631	1.810310	C	3.986647	-4.547956	1.902041
C	2.796229	-6.414555	-0.515640	C	3.498568	-6.223895	-0.247659
H	4.562076	-5.629454	-1.405964	H	5.292748	-5.442161	-1.093606
C	2.419641	-5.865221	1.782376	C	2.878423	-5.382395	1.908656
H	3.900329	-4.681835	2.739319	H	4.102400	-3.856623	2.727831
C	1.934988	-6.436799	0.594078	C	2.559144	-6.176356	0.795592
H	2.500296	-6.909466	-1.436055	H	3.324505	-6.879892	-1.096136
H	1.796846	-5.868217	2.670937	H	2.172762	-5.308007	2.729408
C	0.501762	-6.775030	0.474328	C	1.175928	-6.675206	0.653374
C	-0.291628	-7.125150	1.580496	C	0.391901	-7.050842	1.756596
C	-0.169445	-6.484757	-0.724603	C	0.516894	-6.507423	-0.574553
C	-1.676733	-7.014614	1.536182	C	-0.994926	-7.084227	1.672859
H	0.183451	-7.434012	2.507134	H	0.871755	-7.261221	2.708312
C	-1.550298	-6.367421	-0.765025	C	-0.868332	-6.525040	-0.652106
H	0.398670	-6.200670	-1.603552	H	1.085112	-6.210405	-1.449947
C	-2.336635	-6.536355	0.388658	C	-1.665808	-6.720260	0.489947
H	-2.244972	-7.245150	2.431699	H	-1.561814	-7.331235	2.564499
H	-2.000442	-5.998587	-1.678428	H	-1.326393	-6.241031	-1.591650
C	-3.702862	-5.965662	0.437745	C	-3.081111	-6.279529	0.491847
C	-4.376624	-5.564868	-0.729691	C	-3.756994	-5.951848	-0.697091
C	-4.264054	-5.549186	1.658341	C	-3.713246	-5.907227	1.692033
C	-5.408306	-4.637363	-0.686621	C	-4.870115	-5.121701	-0.691344
H	-4.036541	-5.911558	-1.699587	H	-3.359179	-6.270466	-1.654554
C	-5.319944	-4.648220	1.702699	C	-4.846918	-5.106425	1.698806
H	-3.805359	-5.847039	2.594949	H	-3.258814	-6.155494	2.644686
C	-5.851201	-4.085805	0.528748	C	-5.393343	-4.602154	0.505703
H	-5.798490	-4.259445	-1.625347	H	-5.265760	-4.786360	-1.644015
C	-6.609067	-2.813452	0.542569	C	-6.261040	-3.402362	0.492918
C	-6.470616	-1.931093	1.628126	C	-6.224562	-2.507881	1.577151
C	-7.266102	-2.315566	-0.598603	C	-6.938689	-2.970804	-0.662700
C	-6.800802	-0.587590	1.515461	C	-6.684733	-1.204615	1.456265
H	-5.968194	-2.259417	2.530471	H	-5.704707	-2.783112	2.487234
C	-7.586917	-0.968222	-0.716675	C	-7.389980	-1.661636	-0.788965
H	-7.475235	-2.976698	-1.433762	H	-7.064027	-3.649268	-1.500854
C	-7.279106	-0.055572	0.307426	C	-7.198278	-0.724326	0.240797
H	-6.544136	0.082974	2.328827	H	-6.508439	-0.510813	2.271479
H	-8.028712	-0.607616	-1.641245	H	-7.847432	-1.346361	-1.722495
C	-7.151798	1.398320	0.069591	C	-7.226015	0.736223	0.012129
C	-1.731346	0.762945	-3.302031	C	-1.914125	0.458590	-3.302518
C	-2.731785	-0.119475	-2.745003	C	-2.790599	-0.497224	-2.662772
C	-0.470493	0.249934	-3.624222	C	-0.623197	0.063742	-3.669362
C	-2.419358	-1.468857	-2.542487	C	-2.331447	-1.798440	-2.428561
C	-2.847847	-2.134453	-1.323702	C	-2.629142	-2.453297	-1.166671
C	-3.469022	0.616350	-1.732423	C	-3.550515	0.201235	-1.640843
C	-3.877749	-0.026224	-0.562462	C	-3.832472	-0.429894	-0.427800
C	-3.567382	-1.432475	-0.357184	C	-3.368163	-1.787664	-0.188314
C	0.615919	2.267446	-2.700144	C	0.290034	2.218210	-2.877050
C	-0.696745	2.793941	-2.347323	C	-1.052810	2.623108	-2.477807
C	0.725010	1.016804	-3.318273	C	0.498936	0.959072	-3.452306
C	-1.843922	2.056835	-2.640897	C	-2.129671	1.760387	-2.684988
C	-2.919912	1.964473	-1.667358	C	-3.142338	1.599298	-1.654293

C	-0.573184	3.469134	-1.064917	C	-0.940811	3.360273	-1.229482
C	-1.604334	3.376623	-0.129054	C	-1.911717	3.202050	-0.239672
C	-2.801407	2.610570	-0.436275	C	-3.035149	2.306445	-0.455934
C	2.640898	0.418583	-1.943150	C	2.526058	0.616328	-2.150681
C	2.528199	1.697228	-1.270793	C	2.314245	1.905423	-1.520305
C	1.768452	0.093437	-2.958153	C	1.648422	0.162078	-3.111696
C	1.542947	2.616274	-1.647946	C	1.223220	2.705158	-1.887864
C	0.812718	3.356342	-0.633339	C	0.470087	3.406051	-0.867535
C	2.837719	1.502818	0.135245	C	2.706649	1.805442	-0.128433
C	2.144458	2.221053	1.107950	C	1.993176	2.481129	0.848065
C	1.106631	3.161741	0.715831	C	0.844654	3.278534	0.470994
C	1.762549	-2.430017	-2.182679	C	1.939923	-2.316502	-2.250216
C	2.521014	-1.868956	-0.981538	C	2.688820	-1.633262	-1.110318
C	1.343235	-1.334243	-3.285937	C	1.357945	-1.315088	-3.368122
C	3.029042	-0.589719	-0.926938	C	3.061413	-0.304592	-1.121065
C	3.142353	0.096462	0.344208	C	3.166556	0.436236	0.116587
C	2.065617	-2.500404	0.228085	C	2.357015	-2.253113	0.135450
C	2.190815	-1.867219	1.469098	C	2.465824	-1.547905	1.340847
C	2.740492	-0.532409	1.527501	C	2.888029	-0.176356	1.332982
C	-1.114358	-1.983568	-2.908356	C	-0.998995	-2.193576	-2.844046
C	-0.730197	-2.992156	-1.891222	C	-0.466606	-3.118735	-1.814213
C	-0.165098	-1.147224	-3.452062	C	-0.167689	-1.288386	-3.463838
C	0.581282	-3.103215	-1.480759	C	0.867736	-3.080070	-1.468112
C	0.869604	-3.263943	-0.079781	C	1.235343	-3.151421	-0.079109
C	-1.807227	-3.068309	-0.925771	C	-1.482420	-3.263152	-0.791959
C	-1.530218	-3.266361	0.430440	C	-1.121822	-3.378297	0.555152
C	-0.157456	-3.369757	0.864698	C	0.272094	-3.323739	0.919429
C	1.003328	-0.670275	3.109199	C	1.255387	-0.425316	3.001852
C	1.113984	-1.961042	2.445434	C	1.455243	-1.712716	2.372856
C	-0.033598	-2.697955	2.151826	C	0.384534	-2.588565	2.175505
C	-0.253605	-0.167424	3.449619	C	-0.018823	-0.044330	3.392403
C	0.412439	2.085478	2.691372	C	0.363791	2.235900	2.515920
C	1.718268	1.561509	2.333607	C	1.774080	1.911722	2.198823
C	2.012902	0.213560	2.540558	C	2.237643	0.546946	2.452421
C	-0.555235	1.241078	3.237353	C	-0.478939	1.332372	3.143321
C	-2.303231	2.295496	1.842889	C	-2.398876	2.140120	1.804749
C	-1.295878	3.181290	1.278443	C	-1.519125	3.091490	1.156975
C	0.033981	3.075405	1.690728	C	-0.164358	3.121120	1.499754
C	-1.938934	1.345499	2.799394	C	-1.880428	1.270732	2.771675
C	-3.378515	-0.340739	1.716085	C	-3.192660	-0.597721	1.830240
C	-3.759365	0.650803	0.716911	C	-3.725156	0.307734	0.817736
C	-3.235029	1.941856	0.780871	C	-3.337599	1.648080	0.807539
C	-2.491735	0.000424	2.738232	C	-2.290173	-0.125526	2.785905
C	-1.344221	-2.174168	2.506520	C	-0.947740	-2.189485	2.579869
C	-2.271448	-2.528100	1.439444	C	-1.883978	-2.675798	1.576320
C	-3.264351	-1.627507	1.053675	C	-2.980611	-1.893858	1.209729
C	-1.451538	-0.934253	3.140505	C	-1.143352	-0.933900	3.169920
H	-5.675489	-4.314955	2.672186	H	-5.259276	-4.801528	2.654947
C	2.029092	-1.920285	-4.595851	C	2.037114	-1.884935	-4.688854
C	2.612062	-3.449883	-3.055590	C	2.854413	-3.277705	-3.123848
C	1.816555	-3.429208	-4.373586	C	1.998978	-3.397837	-4.398981
H	0.765683	-3.712117	-4.257619	H	0.991395	-3.784314	-4.217021
H	2.281848	-4.037147	-5.154242	H	2.488261	-3.985614	-5.180444
C	3.874898	-2.717105	-3.467485	C	4.009278	-2.434185	-3.631396
H	2.741765	-4.403494	-2.546840	H	3.112609	-4.189822	-2.584418
C	3.527795	-1.806457	-4.387232	C	3.522864	-1.605562	-4.564942
H	4.836471	-2.837050	-2.985100	H	5.002767	-2.430952	-3.202067
H	1.622962	-1.474808	-5.505049	H	1.542468	-1.525484	-5.592249
H	4.153790	-1.034458	-4.820477	H	4.040831	-0.791168	-5.058678

C 3.991069 3.026767 3.919998	C 3.078935 3.048555 3.510232
C 3.317920 2.630752 5.021784	C 2.580098 2.620148 4.757812
H 2.598547 3.219168 5.581125	H 1.854369 3.157309 5.357749
H 3.917928 3.984412 3.419223	H 2.992006 4.055967 3.120348
C 3.686414 1.248803 5.343884	C 3.009767 1.312446 4.993654
C 4.583012 0.803721 4.437327	C 3.786321 0.880569 3.895948
H 5.045711 -0.175816 4.407264	H 4.345486 -0.048184 3.869335
H 3.280325 0.684436 6.176463	H 2.670371 0.679096 5.805241
H 4.673068 1.612997 2.415316	H 4.441536 2.011615 2.104339
C 4.877306 1.903972 3.453447	C 4.228489 2.129685 3.166234
H 5.940097 2.189946 3.475120	H 5.142843 2.513433 3.647832
C60CPP10_4_5	C60CPP10_4_6_Int
182	182
C60CPP10_4_5 SCF Done: -4985.63869740 A.U.	C60CPP10_4_6_Int SCF Done: -4985.61629307 A.U.
C 5.112762 -4.869018 -1.373846	C -1.720453 6.438915 -1.528337
C 4.017292 -5.721526 -1.318190	C -0.357366 6.665788 -1.431487
C 3.447183 -6.105836 -0.091494	C 0.228695 7.118724 -0.237236
C 4.140296 -5.722820 1.070325	C -0.654958 7.505952 0.787678
C 5.211659 -4.842686 1.015634	C -2.023317 7.274640 0.691106
C 5.677106 -4.324104 -0.206113	C -2.581904 6.646882 -0.437369
H 5.475738 -4.557531 -2.347652	H -2.090140 5.955343 -2.424262
H 3.535637 -6.003479 -2.248584	H 0.267441 6.346302 -2.256489
H 3.789722 -6.050136 2.043373	H -0.265632 7.940245 1.703239
H 5.623508 -4.477668 1.949985	H -2.656092 7.535130 1.534171
C 2.063918 -6.629554 -0.027611	C 1.678880 6.918126 -0.015519
C 1.394864 -7.164198 -1.144626	C 2.551026 6.626532 -1.079982
C 1.280729 -6.370178 1.109883	C 2.192300 6.739606 1.281260
C 0.007580 -7.254943 -1.181315	C 3.768963 5.996493 -0.865735
H 1.960463 -7.460496 -2.022714	H 2.240957 6.813606 -2.102319
C -0.101836 -6.462884 1.073932	C 3.427652 6.142735 1.496224
H 1.738406 -5.951350 1.997584	H 1.576257 6.971169 2.143587
C -0.781993 -6.817817 -0.101915	C 4.203588 5.657710 0.427740
H -0.469740 -7.616933 -2.087082	H 4.338003 5.672372 -1.730226
H -0.660440 -6.113106 1.934734	H 3.749348 5.976762 2.518835
C 6.498389 -3.090994 -0.228827	C -3.900590 5.973713 -0.417609
C 7.150527 -2.602845 0.919616	C -4.482665 5.560687 0.794266
C 6.423311 -2.220941 -1.329984	C -4.486318 5.477399 -1.595989
C 7.509382 -1.264846 1.031135	C -5.466784 4.579896 0.829772
H 7.319804 -3.259517 1.767087	H -4.087198 5.928195 1.735434
C 6.791619 -0.887009 -1.223844	C -5.442622 4.473080 -1.559461
H 5.934910 -2.542989 -2.241826	H -4.130609 5.816975 -2.562900
H 7.939584 -0.906923 1.962246	H -5.836348 4.252291 1.796137
H 6.576134 -0.219330 -2.051073	H -5.759425 4.031580 -2.497988
C -2.208698 -6.453797 -0.245353	C 5.249613 4.627492 0.624945
C -3.039417 -6.237255 0.868797	C 5.239721 3.840559 1.789545
C -2.707156 -6.043392 -1.493366	C 6.109993 4.215514 -0.410332
C -4.197313 -5.476155 0.767701	C 5.911190 2.628831 1.849157
H -2.737176 -6.601337 1.846032	H 4.589911 4.103599 2.615376
C -3.881381 -5.310270 -1.598528	C 6.774592 2.995038 -0.356150
H -2.113087 -6.200552 -2.387289	H 6.218062 4.827684 -1.300103
C -4.603743 -4.917732 -0.458184	C 6.623263 2.134831 0.744190
H -4.736505 -5.232990 1.677274	H 5.760455 1.992946 2.714544
H -4.188764 -4.965875 -2.580049	H 7.376554 2.681143 -1.204343
C -5.542564 -3.772942 -0.492665	C 6.921153 0.687228 0.685385
C -5.432870 -2.818807 -1.519738	C 6.695126 -0.014399 -0.509986
C -6.372918 -3.438516 0.593518	C 7.153504 -0.071774 1.846320
C -5.949614 -1.540032 -1.377090	C 6.529055 -1.394674 -0.511276
H -4.808214 -3.019860 -2.381184	H 6.497935 0.537643 -1.423138
C -6.887121 -2.154083 0.740869	C 6.985210 -1.450983 1.846166

H	-6.565773	-4.169703	1.372555
C	-6.604235	-1.146759	-0.197925
H	-5.703301	-0.797016	-2.127591
H	-7.462636	-1.914586	1.630573
C	-6.693359	0.294614	0.114938
C	-6.445883	0.742279	1.423361
C	-6.737556	1.273167	-0.891924
C	-6.077943	2.056820	1.678547
H	-6.427147	0.026593	2.239366
C	-6.348753	2.581524	-0.638403
H	-7.001106	0.987766	-1.906127
C	-5.910550	2.986290	0.635972
H	-5.830718	2.331734	2.698002
H	-6.297019	3.272378	-1.472370
C	-5.080028	4.203031	0.803351
C	-4.909676	5.143033	-0.231149
C	-4.224080	4.327325	1.912535
C	-3.835861	6.027270	-0.238555
H	-5.590241	5.151673	-1.076353
C	-3.161916	5.219641	1.914537
H	-4.299928	3.631091	2.738886
C	-2.890658	6.029902	0.800504
H	-3.700942	6.691939	-1.087432
H	-2.449680	5.183127	2.732189
C	-1.536770	6.602104	0.652891
C	-0.770094	7.019538	1.753273
C	-0.874069	6.469633	-0.577361
C	0.612656	7.126603	1.664720
H	-1.257212	7.204455	2.706575
C	0.507984	6.560956	-0.659796
H	-1.428829	6.143238	-1.450940
C	1.297828	6.798294	0.479561
H	1.168651	7.403837	2.554319
H	0.977291	6.302121	-1.601082
C	2.734292	6.432555	0.477136
C	3.422861	6.140369	-0.713733
C	3.388660	6.094433	1.675489
C	4.578288	5.370129	-0.711172
H	3.006010	6.437347	-1.670054
C	4.562851	5.354315	1.679046
H	2.924760	6.318915	2.629466
C	5.131506	4.879002	0.484426
H	4.988535	5.056261	-1.664982
C	6.061365	3.726781	0.469622
C	6.077260	2.833534	1.555427
C	6.756041	3.329746	-0.688222
C	6.606308	1.556655	1.434884
H	5.547802	3.082516	2.467530
C	7.276759	2.046561	-0.814043
H	6.841429	4.012281	-1.528076
C	7.140405	1.102406	0.218217
H	6.471395	0.856052	2.252199
H	7.746913	1.754637	-1.748845
C	7.247584	-0.354833	-0.007771
C	1.923190	-0.361805	-3.304605
C	2.749982	0.637091	-2.664454
C	0.612736	-0.032977	-3.668628
C	2.224858	1.912468	-2.427912
C	2.489658	2.581296	-1.166324
H	7.405624	0.432912	2.774388
C	6.581595	-2.130307	0.683660
H	6.203254	-1.881736	-1.424120
H	7.103279	-2.002473	2.774597
C	5.920229	-3.452016	0.740154
C	5.039449	-3.702453	1.803374
C	5.897524	-4.357403	-0.333926
C	4.057228	-4.676073	1.708779
H	5.024042	-3.028064	2.652364
C	4.915574	-5.340349	-0.423695
H	6.620654	-4.251864	-1.138457
C	3.904047	-5.455701	0.549336
H	3.318517	-4.723598	2.498591
H	4.902875	-5.981857	-1.299425
C	2.608229	-6.125613	0.282587
C	2.201402	-6.422747	-1.031316
C	1.627610	-6.250471	1.283642
C	0.867213	-6.652608	-1.346291
H	2.922776	-6.390682	-1.840810
C	0.291640	-6.455309	0.968579
H	1.889812	-6.099790	2.324592
C	-0.135418	-6.580320	-0.364239
H	0.594052	-6.820036	-2.383827
H	-0.443707	-6.405169	1.764954
C	-1.557786	-6.365494	-0.702988
C	-2.599894	-6.585164	0.216124
C	-1.887860	-5.677775	-1.881400
C	-3.843778	-5.985977	0.051472
H	-2.420080	-7.188522	1.101121
C	-3.126965	-5.075276	-2.040052
H	-1.119837	-5.475753	-2.620108
C	-4.106136	-5.125600	-1.031964
H	-4.601062	-6.146915	0.812288
H	-3.268717	-4.437710	-2.903574
C	-5.192541	-4.117296	-1.002550
C	-5.455908	-3.292322	-2.110862
C	-5.820022	-3.760707	0.206089
C	-6.128456	-2.086491	-1.972191
H	-5.056889	-3.546853	-3.086356
C	-6.518348	-2.566450	0.339183
H	-5.687341	-4.376602	1.088317
C	-6.605341	-1.651320	-0.723617
H	-6.184174	-1.422648	-2.828371
C	-6.905484	-0.217693	-0.516316
C	-6.563925	0.390931	0.702138
C	-7.266756	0.638848	-1.572694
C	-6.387304	1.764846	0.797383
H	-6.284315	-0.223535	1.550684
C	-7.096613	2.014211	-1.474499
H	-7.628234	0.219077	-2.506788
C	-6.547134	2.602943	-0.319069
H	-5.976964	2.163122	1.717987
H	-7.333862	2.631058	-2.335546
C	-5.898044	3.934274	-0.342519
C	-0.298017	2.013757	-3.047106
C	-1.602483	1.404933	-2.916067
C	0.819028	1.201372	-3.269799
C	-1.721012	0.013898	-3.016306
C	-2.592786	-0.704842	-2.101672

C	3.546173	-0.023273	-1.644643	C	-2.336431	2.126180	-1.888652
C	3.795274	0.619661	-0.430364	C	-3.171893	1.432541	-1.010314
C	3.261882	1.952160	-0.188622	C	-3.309073	-0.012824	-1.123964
C	-0.186343	-2.232633	-2.879081	C	2.154783	2.551884	-1.689103
C	1.176202	-2.569370	-2.483674	C	0.984193	3.384163	-1.440721
C	-0.460637	-0.983886	-3.449223	C	2.068090	1.475695	-2.578949
C	2.207129	-1.651615	-2.690769	C	-0.213852	3.121511	-2.104340
C	3.211727	-1.440363	-1.660811	C	-1.476006	3.186809	-1.385020
C	1.103897	-3.312854	-1.236427	C	0.972954	3.721597	-0.026577
C	2.066291	-3.106720	-0.247941	C	-0.236886	3.788012	0.665102
C	3.142524	-2.154777	-0.463428	C	-1.486145	3.510885	-0.028259
C	-2.493396	-0.744372	-2.134955	C	3.366846	0.047098	-1.099239
C	-2.214634	-2.020252	-1.506524	C	3.444372	1.140245	-0.149866
C	-1.648664	-0.246553	-3.103281	C	2.709302	0.217609	-2.297195
C	-1.089322	-2.765332	-1.885224	C	2.861772	2.378004	-0.439799
C	-0.301952	-3.429400	-0.872016	C	2.133517	3.098747	0.591382
C	-2.608673	-1.949696	-0.114441	C	3.340136	0.587454	1.190518
C	-1.875393	-2.601433	0.849922	C	2.657754	1.286796	2.186275
C	-0.685358	-3.316701	0.467348	C	2.035934	2.568086	1.878275
C	-2.066626	2.214175	-2.244304	C	1.837333	-2.273672	-2.209462
C	-2.775850	1.494195	-1.101717	C	2.410054	-2.225597	-0.793267
C	-1.436063	1.243099	-3.362471	C	2.005267	-0.909713	-3.047691
C	-3.072715	0.145600	-1.105351	C	3.214225	-1.209376	-0.326246
C	-3.141898	-0.595229	0.133968	C	3.197870	-0.853962	1.079152
C	-2.475120	2.128354	0.139055	C	1.525723	-2.902464	0.118290
C	-2.545278	1.409960	1.342491	C	1.514105	-2.594995	1.483345
C	-2.912263	0.031225	1.342678	C	2.371987	-1.541039	1.976328
C	0.873560	2.240604	-2.842627	C	-0.553393	-0.806358	-3.273472
C	0.296667	3.138456	-1.812353	C	-0.709056	-2.062267	-2.500725
C	0.088598	1.294641	-3.461153	C	0.687579	-0.225210	-3.412491
C	-1.033628	3.032772	-1.464904	C	0.386509	-2.660163	-1.915487
C	-1.402677	3.084899	-0.075184	C	0.279408	-3.171933	-0.574162
C	1.305031	3.333503	-0.790911	C	-1.966419	-1.977405	-1.784941
C	0.939822	3.429654	0.556988	C	-2.082050	-2.508832	-0.497240
C	-0.449244	3.304811	0.921977	C	-0.932247	-3.124387	0.123136
C	-1.292364	0.363337	3.016274	C	0.335616	-1.426135	3.150751
C	-1.544906	1.628140	2.375149	C	0.252687	-2.533281	2.205976
C	-0.523430	2.562013	2.176597	C	-0.943123	-2.788814	1.538298
C	-0.004055	0.038260	3.382263	C	-0.782614	-0.625207	3.386151
C	-0.254274	-2.278083	2.521318	C	0.625454	1.403981	3.361113
C	-1.757611	-2.136310	2.301833	C	1.788368	0.572663	3.109279
C	-2.338740	-0.661882	2.578456	C	1.649064	-0.814094	3.006107
C	0.529082	-1.322102	3.128709	C	-0.635072	0.817457	3.494609
C	2.495567	-2.023738	1.795410	C	-1.649038	2.620727	2.143594
C	1.668823	-3.017205	1.148307	C	-0.336506	3.236623	2.007294
C	0.316715	-3.111484	1.495477	C	0.778140	2.638908	2.600225
C	1.924497	-1.182304	2.757096	C	-1.793969	1.438127	2.870715
C	3.145810	0.751390	1.826029	C	-3.328640	0.538762	1.161114
C	3.725942	-0.124147	0.813148	C	-3.178823	1.771818	0.401051
C	3.410250	-1.483432	0.801051	C	-2.358205	2.791686	0.883393
C	2.261567	0.231223	2.773474	C	-2.656374	0.376265	2.372383
C	0.824243	2.228418	2.577288	C	-2.110387	-1.957251	1.788036
C	1.735990	2.763882	1.576775	C	-2.816387	-1.785968	0.524820
C	2.869691	2.036803	1.208079	C	-3.413974	-0.563448	0.219445
C	1.076849	0.979035	3.159159	C	-2.034104	-0.898835	2.692771
H	4.993583	5.072169	2.634055	H	-6.932711	-2.307662	1.308772
C	-2.147531	1.775032	-4.682098	C	2.793379	-1.418743	-4.331399
C	-3.032084	3.125184	-3.116424	C	2.554190	-3.323833	-3.162603
C	-2.187659	3.288126	-4.394107	C	2.127354	-2.791518	-4.543360

H -1.201130 3.726976 -4.215412	H 1.042852 -2.737687 -4.678978
H -2.709170 3.848515 -5.174828	H 2.578064 -3.352695 -5.366417
C -4.142722 2.221496 -3.619238	C 4.029759 -2.974865 -3.163426
H -3.336185 4.023167 -2.577269	H 2.291051 -4.348390 -2.902489
C -3.616303 1.418299 -4.553325	C 4.172870 -1.838654 -3.859024
H -5.133072 2.166278 -3.186035	H 4.778452 -3.499670 -2.585096
H -1.637305 1.440534 -5.586410	H 2.751731 -0.698602 -5.149772
H -4.092259 0.577167 -5.044506	H 5.071533 -1.242647 -3.972379
C -2.640982 -3.018578 3.286316	C -4.127304 -2.218502 4.779536
C -2.240910 -2.630943 4.696807	H -4.598111 -1.338330 5.200631
H -1.573579 -3.204728 5.329802	C -3.013816 -2.837258 5.229467
H -2.598592 -4.077768 3.030121	H -2.411049 -2.536045 6.079373
C -2.725128 -1.402996 4.927532	C -2.718058 -3.986597 4.370066
C -3.452489 -0.959448 3.672548	H -1.861166 -4.640013 4.494548
H -4.153182 -0.129224 3.774546	C -4.649921 -2.957966 3.577596
H -2.534160 -0.770657 5.787094	H -4.714475 -2.318696 2.687341
H -4.379505 -2.263155 2.133214	H -5.666251 -3.346127 3.749488
C -3.998558 -2.303760 3.156081	C -3.649334 -4.068514 3.395451
H -4.759436 -2.727969 3.817811	H -3.679903 -4.787360 2.586723
**C60CPP10_4_6_TS**	**C60CPP10_4_6**
182	182
C60CPP10_4_6_TS SCF Done: -4985.59628665 A.U.	C60CPP10_4_6 SCF Done: -4985.64152132 A.U.
C 2.587954000 -6.133598000 -1.779516000	C 3.287275 -5.761118 -1.555849
C 1.278584000 -6.581084000 -1.684039000	C 2.022762 -6.321739 -1.483282
C 0.741917000 -7.049046000 -0.471106000	C 1.557311 -6.932876 -0.306625
C 1.652847000 -7.193581000 0.591216000	C 2.499640 -7.111688 0.723507
C 2.957136000 -6.725416000 0.501881000	C 3.768202 -6.545266 0.651565
C 3.436144000 -6.093176000 -0.658595000	C 4.162868 -5.773668 -0.456568
H 2.887915000 -5.678633000 -2.715043000	H 3.533158 -5.178942 -2.435497
H 0.641901000 -6.446159000 -2.549526000	H 1.344897 -6.147700 -2.309874
H 1.324048000 -7.611333000 1.536919000	H 2.221526 -7.649555 1.624533
H 3.584484000 -6.800487000 1.383447000	H 4.438497 -6.658207 1.498404
C -0.725318000 -7.094950000 -0.259995000	C 0.100704 -7.101904 -0.102407
C -1.624094000 -6.875308000 -1.320862000	C -0.805586 -7.017272 -1.174819
C -1.277669000 -7.090109000 1.033718000	C -0.453249 -7.077139 1.189636
C -2.924931000 -6.451366000 -1.088841000	C -2.143402 -6.710968 -0.968706
H -1.286235000 -6.937782000 -2.348592000	H -0.448257 -7.104660 -2.195133
C -2.595318000 -6.716888000 1.264038000	C -1.799799 -6.807371 1.395769
H -0.646851000 -7.288306000 1.893211000	H 0.192847 -7.162708 2.056824
C -3.422722000 -6.279656000 0.214447000	C -2.660622 -6.511615 0.323318
H -3.526300000 -6.150128000 -1.939599000	H -2.766489 -6.522087 -1.836074
H -2.952051000 -6.678041000 2.288145000	H -2.162945 -6.742323 2.415913
C 4.653817000 -5.247539000 -0.662879000	C 5.271128 -4.793508 -0.406076
C 5.348183000 -4.959008000 0.528343000	C 5.707996 -4.261227 0.820003
C 5.026420000 -4.503419000 -1.796977000	C 5.735123 -4.154074 -1.569408
C 6.228886000 -3.888672000 0.616811000	C 6.414089 -3.065699 0.881366
H 5.141363000 -5.525236000 1.429642000	H 5.397384 -4.725416 1.750244
C 5.882230000 -3.415418000 -1.703945000	C 6.408665 -2.943017 -1.507413
H 4.582376000 -4.711975000 -2.762814000	H 5.492969 -4.561997 -2.545028
H 6.701654000 -3.666973000 1.569388000	H 6.670750 -2.666054 1.857122
H 6.024868000 -2.782207000 -2.574498000	H 6.622503 -2.426902 -2.436872
C -4.608170000 -5.423670000 0.450077000	C -3.930726 -5.775514 0.520329
C -4.692838000 -4.675449000 1.635913000	C -4.123503 -5.024612 1.692719
C -5.518633000 -5.100903000 -0.574034000	C -4.859851 -5.578513 -0.518632
C -5.504387000 -3.554852000 1.725751000	C -5.076529 -4.019728 1.758857
H -4.014280000 -4.883600000 2.454488000	H -3.432276 -5.126174 2.520552
C -6.320023000 -3.967457000 -0.491437000	C -5.808214 -4.562999 -0.457927
H -5.555155000 -5.702763000 -1.476454000	H -4.805838 -6.187488 -1.415527
C -6.263760000 -3.119424000 0.627036000	C -5.883267 -3.706515 0.652958

H	-5.432814000	-2.933743000	2.611050000	H	-5.093497	-3.375628	2.631116
H	-6.961724000	-3.712040000	-1.329844000	H	-6.463897	-4.398015	-1.308373
C	-6.740716000	-1.720651000	0.616931000	C	-6.532031	-2.378171	0.607797
C	-6.719071000	-0.989672000	-0.582524000	C	-6.472169	-1.622794	-0.574567
C	-6.985453000	-0.994206000	1.797564000	C	-6.959185	-1.720066	1.774917
C	-6.765787000	0.395594000	-0.574772000	C	-6.651934	-0.244382	-0.554709
H	-6.547996000	-1.501449000	-1.523919000	H	-6.132836	-2.093721	-1.491409
C	-6.991284000	0.396988000	1.800502000	C	-7.136226	-0.342200	1.795954
H	-7.105225000	-1.521372000	2.739101000	H	-7.089395	-2.287232	2.691973
C	-6.785015000	1.130951000	0.618361000	C	-6.898011	0.434947	0.649175
H	-6.747963000	0.941727000	-1.502110000	H	-6.444650	0.323677	-1.455172
H	-7.112125000	0.919276000	2.745384000	H	-7.397503	0.147697	2.729686
C	-6.291858000	2.532012000	0.604348000	C	-6.580966	1.876940	0.734833
C	-5.477147000	2.958144000	1.667181000	C	-5.798411	2.314551	1.814184
C	-6.347797000	3.390234000	-0.510466000	C	-6.770536	2.780541	-0.324112
C	-4.653698000	4.071042000	1.567682000	C	-5.086086	3.501879	1.750541
H	-5.385210000	2.329819000	2.545336000	H	-5.624986	1.647201	2.651097
C	-5.540776000	4.521182000	-0.594223000	C	-6.060789	3.976954	-0.382863
H	-7.038479000	3.231392000	-1.332232000	H	-7.436988	2.515792	-1.140848
C	-4.586505000	4.833397000	0.389000000	C	-5.120298	4.319409	0.607706
H	-3.970552000	4.262633000	2.384836000	H	-4.388095	3.713017	2.550526
H	-5.615206000	5.133876000	-1.487540000	H	-6.197486	4.619015	-1.247571
C	-3.494968000	5.822886000	0.174257000	C	-4.029346	5.295985	0.374071
C	-3.098423000	6.209091000	-1.128661000	C	-3.692013	5.711465	-0.927305
C	-2.541457000	6.060737000	1.184030000	C	-3.124527	5.641083	1.394507
C	-1.777354000	6.554617000	-1.413670000	C	-2.452898	6.271544	-1.214276
H	-3.776051000	6.101133000	-1.976923000	H	-4.371281	5.516157	-1.750203
C	-1.230271000	6.414483000	0.905067000	C	-1.877074	6.176363	1.106560
H	-2.805192000	5.887770000	2.221685000	H	-3.355377	5.409684	2.427995
C	-0.782002000	6.529491000	-0.421674000	C	-1.476161	6.429339	-0.215957
H	-1.494386000	6.745818000	-2.444996000	H	-2.216520	6.522311	-2.243917
H	-0.516340000	6.421209000	1.721268000	H	-1.163898	6.294997	1.915764
C	0.663839000	6.469696000	-0.704307000	C	-0.040854	6.578981	-0.534712
C	1.603704000	6.723609000	0.315135000	C	0.907433	7.017054	0.407078
C	1.157503000	5.895596000	-1.887251000	C	0.459780	6.031495	-1.726588
C	2.918189000	6.290545000	0.216560000	C	2.262753	6.749356	0.244800
H	1.287671000	7.246941000	1.212308000	H	0.577377	7.526845	1.307493
C	2.472795000	5.464319000	-1.989471000	C	1.810639	5.762289	-1.884709
H	0.472488000	5.672105000	-2.698782000	H	-0.227450	5.669472	-2.483411
C	3.363039000	5.567691000	-0.906277000	C	2.740948	6.020469	-0.861555
H	3.602026000	6.487378000	1.036181000	H	2.947524	7.063085	1.026467
H	2.766947000	4.905242000	-2.871323000	H	2.110695	5.213387	-2.767953
C	4.541209000	4.686766000	-0.846286000	C	4.046335	5.315959	-0.848781
C	5.176950000	4.097895000	-1.953340000	C	4.481226	4.571635	-1.960355
C	5.023800000	4.319528000	0.425924000	C	4.774348	5.138673	0.343743
C	6.074302000	3.049820000	-1.770495000	C	5.432724	3.570427	-1.834488
H	4.897080000	4.377685000	-2.964872000	H	4.010167	4.707280	-2.926891
C	5.953863000	3.311612000	0.611624000	C	5.742933	4.148799	0.466321
H	4.354385000	4.561656000	1.245436000	H	4.526501	5.721088	1.224184
C	6.432566000	2.583969000	-0.491435000	C	6.025800	3.273708	-0.595643
H	6.426610000	2.506201000	-2.641695000	H	5.630389	2.932424	-2.689227
C	6.917326000	1.191569000	-0.309216000	C	6.664289	1.954440	-0.398728
C	6.449136000	0.459381000	0.795566000	C	6.469106	1.267153	0.809832
C	7.577618000	0.465038000	-1.317494000	C	7.227840	1.223588	-1.460259
C	6.454062000	-0.925122000	0.800997000	C	6.625259	-0.110257	0.889345
H	5.929461000	0.962869000	1.599965000	H	6.051619	1.789945	1.663169
C	7.584295000	-0.928124000	-1.314367000	C	7.394635	-0.153052	-1.376531
H	8.033402000	0.992648000	-2.151005000	H	7.482305	1.729869	-2.386798
C	6.933385000	-1.650658000	-0.299007000	C	6.993454	-0.871080	-0.233323

H	5.933372000	-1.446720000	1.596512000	H	6.313799	-0.606412	1.801003
H	8.033498000	-1.461707000	-2.147617000	H	7.782040	-0.683681	-2.240437
C	6.442440000	-3.032675000	-0.477017000	C	6.692540	-2.320768	-0.278156
C	0.634600000	-1.915448000	-2.926071000	C	0.832588	-1.803625	-3.050411
C	1.858877000	-1.171205000	-2.754160000	C	1.936820	-0.887915	-2.901517
C	-0.559012000	-1.230477000	-3.192910000	C	-0.453324	-1.299315	-3.280522
C	1.830087000	0.224598000	-2.860104000	C	1.702573	0.490112	-2.995226
C	2.585958000	1.036923000	-1.922442000	C	2.352385	1.397438	-2.066644
C	2.628036000	-1.803062000	-1.692953000	C	2.815692	-1.408364	-1.865384
C	3.344830000	-1.019136000	-0.785400000	C	3.425730	-0.532771	-0.965081
C	3.328211000	0.431619000	-0.902790000	C	3.198091	0.900831	-1.068007
C	-1.789290000	-2.715161000	-1.648404000	C	-1.416584	-2.961118	-1.724059
C	-0.545333000	-3.417133000	-1.358358000	C	-0.076643	-3.475443	-1.470659
C	-1.794264000	-1.637058000	-2.542059000	C	-1.596680	-1.887326	-2.603833
C	0.639871000	-3.025629000	-1.979558000	C	1.023009	-2.908888	-2.116531
C	1.874263000	-2.955240000	-1.218440000	C	2.251164	-2.662139	-1.384146
C	-0.543902000	-3.754578000	0.055046000	C	0.006897	-3.819562	-0.061440
C	0.642464000	-3.690800000	0.787734000	C	1.189259	-3.589412	0.644177
C	1.878995000	-3.280575000	0.137764000	C	2.334240	-2.994456	-0.030998
C	-3.286274000	-0.354014000	-1.108775000	C	-3.223441	-0.846674	-1.125298
C	-3.271517000	-1.449720000	-0.154791000	C	-3.030946	-1.935883	-0.184747
C	-2.577022000	-0.454738000	-2.287299000	C	-2.537676	-0.835343	-2.319822
C	-2.550703000	-2.619583000	-0.423587000	C	-2.154435	-2.986331	-0.481644
C	-1.783829000	-3.259286000	0.632004000	C	-1.278997	-3.516005	0.549683
C	-3.267243000	-0.890306000	1.184425000	C	-3.077554	-1.391140	1.158412
C	-2.548230000	-1.513865000	2.204871000	C	-2.252787	-1.910819	2.157621
C	-1.785214000	-2.722657000	1.920477000	C	-1.328481	-2.994306	1.843797
C	-1.967964000	2.109027000	-2.174514000	C	-2.326088	1.794648	-2.208024
C	-2.577802000	2.004649000	-0.777913000	C	-2.877680	1.590867	-0.796460
C	-1.981736000	0.745052000	-3.020803000	C	-2.137487	0.440019	-3.056951
C	-3.285055000	0.909969000	-0.334360000	C	-3.397140	0.398073	-0.343127
C	-3.264970000	0.559322000	1.070923000	C	-3.294351	0.043433	1.059342
C	-1.793243000	2.768010000	0.152228000	C	-2.195248	2.459072	0.124827
C	-1.779934000	2.467355000	1.519791000	C	-2.093937	2.143730	1.487144
C	-2.536793000	1.328740000	1.986189000	C	-2.658753	0.902538	1.962512
C	0.589105000	0.908937000	-3.161485000	C	0.366853	0.990281	-3.261091
C	0.589569000	2.174404000	-2.391821000	C	0.192937	2.236610	-2.479196
C	-0.580771000	0.199815000	-3.341745000	C	-0.686265	0.114234	-3.414242
C	-0.576390000	2.631423000	-1.830541000	C	-1.021423	2.531756	-1.899952
C	-0.569857000	3.155884000	-0.495826000	C	-1.058805	3.033696	-0.551624
C	1.823017000	2.225363000	-1.633663000	C	1.426927	2.458734	-1.749019
C	1.842390000	2.766070000	-0.338221000	C	1.394420	2.968376	-0.444085
C	0.614987000	3.254266000	0.246316000	C	0.122560	3.265536	0.167672
C	-0.550341000	1.426757000	3.233680000	C	-0.672549	1.282995	3.163879
C	-0.546263000	2.563771000	2.278363000	C	-0.867716	2.405354	2.215893
C	0.626204000	2.956574000	1.657473000	C	0.210044	2.970323	1.571548
C	0.621873000	0.759219000	3.528822000	C	0.590580	0.792132	3.418369
C	-0.558627000	-1.407818000	3.431700000	C	-0.270572	-1.527011	3.345955
C	-1.787447000	-0.713401000	3.149992000	C	-1.594216	-1.014172	3.093728
C	-1.776718000	0.683086000	3.036944000	C	-1.787396	0.370096	2.993332
C	0.625394000	-0.677969000	3.599400000	C	0.796883	-0.630343	3.479424
C	1.866566000	-2.366336000	2.296273000	C	2.248036	-2.118128	2.142425
C	0.637306000	-3.127956000	2.127119000	C	1.134218	-3.045878	1.991460
C	-0.551982000	-2.656010000	2.679883000	C	-0.098886	-2.755375	2.576994
C	1.864543000	-1.165205000	3.018360000	C	2.077182	-0.930411	2.863410
C	3.336979000	-0.118964000	1.392406000	C	3.339982	0.336432	1.218498
C	3.343654000	-1.351842000	0.625640000	C	3.508153	-0.876239	0.440722
C	2.632133000	-2.469264000	1.077309000	C	2.983980	-2.089729	0.898591
C	2.638812000	-0.028400000	2.581353000	C	2.660537	0.308651	2.415182

C	1.982996000	2.420147000	2.041996000	C	1.663382	2.707431	1.952876
C	2.625373000	2.155889000	0.704991000	C	2.284550	2.480744	0.572040
C	3.329794000	0.999335000	0.428148000	C	3.152719	1.455121	0.270401
C	1.972522000	1.264831000	3.058099000	C	1.877294	1.491987	2.985677
H	6.232286000	3.006757000	1.613457000	H	6.230688	4.007821	1.426249
C	-2.764740000	1.206281000	-4.318621000	C	-3.020530	0.745144	-4.343260
C	-2.723416000	3.121104000	-3.133708000	C	-3.278394	2.638635	-3.159635
C	-2.177341000	2.621411000	-4.495402000	C	-2.722005	2.243709	-4.540431
H	-1.086252000	2.650155000	-4.568483000	H	-1.658252	2.466877	-4.666905
H	-2.615338000	3.162519000	-5.338796000	H	-3.294705	2.679704	-5.363529
C	-4.169573000	2.689840000	-3.245025000	C	-4.617972	1.928503	-3.175739
H	-2.538185000	4.169535000	-2.931742000	H	-3.284657	3.694338	-2.889920
C	-4.202457000	1.554019000	-3.947302000	C	-4.464590	0.798934	-3.879917
H	-4.940425000	3.334706000	-2.845217000	H	-5.479175	2.242557	-2.601064
H	-2.646376000	0.491228000	-5.134476000	H	-2.793345	0.066345	-5.166476
H	-5.060918000	0.923918000	-4.152917000	H	-5.182833	-0.003663	-4.005024
C	2.984717903	2.128772337	4.750763559	C	2.648807	2.218398	4.171869
H	3.304508903	1.276955337	5.346268559	H	3.139926	1.507139	4.837495
C	2.041961903	3.154064337	5.299099559	C	1.661427	3.179946	4.806989
H	1.341153903	2.977719337	6.105531559	H	1.122999	2.991298	5.728693
C	2.071815903	4.225269337	4.479528559	C	1.476768	4.189725	3.945933
H	1.408889903	5.081981337	4.518616559	H	0.755645	4.995943	4.020078
C	4.002725903	2.989527337	4.011060559	C	3.536574	3.194215	3.375787
H	4.637192903	2.442354337	3.309365559	H	4.188714	2.703712	2.647988
H	4.646950903	3.531148337	4.718258559	H	4.125194	3.850112	4.023416
C	2.996321903	3.924428337	3.360300559	C	2.340142	3.915290	2.730157
H	3.275214903	4.596838337	2.541020559	H	2.538172	4.756665	2.069150

References

(1) Solà, M.; Duran, M.; Mestres, J. Theoretical Study of the Regioselectivity of Successive 1,3-Butadiene Diels-Alder Cycloadditions to C_{60}. *J. Am. Chem. Soc.* **1996**, 118, 8920–8924.
